# Force‐Induced Ring Flipping in a Threaded Pillar[5]Arene

**DOI:** 10.1002/anie.202516485

**Published:** 2025-08-26

**Authors:** Lei Chen, Tomás Nicolás‐García, Igor Rončević, Guillaume De Bo

**Affiliations:** ^1^ Department of Chemistry University of Manchester Oxford Road Manchester M13 9PL UK

**Keywords:** Flipping, Mechanical force, Pillar[5]arene, Rotaxane, Unstoppering

## Abstract

Pillar[*n*]arenes are popular macrocycles used in various supramolecular systems and materials due to their unique host–guest properties and ease of synthesis. Their pillar shape originates from the cyclic arrangement of methylene‐bridged hydroquinone groups. They usually exist in one of two conformations where all the hydroquinone rings are oriented in the same direction (left or right), which is the source of their planar chirality. However, the controlled formation of pillar[*n*]arenes with intermediate conformations, for example, where only one ring is inverted, remains a challenge. Here we show how mechanical force can be used to reach this elusive conformation via the force‐induced flipping of a single hydroquinone ring in a pillar[5]arene‐based rotaxane. We show that the flipping motion can be controlled with the shape of the stopper and the axle, as well as with the nature of the pillar[5]arene substituents. This flipping behavior acts as a mechanical damper by slowing the scission of the polymer under tension. These results show how mechanical force can be used to access synthetically challenging supramolecular architectures, and we anticipate that this new dampening mechanism will prove useful in the formation of tougher materials.

## Graphical abstract

Mechanical force was used to flip the orientation of a hydroquinone ring in a threaded pillar[5]arene to reach an elusive conformation not accessible to other methods of activation. The flipping can be suppressed with large substituents on the axle or the macrocycle. This unique molecular motion provides a new mechanism to dissipate mechanical energy with rotaxanes.

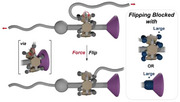

Since their discovery by Ogoshi in 2008,^[^ ^1^ ^]^ pillar[*n*]arenes have attracted considerable attention due to their unique host‐guest properties.^[^ ^2^ ^]^ Pillar[5]arene (P5) is probably the most popular member of the family, as its rigid structure and small size allow for the efficient formation of inclusion complexes and interlocked structures.^[^ ^3^ ^]^ P5 (and other pillar[*n*]arenes) is planar chiral due to the directional arrangement of its *para*‐disubstituted hydroquinone groups. P5 with small substituents (such as Me) occurs as a mixture of enantiomers, as the inversion of directionality is enabled by the oxygen‐through‐the‐annulus rotation of the hydroquinone rings.^[^ ^3^ ^]^ The inversion of chirality can be suppressed with bulky substituents^[^ ^4^ ^]^ and by threading a guest into the cavity of the macrocycle.^[^ ^5^ ^]^ The inversion can also be induced in response to external stimuli, such as temperature^[^ ^6^ ^]^ or pH^[^ ^7^ ^]^ in P5‐based pseudo[1]catenanes. However, in all cases, the rotation of one hydroquinone triggers the fast inversion of the adjacent rings in such a way that intermediate conformations are not observed.^[^ ^8^ ^]^ Consequently, the isolation of such intermediate conformers remains an unsolved synthetic challenge. The only example of single ring inversion has been observed on dihydroxypillar[5]arene, as the unsubstituted hydroquinone ring can adopt two stable conformations, in the same or opposite direction as the other rings, as both are stabilized by internal hydrogen bonding between the hydroquinone ring OH and the alkoxy groups of its two neighbours.^[^ ^9^, ^10^, ^11^ ^]^

We have explored the mechanochemistry of interlocked molecules such as rotaxanes,^[^ ^12^, ^13^ ^]^ catenanes,^[^ ^14^ ^]^ and knots.^[^ ^15^ ^]^ In this context, we found that rotaxanes built from a pillar[5]arene macrocycle are particularly effective force actuators.^[^ ^16^, ^17^, ^18^, ^19^, ^20^ ^]^ Indeed, they have been shown to activate mechanophores (force‐sensitive molecules)^[^ ^21^ ^]^ via a unique pushing activation mechanism^[^ ^16^ ^]^ whereby mechanical force is used to pull P5 along the axle, which in turn pushes against the mechanophore until activation occurs. We have demonstrated this concept with the release of multiple functional molecules, including drugs^[^ ^17^ ^]^ and fluorescent probes,^[^ ^18^ ^]^ in a direct^[^ ^16^, ^17^, ^18^ ^]^ or logic‐gated^[^ ^19^ ^]^ manner, as well as for the activation of orthogonal bonds in 4‐membered ring mechanophores.^[^ ^20^ ^]^ In these examples, the P5 macrocycle is used as a force actuator to promote the scission of covalent bonds in the mechanophore upon application of a mechanical force. Here we show how mechanical force can be used to remodel the structure of the pillar[5]arene macrocycle itself. We have demonstrated the flipping of a single disubstituted hydroquinone ring, which adopts an opposite orientation to the rest of the pillar structure (Figure 1); a transformation only possible with the help of mechanical force within the confines of a rotaxane. We have also shown that this flipping behaviour can be suppressed with larger alkoxy groups on the pillar[5]arene, or with a thicker axle next to the stopper. We anticipate that this flipping behaviour could be used as a force‐dampening mechanism in smart materials and molecular devices.

**Figure 1:**
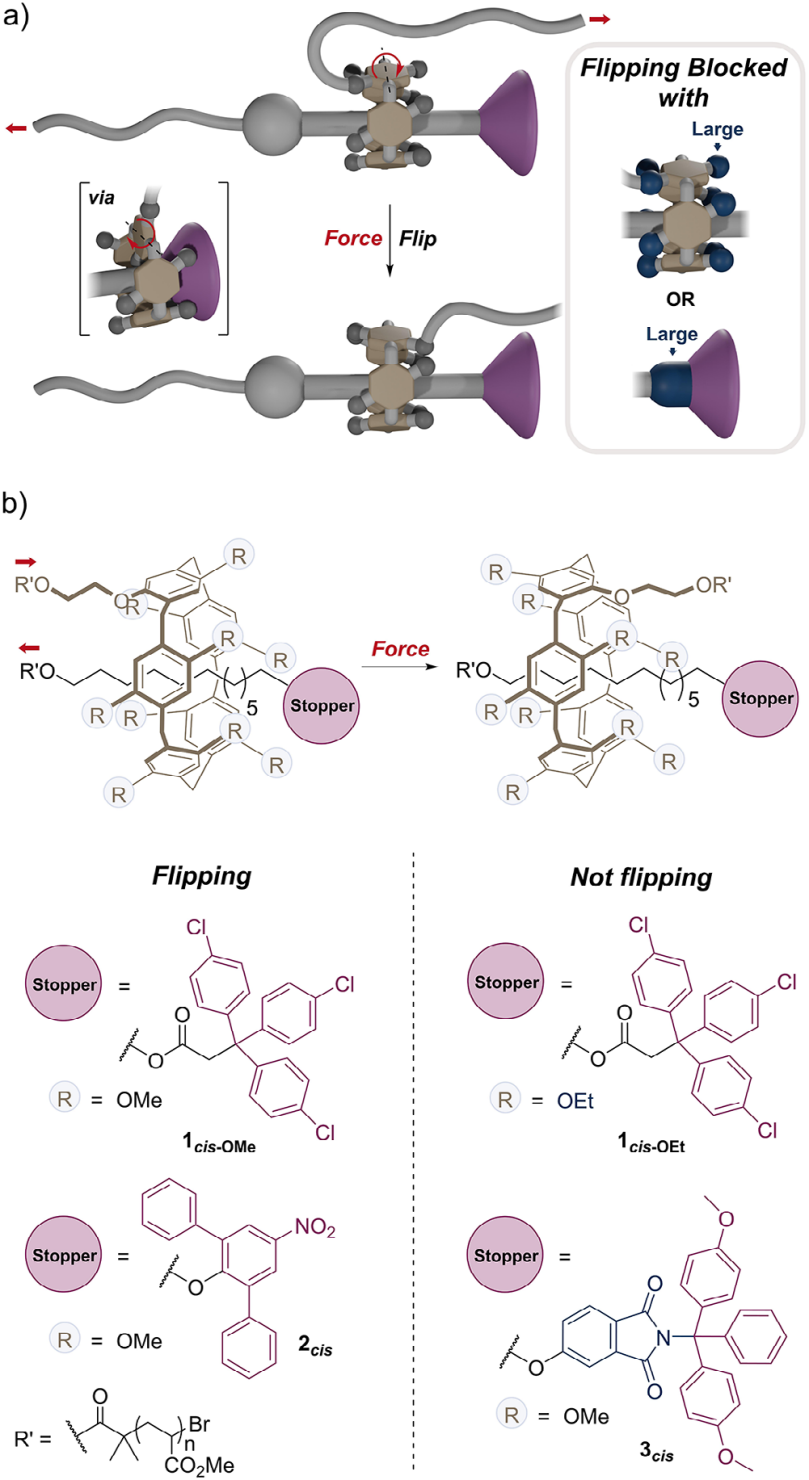
Mechanical flipping of a hydroquinone ring in a threaded pillar[5]arene. a) Cartoons illustrating the flipping process and its suppression with i) a crowded macrocycle or ii) a thick axle. b) The flipping of the hydroquinone ring connected to the polymer is observed in rotaxanes **1** *_cis_*_‐OMe_ and **2** *_cis_* but suppressed in bulkier rotaxanes **1** *_cis_*_‐OEt_ and **3** *_cis_*. Red arrows indicate the direction of the force.

Rotaxane actuators are activated by the intermediacy of polymers attached to the axle and the macrocycle, respectively. Due to the cylindrical shape of the P5 macrocycle, these polymers can be on the same or opposite side of the rotaxane, leading to two isomeric structures labelled *cis* and *trans*, respectively (Figure 2). Both isomers are competent actuators,^[^ ^17^, ^18^ ^]^ but P5 in the *cis* rotaxane experiences a greater level of deformation than its *trans* counterpart, as the pulling provokes the opening of the rim on the polymer side, notably via the rotation of the hydroquinone ring on which the polymer is attached. When the stopper is a mechanophore, the elongation of the *cis* rotaxane actuator leads to the activation of the mechanophore and the release of a small molecule.^[^ ^16^, ^17^, ^18^, ^19^, ^20^ ^]^ We hypothesised that the replacement of the mechanophore by a mechanically strong stopper would elicit the full rotation of this hydroquinone ring around its internal axis to provide a P5 with one inverted (flipped) ring (Figure 2).

**Figure 2:**
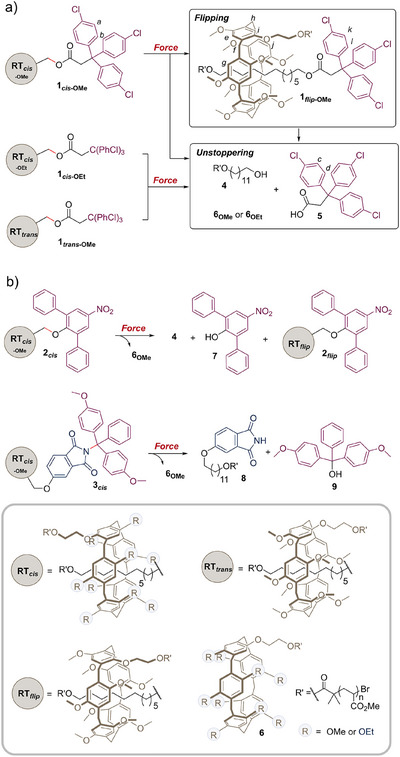
Mechanical activation of the rotaxanes investigated. a) Mechanical activation of rotaxane **1*_cis_*_‐OMe_** leads to **1*_flip_*_‐OMe_** by hydroquinone flipping. Both isomers can experience scission by unstoppering. Only the latter pathway is observed in the *trans* isomer **1*_trans_*_‐OMe_**, and in **1*_cis_*_‐OEt_**, which bears a macrocycle with larger substituents. b) Mechanophore **2*_cis_*** can also experience both flipping and unstoppering but flipping is suppressed in **3*_cis_*** due to its bulkier axle. Scissile bonds are shown in red.

To test this hypothesis, we built rotaxane **1*_cis_*_‐OMe_** from methylated P5 (R = OMe) threaded onto a C12 axle stoppered with a bulky tris(4‐chlorophenyl)methyl group we have used in previous studies,^[^ ^12^, ^13^ ^]^ and compared its mechanical behaviour to rotaxanes with stoppers of varied shapes (**2*_cis_*** and **3*_cis_***) or containing a P5 macrocycle with larger side chains (**1*_cis_*_‐OEt,_** R = OEt, Figure 1). The actuating poly(methyl acrylate) chains (PMA) were grown from the axle and the macrocycle by controlled radical polymerisation. All the macromolecular rotaxanes were synthesised following the same strategy (see Sections S3 and S4).

The mechanical activation of rotaxane **1*_cis_*_‐OMe_** (Figure 2a) was performed in MeCN/H_2_O: 50/1 at 5 °C–10 °C, using high‐intensity ultrasound (20 kHz, 13.0 W/cm^2^, 1s ON/1s OFF, 90 min), as elongational flows are generated in the immediate vicinity of collapsing ultrasound‐induced cavitation bubbles.^[^ ^22^ ^]^ SEC analysis confirms the complete scission of the initial polymers and, to some extent, of the daughter chains from the first scission (Table 1). After sonication, the solvent was removed and the solid residue washed with MeOH to extract any nonpolymeric material. The resulting polymer and MeOH fractions were then analysed separately. The presence of carboxylic acid **5** (c and d, Figure 3a, iii, iv) in the MeOH fraction indicates that some of the rotaxane cleaved via an unstoppering process,^[^ ^12^ ^]^ whereby the elongation of the rotaxane promotes the scission of the C─O bond joining the stopper to the axle, as the macrocycle pushes against the stopper (Figure 2a). This process accounts for the fate of 38% of the rotaxanes (Table 1), while the rest of the species still contain a rotaxane of some sort. However, ^1^H NMR analysis of the polymer fraction showed the emergence of new peaks (k and l, Figure 3a, ii) presenting a similar chemical shift to the peaks associated with the aromatic groups of the stopper (a and b, Figure 3a, i), which suggests that a subtle change to the structure of the rotaxane had occurred. We used 1D selective NOESY ^1^H NMR to confirm the identity of the flipped product. In native P5 macrocycles, where all the hydroquinone rings point in the same direction, the hydrogen atoms on the aromatic rings face the alkoxy groups of adjacent rings. However, if one ring is flipped, its hydrogen atoms (e and i, Figure 2a) now face the hydrogen atoms of the neighbouring aromatic rings (g and j, Figure 2a). This spatial proximity is revealed by the selective irradiation of H*_e_* and H*_i_*, which showed a clear correlation signal with aromatic protons H*_g_* and H*_j_*, and methylene bridge protons H*_f_* and H*_h_*, respectively (Figure 3b and Section S5.8). The identification of diagnostic signals of the flipped ring, allowed us to quantify each species (Table 1). In the case of rotaxane **1*_cis_*_‐OMe_**, we found that flipping was the major reaction pathway (42%), followed by unstoppering (38%). The rest was composed of intact rotaxane (20%). As all the polymer chains break during sonication, the species still containing a rotaxane are expected to be cleaved in the PMA backbone. The flipping process is irreversible in the conditions used; in fact, the flipped macrocycle was left unchanged after being heated at 85 °C for 24 h (see Section S5.8).

**Table 1:** Structural and activation parameters.

			Rotaxane activation (%)^a)^, ^b)^		
Polymer	Pre‐sonic*. M*_n_ (kDa)/Đ	Post‐sonic. *M*_n_ (kDa)/Đ^a)^	Flipping	Unstoppering	*F*_max_ ^c)^ (nN)
**1*_cis_*_‐OMe_**	145/1.16	49/1.33	42 ± 0	38 ± 1	4.5
**1*_cis_*_‐OEt_**	143/1.27	46/1.23	0	28 ± 2	4.3
**2*_cis_***	138/1.14	50/1.33	25 ± 1	52 ± 1	3.3
**3*_cis_***	117/1.19	51/1.29	0	22 ± 2	5.8
**1*_trans_*_‐OMe_**	163/1.21	51/1.30	NA	25 ± 0	4.6

^a)^ Average value from two parallel experiments. See Table S3 for details.

^b)^ See Section S6 for calculation details.

^c)^ From CoGEF calculations (unstoppering). See Section S7 for details.

**Figure 3:**
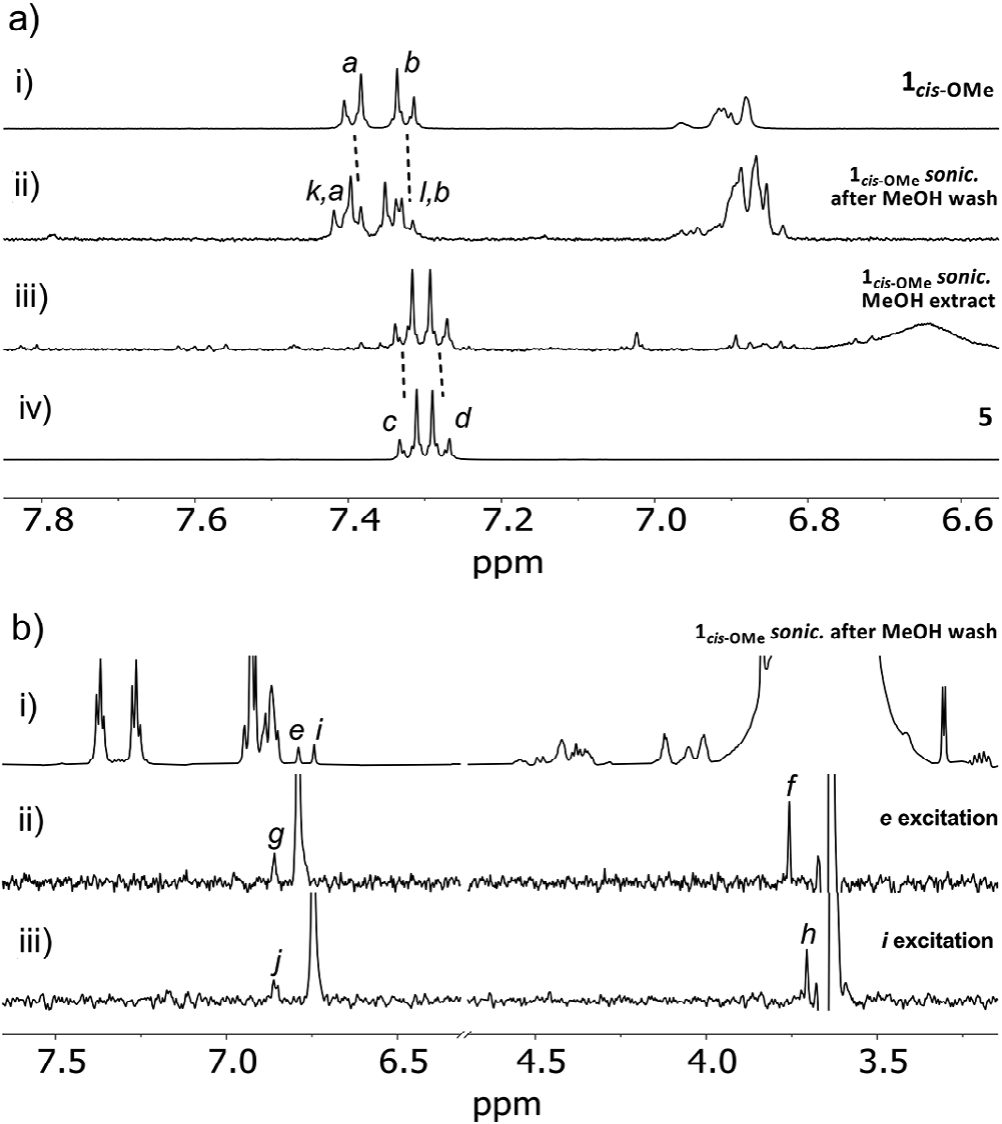
a) Partial ^1^H NMR (400 MHz, acetone‐*d_6_*, 298 K) spectra of mechanophore polymer **1*_cis_*_‐OMe_** before i) and after ii) sonication and MeOH wash, MeOH extract iii), along with reference compound **5** iv). Assignments correspond to the lettering shown in Figure 2. b) Partial ^1^H NMR (700 MHz, acetonitrile‐*d*_6_, 298 K) spectrum of mechanophore **1*_cis_*_‐OMe_** after sonication and MeOH wash i) and partial 1D selective NOESY (700 MHz, acetonitrile‐*d*_6_, 298 K) spectra of the same sample after excitation of protons H_e_ ii), and H_i_ iii).

We turned to molecular simulations to gain insight on the mechanics of the flipping process. We initially investigated the response of **1*_cis‐_*_OMe_′, 1*_trans‐_*_OMe_′**, and **1*_flip‐_*_OMe_′** to various levels of force (0–5 nN) using the external force is explicitly included (EFEI) method (see Section S7.9).^[^ ^23^ ^]^ We found that the largest difference in energy between **1*_cis‐_*_OMe_′** and **1*_flip‐_*_OMe_′** (and **1*_trans‐_*_OMe_′**) occurs at 3.75 nN. To investigate non‐statistical behaviour, we performed molecular dynamics simulations (EFEI@3.75nN, GFN2‐xTB, vac)^[^ ^24^, ^25^ ^]^ of model **1*_cis‐_*_OMe_′** at the same force (see Section S7.10). In this case, the flipping behaviour was observed in 10 of 20 trajectories (50%), nearing the experimental value of 42%. A representative trajectory is shown in Figure 4 (see also Figure S41 and Videos S1–S3). The flipping process can be visualised with the evolution of dihedral angle *ω* in **1*_cis‐_*_OMe_’**, as its value becomes negative when the orientation of the hydroquinone ring flips (Figure 4a), and from selected structures along the reaction pathways (Figure 4b). Initially, the separation of the pulling points leads to the bending of the axle and the opening of the macrocycle (Figure 4b, ii), which adopts a cone‐like shape as the frontal rim contracts and the rear rim expands following the rotation of the hydroquinone units around the contact point with the axle and at the pulling point. The effect is particularly pronounced in the latter, as this ring is held almost perpendicular to the axle until it meets the stopper. However, the ring inversion only occurs when the contact between the two subcomponents forces the alkoxy substituent inside of the macrocycle's cavity (Figure 4b, iii), which corresponds to the state of highest energy of the trajectory (Figure 4a). This suggests that the flipping behaviour will be influenced by the nature of the stopper, the axle, and the macrocycle itself, as the inversion can only occur if the alkoxy substituent has enough room to flip in the small space created between the axle and the hydroquinone ring in the elongated macrocycle. This “flipping space” can be visualised in computed (CoGEF, B3LYP/6–31G, vac) structures **1*_cis_*_‐OMe_’** and **3*_cis_*’** (Figure 4c, see Supporting Information for details), which illustrate how transitioning from a thin (**1*_cis_*_‐OMe_’**) to a thick (**3*_cis_*’**) axle should prevent the hydroquinone inversion as the flipping space is occupied by the phthalimide unit.

**Figure 4:**
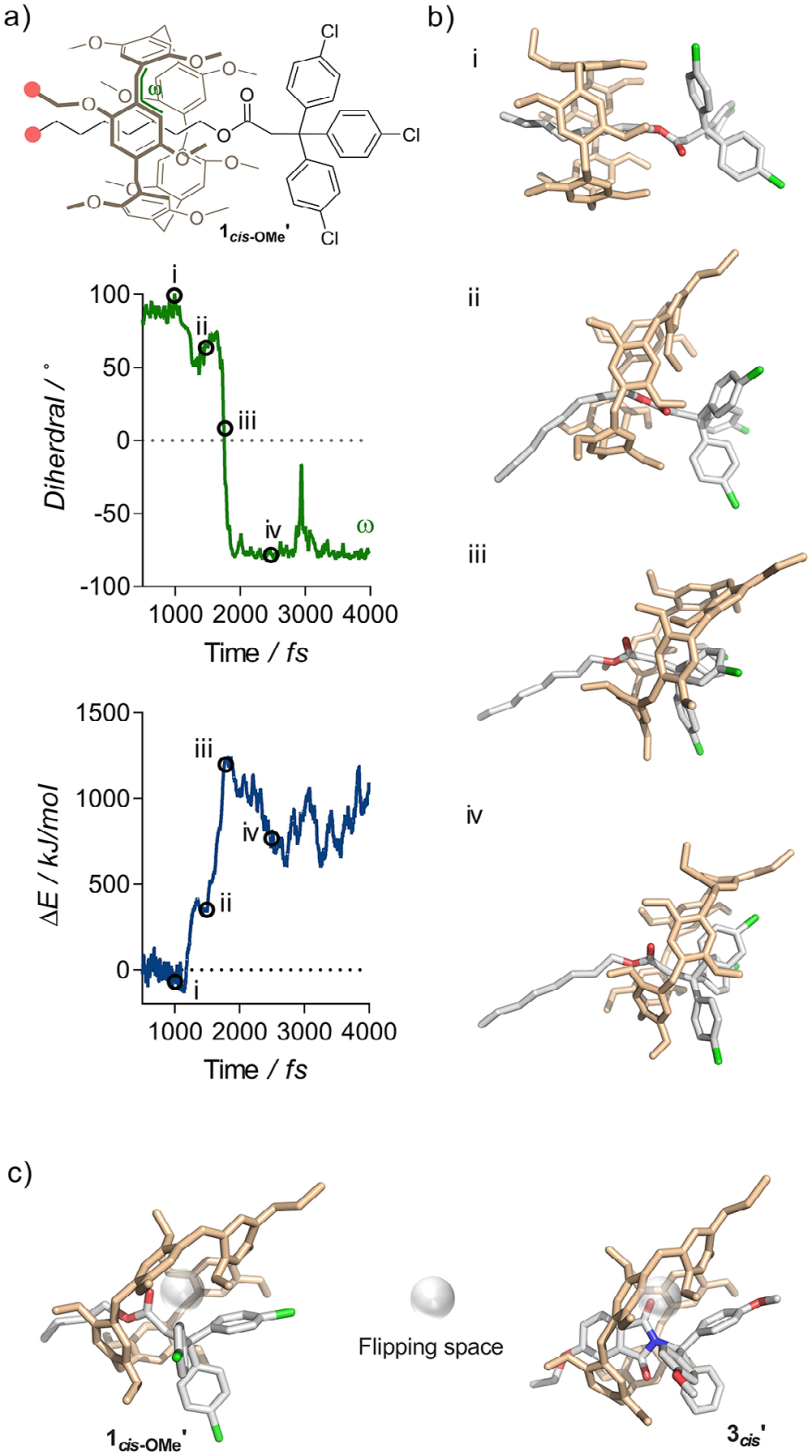
Computational investigation of hydroquinone flipping in rotaxane **1*_cis‐_*_OMe_’**. a) Representative MD (EFEI@3.75nN, GFN2‐xTB) trajectory showing the evolution of dihedral angle *ω* and free energy of rotaxane **1*_cis‐_*_OMe_’** at a constant force of 3.75 nN. Anchor atoms are indicated by the pink disks. b) Representative structures from the MD trajectory depicted. c) CoGEF structures of **1*_cis_*_‐OMe_’** and **3*_cis_*’** showing the impact of the axle's thickness on the accessibility of the “flipping space”. See Section S7 for details. Hydrogen atoms omitted for clarity.

To test this hypothesis and assess the influence of the stopper, the axle, and the macrocycle on the flipping process, we designed rotaxanes **2*_cis_*, 3*_cis_***, and **1*_cis_*_‐OEt_** respectively (Figure 1). Rotaxane **1*_cis_*_‐OMe_** displays a stopper with a steric bulk projecting away from the macrocycle and attached to a thin axle. In rotaxanes **2*_cis_*,** we kept the same axle but appended a stopper with a steric bulk projecting toward the macrocycle, and with a profile relatively flat compared to the conical shape of the stopper used in **1*_cis_*_‐OMe_**. This arrangement still permits flipping, though to a lesser extent than in **1*_cis_*_‐OMe_**, while enhancing the unstoppering process (to release phenol **7**, Figure 2b). This is potentially due to the stopper being a less effective pivot point (because of its shape) and/or the fact that this stopper is more susceptible to mechanical scission (Table 1). Flipping can be suppressed by increasing the thickness of the axle (**3*_cis_***) or the size of the alkoxy substituents on the macrocycle (**1*_cis_*_‐OEt_**), as the flipping space is then too small to allow the rotation of the hydroquinone ring as predicted above (see Figure S42). In both cases, only unstoppering (and PMA cleavage) is observed (Table 1), to release a trityl cation, observed as trityl alcohol **9**, and carboxylic acid **5,** respectively (Figure 2).

Finally, we sought to investigate the influence of the flipping process on the rate and selectivity (i.e., flipping versus unstoppering versus PMA cleavage) of the mechanochemical activation. In terms of rate, we found that polymer **1*_cis_*_‐OMe_** containing a *cis* rotaxane cleaves at a slower rate than the corresponding *trans* isomer **1*_trans_*_‐OMe_** (Figure 5a and Table S2). In the early stage of the sonication of **1*_cis_*_‐OMe_**, the flipping process is faster than scission by unstoppering (Figure 5b). However, the flipping starts to plateau after ∼10 min, while the proportion of unstoppering rises steadily over the course of the reaction to reach a similar level to flipping (Figure 5b). After 90 min of sonication, we observed that 42% of the rotaxanes had experienced flipping, 38% unstoppering, and 20% were left intact (Table 1 and Figure 5c). As all the polymer chains have undergone at least one cycle of scission, we can expect the surviving rotaxanes (flipped or not) to be broken in one of their PMA arms (Figure 5c). Considering that **1*_trans_*_‐OMe_** can also lead to unstoppering, albeit to a lower level (25%), it is likely that **1*_flip_*_‐OMe_** will display the same actuating capability due to its similar structure. This suggests that the flipped rotaxane can subsequentially undergo a scission by unstoppering or by the cleavage of one of its PMA arms. If we assume that the mechanical behaviour of **1*_flip_*_‐OMe_** is similar to that of **1*_trans_*_‐OMe_** (where 25% unstoppering is observed), we can approximate that out of the 80% of the **1*_cis_*_‐OMe_** rotaxanes which don't experience PMA scission in the first elongation event, 56% (42%/0.75, see below) should undergo a ring flip, leaving 24% to cleave by unstoppering. As **1*_flip_*_‐OMe_** is still mechanically active due to the presence of intact PMA arms, a second elongation event should lead to another 14% (0.25 × 56%) of unstoppering (tallying to the observed 38%) and 42% of flipped rotaxanes with a broken PMA arm, which match our observations (Table 1). The ability of the *cis*‐rotaxane to undergo flipping before being broken by unstoppering or PMA scission in a second elongation event explains why the rotaxanes experiencing flipping also have a higher proportion of unstoppering (**1*_cis_*_‐OMe_** and **2*_cis_***, Table 1). In effect, the flipping pathway offers a chance to the polymer to dissipate mechanical energy without breaking and acts as a kind of mechanical damper. This phenomenon was further supported by the analysis of the MD trajectories. Unstoppering was consistently observed in all trajectories for **1*_trans_*_‐OMe_** ′, **1*_flip_*_‐OMe_** ′, and **1*_cis_*_‐OEt_** ′, as well as in all **1*_cis_*_‐OMe_** ′ trajectories in which flipping was not observed (some of the flipping trajectories eventually lead to unstoppering, see Section S7).

**Figure 5:**
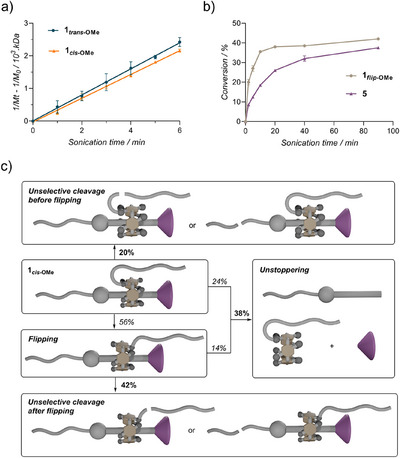
Dissociation kinetics and reaction pathways during the mechanical activation of **1*_cis_*_‐OMe_**. a) *Cis* isomer **1*_cis_*_‐OMe_** (*M_n_* = 166 kDa, *Đ* = 1.17) cleaves at a slower rate (*k_cis_* * = 36.5 ± 0.3 min^−1 ^kDa^−1^ 10^5^ and *k_trans_* * = 39.9 ± 0.5 min^−1 ^kDa^−1^ 10^5^, p = 0.03) than the corresponding *trans* isomer **1*_trans_*_‐OMe_** (*M_n_* = 163 kDa, *Đ* = 1.21). See Section S5.7 for details. b) Conversion in flipped (**1*_flip_*_‐OMe_**) or unstoppering (acid **5**) products upon sonication of **1*_cis_*_‐OMe_** (*M_n_* = 145 kDa, *Đ* = 1.16). c) Possible reaction pathways during the mechanical activation of **1*_cis_*_‐OMe_**. Italicised and bold percentages indicate estimated and measured conversions, respectively.

In conclusion, we have demonstrated the formation of an elusive pillar[5]arene isomer, which possesses an inverted hydroquinone ring. This transformation can only be realised upon application of mechanical force to a threaded macrocycle in a *cis* rotaxane actuator where both polymer arms face each other. We found that this flipping behaviour is influenced by the size and shape of the subcomponents, as it can be suppressed with a thick axle or large substituents on the rim of the pillar[5]arene. Macromolecular rotaxanes able to flip one of their aromatic rings are not only slower to break, but they are also less susceptible to cleave in their PMA arms. This leads to a higher proportion of unstoppering, whereby the terminal stopper is excised from the axle by the pushing actuation of the macrocycle. In effect, the flipping motion acts as a mechanical damper that enables the polymer to dissipate mechanical energy without breaking. This allows the flipped rotaxane to experience another elongation event, which can lead to PMA scission or unstoppering, overall increasing the proportion of unstoppering compared to rotaxanes unable to flip. This flipping behaviour provides another mechanism to dissipate mechanical energy with mechanical bonds^[^ ^26^, ^27^, ^28^, ^29^ ^]^ and should prove useful for the design of stronger materials. Finally, these results further demonstrate the unique ability of mechanical force to access molecular architectures otherwise inaccessible.

## Conflict of Interests

The authors declare no conflict of interest.

## Supporting information



Supporting Information

Supporting Information

Supporting Information

Supporting Information

## Data Availability

The data that support the findings of this study are available in the Suppporting Information of this article.
