# Supplementary material for: Force‐Induced Ring Flipping in a Threaded Pillar[5]Arene
Source: Angew Chem Int Ed Engl. 2025 Aug 26;64(41):e202516485. doi: 10.1002/anie.202516485 (PMC12501734; doi:10.1002/anie.202516485)
Supplement: Supplementary file 1 — Supporting Information [file ANIE-64-e202516485-s001.pdf]

Supporting Information

Force-Induced Ring Flipping in a Threaded Pillar[5]arene

*Lei Chen, Tomás Nicolás-García, Igor Rončević, and Guillaume De Bo**

Department of Chemistry, University of Manchester, Oxford Road, Manchester, M13 9PL, UK

*E-mail: guillaume.debo@manchester.ac.uk

1 Table of Contents

1 Table of Contents 2
2 General Experimental Details 5
3 Synthesis of Rotaxane and Control Compounds 6
3.1 Synthesis of Rotaxane Compounds 6
3.1.1 Synthesis of S2 6
3.1.2 Synthesis of S4 7
3.1.3 Synthesis of S6 7
3.1.4 Synthetic Route to Rotaxane Precursors S11 and S12 8
3.1.5 Synthesis of S8 9
3.1.6 Synthesis of S9 9
3.1.7 Synthesis of S11 and S12 10
3.1.8 Synthesis of S13 11
3.1.9 Synthesis of S14 12
3.1.10 Determination of the *cis*/*trans* Isomers of S13 and S14 12
3.2 Synthesis of Control Compounds 14
3.2.1 Synthesis of S16 14
4 Synthesis of Polymers 15
4.1 Representative Procedure for SET-LRP of Methyl Acrylate Using Mechanophore Initiators 15
4.2 Synthesis of Mechanophore Polymers 15
4.2.1 Synthesis of Polymer $1_{cis\text{-OMe-145}}$ and $1_{cis\text{-OMe-166}}$ 15
4.2.2 Synthesis of Polymer $1_{cis\text{-OEt}}$ 16
4.2.3 Synthesis of Polymer $1_{trans\text{-OMe}}$ 16
4.2.4 Synthesis of Polymer 2_{cis} 17
4.2.5 Synthesis of Polymer 3_{cis} 17
4.2.6 Synthesis of Polymer S19 18
4.3 Synthesis of Control Polymers 19
4.3.1 Synthesis of Polymer S20 19
4.3.2 Synthesis of Polymer S21 19
4.4 SEC Data for Synthesized Polymers 20
4.5 SEC Traces for Mechanophore and Control Polymers 20
5 Mechanophore Activation via Ultrasound 21
5.1 General Procedure for Sonication Experiments 21
5.2 Sonication of Mechanophore Polymer $1_{cis\text{-OMe-145}}$ 21
5.3 Sonication of Mechanophore Polymer $1_{cis\text{-OEt}}$ 25
5.4 Sonication of Mechanophore Polymer 3_{cis} 29
5.5 Sonication of Mechanophore Polymer $1_{trans\text{-OMe}}$ 32
5.6 Sonication of Mechanophore Polymer 2_{cis} 36
5.7 Kinetic Investigation 39
5.7.1 Comparison of Rate of Flipping and Unstoppering in Polymers $1_{cis\text{-OMe-145}}$ 39
5.7.2 Comparison of Dissociation Rate of Polymers $1_{cis\text{-OMe}}$ and $1_{trans\text{-OMe}}$ 40
5.8 Determination of Flipped Polymer $1_{flip\text{-OMe}}$ and the Thermal Stability 43
5.9 Sonication of Control Polymers S20 and S21 46
6 Calculation of Extent of Mechanophore Activation 49
6.1 Calculations for Polymer $1_{cis\text{-OMe}}$ 49
6.2 Calculations for Polymer 3_{cis} 51
6.3 Calculations for Polymers $1_{cis\text{-OEt}}$ and $1_{trans\text{-OMe}}$ 52
6.4 Calculations for Polymer 2_{cis} 54
6.5 Summary of Mechanical Activation 56
7 Computational modelling 58
7.1 General method 58
7.2 Computational modelling of $1_{cis\text{-OMe}}$’ 59

7.3 Computational Calculations of Model $1_{\textit{cis}\text{-OEt}}'$ 60
7.4 Computational Calculations of Model $3_{\textit{cis}}'$ 61
7.5 Computational Calculations of Model $1_{\textit{trans}\text{-OMe}}'$ 62
7.6 Computational Calculations of Model $2_{\textit{cis}}'$ 63
7.7 Computational Calculations of Model $1_{\textit{flip}\text{-OMe}}'$ 64
7.8 Comparison of Unstoppering Activation of Model $1_{\textit{trans}\text{-OMe}}'$, $1_{\textit{flip}\text{-OMe}}'$, $1_{\textit{cis}\text{-OMe}}'$ and $1_{\textit{cis}\text{-OEt}}'$ in Computational Simulation 65
7.9 Comparison of Activation Energies upon External Force of Model $1_{\textit{cis}\text{-OMe}}'$, $1_{\textit{cis}\text{-OEt}}'$, $1_{\textit{flip}\text{-OMe}}'$ and $1_{\textit{trans}\text{-OMe}}'$ 66
7.10 Molecular Dynamics Simulations 66
7.10.1 Molecular Dynamics Simulations of Model $1_{\textit{cis}\text{-OMe}}'$ 67
7.10.2 Molecular Dynamics Simulations of Model $1_{\textit{cis}\text{-OEt}}'$ 70
7.10.3 Molecular Dynamics Simulations of Model $1_{\textit{flip}\text{-OMe}}'$ 72
7.10.4 Molecular Dynamics Simulations of Model $1_{\textit{trans}\text{-OMe}}'$ 73
8 NMR Spectra 74
8.1 Small Molecule NMR Spectra 74
8.1.1 Spectra of S2 74
8.1.2 Spectra of S4 75
8.1.3 Spectra of S6 76
8.1.4 Spectra of S16 77
8.1.5 Spectra of S8 78
8.1.6 Spectra of S9 79
8.1.7 Spectra of S11 80
8.1.8 Spectra of S12 81
8.1.9 Spectra of S13 83
8.1.10 Spectra of S14 85
8.2 Polymer NMR Spectra 88
8.2.1 Spectra of polymer $1_{\textit{cis}\text{-OMe-145}}$ 88
8.2.2 Spectra of polymer $1_{\textit{cis}\text{-OEt}}$ 89
8.2.3 Spectra of polymer S19 90
8.2.4 Spectra of polymer $3_{\textit{cis}}$ 90
8.2.5 Spectra of polymer S23 91
8.2.6 Spectra of polymer $1_{\textit{trans}\text{-OMe}}$ 92
8.2.7 Spectra of polymer $2_{\textit{cis}}$ 92
8.2.8 Spectra of polymer S20 93
8.2.9 Spectra of polymer S21 94
8.2.10 Spectra of polymer 6_{OMe} 94
8.3 Post-Sonication NMR Spectra 95
8.3.1 Post-Sonication ^{1}H NMR Spectra of Polymer $1_{\textit{cis}\text{-OMe-145}}$ (Run 1) 95
8.3.2 Post-Sonication ^{1}H NMR Spectra of Polymer $1_{\textit{cis}\text{-OMe-145}}$ (Run 2) 96
8.3.3 Post-Sonication ^{1}H NMR Spectra of Polymer $1_{\textit{cis}\text{-OMe-145}}$ (Run 1 and run 2) 98
8.3.4 Post-Sonication ^{1}H NMR Spectra of Polymer $1_{\textit{cis}\text{-OMe-145}}$ (Run 3) 99
8.3.5 Post-Sonication ^{1}H NMR Spectra of Polymer $1_{\textit{cis}\text{-OMe-145}}$ (2 min sonication, Run 1) ... 100
8.3.6 Post-Sonication ^{1}H NMR Spectra of Polymer $1_{\textit{cis}\text{-OMe-145}}$ (2 min sonication, Run 2) ... 102
8.3.7 Post-Sonication ^{1}H NMR Spectra of Polymer $1_{\textit{cis}\text{-OMe-145}}$ (5 min sonication, Run 1) ... 104
8.3.8 Post-Sonication ^{1}H NMR Spectra of Polymer $1_{\textit{cis}\text{-OMe-145}}$ (5 min sonication, Run 2) ... 105
8.3.9 Post-Sonication ^{1}H NMR Spectra of Polymer $1_{\textit{cis}\text{-OMe-145}}$ (10 min sonication, Run 1) . 107
8.3.10 Post-Sonication ^{1}H NMR Spectra of Polymer $1_{\textit{cis}\text{-OMe-145}}$ (10 min sonication, Run 2) . 108
8.3.11 Post-Sonication ^{1}H NMR Spectra of Polymer $1_{\textit{cis}\text{-OMe-145}}$ (20 min sonication, Run 1) . 110
8.3.12 Post-Sonication ^{1}H NMR Spectra of Polymer $1_{\textit{cis}\text{-OMe-145}}$ (20 min sonication, Run 2) . 111
8.3.13 Post-Sonication ^{1}H NMR Spectra of Polymer $1_{\textit{cis}\text{-OMe-145}}$ (40 min sonication, Run 1) . 113
8.3.14 Post-Sonication ^{1}H NMR Spectra of Polymer $1_{\textit{cis}\text{-OMe-145}}$ (40 min sonication, Run 2) . 114
8.3.15 Post-Sonication ^{1}H NMR Spectra of Polymer $1_{\textit{cis}\text{-OEt}}$ (Run 1) 116
8.3.16 Post-Sonication ^{1}H NMR Spectra of Polymer $1_{\textit{cis}\text{-OEt}}$ (Run 2) 117

8.3.17 Post-Sonication ^1H NMR Spectra of Polymer 3_{cis} (Run 1) 119
8.3.18 Post-Sonication ^1H NMR Spectra of Polymer 3_{cis} (Run 2) 120
8.3.19 Post-Sonication ^1H NMR Spectra of Polymer 3_{cis} (Run 1 and run 2) 122
8.3.20 Post-Sonication ^1H NMR Spectra of Polymer $1_{trans\text{-OMe}}$ (Run 1) 122
8.3.21 Post-Sonication ^1H NMR Spectra of Polymer $1_{trans\text{-OMe}}$ (Run 2) 124
8.3.22 Post-Sonication ^1H NMR Spectra of Polymer $1_{trans\text{-OMe}}$ (Run 3) 125
8.3.23 Post-Sonication ^1H NMR Spectra of Polymer 2_{cis} (Run 1) 127
8.3.24 Post-Sonication ^1H NMR Spectra of Polymer 2_{cis} (Run 2) 128
8.3.25 Post-Sonication ^1H NMR Spectra of Polymer 2_{cis} (Run 1 and run 2) 130
8.3.26 Post-Sonication ^1H NMR Spectra of Polymer S20 130
8.3.27 Post-Sonication ^1H NMR Spectra of Polymer S21 132
9 Mass Spectrometry Isotopic Patterns 134
9.1 Isotopic distribution of S2 134
9.2 Isotopic distribution of S4 134
9.3 Isotopic distribution of S6 135
9.4 Isotopic distribution of S16 135
9.5 Isotopic distribution of S9 136
9.6 Isotopic distribution of S11 136
9.7 Isotopic distribution of S12 137
9.8 Isotopic distribution of S13 137
9.9 Isotopic distribution of S14 138
10 References 139

2 General Experimental Details

Unless otherwise stated, all reagents and solvents were purchased from commercial suppliers and used without further purification. Dry solvents were obtained by passing through an activated alumina column on a Phoenix SDS solvent drying system (JC Meyer Solvent Systems, CA, USA). Compounds **S1**, **S3**, **S5**, **S15**, **S17**, **S18**, **S10**, **S7** and polymers **S23**, **S21**, **6_{OMe}** were prepared according to literature procedures.[1,2] Polymers **S23**, **S21** and **6_{OMe}** were reused in this study.

Size exclusion chromatography (SEC) analyses were performed in THF solution (1.0 mg mL^{-1}) at 40 °C using a GPC/SEC Agilent 1260 Infinity II with 2 × PL gel 10 µm mixed-C and a PL gel 500 Å column and equipped with a differential refractive index (DRI) detector employing narrow polydispersity polystyrene standards (Agilent Technologies) as a calibration reference. Samples were filtered through a Whatman Puradisc 4 mm syringe filter with 0.45 µm PTFE membrane before injection to equipment, and experiments were carried out with injection volume of 50 µL, flow rate of 1 mL min^{-1}. Results were analyzed using *n*-dodecane as an internal marker using Agilent GPC/SEC Software Version 2.2.

Ultrasound experiments were performed using a Sonics VCX 500 ultrasonic processor equipped with a 13 mm diameter solid or replaceable-tip probe. The distance between the titanium tip and the bottom of the Suslick cell was 2 cm. The ultrasonic intensity was calibrated using the method outlined by Hickenboth *et al*.[3] The Suslick cells were fabricated by the Department of Chemistry glass workshop at the University of Manchester.

Analytical TLC was performed on precoated silica gel plates (0.25 mm thick, 60 F254, Merck, Germany) and observed under UV light or stained with a potassium permanganate base solution. Preparative TLC was performed on precoated silica gel plates: 500 µm or 2000 µm, UNIPLATE GF, Analtech Inc., DE, USA. Flash column chromatography was performed with silica gel 60 (230-400 mesh) from Sigma-Aldrich. ^{1}H and ^{13}C NMR spectra were recorded on a Bruker Avance III 700 MHz Prodigy instrument, a Bruker Avance III 500 MHz Prodigy instrument or a Bruker Avance III 400 MHz Prodigy instrument. Chemical shifts are reported in parts per million (ppm) from high to low frequency and referenced to the residual solvent resonance. Coupling constants (*J*) are reported in Hertz (Hz) and splitting patterns are designated as follows: b = broad, s = singlet, d = doublet, t = triplet, q = quartet, p = pentet and m = multiplet. ^{1}H and ^{13}C assignments were made using 1D or 2D NMR methods (HSQC, HMBC, COSY). Mass spectra were obtained through the Mass Spectrometry services in the Department of Chemistry at the University of Manchester.

Abbreviations: BiBB: bromoisobutyryl bromide; BTBSCl: 3,5-bis(trifluoromethyl)benzenesulfonyl chloride; CoGEF: constrained geometries simulate external force; DCM: dichloromethane; DMSO: dimethylsulfoxide; ESI: electrospray ionization; HRMS: high resolution mass spectrometry; MA: methyl acrylate; MS: mass spectrometry; Me_6TREN: tris[2-(dimethylamino)ethyl]amine; PE: petroleum ether; PMDETA: N,N,N′,N′ ′,N′ ′-Pentamethyldiethylenetriamine; THF: tetrahydrofuran; TLC: thin layer chromatography.

3 Synthesis of Rotaxane and Control Compounds

3.1 Synthesis of Rotaxane Compounds

3.1.1 Synthesis of S2

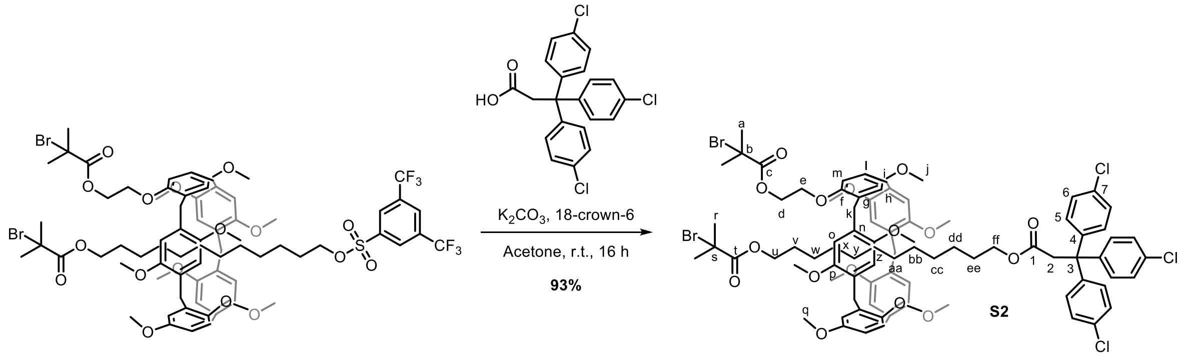

S1

To a solution of 3,3,3-Tris(4-chlorophenyl)propionic acid (4 mg, 10 µmol, 1.0 eq.) in acetone (1 mL) was added K_2CO_3 (1 mg, 10 µmol, 1.0 eq.) and 18-crown-6 (3 mg, 10 µmol, 1.0 eq.). The mixture was stirred for 2 h at room temperature. **S1** (15 mg, 10 µmol, 1.0 eq.) was added and the mixture was stirred for a further 16 h at room temperature. The solution was filtered, and the filtrate concentrated under vacuum. The residue was purified by preparative TLC (500 µm, PE/EtOAc, 8/1, eluted twice) to yield **S2** as a white powder (15 mg, 9 µmol, 93% yield).

^{1}H NMR (400 MHz, Acetone-d_6, 298 K) δ = 7.41 – 7.35 (m, 6H, H_6), 7.35 – 7.28 (m, 6H, H_5), 7.01 – 6.85 (m, 10H, $H_{h,m,o}$), 4.83 (ddd, J = 12.1, 9.4, 2.8 Hz, 1H, H_d), 4.46 (dt, J = 11.9, 2.9 Hz, 1H, H_d), 4.35 (dt, J = 10.8, 3.0 Hz, 1H, H_e), 4.14 – 4.05 (m, 1H, H_e), 3.89 – 3.67 (m, 39H, $H_{2,j,k,q}$), 3.66 – 3.60 (m, 2H, H_u), 3.46 (t, J = 7.4 Hz, 2H, H_{ff}), 1.98 (s, 6H, H_r), 1.98 – 1.96 (s, s, 6H, H_a), 0.79 – 0.67 (m, 4H, $H_{aa,ee}$), 0.67 – 0.51 (m, 6H, $H_{v,bb,x}$), 0.24 – 0.12 (m, 2H, H_{cc}), 0.11 – 0.02 (m, 2H, H_y), 0.02 – -0.09 (m, 2H, H_{dd}), -0.71 – -0.84 (m, 4H, $H_{w,z}$).
^{13}C NMR (101 MHz, Acetone-d_6, 298 K) δ = δ 172.06, 172.03 ($C_{c,t}$), 170.72 (C_1), 151.60, 151.15, 151.10, 151.07, 151.03, 151.00 ($C_{i,p}$), 149.94 (C_f), 145.93 (C_4), 132.80 (C_7), 131.76 (C_5), 129.52, 128.85, 128.81, 128.69, 128.67, 128.66, 128.57, 128.55 ($C_{6,g,l,n}$), 115.40, 114.02, 113.78, 113.72, 113.62, 113.57, 113.52, 113.44 ($C_{h,m,o}$), 67.59 (C_u), 67.23 (C_e), 65.56 (C_{ff}), 65.45 (C_d), 57.49 (C_s), 57.11 (C_b), 55.92, 55.75, 55.71, 55.65, 55.58 ($C_{j,q}$), 55.42 (C_3), 46.11 (C_2), 31.62 (C_{aa}), 31.17, 31.14, 31.12, 31.06, 30.99 ($C_{a,r,bb,y,z}$), 30.46 – 29.21 ($C_{k,cc}$, overlapped with solvent peak), 28.98, 28.95, 28.90 ($C_{x,v,ee}$), 25.45 (C_{dd}), 24.53 (C_w).
HRMS-ESI(+): 1687.4412 $[M+Na]^+$, calculated for $C_{87}H_{101}Br_2Cl_3O_{16}Na^+$: 1687.4414.

3.1.2 Synthesis of S4

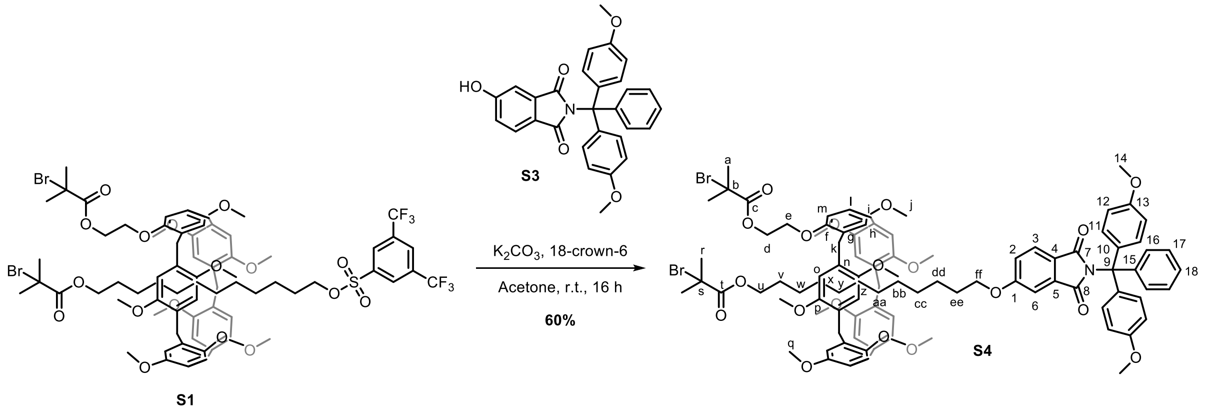

To a solution of **S3** (3 mg, 6 µmol, 1.0 eq.) in acetone (1 mL) was added K_2CO_3 (1 mg, 6 µmol, 1.0 eq.) and 18-crown-6 (2 mg, 6 µmol, 1.0 eq.). The mixture was stirred for 2 h at room temperature. **S1** (9 mg, 10 µmol, 1.0 eq.) was added and the mixture was stirred for a further 16 h at room temperature. The solution was filtered, and the filtrate concentrated under vacuum. The residue was purified by preparative TLC (500 µm, PE/ acetone, 4/1, eluted twice) to yield **S4** as a white powder (6 mg, 6 µmol, 60% yield).

^{1}H NMR (500 MHz, Acetone-d_6, 298 K) δ = 7.72 – 7.69 (m, 1H, H_3), 7.53 – 7.50 (m, 2H, H_{16}), 7.50 – 7.45 (m, 4H, H_{11}), 7.28 – 7.22 (m, 4H, $H_{17,6,2}$), 7.17 – 7.12 (m, 1H, H_{18}), 7.01 – 6.90 (m, 10H, $H_{h,m,o}$), 6.86 – 6.80 (m, 4H, H_{12}), 4.82 (ddd, J = 12.1, 9.3, 3.0 Hz, 1H, H_d), 4.47 (dt, J = 11.9, 3.1 Hz, 1H, H_d), 4.33 (dt, J = 10.9, 3.2 Hz, 1H, H_e), 4.13 (ddd, J = 10.9, 9.3, 3.0 Hz, 1H, H_e), 3.92 (t, J = 7.3 Hz, 2H, H_u), 3.89 – 3.64 (m, 43H, $H_{j,k,q,14}$), 3.17 – 3.03 (m, 2H, H_{ff}), 2.00 – 1.95 (m, 12H, $H_{a,r}$), 1.19 – 1.13 (m, 2H, H_v), 0.92 – 0.78 (m, 4H, $H_{z,aa}$), 0.73 – 0.64 (m, 2H, H_y), 0.49 – 0.38 (m, 2H, H_{bb}), 0.35 – 0.22 (m, 4H, $H_{w,x}$), 0.20 – 0.09 (m, 2H, H_{ee}), -0.28 – -0.40 (m, 2H, H_{cc}), -0.80 – -0.92 (m, 2H, H_{dd}).
^{13}C NMR (126 MHz, Acetone-d_6, 298 K) δ = δ 172.07, 171.97 ($C_{c,t}$), 168.81 (C_8), 168.51 (C_7), 165.43 (C_1), 159.01 (C_{13}), 151.74, 151.29, 151.28, 151.25, 151.21, 151.18, 151.16 ($C_{i,p}$), 150.10 (C_f), 145.27 (C_{15}), 136.48 (C_{10}), 135.77 (C_5), 130.93 (C_{11}), 129.62, 129.16, 129.08, 129.05, 129.01, 128.96, 128.93, 128.80, 128.79, 128.72, 128.69 ($C_{g,l,n,16}$), 128.18 (C_{17}), 126.74 (C_{18}), 125.34 (C_3), 124.75 (C_4), 121.63 (C_2), 115.52, 114.36, 114.32, 114.15, 114.10, 114.03, 113.67, 113.59, 113.53 ($C_{h,m,o}$), 113.50 (C_{12}), 108.17 (C_6), 73.49 (C_9), 70.31 (C_{ff}), 67.29 (C_e), 67.11 (C_u), 65.35 (C_d), 57.54, 57.10 ($C_{s,b}$), 56.13, 56.05, 55.94, 55.76, 55.71, 55.48, 55.43 ($C_{j,q,14}$), 31.57 (C_{aa}), 31.28, 31.15, 31.10, 31.08, 31.07, 31.00 ($C_{a,r,bb,y,z}$), 30.44 – 29.29 ($C_{k,cc,x}$, overlapped with solvent peak), 29.09 (C_v), 28.88 (C_{ee}), 25.61 (C_w), 24.40 (C_{dd}).
HRMS-ESI(+): 1748.5920 $[M+Na]^+$, calculated for $C_{95}H_{109}Br_2NO_{19}Na^+$: 1748.5853.

3.1.3 Synthesis of S6

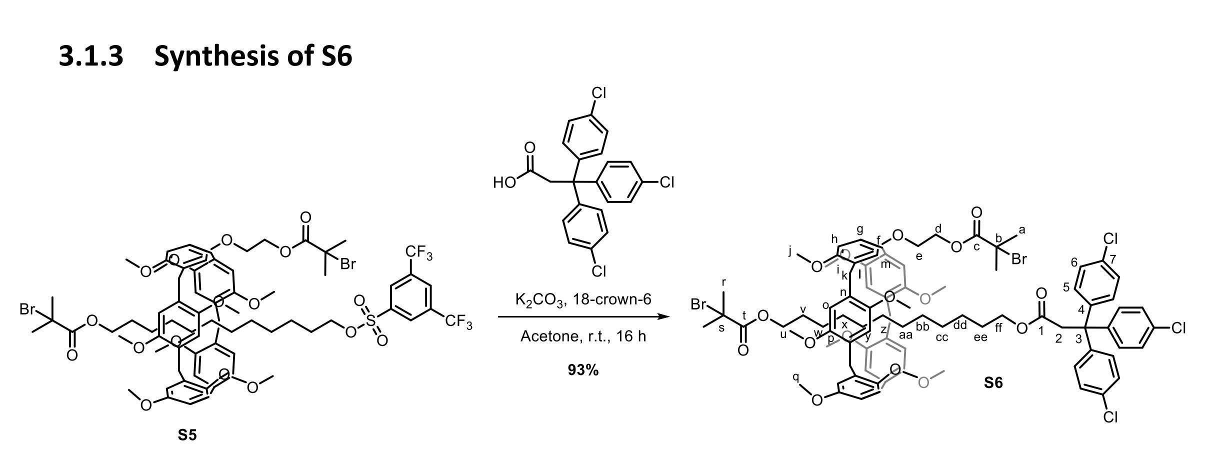

To a solution of 3,3,3-Tris(4-chlorophenyl)propionic acid (2 mg, 6 µmol, 1.0 eq.) in acetone (1 mL) was added K_2CO_3 (1 mg, 6 µmol, 1.0 eq.) and 18-crown-6 (2 mg, 6 µmol, 1.0 eq.). The mixture was stirred

for 2 h at room temperature. **S5** (9 mg, 10 µmol, 1.0 eq.) was added and the mixture was stirred for a further 16 h at room temperature. The solution was filtered, and the filtrate concentrated under vacuum. The residue was purified by preparative TLC (500 µm, DCM/EtOAc, 10/1, eluted three times) to yield **S6** as a white powder (9 mg, 5 µmol, 93% yield).

^{1}H NMR (500 MHz, Acetone-d_6, 298 K) δ = 7.41 – 7.36 (m, 6H, H_6), 7.35 – 7.30 (m, 6H, H_5), 6.98 – 6.87 (m, 10H, $H_{h,m,o}$), 4.76 (ddd, J = 12.1, 9.5, 2.8 Hz, 1H, H_d), 4.33 (dt, J = 11.9, 2.9 Hz, 1H, H_d), 4.27 (dt, J = 10.9, 3.0 Hz, 1H, H_e), 4.07 (ddd, J = 10.8, 9.5, 2.7 Hz, 1H, H_e), 3.87 (s, 2H, H_2), 3.82 – 3.67 (m, 39H, $H_{j,k,q,u}$), 3.39 – 3.32 (m, 2H, H_{ff}), 1.99 – 1.96 (m, 12H, $H_{a,r}$), 0.85 – 0.81 (m, 2H, H_v), 0.72 – 0.56 (m, 4H, $H_{aa,z}$), 0.55 – 0.46 (m, 2H, H_{ee}), 0.44 – 0.34 (m, 2H, H_{bb}), 0.30 – 0.21 (m, 2H, H_y), -0.11 – -0.22 (m, 2H, H_{cc}), -0.30 – -0.47 (m, 6H, $H_{w,x,dd}$).
^{13}C NMR (126 MHz, Acetone-d_6, 298 K) δ = 172.12 (C_c), 171.89 (C_t), 170.85 (C_1), 151.53, 151.12, 151.04, 151.00 ($C_{i,p}$), 149.94 (C_f), 145.96 (C_4), 132.81 (C_7), 131.77 (C_5), 129.42, 128.83, 128.79, 128.69, 128.67, 128.63, 128.59, 128.54 ($C_{g,l,n,6}$), 115.28, 114.03, 113.68, 113.61, 113.50, 113.46 ($C_{h,m,o}$), 67.35 (C_u), 67.07 (C_e), 65.75 (C_{ff}), 65.50 (C_d), 57.50 (C_s), 57.11 (C_b), 55.78, 55.74, 55.69, 55.66, 55.59, 55.58 ($C_{j,q}$), 55.34 (C_3), 46.03 (C_2), 31.56 (C_{aa}), 31.19, 31.12, 31.10, 31.08, 31.06, 31.00 ($C_{a,r,y,z,bb}$), 30.35 – 29.33 ($C_{k,cc}$, overlapped with solvent peak), 29.23 (C_x), 29.01 (C_v), 28.90 (C_{ee}), 25.02 (C_w), 24.96 (C_{dd}).
HRMS-ESI(+): 1687.4389 $[M+Na]^+$, calculated for $C_{87}H_{101}Br_2Cl_3O_{16}Na^+$: 1687.4414.

3.1.4 Synthetic Route to Rotaxane Precursors S11 and S12

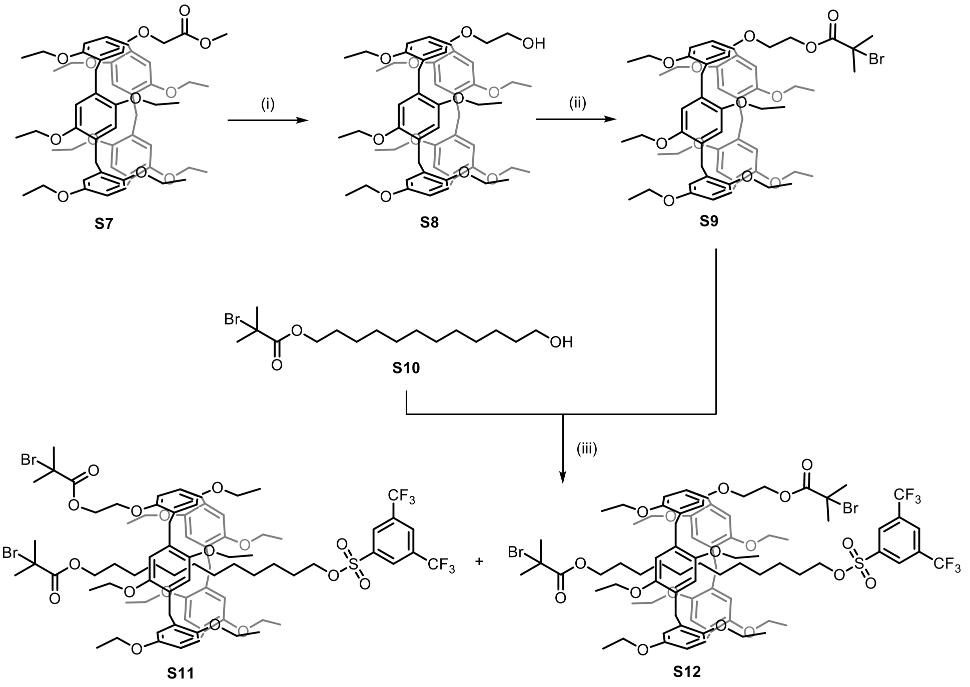

Scheme S1. Synthetic routes to rotaxane precursors **S11** and **S12**. Conditions: (i) $LiAlH_4$, THF, 65 °C, 16 h, 90% yield; (ii) BiBB, Et_3N, DCM, r.t., 16 h, 93% yield; (iii) BTBSCl, Et_3N, $CHCl_3$, -15 °C, 2 h, 26% and 16% yields for **S11** and **S12** respectively.

3.1.5 Synthesis of S8

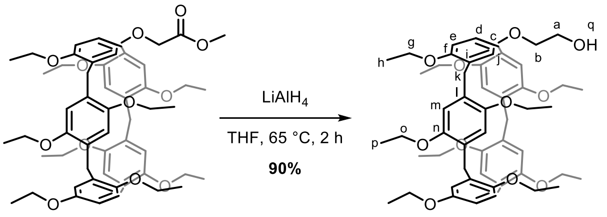

S7 S8

To a solution of **S7** (400 mg, 428 µmol, 1.0 eq.) in dry THF (30 mL), cooled in an ice bath, was added $LiAlH_4$ solution (1 M in THF, 1.7 mL, 1700 µmol, 4.0 eq.). The mixture was stirred for 2 h at 65 °C. After the solution was cooled to r.t., 0.5 mL 10% aqueous NaOH solution was added. The resulting mixture was dried over magnesium sulfate, filtered, and concentrated under vacuum to give **S8** as a white powder (350 mg, 386 µmol, 90% yield).

^{1}H NMR (500 MHz, Acetone-*d*$_6$, 298 K) δ = 6.98 – 6.86 (m, 10H, $H_{e,j,m}$), 3.98 – 3.89 (m, 22H, $H_{a,b,g,o}$), 3.78 – 3.70 (m, 10H, H_k), 1.45 – 1.35 (m, 27H, $H_{h,p}$).
^{13}C NMR (126 MHz, Acetone-*d*$_6$, 298 K) δ = 150.58, 150.56, 150.44, 150.43, 150.40, 150.39, 150.32 ($C_{c,f,n}$), 129.51, 129.30, 129.19, 129.16, 129.15, 129.14, 129.09, 129.04 ($C_{d,i,l}$), 115.34, 115.17, 115.15, 115.12, 115.08 ($C_{e,j,m}$), 70.93, 70.91, 64.23, 64.17, 61.90, 61.78 ($C_{a,b,g,o}$), 30.63 – 29.30 (C_k, overlapped with solvent signal), 15.63, 15.60 ($C_{h,p}$).
HRMS-APCI (+): 907.4948 $[M+H]^+$, calculated for $C_{55}H_{70}O_{11}H^+$: 907.4991.

3.1.6 Synthesis of S9

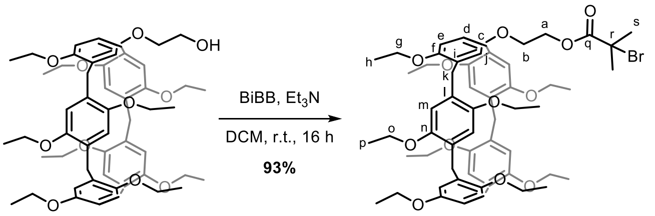

S8 S9

To a solution of **S8** (350 mg, 386 µmol, 1.0 eq.) in DCM (25 mL), cooled in an ice bath, was added BiBB (264 mg, 1158 µmol, 3.0 eq.) and Et_3N (117 mg, 1158 µmol, 3.0 eq.). The mixture was stirred for 2 h at room temperature before being concentrated under vacuum. The residue was purified by flash column chromatography (SiO_2, PE/EtOAc, 10/1) to yield **S9** as a white powder (380 mg, 360 µmol, 93% yield).

^{1}H NMR (500 MHz, Acetone-*d*$_6$, 298 K) δ = 6.98 – 6.85 (m, 10H, $H_{e,j,m}$), 4.62 – 4.57 (m, 2H, H_a), 4.19 – 4.15 (m, 2H, H_b), 3.98 – 3.89 (m, 18H, $H_{g,o}$), 3.77 – 3.70 (m, 10H, H_k), 1.97 (s, 6H, H_s), 1.45 – 1.37 (m, 27H, $H_{h,p}$).
^{13}C NMR (126 MHz, Acetone-*d*$_6$, 298 K) δ = 171.88 (C_c), 151.53, 151.00, 150.97, 150.95, 150.90 ($C_{i,p}$), 149.58 (C_f), 129.17, 129.12, 128.57, 128.47, 128.42, 128.36, 128.32, 128.25, 128.21, 128.12 ($C_{g,l,n}$), 116.05, 114.34, 114.32, 114.29, 114.25, 114.20, 114.12 ($C_{h,m,o}$), 66.91 (C_e), 64.73 (C_d), 56.08, 56.02, 55.98, 55.95, 55.90, 55.84, 55.80 (C_j), 55.66 (C_b), 30.89 (C_a), 29.94, 29.87, 29.73, 29.70 (C_k).
^{13}C NMR (126 MHz, Acetone) δ 172.04 (C_q), 150.97, 150.44, 150.42, 149.94 ($C_{c,f,n}$), 129.78, 129.32, 129.24, 129.23, 129.18, 129.05, 129.02 ($C_{d,i,l}$), 115.96, 115.35, 115.19, 115.16, 115.14, 115.12, 115.10 ($C_{e,j,m}$), 67.34 (C_a), 65.47 (C_b), 64.31, 64.24, 64.21, 64.19 ($C_{g,o}$), 57.13 (C_r), 31.05 (C_s),30.65 – 29.30 (C_k, overlapped with solvent signal), 15.69, 15.62, 15.59, 15.55 ($C_{h,p}$).
HRMS-APCI (+): 1055.4526 $[M+H]^+$, calculated for $C_{59}H_{75}BrO_{12}H^+$: 1055.4515.

3.1.7 Synthesis of S11 and S12

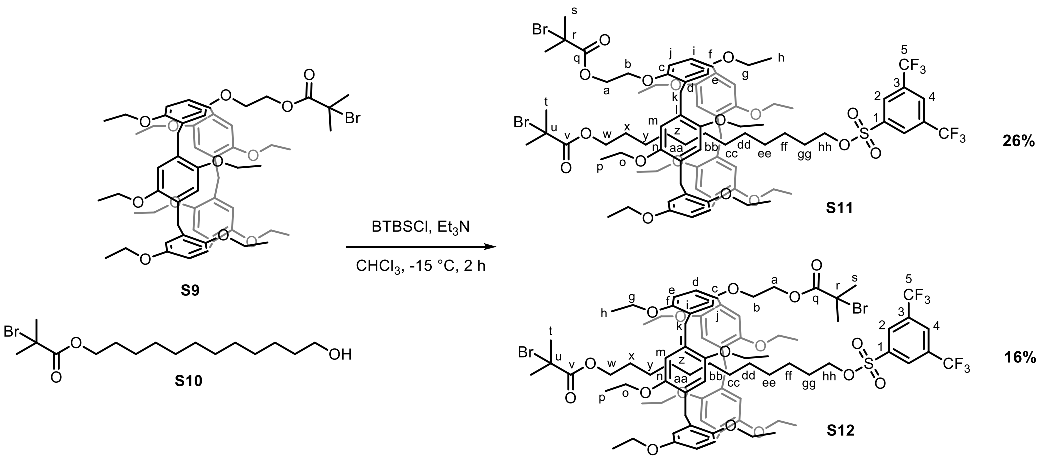

A solution of **S10** (30 mg, 86 µmol, 1.0 eq.) and **S9** (361 mg, 343 µmol, 3.0 eq.) in dry $CHCl_3$ (0.8 mL) was stirred at room temperature for 5 h. The reaction mixture was cooled in a brine and ice bath and a solution of BTBSCl (53 mg, 171 µmol, 2.0 eq.) and Et_3N (22 mg, 214 µmol, 2.5 eq.) in dry $CHCl_3$ (0.5 mL) was added dropwise. The mixture was then stirred for 2 h. The reaction mixture was neutralized by addition of an aqueous 0.2 M HCl solution before being extracted with DCM (3 x 10 mL). The organic phases were combined and dried over magnesium sulfate. The solvent was removed under vacuum and the residue purified by flash column chromatography (SiO_2, PE/EtOAc, 30/1 to 15/1) to yield **S11** (37 mg, 22 µmol, 26% yield) and **S12** (23 mg, 14 µmol, 16% yield) as white powders.

Compound **S11**:

^{1}H NMR (500 MHz, Acetone-d_6, 298 K) δ = 8.62 (s, 1H, H_4), 8.53 (s, 2H, H_2), 7.02 – 6.87 (m, 10H, $H_{e,j,m}$), 4.82 (ddd, J = 12.2, 9.3, 3.1 Hz, 1H, H_a), 4.47 (dt, J = 11.9, 3.2 Hz, 1H, H_a), 4.34 (dt, J = 10.7, 3.3 Hz, 1H, H_b), 4.12 (ddd, J = 10.6, 9.3, 3.1 Hz, 1H, H_b), 4.09 – 3.64 (m, 32H, $H_{g,o,w,hh}$), 2.00 (s, 6H, H_t), 1.98 – 1.95 (s, s, 6H, H_s), 1.55 – 1.41 (m, 27H, $H_{h,p}$), 1.19 – 1.12 (m, 2H, H_{gg}), 0.81 – 0.72 (m, 2H, H_x), 0.62 – 0.54 (m, 2H, H_{cc}), 0.51 – 0.43 (m, 2H, H_{bb}), 0.39 – 0.30 (m, 2H, H_{dd}), 0.19 – 0.09 (m, 2H, H_{ff}), 0.07 – 0.00 (m, 2H, H_{aa}), 0.00 – -0.07 (m, 2H, H_{ee}), -0.54 – -0.64 (m, 2H, H_y), -0.65 – -0.74 (m, 2H, H_z).
^{13}C NMR (126 MHz, Acetone-d_6, 298 K) δ = 172.06, 172.03 ($C_{q,v}$), 150.94, 150.49, 150.44, 150.43, 150.41, 150.36, 150.33, 149.91 ($C_{c,f,n}$), 140.47 (C_1), 133.76 (q, J = 34.5 Hz, C_3), 129.53, 129.14 (q, J = 4.2 Hz), 129.00, 128.98, 128.90, 128.77, 128.70, 128.66 ($C_{2,4,i,d,l}$), 123.61 (q, J = 272.8 Hz, C_5), 115.26, 114.74, 114.69, 114.65, 114.55, 114.53, 114.44, 114.34, 114.31 ($C_{e,j,m}$), 73.57 (C_{hh}), 67.65 (C_w), 67.04 (C_b), 65.41 (C_a), 63.98, 63.94, 63.81 ($C_{g,o}$), 57.40 (C_u), 57.07 (C_r), 31.33, 31.30, 31.25, 31.19, 31.07, 31.05, 30.93 ($C_{s,t,aa\text{-}dd}$), 30.45 – 29.28 ($C_{k,gg,ee}$, overlapped with solvent signal), 28.93 (C_z), 28.83 (C_x), 25.15 (C_{ff}), 24.91 (C_y), 16.03, 15.98, 15.96, 15.79, 15.75 ($C_{h,p}$).
^{19}F NMR (471 MHz, Acetone-d_6, 298 K) δ = -63.41 (s, 6F, F_5).
HRMS-ESI (+): 1680.5543 $[M]^+$, calculated for $C_{83}H_{108}Br_2F_6O_{17}S^+$: 1680.5573.

Compound **S12**:

^{1}H NMR (500 MHz, Acetone-d_6, 298 K) δ = 8.64 (s, 1H, H_4), 8.54 (s, 2H, H_2), 7.02 – 6.88 (m, 10H, $H_{e,j,m}$), 4.80 (ddd, J = 12.1, 9.2, 3.1 Hz, 1H, H_a), 4.47 (dt, J = 11.9, 3.2 Hz, 1H, H_a), 4.31 (dt, J = 10.8, 3.3 Hz, 1H, H_b), 4.13 (ddd, J = 10.8, 9.2, 3.0 Hz, 1H, H_b), 4.08 – 3.70 (m, 32H, $H_{g,o,w,hh}$), 1.99 (s, 6H, H_t), 1.98 – 1.95 (s, s, 6H, H_s), 1.54 – 1.39 (m, 27H, $H_{h,p}$), 1.02 – 0.92 (m, 4H, $H_{gg,x}$), 0.56 – 0.45 (m, 4H, $H_{bb,cc}$), 0.26 – 0.18 (m, 2H, H_a), 0.18 – 0.11 (m, 2H, H_{dd}), -0.15 – -0.25 (m, 4H, $H_{y,ff}$), -0.28 – -0.35 (m, 2H, H_z), -0.35 – -0.42 (m, 2H, H_{ee}).

^{13}C NMR (126 MHz, Acetone-d_6, 298 K) δ = 172.03 (C_v), 171.94 (C_q), 151.03, 150.54, 150.52, 150.48, 150.44, 150.37, 150.34, 149.86 ($C_{c,f,n}$), 140.51 (C_1), 133.80 (q, J = 34.5 Hz, C_3), 129.65, 129.12, 129.10, 129.05, 129.01, 128.92, 128.86, 128.82, 128.80, 128.59 ($C_{2,4,i,d,l}$), 123.61 (q, J = 272.8 Hz, C_5), 115.53, 114.98, 114.72, 114.60, 114.59, 114.50, 114.47, 114.46, 114.34 ($C_{e,j,m}$), 73.58 (C_{hh}), 67.41 (C_w), 67.25 (C_b), 65.31 (C_a), 64.17, 64.10, 63.94, 63.93, 63.87 ($C_{g,o}$), 57.40 (C_u), 57.04 (C_r), 31.66, 31.42, 31.26, 31.20, 31.15, 31.05, 31.03, 30.92 ($C_{s,t,aa-dd}$), 30.63 – 29.20 ($C_{k,gg,ee,z}$, overlapped with solvent signal), 28.96 (C_x), 25.32 (C_y), 24.80 (C_{ff}), 15.94, 15.91, 15.79, 15.74, 15.72 ($C_{h,p}$).
^{19}F NMR (471 MHz, Acetone-d_6, 298 K) δ = -63.34 (s, 6F, F_5).
HRMS-ESI (+): 1680.5575 $[M]^+$, calculated for $C_{83}H_{108}Br_2F_6O_{17}S^+$: 1680.5573.

3.1.8 Synthesis of S13

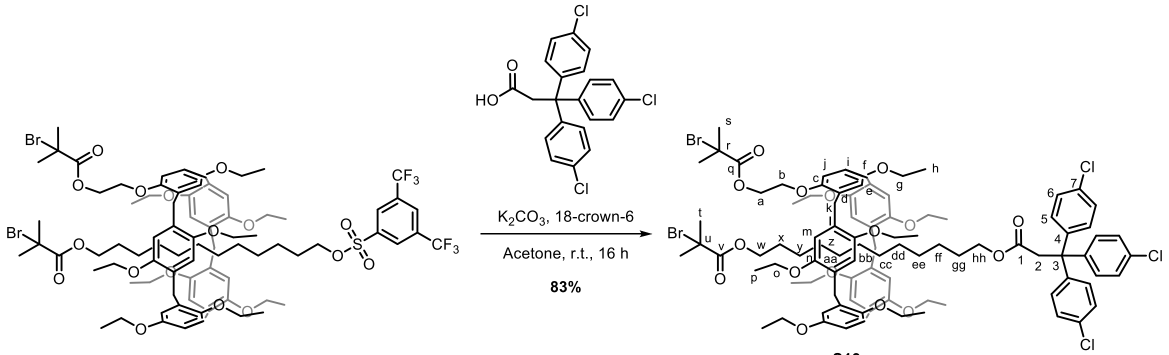

To a solution of 3,3,3-Tris(4-chlorophenyl)propionic acid (2 mg, 5 µmol, 1.0 eq.) in acetone (1 mL) was added K_2CO_3 (1 mg, 5 µmol, 1.0 eq.) and 18-crown-6 (1 mg, 5 µmol, 1.0 eq.). The mixture was stirred for 2 h at room temperature. **S11** (9 mg, 5 µmol, 1.0 eq.) was added and the mixture stirred for a further 16 h at room temperature. The solution was filtered, concentrated under vacuum, and the residue was purified by preparative TLC (500 µm, PE/DCM/EtOAc, 6/2/0.2, eluted twice) to yield **S13** as a white powder (8 mg, 4 µmol, 83% yield).

^{1}H NMR (500 MHz, Acetone-d_6, 298 K) δ = 7.41 – 7.35 (m, 6H, H_6), 7.35 – 7.28 (m, 6H, H_5), 7.01 – 6.87 (m, 10H, $H_{e,j,m}$), 4.83 (ddd, J = 12.1, 9.5, 3.0 Hz, 1H, H_a), 4.47 (dt, J = 11.9, 2.9 Hz, 1H, H_a), 4.36 (dt, J = 10.6, 3.0 Hz, 1H, H_b), 4.11 (td, J = 10.5, 3.0 Hz, 1H, H_b), 4.08 – 3.85 (m, 18H, $H_{g,o}$), 3.83 (s, 2H, H_2), 3.80 – 3.69 (m, 10H, H_k), 3.65 (t, J = 7.3 Hz, 2H, H_{hh}), 3.62 – 3.51 (m, 2H, H_w), 2.01 (s, 6H, H_t), 1.98 – 1.95 (s, s, 6H, H_s), 1.52 – 1.43 (m, 27H, $H_{h,p}$), 1.09 – 0.99 (m, 2H, H_{gg}), 0.73 – 0.62 (m, 4H, $H_{cc,dd}$), 0.57 – 0.47 (m, 2H, H_x), 0.47 – 0.36 (m, 6H, $H_{ff,ee,bb}$), -0.18 – -0.28 (m, 2H, H_{aa}), -1.04 – -1.13 (m, 2H, H_y), -1.13 – -1.22 (m, 2H, H_z).
^{13}C NMR (126 MHz, Acetone-d_6, 298 K) δ = 172.11, 172.03 ($C_{q,v}$), 170.74 (C_1), 150.94, 150.43, 150.37, 150.34, 149.87 ($C_{c,f,n}$), 145.91 (C_4), 132.87 (C_7), 131.74 (C_5), 129.49, 128.85, 128.78, 128.74, 128.64, 128.62, 128.55 ($C_{i,d,l,6}$), 115.21, 114.70, 114.54, 114.52, 114.39, 114.30, 114.26 ($C_{e,j,m}$), 67.91 (C_w), 67.04 (C_b), 65.48, 65.47 ($C_{a,hh}$), 63.96, 63.94, 63.82, 63.80 ($C_{g,o}$), 57.38 (C_u), 57.10 (C_r), 55.47 (C_3), 46.30 (C_2), 31.30, 31.27, 31.23, 31.21, 31.13, 31.07, 31.06 ($C_{s,t,aa-dd}$), 30.50 – 29.29 ($C_{k,ee}$, overlapped with solvent peak), 29.24 (C_{gg}), 28.70 (C_x), 28.48 (C_z), 25.97 (C_{ff}), 24.42 (C_y), 16.04, 16.01, 16.00, 15.90, 15.86 ($C_{h,p}$).
HRMS-ESI(+): 1790.5993 $[M]^+$, calculated for $C_{96}H_{119}Br_2Cl_3O_{16}^+$: 1790.5925.

3.1.9 Synthesis of S14

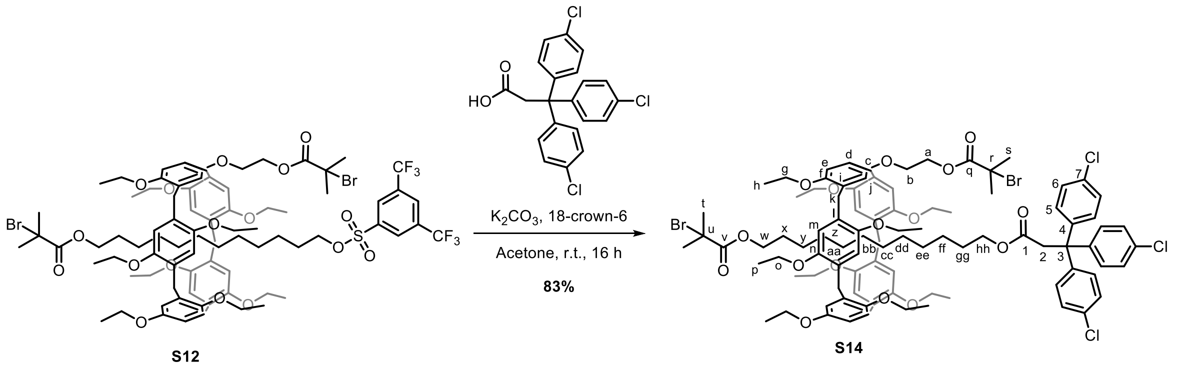

To a solution of 3,3,3-Tris(4-chlorophenyl)propionic acid (2 mg, 5 µmol, 1.0 eq.) in acetone (1 mL) was added K_2CO_3 (1 mg, 5 µmol, 1.0 eq.) and 18-crown-6 (1 mg, 5 µmol, 1.0 eq.). The mixture was stirred for 2 h at room temperature. **S12** (9 mg, 5 µmol, 1.0 eq.) was added and the mixture stirred for a further 16 h at room temperature. The solution was filtered, concentrated under vacuum, and the residue was purified by preparative TLC (500 µm, PE/EtOAc, 15/1, eluted twice) to yield **S14** as a white powder (8 mg, 4 µmol, 83% yield).

^{1}H NMR (500 MHz, Acetone-d_6, 298 K) δ = 7.41 – 7.35 (m, 6H, H_6), 7.34 – 7.28 (m, 6H, H_5), 6.99 – 6.88 (m, 10H, $H_{e,j,m}$), 4.76 (ddd, J = 12.0, 9.3, 3.0 Hz, 1H, H_a), 4.40 (dt, J = 11.8, 3.0 Hz, 1H, H_a), 4.25 (dt, J = 10.7, 3.1 Hz, 1H, H_b), 4.13 – 4.08 (m, 1H, H_b), 4.08 – 3.85 (m, 18H, $H_{g,o}$), 3.85 (s, 2H, H_2), 3.81 – 3.64 (m, 12H, $H_{k,w}$), 3.57 (t, J = 7.6 Hz, 2H, H_{hh}), 1.99 (s, 6H, H_t), 1.97 (s, 6H, H_s), 1.53 – 1.42 (m, 27H, $H_{h,p}$), 0.88 – 0.83 (m, 2H, H_{gg}), 0.81 – 0.72 (m, 2H, H_x), 0.62 – 0.54 (m, 2H, H_{cc}), 0.48 – 0.35 (m, 4H, $H_{bb,dd}$), 0.06 – -0.00 (m, 4H, $H_{ee,ff}$), -0.00 – -0.07 (m, 2H, H_{aa}), -0.56 – -0.64 (m, 2H, H_y), -0.70 – -0.79 (m, 2H, H_z).
^{13}C NMR (126 MHz, Acetone-d_6, 298 K) δ = δ 172.06, 172.03 ($C_{c,t}$), 170.72 (C_1), 151.60, 151.15, 151.10, 151.07, 151.03, 151.00 ($C_{i,p}$), 149.94 (C_f), 145.93 (C_4), 132.80 (C_7), 131.76 (C_5), 129.52, 128.85, 128.81, 128.69, 128.67, 128.66, 128.57, 128.55 ($C_{6,g,l,n}$), 115.40, 114.02, 113.78, 113.72, 113.62, 113.57, 113.52, 113.44 ($C_{h,m,o}$), 67.59 (C_u), 67.23 (C_e), 65.56 (C_{ff}), 65.45 (C_d), 57.49 (C_s), 57.11 (C_b), 55.92, 55.75, 55.71, 55.65, 55.58 ($C_{j,q}$), 55.42 (C_3), 46.11 (C_2), 31.62 (C_{aa}), 31.17, 31.14, 31.12, 31.06, 30.99 ($C_{a,r,bb,y,z}$), 30.46 – 29.21 ($C_{k,cc}$, overlapped with solvent peak), 28.98, 28.95, 28.90 ($C_{x,v,ee}$), 25.45 (C_{dd}), 24.53 (C_w).
^{13}C NMR (126 MHz, Acetone) δ 172.11 (C_v), 171.95 (C_q), 170.79 (C_1), 150.95, 150.46, 150.41, 150.35, 150.33, 149.81 ($C_{c,f,n}$), 145.93 (C_4), 132.89 (C_7), 131.74 (C_5), 129.44, 128.91, 128.88, 128.84, 128.78, 128.76, 128.71, 128.66, 128.64, 128.51 ($C_{i,d,l,6}$), 115.27, 114.79, 114.58, 114.46, 114.44, 114.41, 114.39, 114.30 ($C_{e,j,m}$), 67.62 (C_w), 66.94 (C_b), 65.69 (C_{hh}), 65.36 (C_a), 63.94, 63.91, 63.86, 63.78 ($C_{g,o}$), 57.37 (C_u), 57.05 (C_r), 55.38 (C_3), 46.24 (C_2), 31.30, 31.24, 31.22, 31.19, 31.12, 31.07, 31.05 ($C_{s,t,aa\text{-}dd}$), 30.45 – 29.29 ($C_{k,ee}$, overlapped with solvent peak), 29.20 (C_{gg}), 28.92 (C_z), 28.87 (C_x), 25.60 (C_{ff}), 24.92 (C_y), 16.00, 15.96, 15.94, 15.91 ($C_{h,p}$).
HRMS-ESI(+): 1813.5785 $[M+Na]^+$, calculated for $C_{96}H_{119}Br_2Cl_3O_{16}Na^+$: 1813.5823.

3.1.10 Determination of the *cis/trans* Isomers of S13 and S14

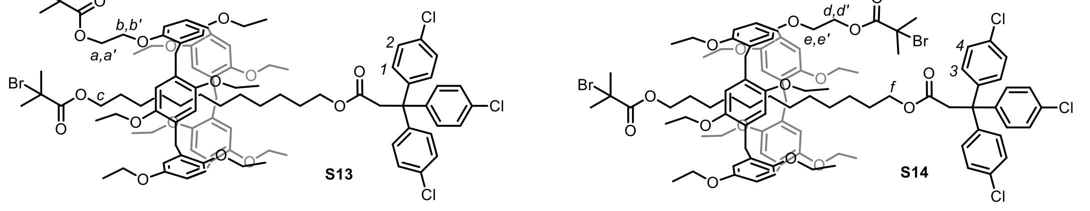

Figure S1. *cis*- and *trans*-structures of rotaxane isomers **S13** and **S14**.

We used 1D selective NOESY to determine the orientation of **S13** and **S14**. For **S13**, selective excitation

of proton H_b found on the macrocycle linker (**Figure S1**) indicates a correlation with proton H_c found on the rotaxane axle (**Figure S2**iv), hence demonstrating the *cis* isomerism. Likewise, for **S14**, selective radiation of protons H_d, $H_{d'}$, or H_e (all found on the macrocycle linker, **Figure S1**) demonstrates a correlation with protons H_f and $H_{4,5}$ found on the rotaxane axle (**Figure S3**), hence confirming the *trans* isomerism. The configuration of the precursor rotaxanes of these two isomers, **S11 and S12**, can be infered from these assignements (see section 0).

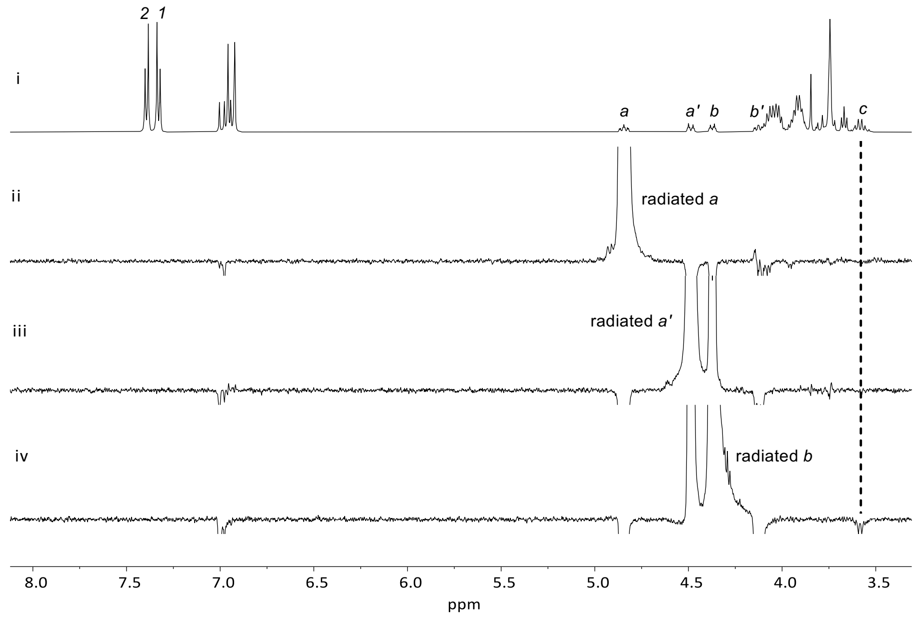

Figure S2. Partial ^{1}H NMR (500 MHz, Acetone-d_6, 298 K) spectrum of rotaxane **S13** (i) and partial 1D selective NOESY ^{1}H NMR (500 MHz, Acetone-d_6, 298 K) spectra of rotaxane **S13** with selective excitation of protons H_a (ii), $H_{a'}$ (iii) and H_b (iv). Assignments correspond to the lettering shown in **Figure S1**.

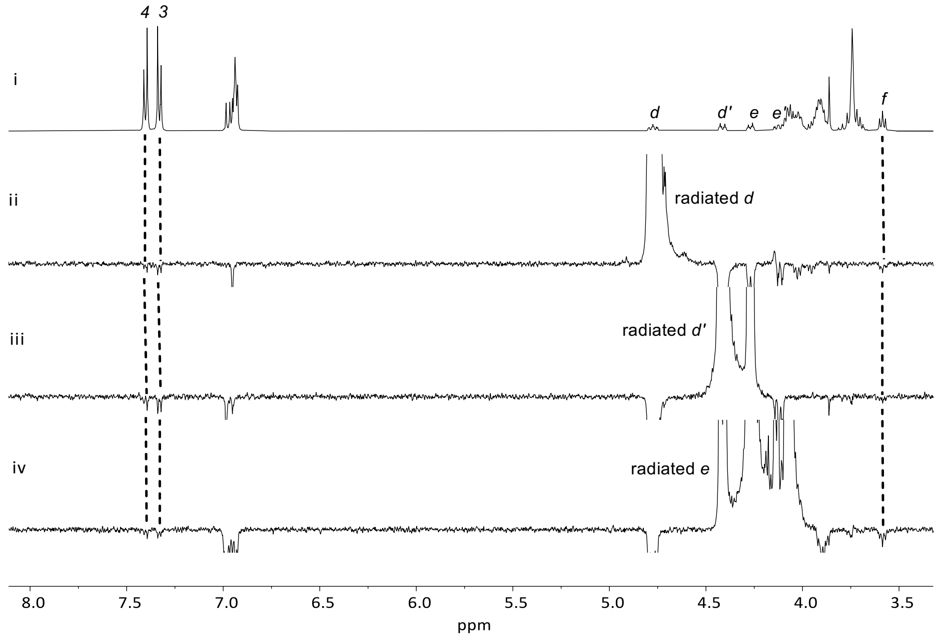

Figure S3. Partial ^{1}H NMR (500 MHz, Acetone-d_6, 298 K) spectrum of rotaxane **S14** (i) and partial 1D selective NOESY ^{1}H NMR (500 MHz, Acetone-d_6, 298 K) spectra of rotaxane **S14** with selective excitation of protons H_a (ii), $H_{a'}$ (iii) and H_b (iv). Assignments correspond to the lettering shown in **Figure S1**.

3.2 Synthesis of Control Compounds

3.2.1 Synthesis of S16

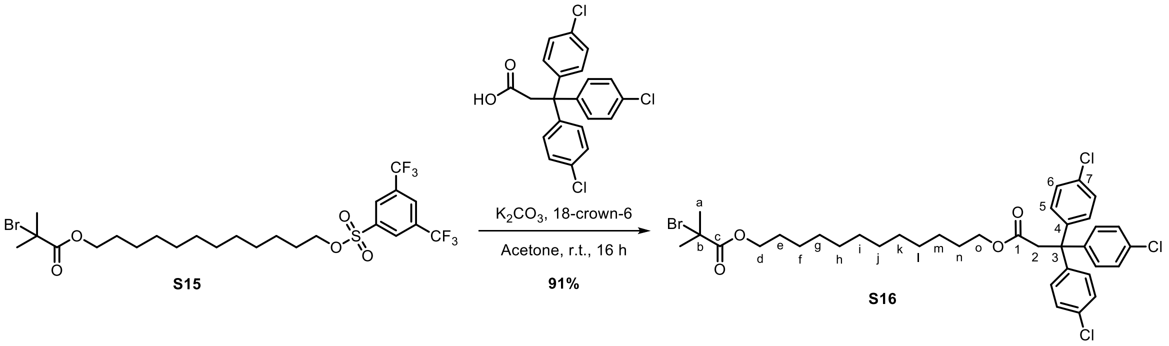

To a solution of 3,3,3-Tris(4-chlorophenyl)propionic acid (10 mg, 24 µmol, 1.0 eq.) in acetone (1 mL) was added K_2CO_3 (3 mg, 24 µmol, 1.0 eq.) and 18-crown-6 (6 mg, 34 µmol, 1.0 eq.). The mixture was stirred for 2 h at room temperature. **S15** (15 mg, 24 µmol, 1.0 eq.) was added and the mixture stirred for a further 16 h at room temperature. The solution was filtered, concentrated under vacuum, and the residue was purified by preparative TLC (500 µm, PE/EtOAc, 20/1, eluted twice) to yield **S16** as a white powder (16 mg, 22 µmol, 91% yield).

^{1}H NMR (400 MHz, Acetone-d_6, 298 K) δ = 7.36 – 7.31 (m, 6H, H_6), 7.31 – 7.26 (m, 6H, H_5), 4.16 (t, J = 6.5 Hz, 2H, H_d), 3.82 – 3.74 (m, 4H, $H_{2,o}$), 1.92 (s, 6H, H_a), 1.73 – 1.63 (m, 2H, H_e), 1.46 – 1.38 (m, 2H, H_f), 1.38 – 1.07 (m, 18H, $H_{g\text{-}n}$).
^{13}C NMR (101 MHz, Acetone-d_6, 298 K) δ = 171.79 (C_c), 170.78 (C_1), 145.79 (C_4), 132.73 (C_7), 131.69 (C_5), 131.66 (C_d), 128.77 (C_o), 66.47 (C_b), 64.93 (C_3), 55.61 (C_2), 46.24 (C_c), 30.93 (C_a), 30.46 – 29.09 ($C_{e,g\text{-}l,n}$, overlapped with solvent peak), 26.58 (C_m), 26.50 (C_f).
HRMS-ESI(+): 759.1373 $[M+Na]^+$, calculated for $C_{37}H_{44}BrCl_3O_4Na^+$: 759.1381.

4 Synthesis of Polymers

4.1 Representative Procedure for SET-LRP of Methyl Acrylate Using Mechanophore Initiators

Methyl acrylate was filtered through basic alumina to remove the inhibitor prior to use. A stock catalytic solution of Me_6TREN (16 µL, 0.060 mmol) and $CuBr_2$ (5.6 mg, 0.025 mmol) in dry DMSO (1 mL) was prepared. To a 5 mL microwave vial was added the appropriate initiator compound along with the catalytic solution, methyl acrylate, and dry DMSO. This solution was degassed by bubbling N_2 for 10 min. A Cu(0) wire wrapped around a stirrer bar, having been cleaned in 12 N HCl for 10 min, was added to the reaction mixture. The reaction mixture was degassed for a further 2 min before being stirred for 15 - 40 min (until the extent of polymerization, as determined approximately by the increasing viscosity of the solution, was deemed acceptable). The solution was added dropwise to a solution of vigorously stirred methanol; the precipitated polymer was recovered and dried under vacuum for two days to yield a white material. Molecular weight and dispersity indices were determined using an analytical SEC that had been calibrated with polystyrene standards.

4.2 Synthesis of Mechanophore Polymers

4.2.1 Synthesis of Polymer **1**$_{cis\text{-OMe-145}}$ and **1**$_{cis\text{-OMe-166}}$

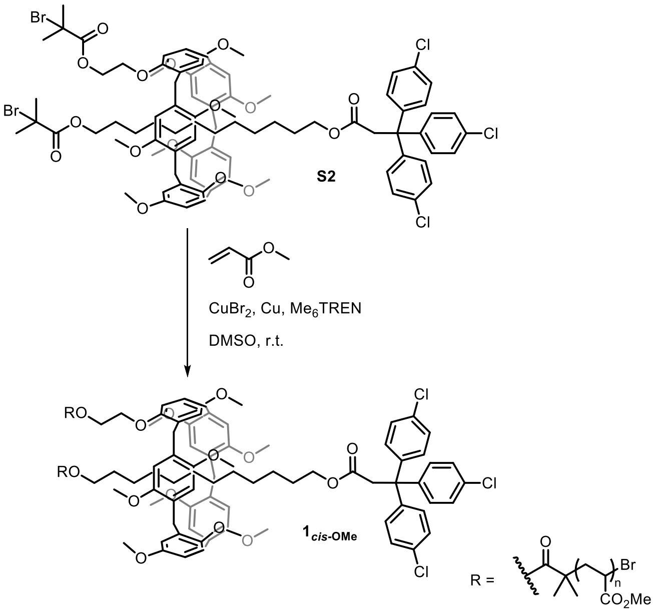

Synthesis followed the representative procedure. **S2** (4.0 mg, 2.4 µmol, 1.0 eq.), 19 µL of catalytic solution ($CuBr_2$: 0.5 µmol, 0.2 eq.; Me_6TREN: 1.2 µmol, 0.5 eq.), methyl acrylate (428 µL, 4.8 mmol, 2000.0 eq.), Cu (0) wire (~3 cm, ~30 mg, 0.5 mmol, ~197.0 eq.) and dry DMSO (428 µL) were used in the reaction to yield polymer **1**$_{cis\text{-OMe-145}}$ (132 mg, M_n = 145 kDa; $Đ$ = 1.16) and lc **1**$_{cis\text{-OMe-166}}$ (155 mg, M_n = 166 kDa; $Đ$ = 1.17) in two batches. Nomenclature: $_{145}$ and $_{166}$ are the M_n of the polymer in kDa.

4.2.2 Synthesis of Polymer $\mathbf{1}_{cis\text{-}OEt}$

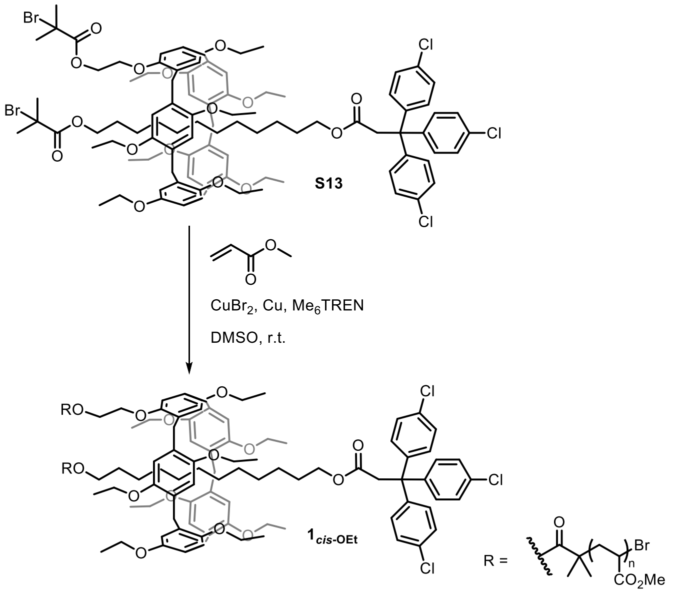

Synthesis followed the representative procedure. **S13** (1.0 mg, 0.6 µmol, 1.0 eq.), 4 µL of catalytic solution ($CuBr_2$: 0.5 µmol, 0.2 eq.; Me_6TREN: 1.2 µmol, 0.5 eq.), methyl acrylate (100 µL, 1.1 mmol, 2000.0 eq.), Cu (0) wire (~3 cm, ~30 mg, 0.5 mmol, ~845.0 eq.) and dry DMSO (100 µL) were used in the reaction to yield polymer $\mathbf{1}_{cis\text{-}OEt}$ (45 mg, M_n = 143 kDa; $Đ$ = 1.27).

4.2.3 Synthesis of Polymer $\mathbf{1}_{trans\text{-}OMe}$

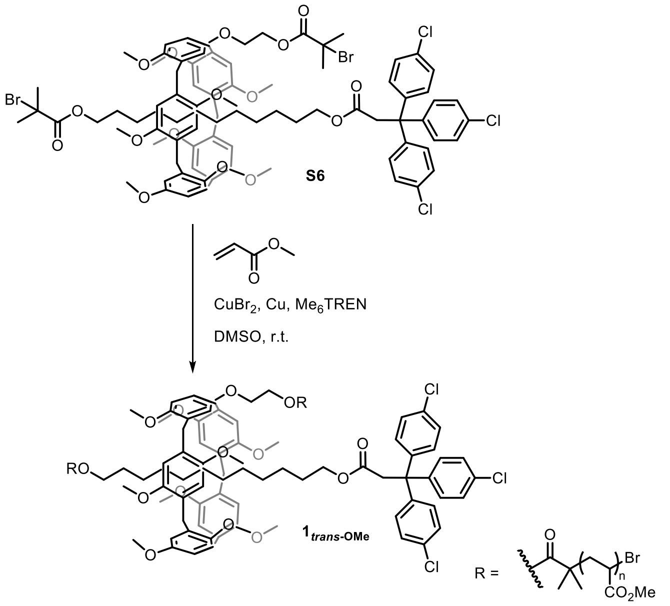

Synthesis followed the representative procedure. **S6** (4.0 mg, 2.4 µmol, 1.0 eq.), 19 µL of catalytic solution ($CuBr_2$: 0.5 µmol, 0.2 eq.; Me_6TREN: 1.2 µmol, 0.5 eq.), methyl acrylate (428 µL, 4.8 mmol, 2000.0 eq.), Cu (0) wire (~3 cm, ~30 mg, 0.5 mmol, ~197.0 eq.) and dry DMSO (428 µL) were used in the reaction to yield polymer $\mathbf{1}_{trans\text{-}OMe}$ (150 mg, M_n = 163 kDa; $Đ$ = 1.21).

4.2.4 Synthesis of Polymer 2_{cis}

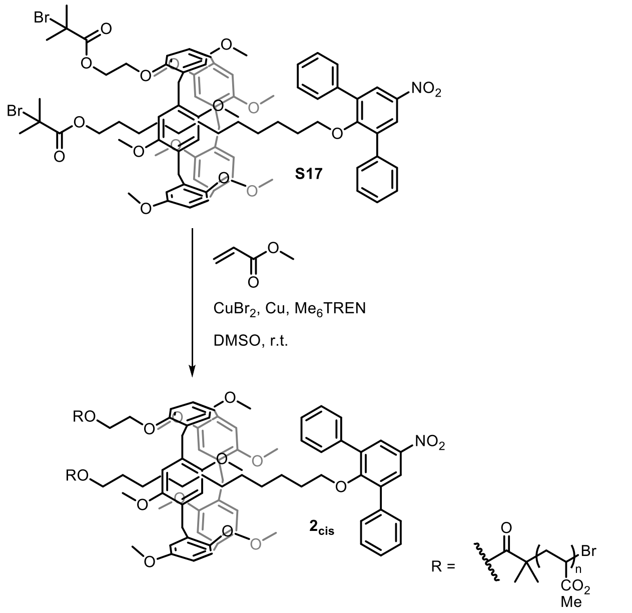

Synthesis followed the representative procedure. **S17** (4.0 mg, 2.6 µmol, 1.0 eq.), 21 µL of catalytic solution ($CuBr_2$: 0.5 µmol, 0.2 eq.; Me_6TREN: 1.2 µmol, 0.5 eq.), methyl acrylate (459 µL, 5.1 mmol, 2000.0 eq.), Cu (0) wire (~3 cm, ~30 mg, 0.5 mmol, ~183.0 eq.) and dry DMSO (459 µL) were used in the reaction to yield polymer $\mathbf{2}_{\mathbf{cis}}$ (135 mg, M_n = 138 kDa; $Đ$ = 1.14).

4.2.5 Synthesis of Polymer 3_{cis}

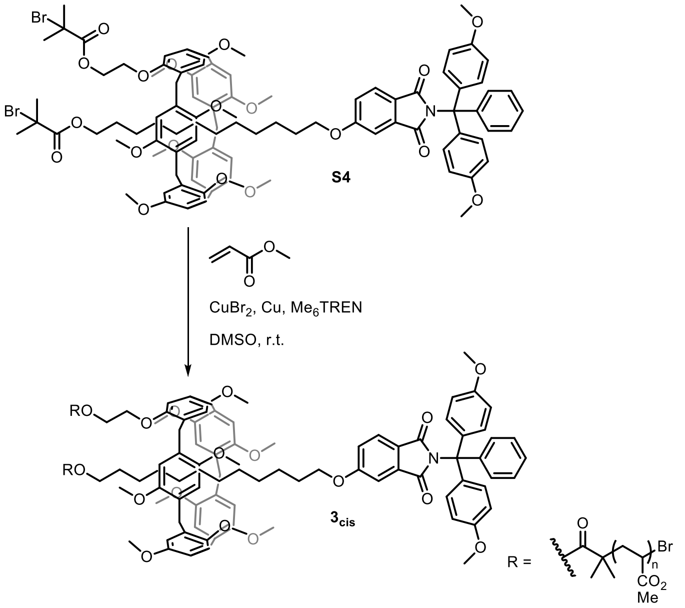

Synthesis followed the representative procedure. **S4** (3.0 mg, 1.7 µmol, 1.0 eq.), 14 µL of catalytic solution ($CuBr_2$: 0.3 µmol, 0.2 eq.; Me_6TREN: 0.8 µmol, 0.5 eq.), methyl acrylate (310 µL, 3.5 mmol, 2000.0 eq.), Cu (0) wire (~3 cm, ~30 mg, 0.5 mmol, ~272.0 eq.) and dry DMSO (310 µL) were used in the reaction to yield polymer $\mathbf{3}_{\mathbf{cis}}$ (82 mg, M_n = 117 kDa; $Đ$ = 1.19).

4.2.6 Synthesis of Polymer S19

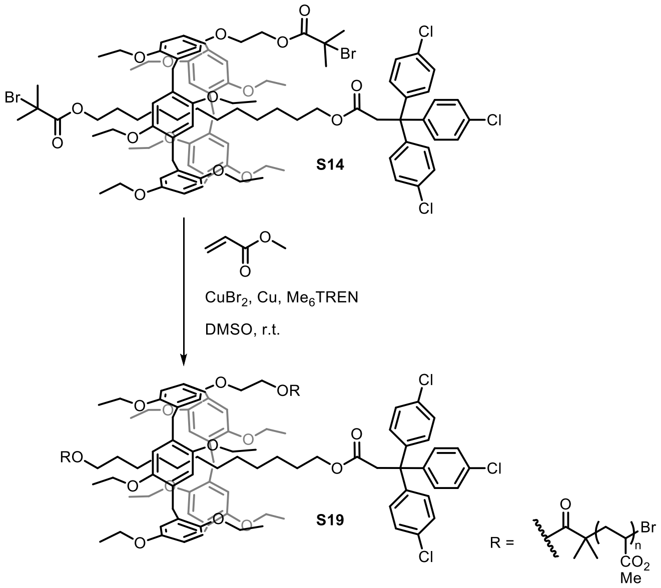

Synthesis followed the representative procedure. **S14** (1.0 mg, 0.6 µmol, 1.0 eq.), 4 µL of catalytic solution ($CuBr_2$: 0.5 µmol, 0.2 eq.; Me_6TREN: 1.2 µmol, 0.5 eq.), methyl acrylate (100 µL, 1.1 mmol, 2000.0 eq.), Cu (0) wire (~3 cm, ~30 mg, 0.5 mmol, ~845.0 eq.) and dry DMSO (100 µL) were used in the reaction to yield polymer **S19** (48 mg, M_n = 160 kDa; $Đ$ = 1.21).

4.3 Synthesis of Control Polymers

4.3.1 Synthesis of Polymer S20

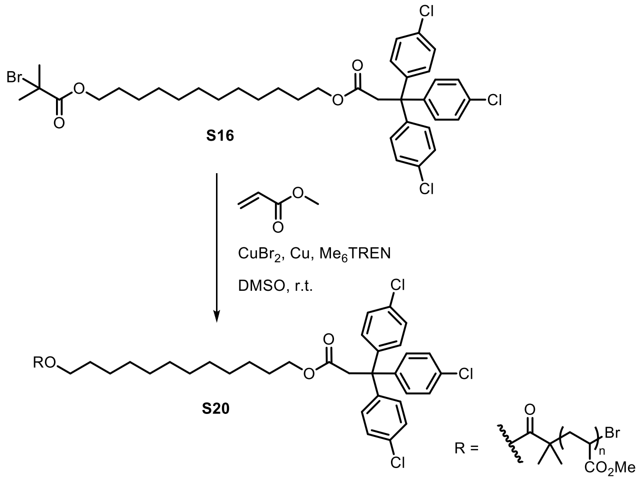

Synthesis followed the representative procedure. **S16** (4.0 mg, 5.4 µmol, 1.0 eq.), 43 µL of catalytic solution ($CuBr_2$: 1.1 µmol, 0.2 eq.; Me_6TREN: 2.6 µmol, 0.5 eq.), methyl acrylate (970 µL, 10.9 mmol, 2000.0 eq.), Cu (0) wire (~3 cm, ~30 mg, 0.5 mmol, ~87.0 eq.) and dry DMSO (970 µL) were used in the reaction to yield polymer **S20** (238 mg, M_n = 111 kDa; $Đ$ = 1.37).

4.3.2 Synthesis of Polymer S21

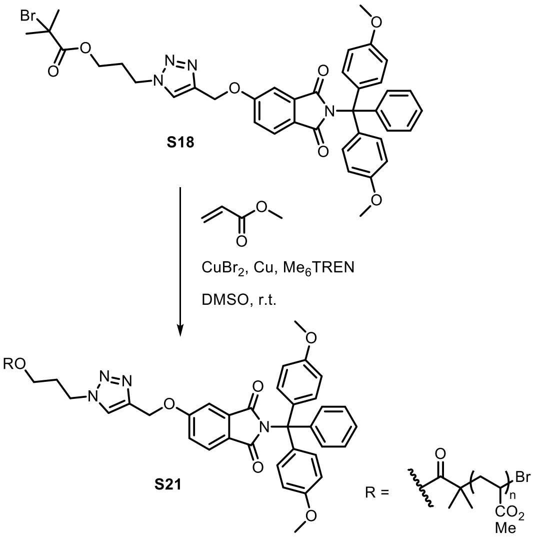

Synthesis followed the representative procedure. **S18** (3.0 mg, 4.0 µmol, 1.0 eq.), 32 µL of catalytic solution ($CuBr_2$: 0.8 µmol, 0.2 eq.; Me_6TREN: 1.9 µmol, 0.5 eq.), methyl acylate (712 µL, 8.0 mmol, 2000.0 eq.), Cu (0) wire (~3 cm, ~30 mg, 0.5 mmol, ~118.0 eq.) and dry DMSO (712 µL) were used in the reaction to yield polymer **S21** (120 mg, M_n = 93 kDa; $Đ$ = 1.16).

4.4 SEC Data for Synthesized Polymers

Table S1. M_n and *Đ* values for all synthesized polymers.

Polymer	M_n / kDa	*Đ*
**1**$_{cis\text{-OMe-145}}$	145	1.16
**1**$_{cis\text{-OMe-166}}$	166	1.17
**1**$_{cis\text{-OEt}}$	143	1.27
**1**$_{trans\text{-OMe}}$	163	1.21
**2**$_{cis}$	138	1.14
**3**$_{cis}$	117	1.19
**S19**	160	1.21
**S20**	111	1.37
**S21**	93	1.16

4.5 SEC Traces for Mechanophore and Control Polymers

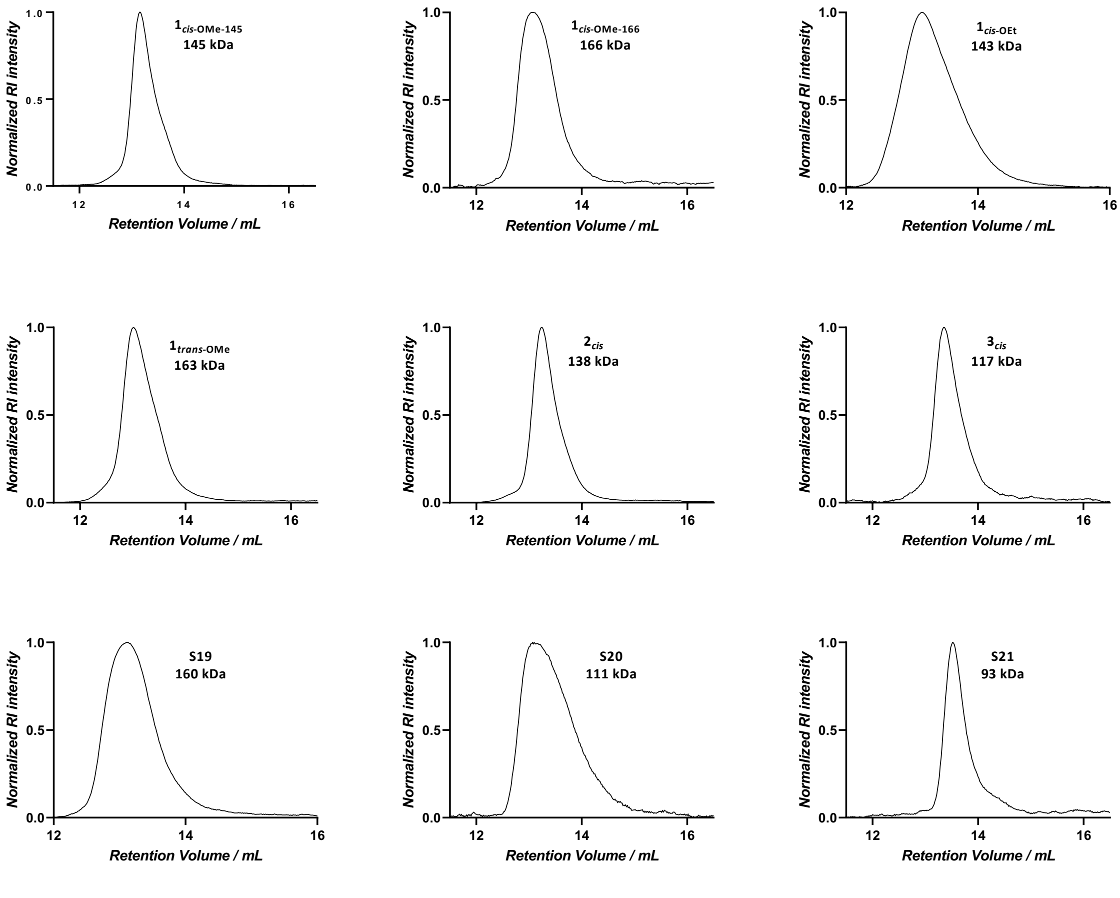

Figure S4. SEC traces for polymers **1**$_{cis\text{-OMe-145}}$, **1**$_{cis\text{-OMe-166}}$, **1**$_{cis\text{-OEt}}$, **3**$_{cis}$, **2**$_{cis}$, **1**$_{trans\text{-OMe}}$, **S19**, **S20** and **S21**.

5 Mechanophore Activation via Ultrasound

5.1 General Procedure for Sonication Experiments

The appropriate polymer (20 mg) was added to a Suslick cell and dissolved in the appropriate solvent 20 mL). The solution was degassed by bubbling N_2 through it for a minimum of 10 min prior to the start of sonication; bubbling of N_2 was also maintained throughout the experiment. The Suslick cell was cooled with a refrigerated circulating bath (Ecocool 100R) throughout the duration of the sonication to maintain a temperature of ~ 5-10 °C inside the cell. Pulsed ultrasound was applied to the system (1 s ON / 1 s OFF, 25% amplitude (13.0 W cm^{-2}), 20 kHz) for the desired period. After sonication, the solvent was evaporated, and the polymer was analysed by SEC and NMR spectroscopy. The post-sonication polymer was recovered and washed with MeOH to extract any nonpolymeric material. The remaining MeOH-washed polymer and the concentrated MeOH washings were then analysed by NMR spectroscopy.

5.2 Sonication of Mechanophore Polymer $\mathbf{1}_{\textit{cis}\text{-OMe-145}}$

The sonication of $\mathbf{1}_{\textit{cis}\text{-OMe-145}}$ was performed MeCN/H_2O (50/1) or dry THF following the general procedure described above. SEC analysis of the sonicated polymers showed complete cleavage (M_n of the post-sonication material was less than half of that of the pre-sonication polymer). Comparison of the ^{1}H NMR spectra of polymer $\mathbf{1}_{\textit{cis}\text{-OMe-145}}$ before and after sonication showed that both the flipping of hydroquinone ring and disassembly of the rotaxane by unstoppering occurred. The unstoppering process is evidenced by presence of 3,3,3-Tris(4-chlorophenyl)propionic acid **5** in both the crude reaction mixture and the MeOH extract after comparing with peaks (*e, f*) of a reference of **5**. In the ^{1}H NMR spectra of the polymer after sonication and MeOH wash, another set of peaks (*c, d*) near the peaks (*a, b*) of intact rotaxane polymer can be observed. This peaks better align with peaks the corresponding peaks of the *trans* rotaxane than those of *cis* rotaxane (**Figure S8**). It was confirmed to be a new rotaxane in which one hydroquinone ring of the pillar[5]arene macrocycle was flipped (see section 5.8). In the MeOH extract, we also found a small amount of **S22** (less than 2%), which probably originates from oxidation of the corresponding trityl radical (coming from the decomposition of **5** or a minor unstoppering pathway).[4,5]

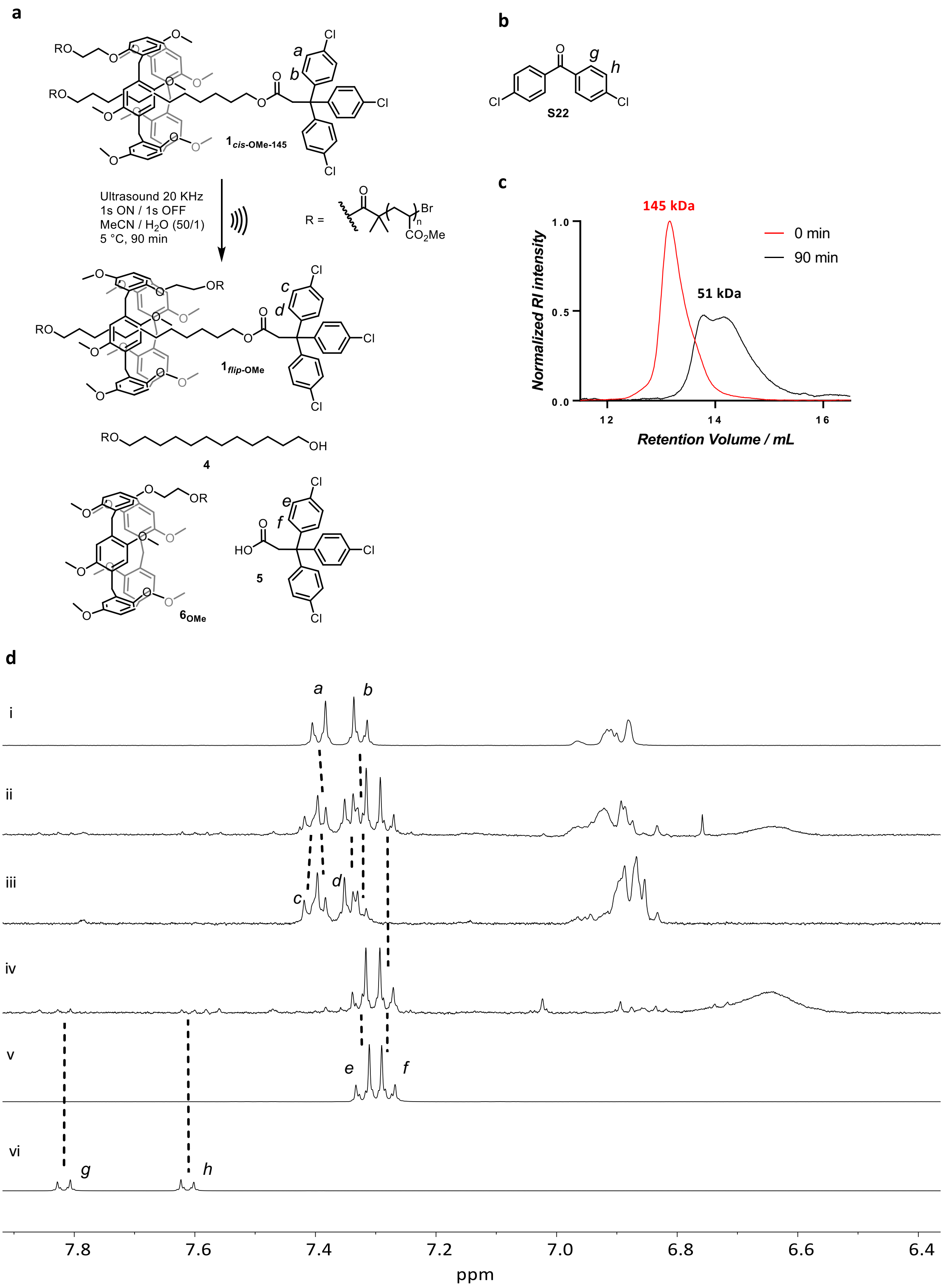

Figure S5. Sonication (run 1) of polymer $\mathbf{1}_{cis\text{-}\mathbf{OMe}\text{-}\mathbf{145}}$ in MeCN/H_2O (50/1). (a) Sonication of polymer $\mathbf{1}_{cis\text{-}\mathbf{OMe}\text{-}\mathbf{145}}$ affords flipped rotaxane polymer $\mathbf{1}_{flip\text{-}\mathbf{OMe}}$, polymer fragments **4**, $\mathbf{6}_{\mathbf{OMe}}$ and **5**. (b) Reference **S22**. (c) SEC traces of polymer $\mathbf{1}_{cis\text{-}\mathbf{OMe}\text{-}\mathbf{145}}$ before (red) and after (black) sonication. (d) Partial ^{1}H NMR (400 MHz, Acetone-d_6, 298 K) spectra comparison of polymer $\mathbf{1}_{cis\text{-}\mathbf{OMe}\text{-}\mathbf{145}}$ before (i), after sonication (ii), and after sonication and MeOH wash (iii), MeOH extract (iv), references of **5** (v) and **S22** (vi).

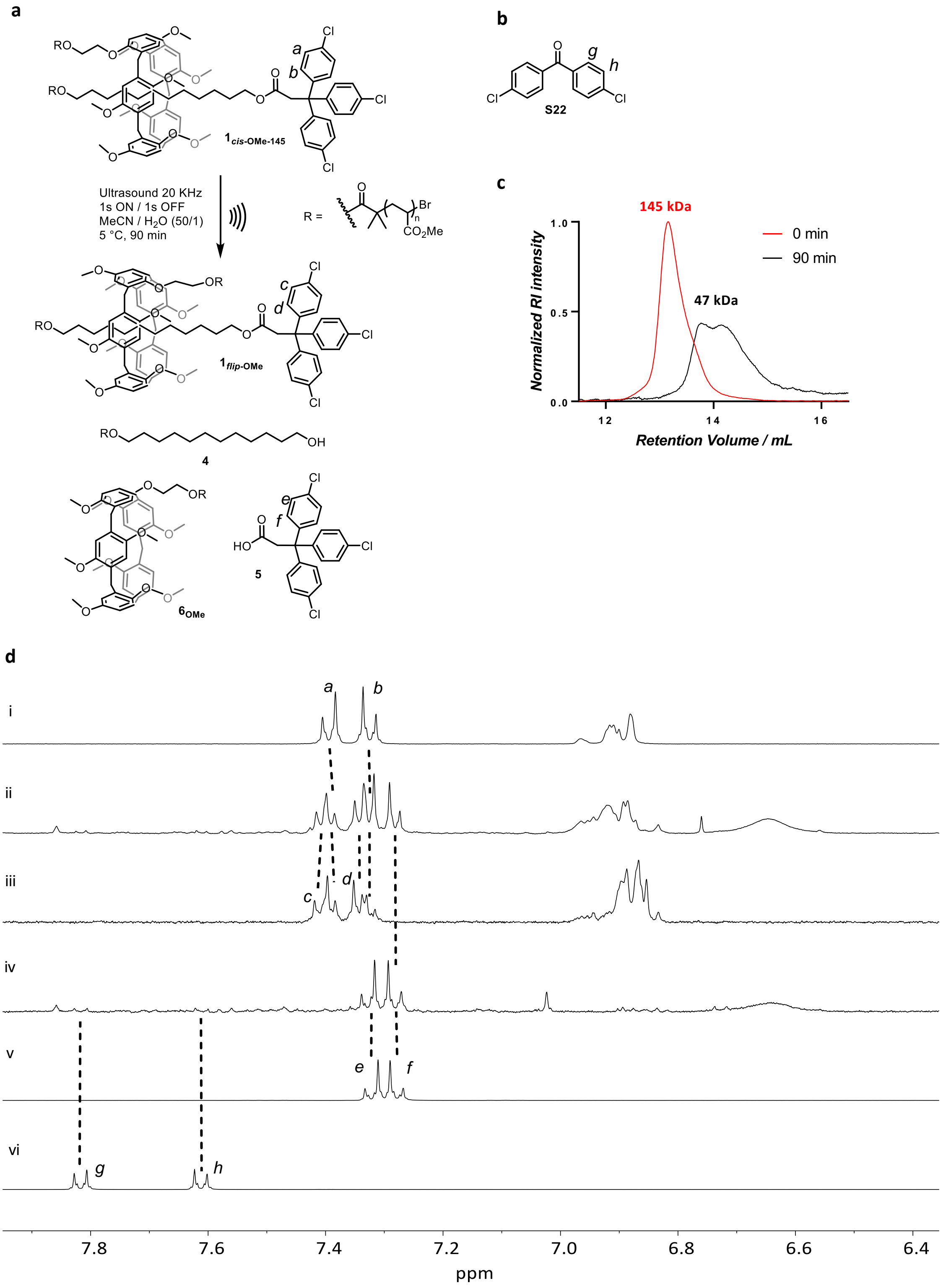

Figure S6. Sonication (run 2) of polymer $\mathbf{1}_{\mathit{cis}\text{-}\mathbf{OMe}\text{-}\mathbf{145}}$ in MeCN/H_2O (50/1). (a) Sonication of polymer $\mathbf{1}_{\mathit{cis}\text{-}\mathbf{OMe}\text{-}\mathbf{145}}$ affords flipped rotaxane polymer $\mathbf{1}_{\mathit{flip}\text{-}\mathbf{OMe}}$, polymer fragments **4**, $\mathbf{6}_{\mathbf{OMe}}$ and **5**. (b) Reference **S22**. (c) SEC traces of polymer $\mathbf{1}_{\mathit{cis}\text{-}\mathbf{OMe}\text{-}\mathbf{145}}$ before (red) and after (black) sonication. (d) Partial ^{1}H NMR (400 MHz, Acetone-d_6, 298 K) spectra comparison of polymer $\mathbf{1}_{\mathit{cis}\text{-}\mathbf{OMe}\text{-}\mathbf{145}}$ before (i), after sonication (ii), and after sonication and MeOH wash (iii), MeOH extract (iv), references of **5** (v) and **S22** (vi).

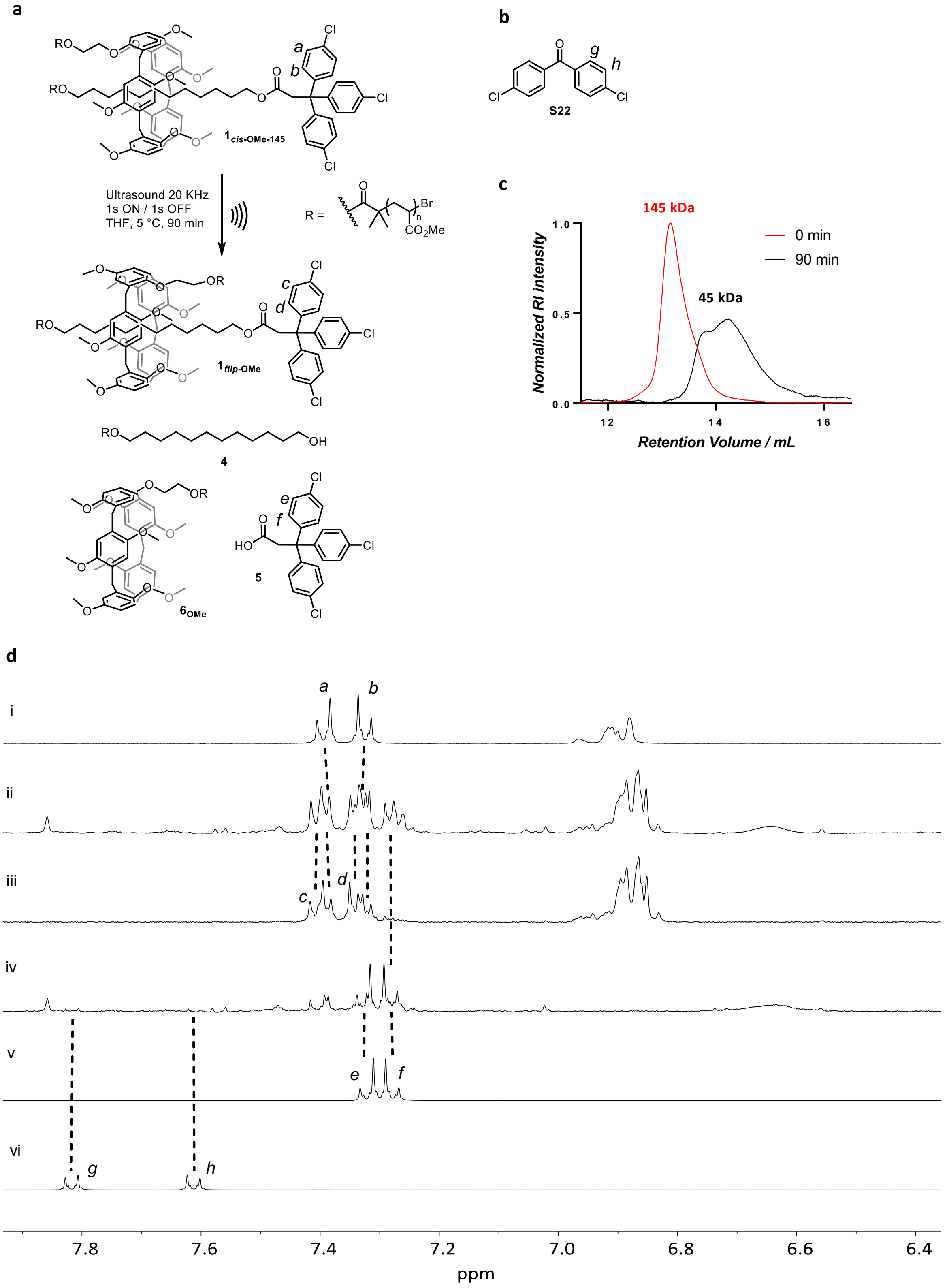

Figure S7. Sonication (run 3) of polymer $\mathbf{1}_{cis\text{-OMe-145}}$ in dry THF. (a) Sonication of polymer $\mathbf{1}_{cis\text{-OMe-145}}$ affords flipped rotaxane polymer $\mathbf{1}_{flip\text{-OMe}}$, polymer fragments **4**, $\mathbf{6}_{\mathbf{OMe}}$ and **5**. (b) Reference **S22**. (c) SEC traces of polymer $\mathbf{1}_{cis\text{-OMe-145}}$ before (red) and after (black) sonication. (d) Partial ^{1}H NMR (400 MHz, Acetone-d_6, 298 K) spectra comparison of polymer $\mathbf{1}_{cis\text{-OMe-145}}$ before (i), after sonication (ii), and after sonication and MeOH wash (iii), MeOH extract (iv), references of **5** (v) and **S22** (vi).

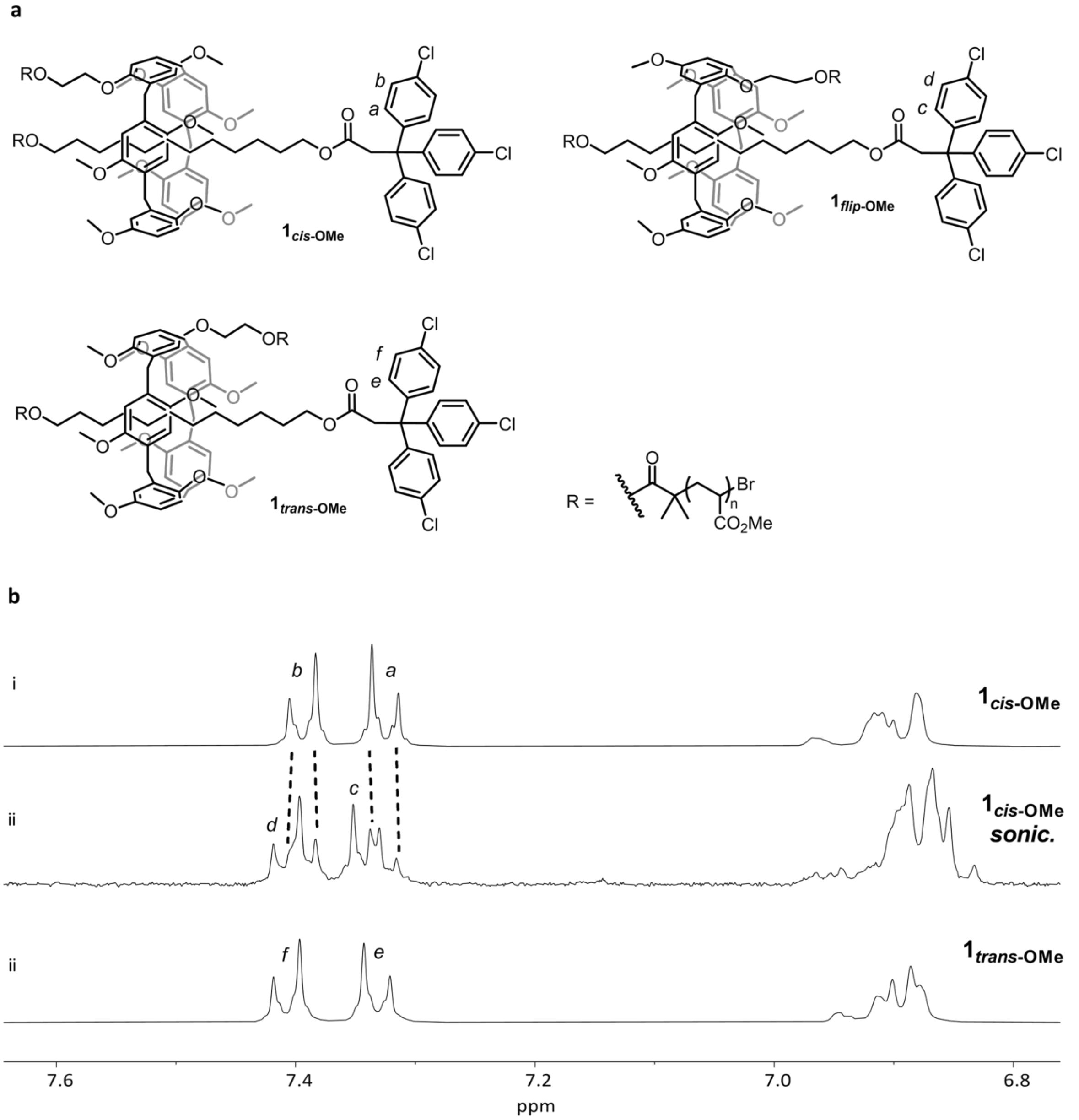

Figure S8. Comparison in the stopper's aromatic signals between **1**$_{cis\text{-OMe}}$ and **1**$_{trans\text{-OMe}}$. A similar shift would be expected from **1**$_{flip\text{-OMe}}$. The structure (a) and partial ^{1}H NMR (400 MHz, Acetone-d_6, 298 K) spectra comparison (b) of rotaxane mechanophores **1**$_{cis\text{-OMe}}$ and **1**$_{trans\text{-OMe}}$ along with post-sonication polymer **1**$_{cis\text{-OMe}}$ after MeOH wash.

5.3 Sonication of Mechanophore Polymer **1**$_{cis\text{-OEt}}$

The sonication of **1**$_{cis\text{-OEt}}$ was performed MeCN/H_2O (50/1) following the general procedure described above. SEC analysis of the sonicated polymers showed complete cleavage (M_n of the post-sonication material was less than half of that of the pre-sonication polymer). Comparison of the ^{1}H NMR spectra of polymer **1**$_{cis\text{-OEt}}$ before and after sonication showed that no flipping of hydroquinone ring occurred, though unstoppering is observed. The latter is evidenced by presence of 3,3,3-Tris(4-chlorophenyl)propionic acid **5** in both the crude reaction mixture and the MeOH extract after comparing with peaks (*c, d*) of a reference of **5**. Unlike **1**$_{cis\text{-OMe}}$, no change in the aromatic region

attributed to ring flipping (e.g. a/a' and b/b', **Figure S11**) is observed during the sonication of **1***cis***-OEt**, as the larger EtO substituent prevents the oxygen-through-the-annulus rotation of the hydroquinone ring. Similarly to **1***cis***-OMe**, a very small amount of **S22** (less than 6 %) is found in the MeOH extract.

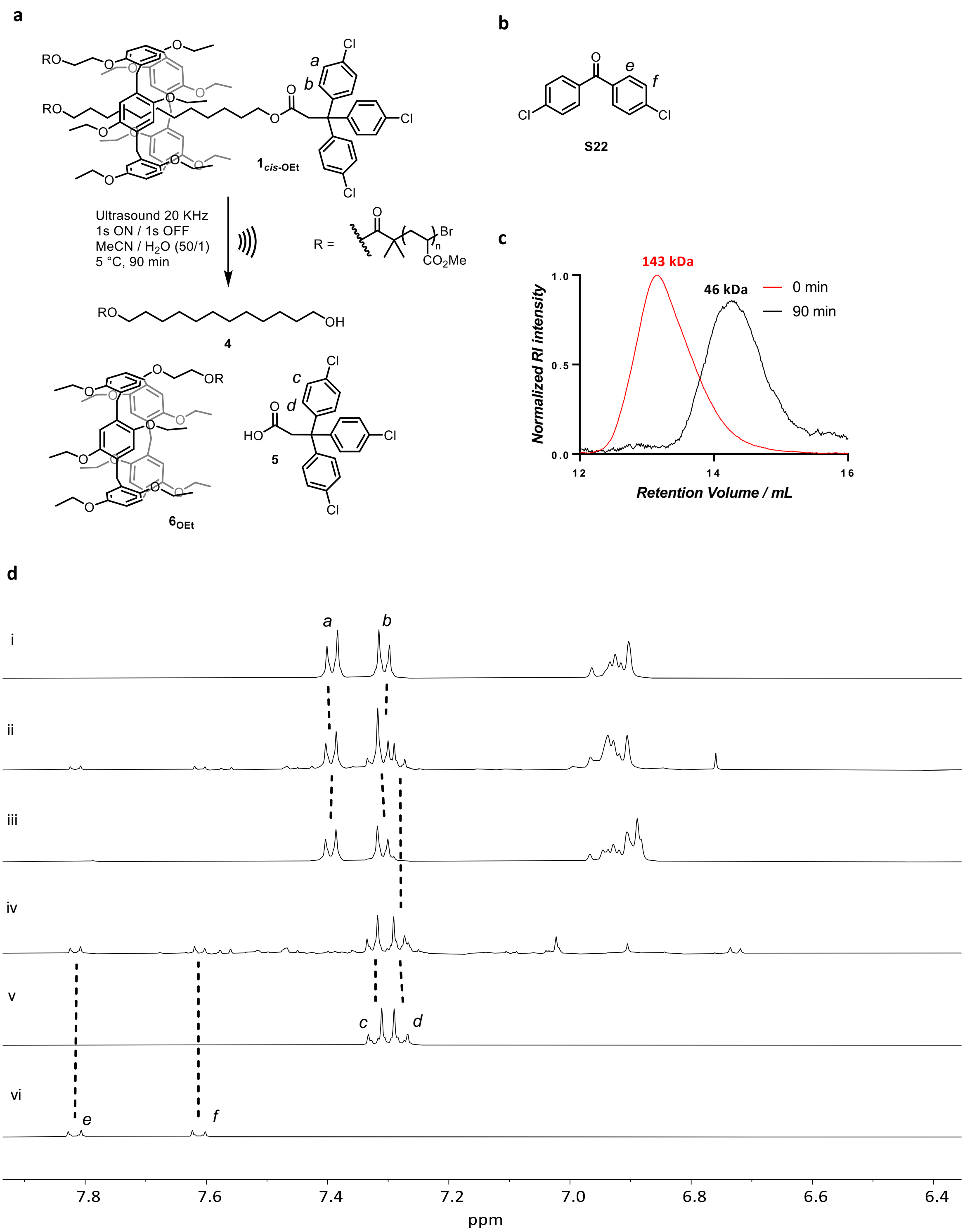

Figure S9. Sonication (run 1) of polymer **1***cis***-OEt** in MeCN/H_2O (50/1). (a) Sonication of polymer **1***cis***-OEt** affords polymer fragments **4**, **6OEt** and **5**. (b) Reference **S22**. (c) SEC traces of polymer **1***cis***-OEt** before (red) and after (black) sonication. (d) Partial ^{1}H NMR (400 MHz, Acetone-d_6, 298 K) spectra comparison of polymer **1***cis***-OEt** before (i), after sonication (ii), and after sonication and MeOH wash (iii), MeOH extract (iv), references of **5** (v) and **S22** (vi).

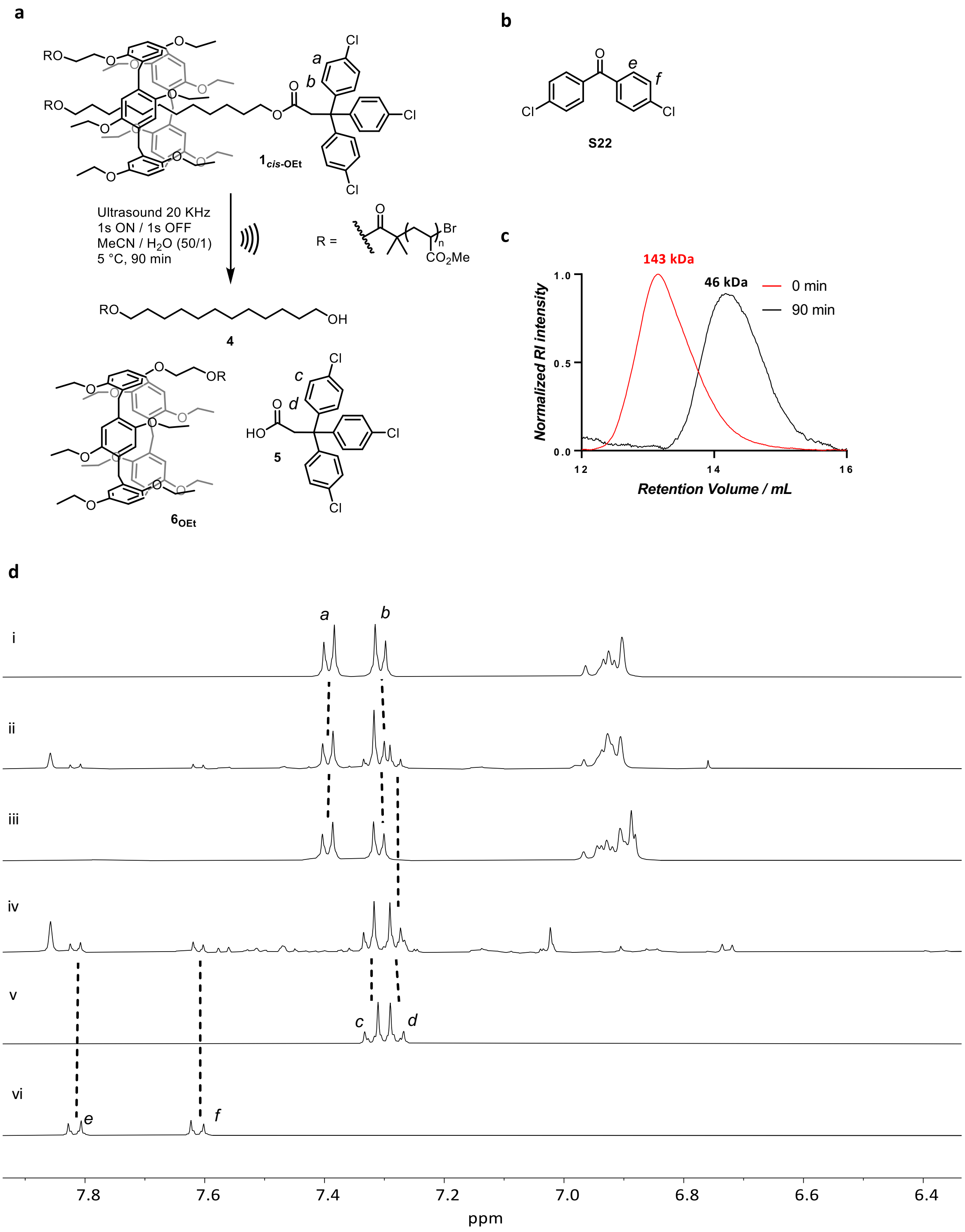

Figure S10. Sonication (run 2) of polymer $\mathbf{1}_{cis\text{-OEt}}$ in MeCN/H_2O (50/1). (a) Sonication of polymer $\mathbf{1}_{cis\text{-OEt}}$ affords polymer fragments **4**, $\mathbf{6}_{\text{OEt}}$ and **5**. (b) Reference **S22**. (c) SEC traces of polymer $\mathbf{1}_{cis\text{-OEt}}$ before (red) and after (black) sonication. (d) Partial ^{1}H NMR (400 MHz, Acetone-d_6, 298 K) spectra comparison of polymer $\mathbf{1}_{cis\text{-OEt}}$ before (i), after sonication (ii), and after sonication and MeOH wash (iii), MeOH extract (iv), references of **5** (v) and **S22** (vi).

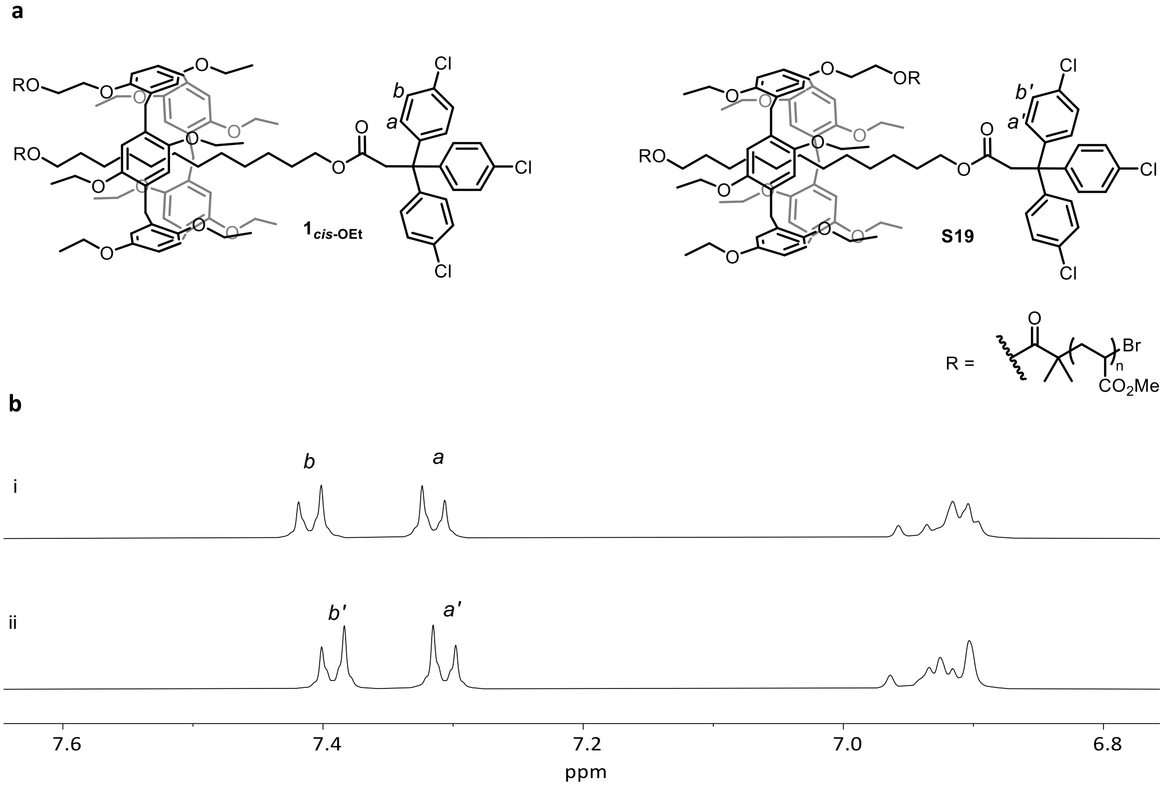

Figure S11. Comparison in the stopper's aromatic signals between $\mathbf{1}_{\textit{cis}\text{-OEt}}$ and $\mathbf{1}_{\textit{trans}\text{-OEt}}$. A similar shift would be expected from $\mathbf{1}_{\textit{flip}\text{-OEt}}$. The structure (a) and partial ^{1}H NMR (500 MHz, Acetone-d_6, 298 K) spectra comparison (b) of *cis*/*trans* rotaxane mechanophores $\mathbf{1}_{\textit{cis}\text{-OEt}}$ and **S19** with the same stopper.

5.4 Sonication of Mechanophore Polymer $\mathbf{3}_{cis}$

The sonication of $\mathbf{3}_{cis}$ was performed MeCN/H_2O (50/1) following the general procedure described above. SEC analysis of the sonicated polymers showed complete cleavage (M_n of the post-sonication material was less than half of that of the pre-sonication polymer). Comparison of the ^{1}H NMR spectra of polymer $\mathbf{3}_{cis}$ before and after sonication showed that no flipping of hydroquinone ring occurred, though unstoppering is observed. The latter is evidenced by presence of 4,4'-dimethoxytrityl alcohol **9** in the MeOH extract and the remaining phthalimide group in the post-sonication polymer after comparing with peaks (*c*-*g*) in reference **9** and polymer **8** respectively. Unlike $\mathbf{1}_{cis\text{-OMe}}$, no change in the aromatic region attributed to ring flipping (e.g. a/a' and b/b', **Figure S14**) is observed during the sonication of $\mathbf{3}_{cis}$, as the larger phthalimide axle prevents the oxygen-through-the-annulus rotation of the hydroquinone ring.

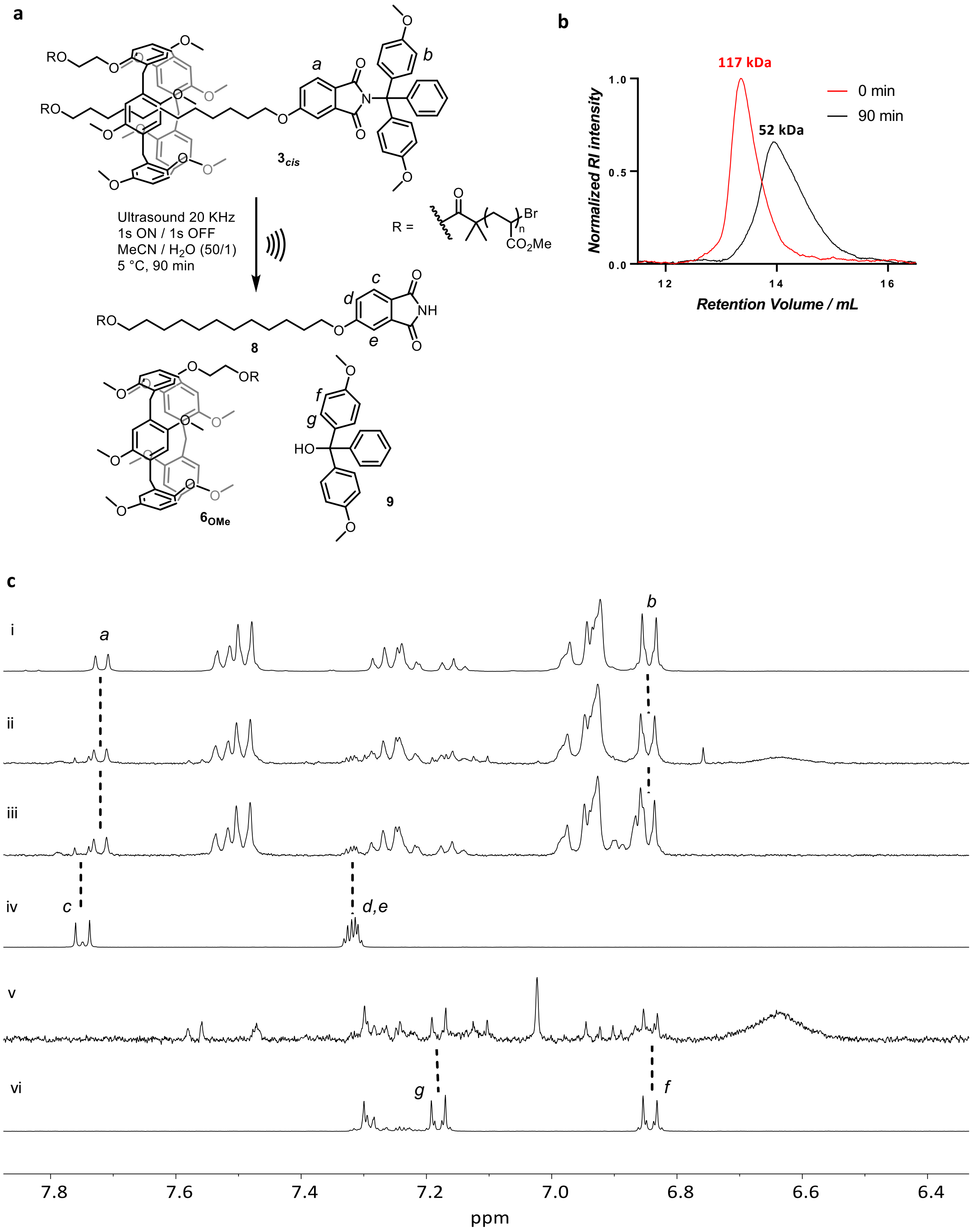

Figure S12. Sonication (run 1) of polymer **3***cis* in MeCN/H_2O (50/1). (a) Sonication of polymer **3***cis* affords polymer fragments **8**, **6OMe** and **9**. (b) SEC traces of polymer **3***cis* before (red) and after (black) sonication. (c) Partial ^{1}H NMR (400 MHz, Acetone-d_6, 298 K) spectra comparison of polymer **3***cis* before (i), after sonication (ii), and after sonication and MeOH wash (iii), reference polymer **8** (iv), MeOH extract (v) and reference **9** (vi).

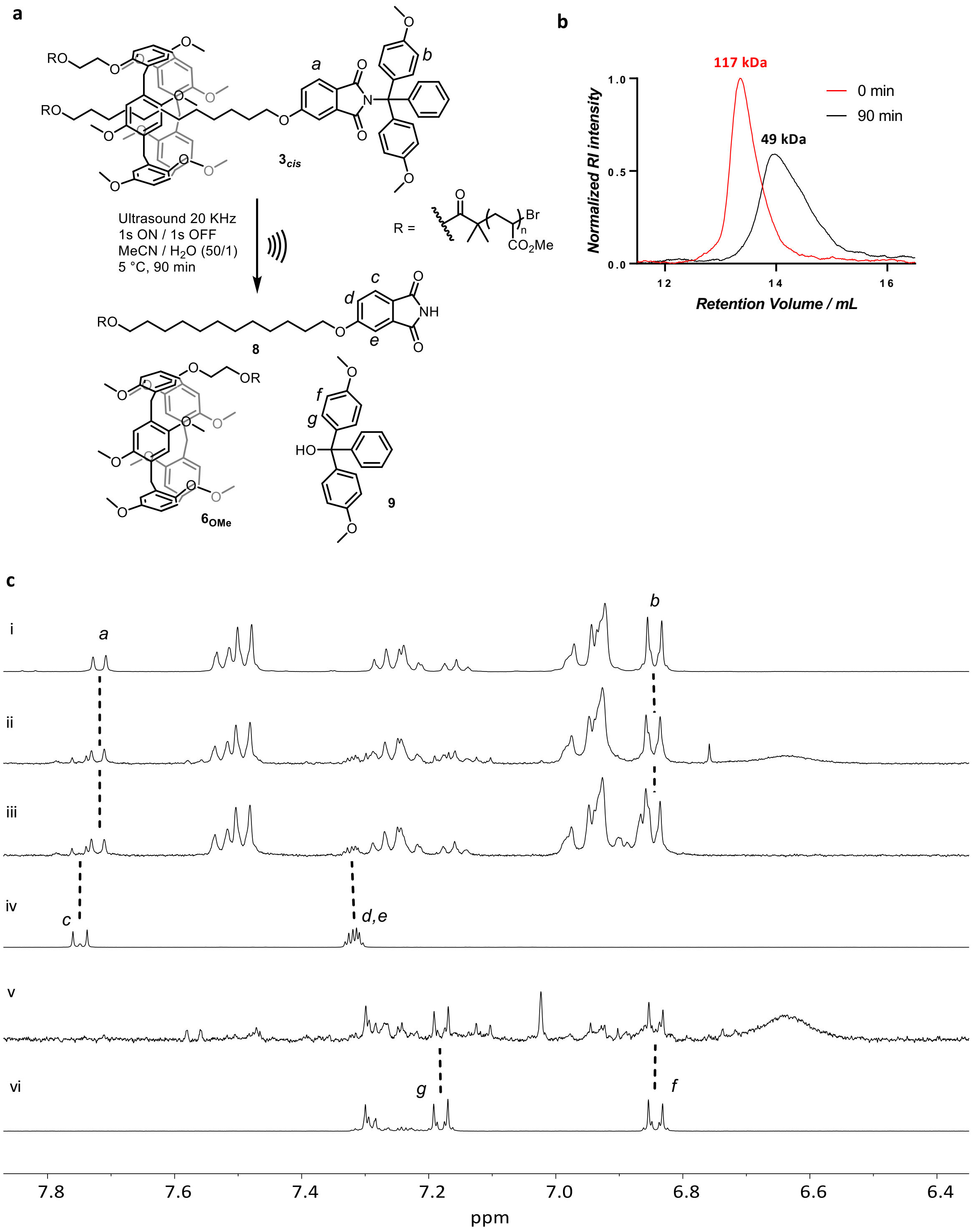

Figure S13. Sonication (run 2) of polymer $\mathbf{3}_{cis}$ in MeCN/H_2O (50/1). (a) Sonication of polymer $\mathbf{3}_{cis}$ affords polymer fragments **8**, $\mathbf{6}_{\mathbf{OMe}}$ and **9**. (b) SEC traces of polymer $\mathbf{3}_{cis}$ before (red) and after (black) sonication. (c) Partial ^{1}H NMR (400 MHz, Acetone-d_6, 298 K) spectra comparison of polymer $\mathbf{3}_{cis}$ before (i), after sonication (ii), and after sonication and MeOH wash (iii), reference polymer **8** (iv), MeOH extract (v) and reference **9** (vi).

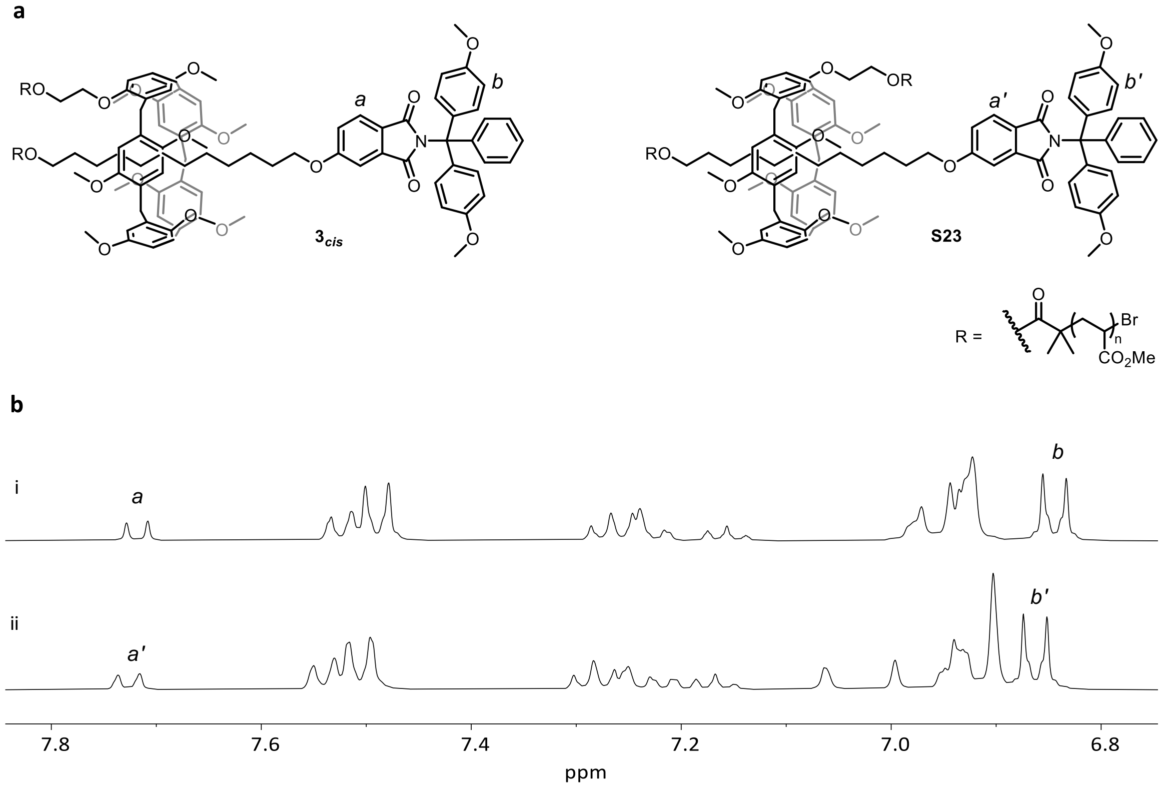

Figure S14. Comparison in the stopper's aromatic signals between $\mathbf{3}_{cis}$ and **S23**. A similar shift would be expected from $\mathbf{3}_{flip}$. The structure (a) and partial ^{1}H NMR (400 MHz, Acetone-d_6, 298 K) spectra comparison (b) of *cis*/*trans* rotaxane mechanophores $\mathbf{3}_{cis}$ and **S19** with the same stopper.

5.5 Sonication of Mechanophore Polymer $\mathbf{1}_{trans\text{-}OMe}$

The sonication of $\mathbf{1}_{trans\text{-}OMe}$ was performed MeCN/H_2O (50/1) or dry THF following the general procedure described above. SEC analysis of the sonicated polymers showed complete cleavage (M_n of the post-sonication material was less than half of that of the pre-sonication polymer). Comparison of the ^{1}H NMR spectra of polymer $\mathbf{1}_{trans\text{-}OMe}$ before and after sonication showed that unstoppering occurred. It is evidenced by presence of 3,3,3-Tris(4-chlorophenyl)propionic acid **5** in both the crude reaction mixture and the MeOH extract after comparing with peaks (*c, d*) of a reference of **5**. Similarly to $\mathbf{1}_{cis\text{-}OMe}$, a very small amount of **S22** (less than 4 %) is found in the MeOH extract.

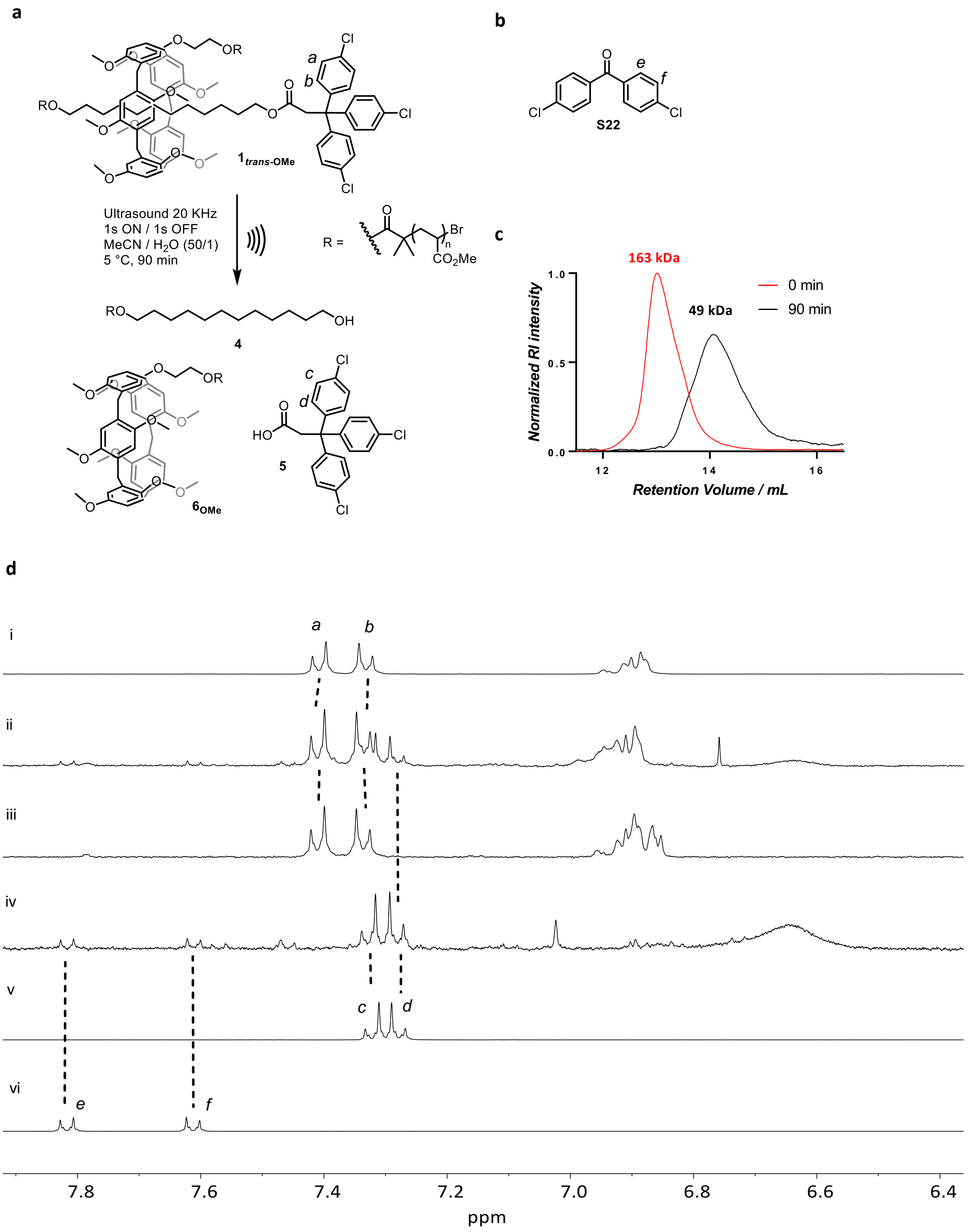

Figure S15. Sonication (run 1) of polymer $\mathbf{1}_{\textit{trans}\text{-OMe}}$ in MeCN/H_2O (50/1). (a) Sonication of polymer $\mathbf{1}_{\textit{trans}\text{-OMe}}$ affords polymer fragments **4**, $\mathbf{6}_{OMe}$ and **5**. (b) Reference **S22**. (c) SEC traces of polymer $\mathbf{1}_{\textit{trans}\text{-OMe}}$ before (red) and after (black) sonication. (d) Partial ^1H NMR (400 MHz, Acetone-d_6, 298 K) spectra comparison of polymer $\mathbf{1}_{\textit{trans}\text{-OMe}}$ before (i), after sonication (ii), and after sonication and MeOH wash (iii), MeOH extract (iv), references of **5** (v) and **S22** (vi).

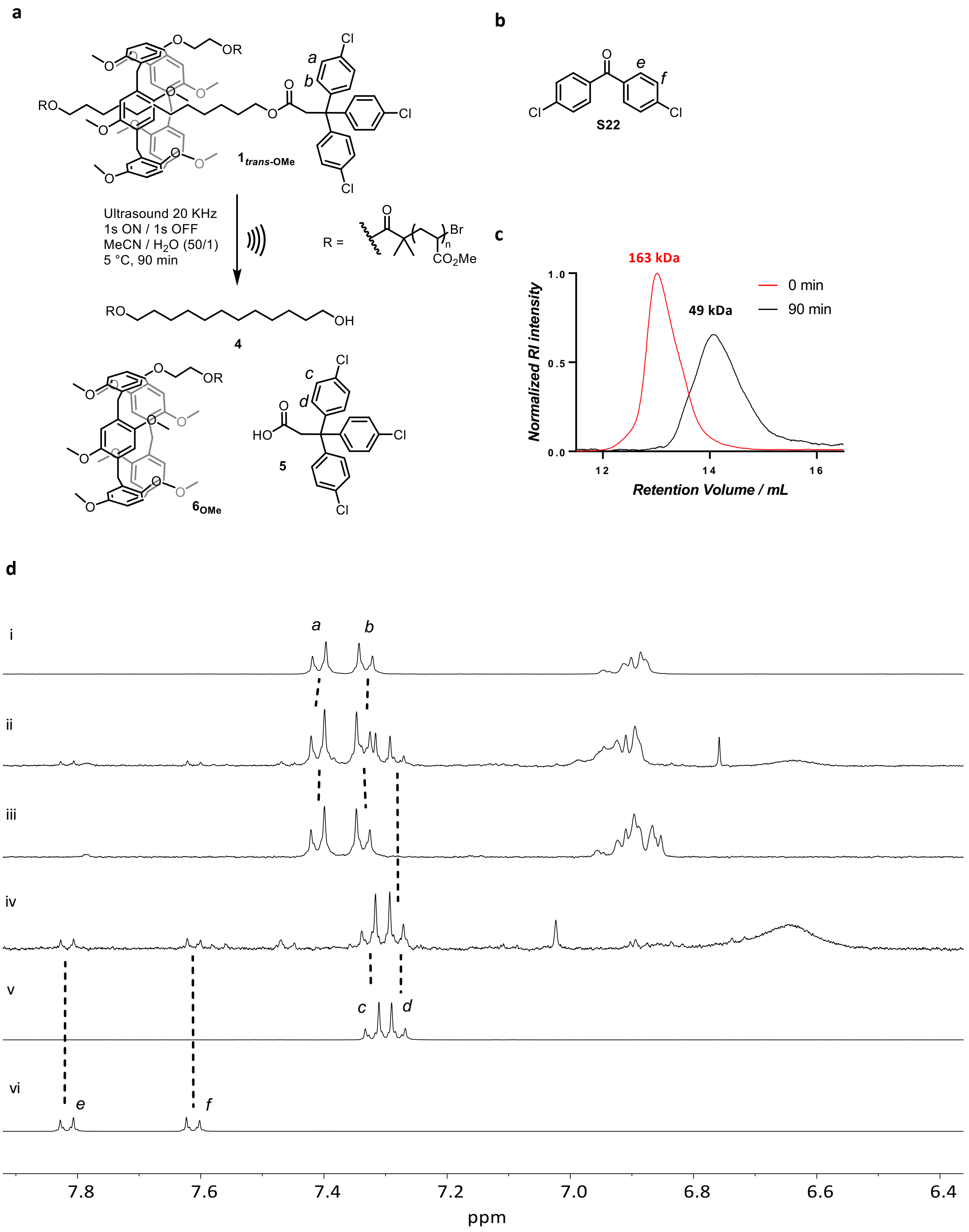

Figure S16. Sonication (run 2) of polymer $\mathbf{1}_{\textit{trans}\text{-OMe}}$ in MeCN/H_2O (50/1). (a) Sonication of polymer $\mathbf{1}_{\textit{trans}\text{-OMe}}$ affords polymer fragments **4**, $\mathbf{6}_{OMe}$ and **5**. (b) Reference **S22**. (c) SEC traces of polymer $\mathbf{1}_{\textit{trans}\text{-OMe}}$ before (red) and after (black) sonication. (d) Partial ^{1}H NMR (400 MHz, Acetone-d_6, 298 K) spectra comparison of polymer $\mathbf{1}_{\textit{trans}\text{-OMe}}$ before (i), after sonication (ii), and after sonication and MeOH wash (iii), MeOH extract (iv), references of **5** (v) and **S22** (vi).

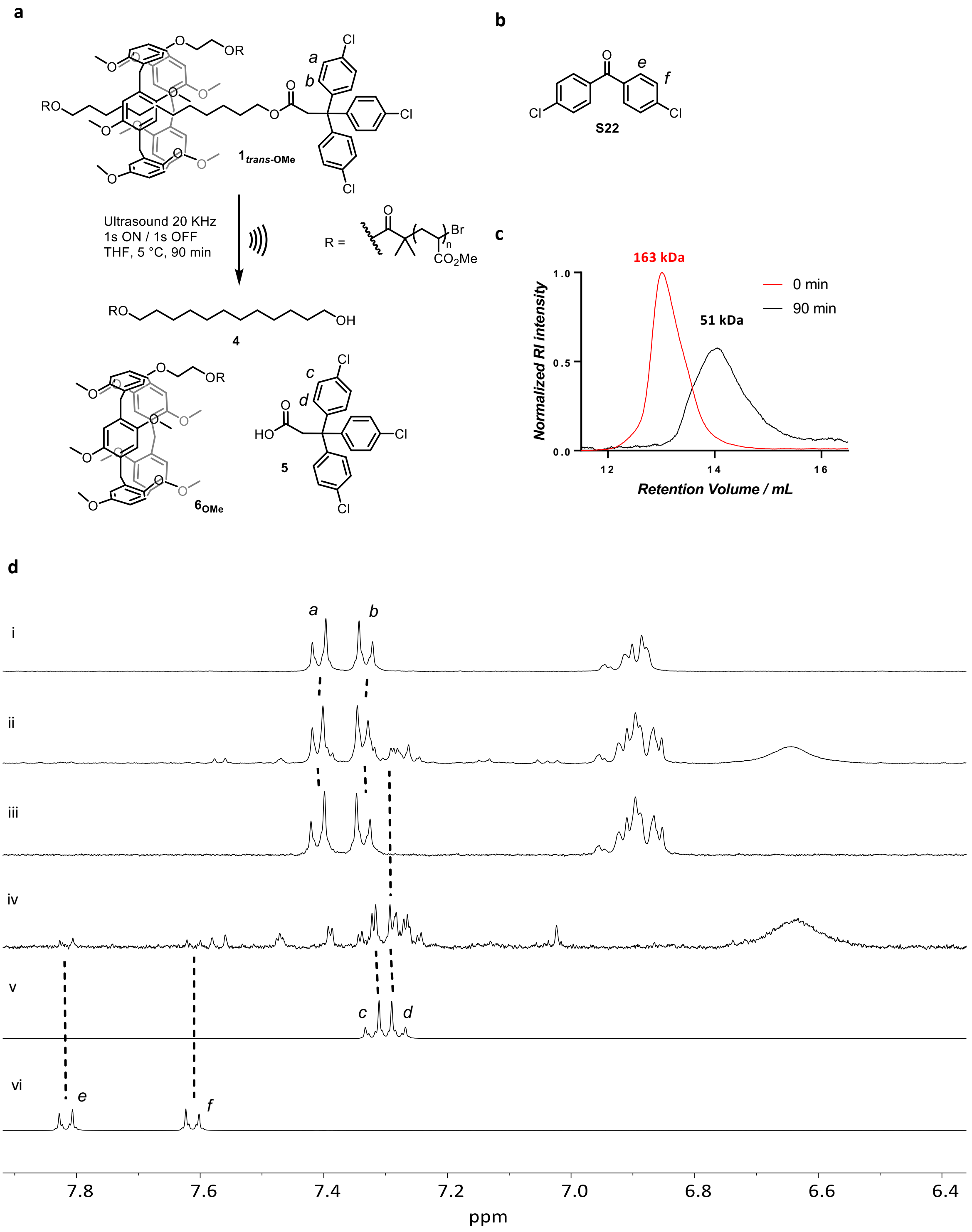

Figure S17. Sonication (run 3) of polymer $\mathbf{1}_{trans\text{-OMe}}$ in dry THF. (a) Sonication of polymer $\mathbf{1}_{trans\text{-OMe}}$ affords polymer fragments **4**, $\mathbf{6}_{\text{OMe}}$ and **5**. (b) Reference **S22**. (c) SEC traces of polymer $\mathbf{1}_{trans\text{-OMe}}$ before (red) and after (black) sonication. (d) Partial ^{1}H NMR (400 MHz, Acetone-d_6, 298 K) spectra comparison of polymer $\mathbf{1}_{trans\text{-OMe}}$ before (i), after sonication (ii), and after sonication and MeOH wash (iii), MeOH extract (iv), references of **5** (v) and **S22** (vi).

5.6 Sonication of Mechanophore Polymer $\mathbf{2}_{cis}$

The sonication of $\mathbf{2}_{cis}$ was performed MeCN/H_2O (50/1) following the general procedure described above. SEC analysis of the sonicated polymers showed complete cleavage (M_n of the post-sonication material was less than half of that of the pre-sonication polymer). Comparison of the ^1H NMR spectra of polymer $\mathbf{2}_{cis}$ before and after sonication showed that both the flipping of hydroquinone ring and disassembly of the rotaxane by unstoppering occurred. The unstoppering process is evidenced by presence of 4-nitro-2,6-diphenylphenol **7** in both the crude reaction mixture and the MeOH extract after comparing with peaks (*i*-*l*) of a reference of **7**. In the ^1H NMR spectra of the polymer after sonication and MeOH wash, another set of peaks (*e*-*h*) similar to the peaks (*a*-*d*) of intact rotaxane polymer can be observed. It was confirmed to be a new rotaxane in which one hydroquinone ring of the pillar[5]arene macrocycle was flipped.

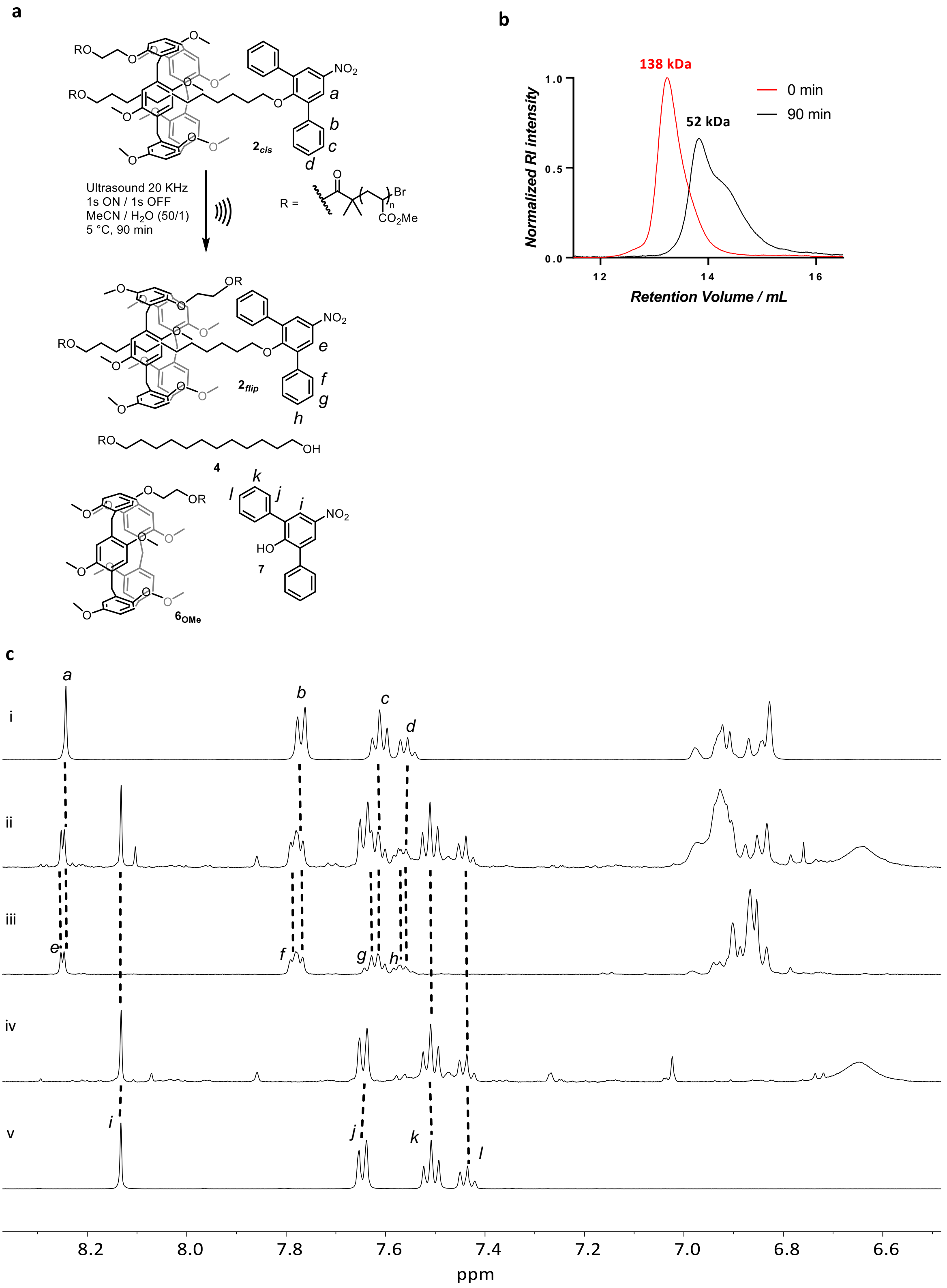

Figure S18. Sonication (run 1) of polymer **2**$_{cis}$ in MeCN/H_2O (50/1). (a) Sonication of polymer **2**$_{cis}$ affords flipped rotaxane polymer **2**$_{flip}$, polymer fragments **4**, **6**$_{OMe}$ and **7**. (b) SEC traces of polymer **2**$_{cis}$ before (red) and after (black) sonication. (c) Partial ^1H NMR (500 MHz, Acetone-d_6, 298 K) spectra comparison of polymer **2**$_{cis}$ before (i), after sonication (ii), and after sonication and MeOH wash (iii), MeOH extract (iv) and reference **7** (v).

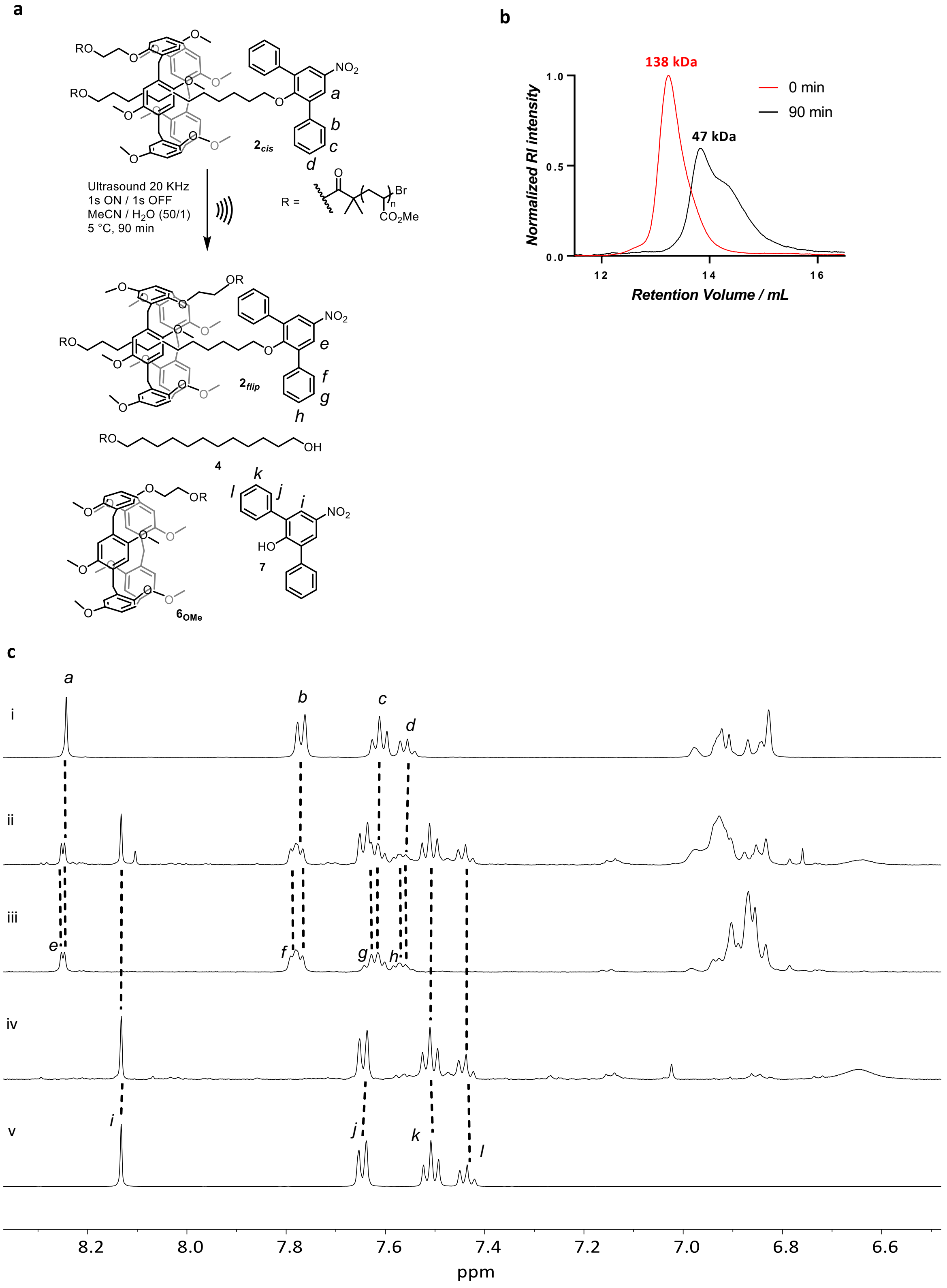

Figure S19. Sonication (run 2) of polymer $\mathbf{2}_{cis}$ in MeCN/H_2O (50/1). (a) Sonication of polymer $\mathbf{2}_{cis}$ affords flipped rotaxane polymer $\mathbf{2}_{flip}$, polymer fragments **4**, $\mathbf{6}_{\mathbf{OMe}}$ and **7**. (b) SEC traces of polymer $\mathbf{2}_{cis}$ before (red) and after (black) sonication. (c) Partial ^{1}H NMR (500 MHz, Acetone-d_6, 298 K) spectra comparison of polymer $\mathbf{2}_{cis}$ before (i), after sonication (ii), and after sonication and MeOH wash (iii), MeOH extract (iv) and reference **7** (v).

5.7 Kinetic Investigation

5.7.1 Comparison of Rate of Flipping and Unstoppering in Polymers $\mathbf{1}_{cis\text{-OMe-145}}$

The sonication of $\mathbf{1}_{cis\text{-OMe-145}}$ was performed MeCN/H_2O (50/1) following the general procedure described above at varied sonication times (2, 5, 10, 20, and 40min). The conversion of flipped product $\mathbf{1}_{flip\text{-OMe}}$, and unstoppering product **5** was determined by ^{1}H NMR (**Figure S20d**) following the procedure described in section 6.1. The plotting of this values show that the rate of flipping is faster than that of unstoppering (**Figure S20**c), as the conversion of $\mathbf{1}_{flip\text{-OMe}}$ plateaus after 10 min while unstoppering needs 40 min to reach a similar level of conversion.

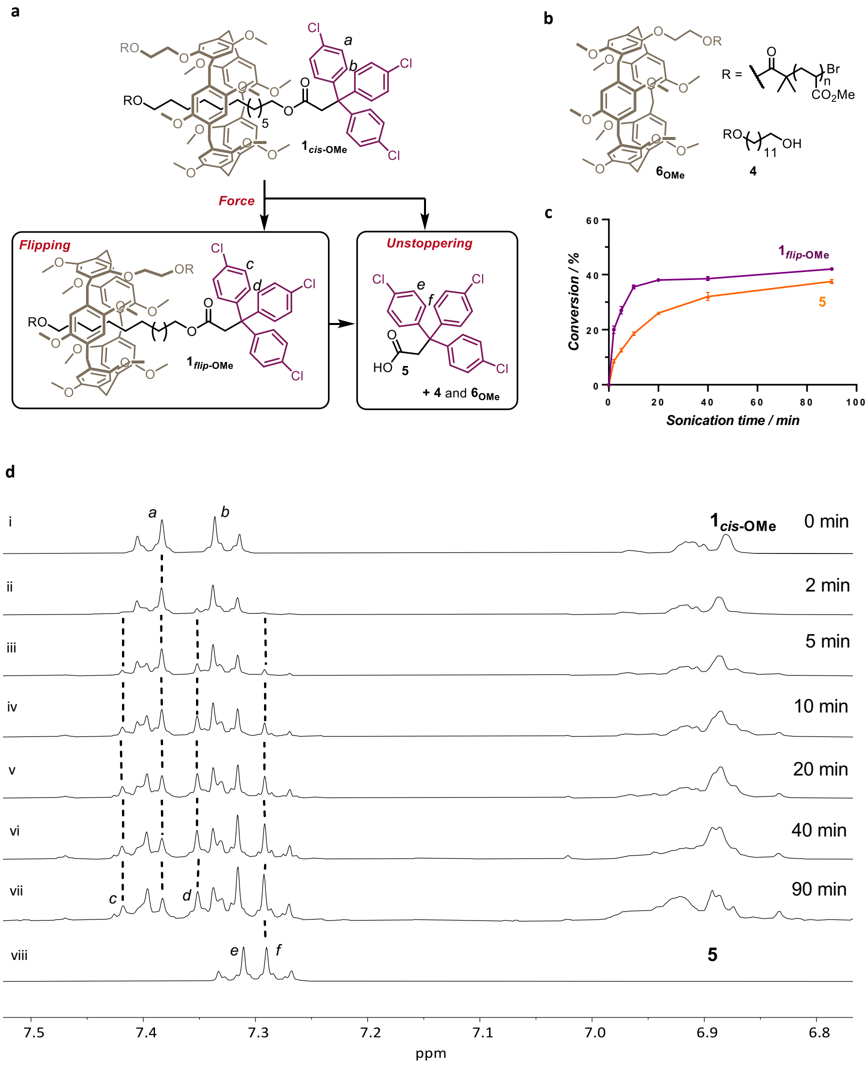

Figure S20. Sonications of polymer $\mathbf{1}_{cis\text{-OMe}}$ in MeCN/H_2O (50/1) in different sonication time. (a) Sonication of polymer $\mathbf{1}_{cis\text{-OMe}}$ affords flipped rotaxane polymer $\mathbf{1}_{flip\text{-OMe}}$ from flipping process, and fragments **5**, **4** and $\mathbf{6}_{OMe}$ from the unstoppering via either initial polymer or flipped polymer. (b) Structures of fragments **4** and $\mathbf{6}_{OMe}$. (c) Comparison of conversion of flipped product $\mathbf{1}_{flip\text{-OMe}}$ (purple) and unstoppering product **5** (orange) over the sonication time. (d) Partial ^{1}H NMR (400 MHz, Acetone-d_6, 298 K) spectra comparison of polymer $\mathbf{1}_{cis\text{-OMe}}$ before (i), after sonication with 2, 5, 10, 20, 40, and 90 min (ii-vii), and reference **5** (vi). Note: Data of 90 min sonication is from section 5.2.

5.7.2 Comparison of Dissociation Rate of Polymers $1_{cis\text{-OMe}}$ and $1_{trans\text{-OMe}}$

The sonications of $1_{cis\text{-OMe-166}}$ and $1_{trans\text{-OMe}}$ were performed MeCN/H_2O (50/1) following the general procedure described above. Aliquots of 200 µL were taken at sonication times of: 0, 1, 2, 3, 4, 5, 6, 8, 10, 15, 40 and 90 min. The solvent was evaporated off under a stream of N_2 and redissolved in 700 µL of THF. The sample was filtered through a syringe filter (PTFE, 0.45 µm pore size) and analysed by SEC. Upon sonication, the M_n reduces to ~50 kDa. The apparent initial rate constant (*k**) was derived from first 6 minutes of sonication using Nalepa's method (**Table S2**, **Figure S21-S23**).[6]

Table S2. M_n and k* values for polymers $1_{cis\text{-OMe-166}}$ and $1_{trans\text{-OMe}}$.

Mechanophore polymer		M_n/ kDa	k*/ $min^{-1}.kDa^{-1}.10^5$				T-test of *k**
			Run 1	Run 2	Average	SD	
$1_{cis\text{-OMe-166}}$	*Cis*	166	36.8	36.1	36.5	0.3	Significant difference (P = 0.03)
$1_{trans\text{-OMe}}$	*Trans*	163	39.4	40.4	39.9	0.5	

Note: First seven points were used for the calculation of *k** due to the secondary cleavage after 6 min sonication time.

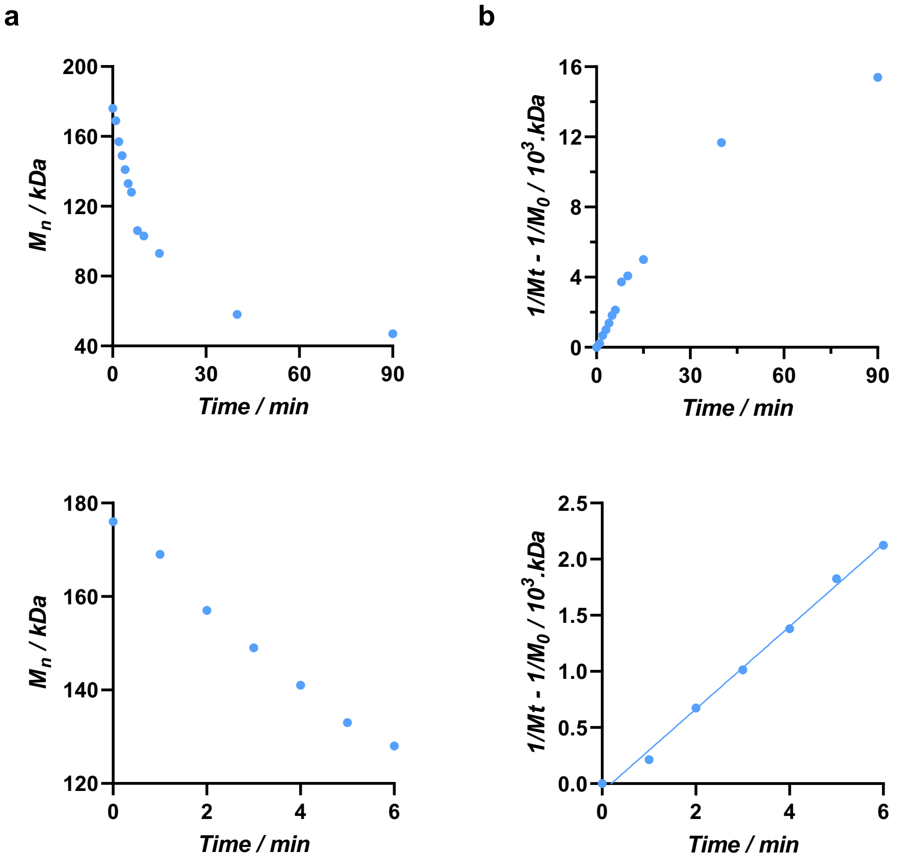

Figure S21. Kinetics of polymer $1_{cis\text{-OMe-166}}$, showing M_n decay over the course of the sonication (a) and the determination of the apparent rate constant *k** (b). Note: full (above) and zoom-in with linearisation (below) profiles are shown.

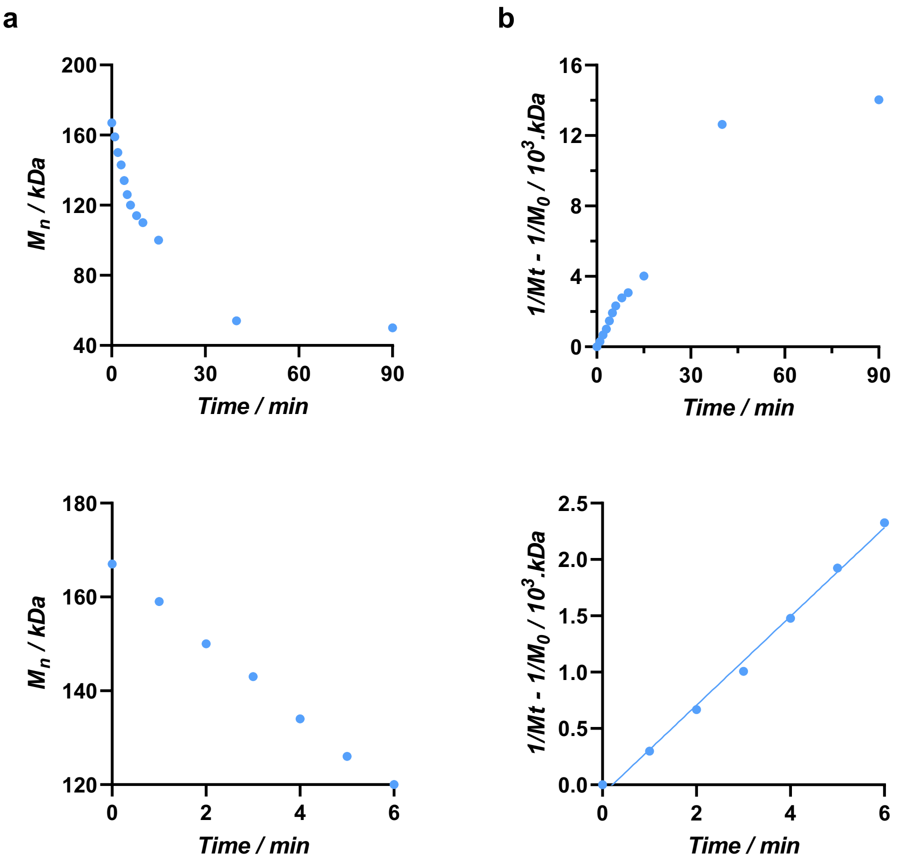

Figure S22. Kinetics of polymer $1_{trans\text{-OMe}}$, showing M_n decay over the course of the sonication (a) and the determination of the apparent rate constant k^* (b). Note: full (above) and zoom-in with linearisation (below) profiles are shown.

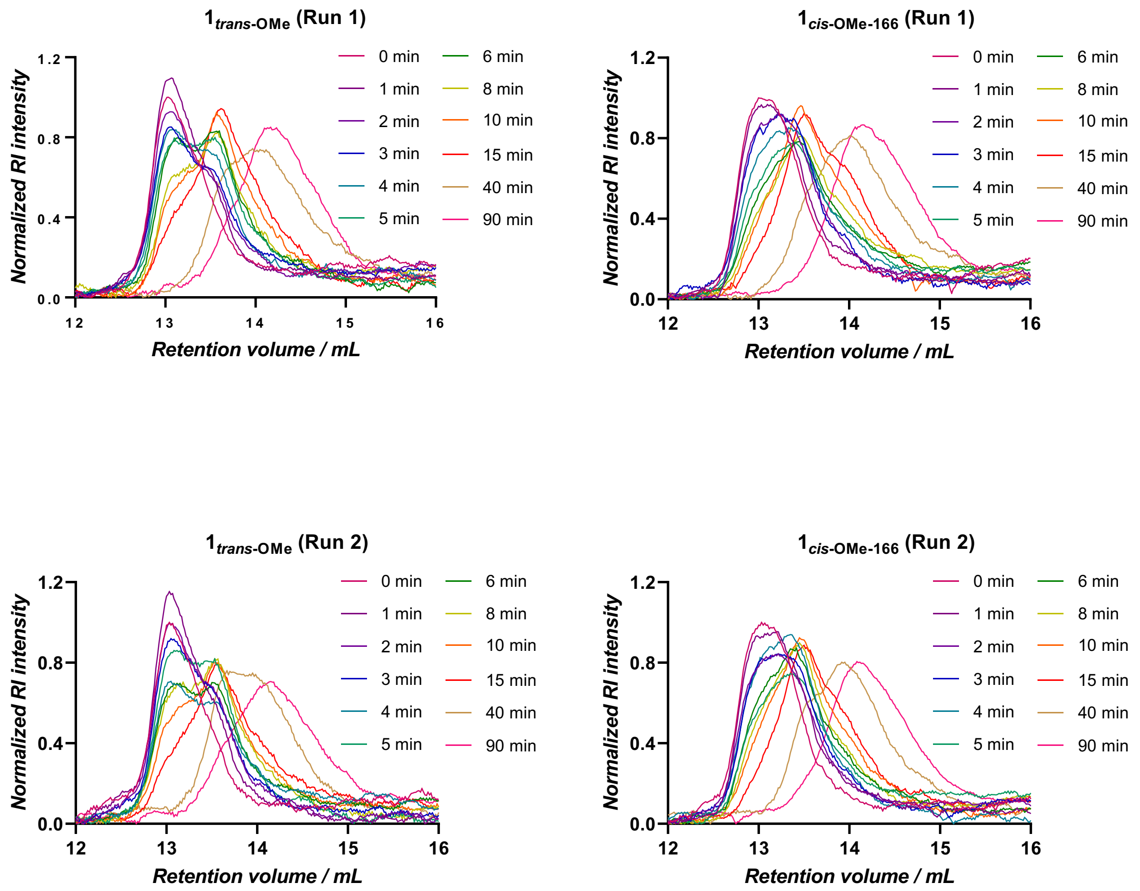

Figure S23. Kinetics of polymers $\mathbf{1}_{cis\text{-}OMe\text{-}166}$ and $\mathbf{1}_{trans\text{-}OMe}$ carried out in duplicate, showing area-normalised response traces.

5.8 Determination of Flipped Polymer $\mathbf{1}_{\textit{flip}\text{-OMe}}$ and the Thermal Stability

We determined the structure of the ring-flipped rotaxane via 1D selective NOESY. In rotaxane $\mathbf{1}_{\textit{cis}\text{-OMe-145}}$, the aromatic protons (such as *a*, *c*, *d*, *f* in **Figure S24**a) of the pillar[5]arene macrocycle exist in a relatively compact multiplet due to its pseudo-symmetric structure. However, when one hydroquinone ring is flipped, the aromatic protons (*a′*, *d′* in **Figure S24**a) on the flipped ring now face the aromatic protons (*c′*, *f′* in **Figure S24**a) of the adjacent hydroquinone rings. This spatial proximity can be revealed in a NOESY experiment. In fact, the comparison of the ^{1}H NMR spectra (**Figure S25**c) of polymers $\mathbf{1}_{\textit{cis}\text{-OMe-145}}$, $\mathbf{2}_{\textit{cis}}$ and $\mathbf{3}_{\textit{cis}}$ before and after sonication showed, in the polymer containing a flipped ring such as $\mathbf{1}_{\textit{cis}\text{-OMe-145}}$ and $\mathbf{2}_{\textit{cis}}$, the presence of new aromatic peaks (*d, e* or *f, g*) shifted upfield from the rest of the pillar[5]arene aromatic peaks (a_x-c_x, x = 1 – 4, **Figure S25**$c_{ii,iv}$). We hypothesised that these upshifted peaks belong to the flipped hydroquinone ring of pillar[5]arene, as the ring inversion would change their environment as explained above. Since different stoppers in $\mathbf{1}_{\textit{flip}\text{-OMe}}$ and $\mathbf{2}_{\textit{flip}}$ have a different effect on these aromatic peaks, we attributed the peaks with the largest shifts to the proton facing the stopper (*e*, *g*).

The ring flipping was confirmed by 1D selective NOESY ^{1}H NMR experiments as the excitation of proton $H_{a'}$ and $H_{d'}$ of polymer $\mathbf{1}_{\textit{cis}\text{-OMe-145}}$ after sonication and MeOH wash, showed a correlation signal with protons $H_{b',c'}$ and $H_{e',f'}$ respectively (**Figure S24**a-b).

Finally, the thermal stability of the flipped-ring rotaxane was assessed. We found that the structure was left unchanged after being heated at 85 °C for 24 h (**Figure S24**c).

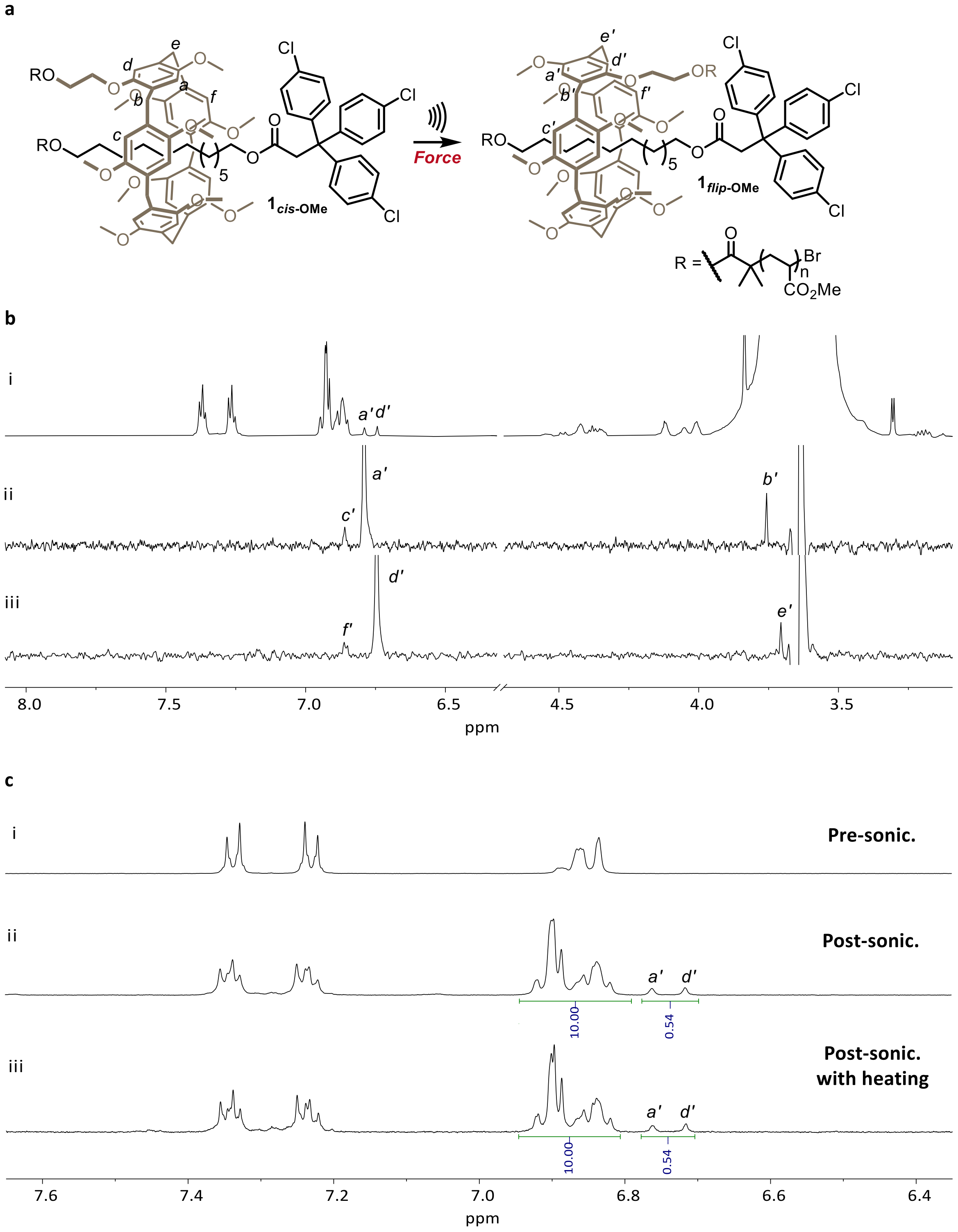

Figure S24. (a) Polymer $\mathbf{1}_{cis\text{-OMe-145}}$ affords flipped polymer $\mathbf{1}_{flip\text{-OMe}}$ after activation. (b) Partial ^{1}H NMR (700 MHz, Acetonitrile-d_6, 298 K) spectra of polymer $\mathbf{1}_{cis\text{-OMe-145}}$ after sonication and MeOH wash (i) and partial 1D selective NOESY ^{1}H NMR (700 MHz, Acetonitrile-d_6, 298 K) spectra of polymer $\mathbf{1}_{cis\text{-OMe-145}}$ after sonication and MeOH wash after excitation of protons $H_{a'}$ (ii), and $H_{d'}$ (iii). And (c) partial ^{1}H NMR (500 MHz, Acetonitrile-d_6, 298 K) spectra of polymer $\mathbf{1}_{cis\text{-OMe-145}}$ before (i), after (ii) sonication and MeOH wash, and post-sonication polymer with heating (iii) at 85 °C for 24 h in acetonitrile-d_6.

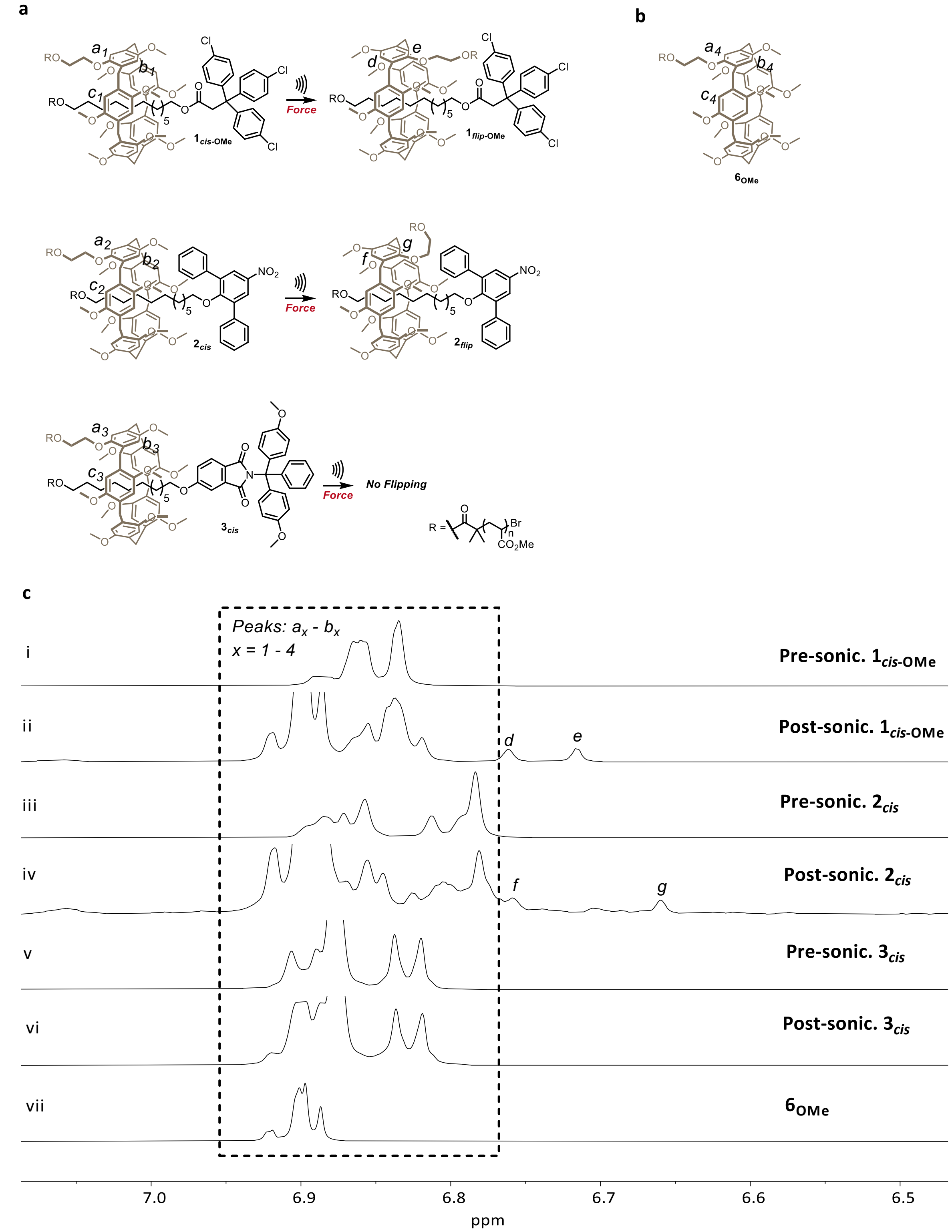

Figure S25. (a) Mechanical activation of polymers $\mathbf{1}_{cis\text{-OMe-145}}$, $\mathbf{2}_{cis}$ and $\mathbf{3}_{cis}$ and (b) reference polymer $\mathbf{6}_{OMe}$. (c) Partial ^{1}H NMR (500 MHz, Acetonitrile-d_6, 298 K) spectra of pre- and post-sonication polymer $\mathbf{1}_{cis\text{-OMe-145}}$ (i, ii), $\mathbf{2}_{cis}$ (iii, iv) and $\mathbf{3}_{cis}$ (v, vi) after MeOH wash and reference polymer $\mathbf{6}_{OMe}$ (vii).

5.9 Sonication of Control Polymers S20 and S21

Sonication of control polymers **S20** or **S21**, using the methodology described in the general procedure (section 5.1) and with the solvent used being MeCN/H_2O (50/1), was carried out to determine the stability of the axles/stoppers without rotaxane actuator. SEC analysis of the sonicated polymers showed complete cleavage (M_n of the post-sonication material was less than half of that of the pre-sonication polymer). Comparison of the ^1H NMR spectra of polymers before and after sonication and their reference compounds confirmed the stability of these structures in the absence of a rotaxane actuator (**Figure S26-26**).

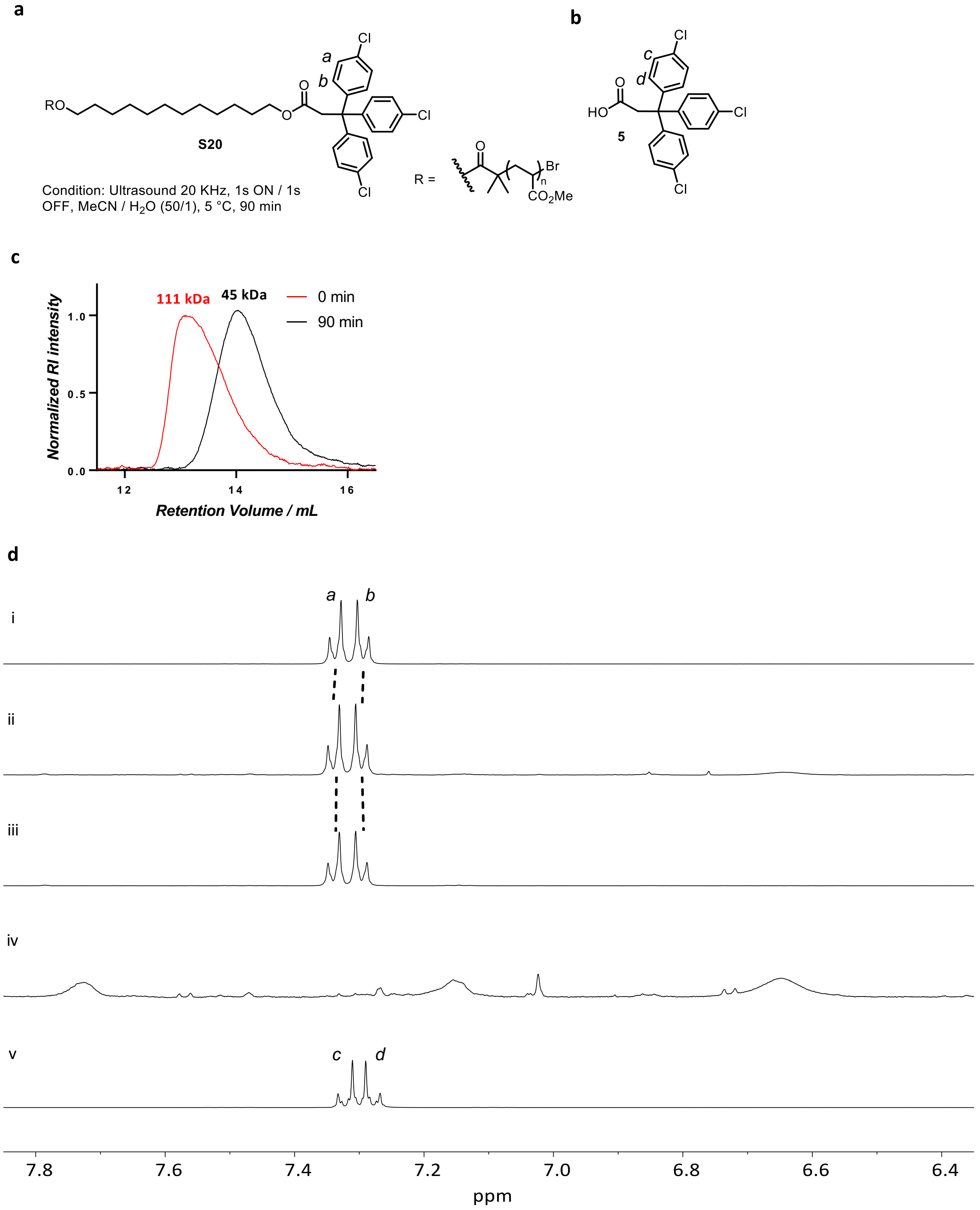

Figure S26. Sonication of polymer **S20** in MeCN/H_2O (50/1). (a) Structure of polymer **S20** and condition of sonication. (b) Reference **5**. (c) SEC traces of polymer **S20** before (red) and after (black) sonication. (d) Partial ^1H NMR (500 MHz, Acetone-d_6, 298 K) spectra comparison of polymer **S20** before (i), after sonication (ii), and after sonication and MeOH wash (iii), MeOH extract (iv), and reference **5** (v).

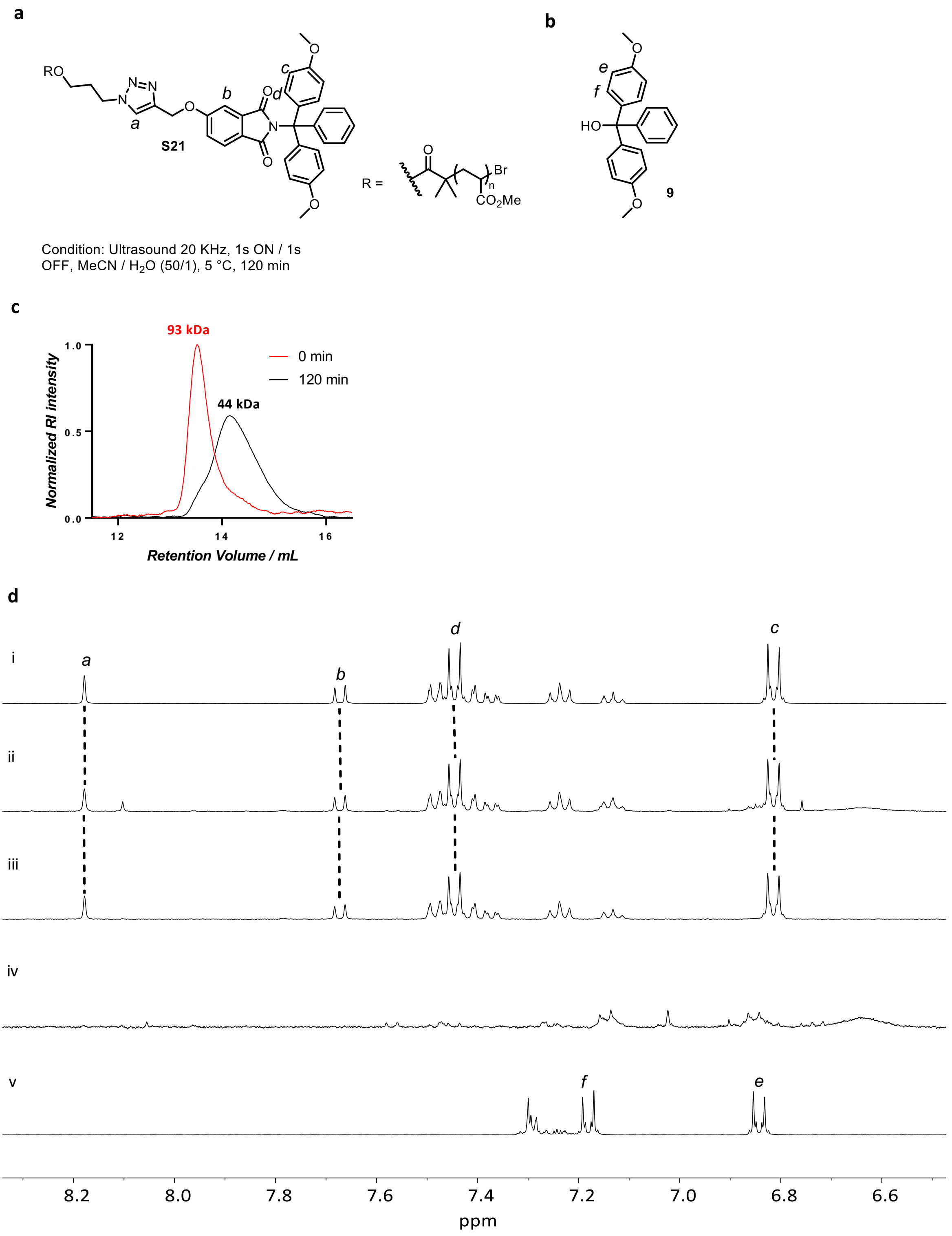

Figure S27. Sonication of polymer **S21** in MeCN/H_2O (50/1). (a) Structure of polymer **S21** and condition of sonication. (b) Reference **9**. (c) SEC traces of polymer **S21** before (red) and after (black) sonication. (d) Partial ^1H NMR (400 MHz, Acetone-d_6, 298 K) spectra comparison of polymer **S21** before (i), after sonication (ii), and after sonication and MeOH wash (iii), MeOH extract (iv), and reference **9** (v).

6 Calculation of Extent of Mechanophore Activation

6.1 Calculations for Polymer $\mathbf{1}_{\mathit{cis}\text{-OMe}}$

Here we use sonication (run 1) of polymer $\mathbf{1}_{\mathit{cis}\text{-OMe-145}}$ as an example of how we calculated the extent of flipping and unstoppering in polymer $\mathbf{1}_{\mathit{cis}\text{-OMe}}$.

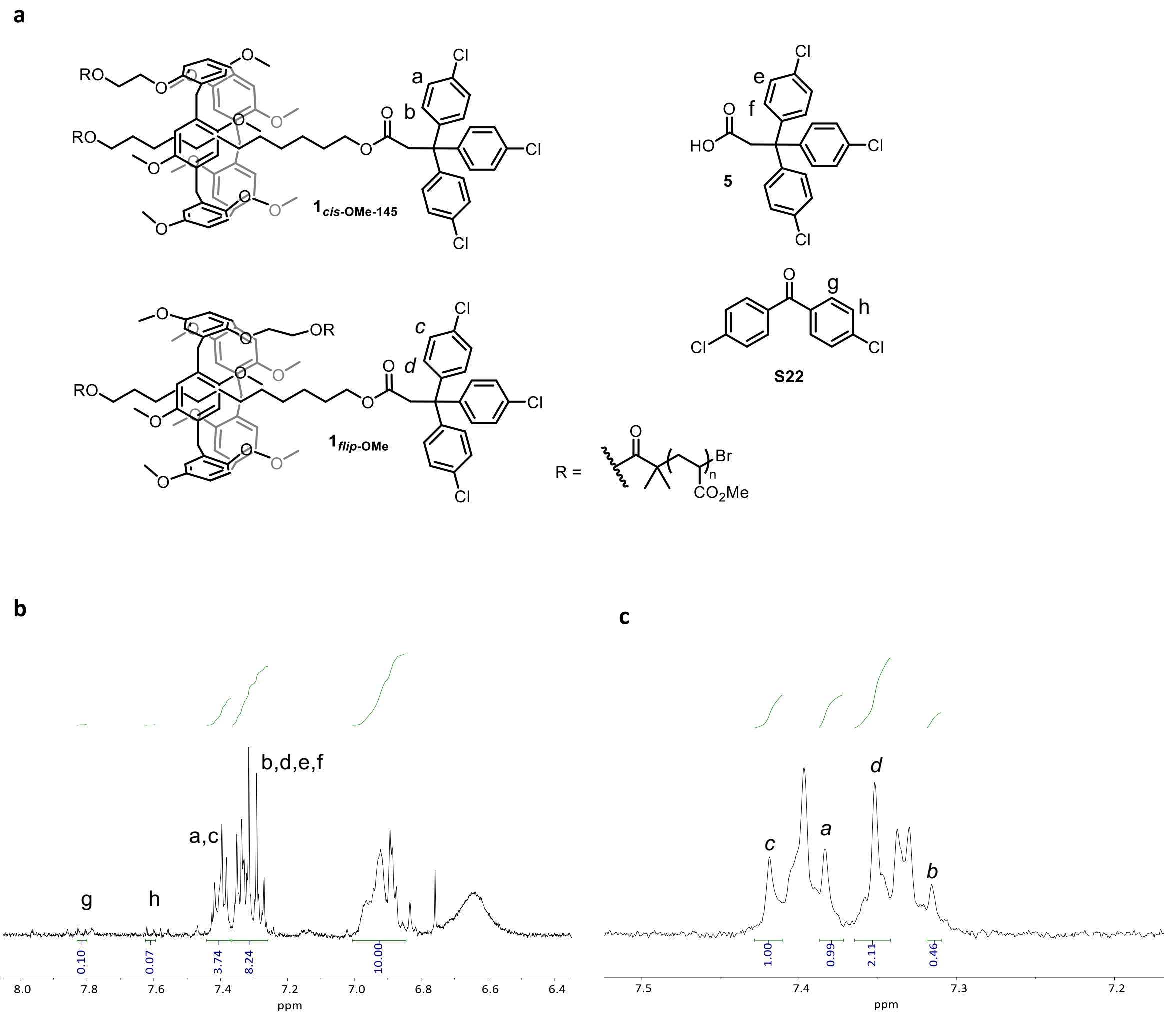

Figure S28. (a) Polymer $\mathbf{1}_{\mathit{cis}\text{-OMe}}$ affords flipped polymer $\mathbf{1}_{\mathit{flip}\text{-OMe}}$, and fragment **5**, **S22** after activation. And, partial ^{1}H NMR (400 MHz, Acetone-d_6, 298 K) spectra of post-sonication polymer $\mathbf{1}_{\mathit{cis}\text{-OMe}}$ before (b) and after (c) being washed with methanol.

Here, we use values determined from integration of various specific signals in the ^{1}H NMR spectra of the mechanophore-containing polymer post-sonication, both before and after being washed with methanol. The nomenclature of the designation for each of these values is as follows:

$\boldsymbol{I}^{\mathbf{x}}_{\mathbf{y}}$, where ***I*** represents a total numerical value of integration, **x** represents the ^{1}H NMR spectrum being used (β = post-sonication polymer before being washed with methanol –**Figure S28***b*, and γ = post-sonication polymer after being washed with methanol – **Figure S28***c*), and **y** represents a list of the signal designations being integrated. It should be noted that, peaks $H_{a,b}$ and $H_{c,d}$ requiring integration partially overlap with each other; the symmetric mulitplets of $H_{a,b}$ and $H_{c,d}$ allow us to take the non-overlapped part of the multiplet as the half-integration of the total peak which we then carry through

to our calculations.

The percentage of released **5** (C_{US}) via unstoppering, and **S22** (C_{DB}) via degradation having occurred during the activation, were determined with the formula below:

$$C_{US} = \frac{I^{\beta}{}_{b,d,e,f} - I^{\beta}{}_{a,c}}{I^{\beta}{}_{g} + I^{\beta}{}_{h} + I^{\beta}{}_{a,c} + I^{\beta}{}_{b,d,e,f}} \times 100\%$$

$$C_{DB} = \frac{I^{\beta}{}_{g} + I^{\beta}{}_{h}}{I^{\beta}{}_{g} + I^{\beta}{}_{h} + I^{\beta}{}_{a,c} + I^{\beta}{}_{b,d,e,f}} \times 100\%$$

In this case, $\boldsymbol{C_{US}}$ **= 37%** [(8.27–3.74) / (0.10+0.07+3.74 +8.24) × 100%], and $\boldsymbol{C_{DB}}$ **= 1%** [(0.10 +0.07) / (0.10 +0.07 +3.74 +8.24) × 100%].

Then the percentage of flipped rotaxane (C_{Flip}) and intact rotaxane (C_R) was determined by the formula below:

$$C_{Flip} = \frac{I^{\gamma}{}_{\frac{c,d}{2}}}{I^{\gamma}{}_{\frac{c,d}{2}} + I^{\gamma}{}_{\frac{a,b}{2}}} \times (1 - \mathrm{C}_{US} - \mathrm{C}_{DB}) \times 100\%$$

$$C_{R} = \frac{I^{\gamma}{}_{\frac{a,b}{2}}}{I^{\gamma}{}_{\frac{c,d}{2}} + I^{\gamma}{}_{\frac{a,b}{2}}} \times (1 - \mathrm{C}_{US} - \mathrm{C}_{DB}) \times 100\%$$

In this case, $\boldsymbol{C_{Flip}}$ **= 42%**; [(1.00+2.11) / (1.00+2.11+0.99+0.46) × (1-0.37-0.01) ×100%], and $\boldsymbol{C_{R}}$ **= 20%**; [(0.99+0.46) / (1.00+2.11+0.99+0.46) × (1-0.37-0.01) × 100%].

6.2 Calculations for Polymer $\mathbf{3}_{cis}$

Here we use sonication (run 1) of polymer $\mathbf{3}_{cis}$ as an example of how we calculated the extent of unstoppering in polymer $\mathbf{3}_{cis}$.

a

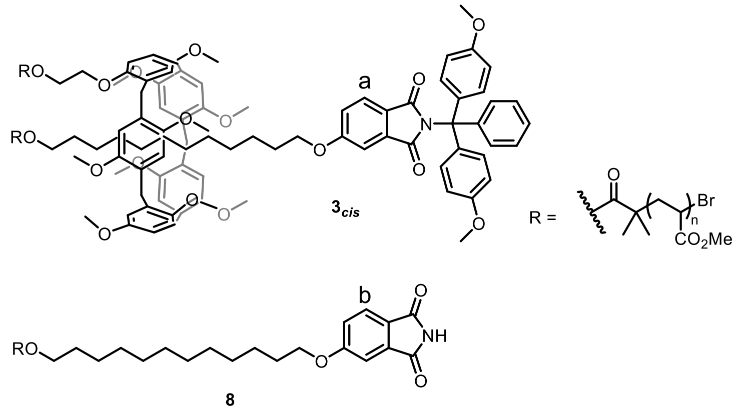

b

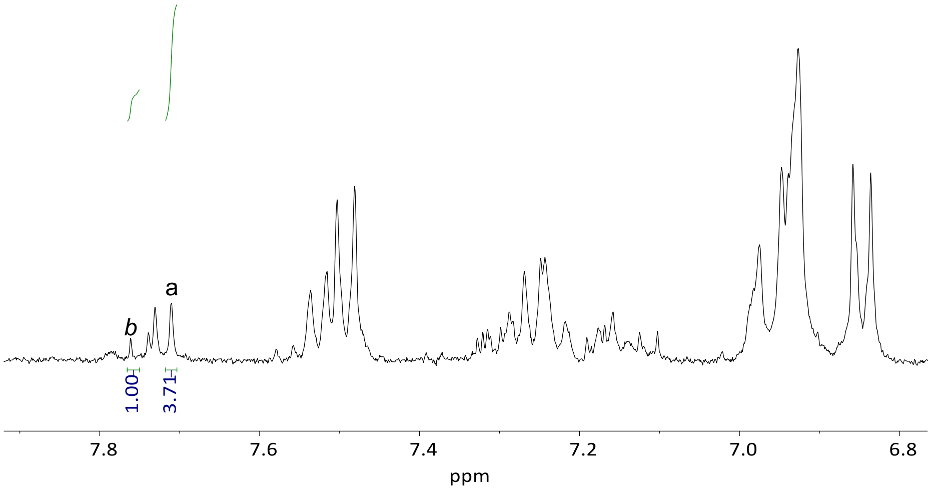

Figure S29. (a) Polymer $\mathbf{3}_{cis}$ affords polymer fragment **8** after activation. And, (b) partial ^{1}H NMR (400 MHz, Acetone-d_6, 298 K) spectra of post-sonication polymer $\mathbf{3}_{cis}$ before being washed with methanol.

It should be noted that, peaks H_a and H_b requiring integration partially overlap with each other; the symmetric doublet of H_a and H_b allow us to take the non-overlapped part of the doublet as the half-integration which we then carry through to our calculations.

The percentage of released **9** (C_{US}) via unstoppering and intact rotaxane (C_R) were determined by the formula below:

$$C_{US} = \frac{I_{\frac{b}{2}}}{I_{\frac{a}{2}} + I_{\frac{b}{2}}} \times 100\%$$

$$C_R = \frac{\frac{I_a}{2}}{\frac{I_a}{2} + \frac{I_b}{2}} \times 100\%$$

In this case, $\boldsymbol{C_{US}}$ **= 21%** [1.00/ (1.00+3.71) × 100%], and $\boldsymbol{C_R}$ **= 79%** [(3.71) / (1.00+3.71) × 100%].

6.3 Calculations for Polymers $\mathbf{1}_{cis\text{-OEt}}$ and $\mathbf{1}_{trans\text{-OMe}}$

Here we use sonication (run 1) of polymer $\mathbf{1}_{trans\text{-OMe}}$ as an example of how we calculated the extent of unstoppering in polymers $\mathbf{1}_{cis\text{-OEt}}$ and $\mathbf{1}_{trans\text{-OMe}}$.

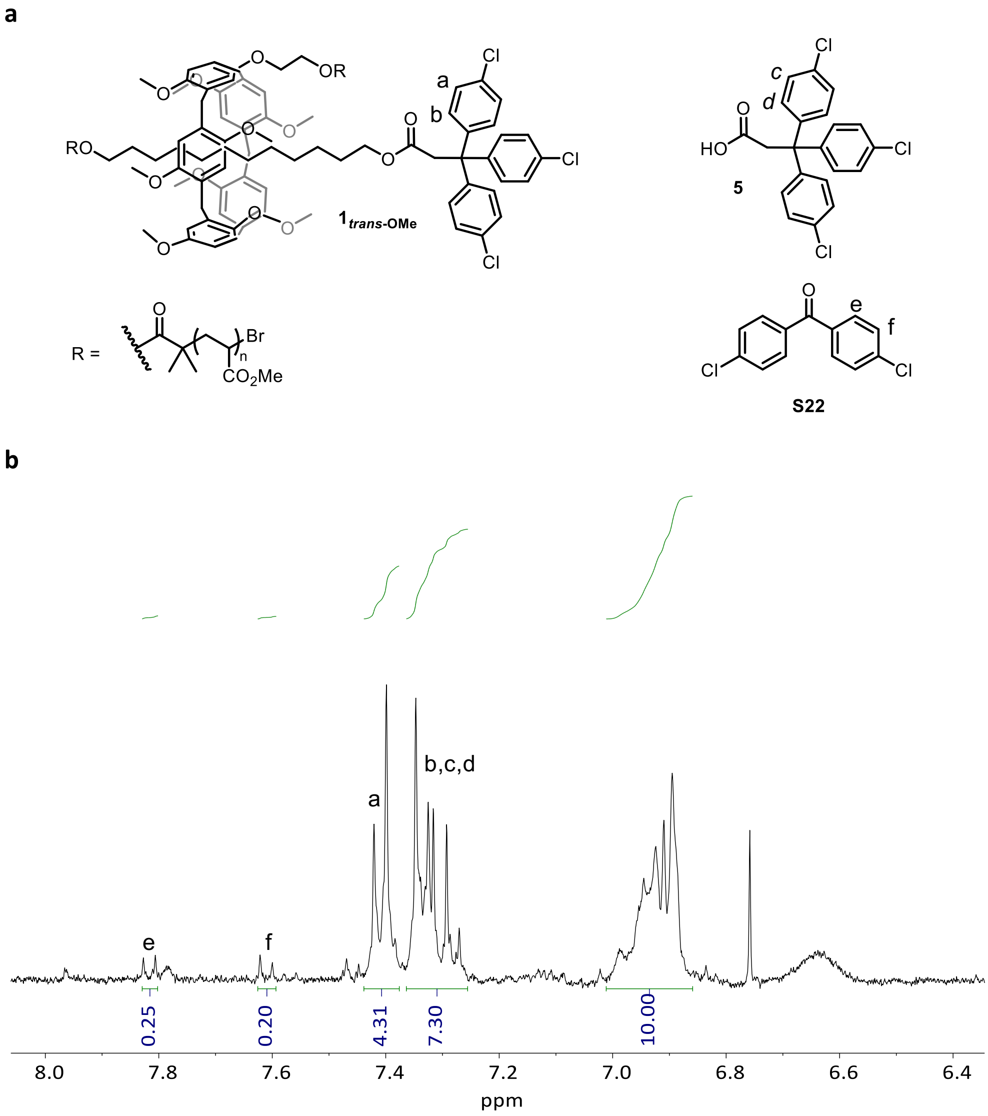

Figure S30. (a) Polymer $\mathbf{1}_{trans\text{-OMe}}$ affords fragment **5** after activation. And, (b) partial ^{1}H NMR (400 MHz, Acetone-d_6, 298 K) spectra of post-sonication polymer $\mathbf{1}_{trans\text{-OMe}}$ before being washed with methanol.

The percentage of released **5** (C_{US}) via unstoppering, **S22** (C_{DB}) via degradation and intact rotaxane (C_R) were determined by the formula below:

$$C_{US} = \frac{I_{b,c,d} - I_a}{I_e + I_f + I_a + I_{b,c,d}} \times 100\%$$

$$C_{DB} = \frac{I_e + I_f}{I_e + I_f + I_a + I_{b,c,d}} \times 100\%$$

$$C_R = \frac{2I_a}{I_e + I_f + I_a + I_{b,c,d}} \times 100\%$$

In this case, ***C_{US}* = 25%** [(7.30-4.31) / (0.25+0.20+4.31+7.30) × 100%], ***C_{DB}* = 4%** [(0.25+0.20) / (0.25+0.20+4.31+7.30) × 100%], and ***C_R* = 71%** [2*4.31/ (0.25+0.20+4.31+7.30) × 100%].

6.4 Calculations for Polymer 2*cis*

Here we use sonication (run 1) of polymer **2*cis*** as an example of how we calculated the extent of flipping and unstoppering in polymer **2*cis***.

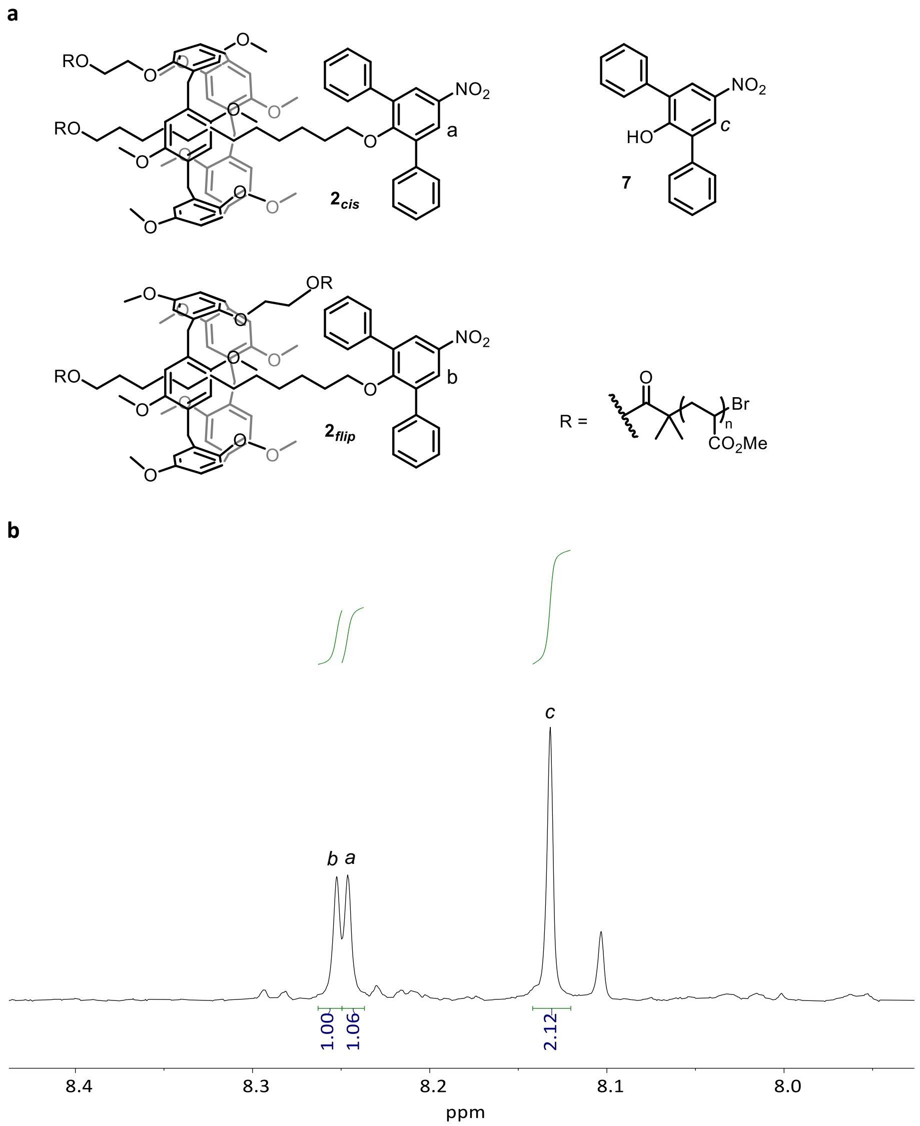

Figure S31. (a) Polymer **2*cis*** affords flipped polymer **2*flip***, and fragment **7** after activation. And, (b) partial ^{1}H NMR (500 MHz, Acetone-d_6, 298 K) spectra of post-sonication polymer **2*cis*** before being washed with methanol.

the percentage of unstoppering (C_{US}), Flipped rotaxane (C_{Flip}) and intact rotaxane (C_R) was determined by the formula below:

$$C_{US} = \frac{I_c}{I_a + I_b + I_c} \times 100\%$$

$$C_{Flip} = \frac{I_b}{I_a + I_b + I_c} \times 100\%$$

$$C_R = \frac{I_a}{I_a + I_b + I_c} \times 100\%$$

In this case, $\boldsymbol{C_{US}}$ **= 51%** [2.12 / (1.00+1.06+2.12) × 100%], $\boldsymbol{C_{Flip}}$ **= 25%** [1.06 / (1.00+1.06+2.12) × 100%], and $\boldsymbol{C_R}$ **= 24%** [1.00 / (1.00+1.06+2.12) × 100%].

6.5 Summary of Mechanical Activation

Table S3. Analysis of mechanical activation *via* ultrasound of mechanophore and control polymers.

No.	Mechanophore polymer			Sonic. solvent	Pre-sonication		Post-sonication		Mechanophore activation (%)								F_{max} (nN)
					M_n (kDa)	*Đ*	M_n (kDa)	*Đ*	Intact Mechanophpore		Flipping		Unstoppering		Ketone formation		
									Individual	Average	Individual	Average	Individual	Average	Individual	Average	
**1**	**1**$_{cis\text{-OMe-145}}$	***Cis***	**Run 1**	**MeCN/H$_2$O**	145	1.16	51	1.27	20	19	42	42	37	38	1	1	4.5
**2**			**Run 2**				47	1.38	19		42		38		2		
**3**	**1**$_{cis\text{-OEt}}$	***Cis***	**Run 1**		143	1.27	46	1.22	66	66	0	0	30	28	5	5	4.3
**4**			**Run 2**				46	1.24	67		0		27		6		
**5**	**3**$_{cis}$	***Cis***	**Run 1**		117	1.19	52	1.30	79	78	0	0	21	22	NA		5.8
**6**			**Run 2**				49	1.28	77		0		23				
**7**	**2**$_{cis}$	***Cis***	**Run 1**		138	1.14	52	1.27	24	23	25	25	51	52	NA		3.3
**8**			**Run 2**				47	1.39	23		25		53				
**9**	**1**$_{trans\text{-OMe}}$	***Trans***	**Run 1**		163	1.21	49	1.29	71	71	NA		25	25	4	4	4.6
**10**			**Run 2**				53	1.31	71				25		4		
**11**	**S20**	**Control**	**NA**		111	1.37	45	1.43	100	NA	NA	NA	0	NA	NA		NA
**12**	**S21**				93	1.16	44	1.31	100				0				
**13**	**1**$_{trans\text{-OMe}}$	***Trans***		**THF**	163	1.21	51	1.33	70				29		1	NA	
**14**	**1**$_{cis\text{-OMe-145}}$	***Cis***			145	1.16	45	1.30	24		42		33		0		

Table S4. Analysis of mechanical activation of mechanophore **1**$_{\textbf{\textit{cis}-OMe-145}}$ *via* ultrasound with various sonication time.

**No.**	**Sonic. time (min)**		**Sonic. solvent**	**Mechanophore activation (%)**							
				**Intact Mechanophpore**		**Flipping**		**Unstoppering**		**Ketone formation**	
				**Individual**	**Average**	**Individual**	**Average**	**Individual**	**Average**	**Individual**	**Average**
**1**	**2**	**Run 1**	**MeCN/H₂O**	70	72	21	20	9	9	0	0
**2**		**Run 2**		73		19		8		0	
**3**	**5**	**Run 1**		58	59	28	27	13	13	1	1
**4**		**Run 2**		61		26		12		1	
**5**	**10**	**Run 1**		45	45	35	36	19	18	1	1
**6**		**Run 2**		45		36		18		1	
**7**	**20**	**Run 1**		34	34	38	38	26	26	2	2
**8**		**Run 2**		34		38		26		2	
**9**	**40**	**Run 1**		27	27	38	38	33	32	2	2
**10**		**Run 2**		27		39		31		3	

7 Computational modelling

7.1 General method

CoGEF calculations were performed using Gaussian16 following Beyer's method.[7] The structure of the mechanophore was built in GaussianView6. The distance between the terminal methyl groups was constrained and increased by increments with 0.5 Å. Each step was run with DFT (B3LYP/6-31G) in vacuum. The relative energy of each intermediate was determined by setting the energy of the initial state at 0 kJ/mol. F_{max} values were determined from the slope of the final 3 points before bond scission.

We performed simulations using the External Force is Explicitly Included (EFEI) method to investigate the effect of mechanical forces on the rotaxane's molecular structures. The EFEI calculations were conducted using the semiempirical GFN2-xTB method, using ORCA 6.0.1 interfaced with xtb. ORCA allows for the direct inclusion of external force vectors during geometry optimizations, enabling the study of force-induced structural changes and mechanochemical behaviour. In our case, force was applied along a defined coordinate where atoms indicated in Fig. S39a were selectively subjected to directional force vectors.

To simulate the dynamic effects of external mechanical forces, we performed molecular dynamics (MD) simulations at the GFN2-xTB level of theory using the ORCA/xtb interface. Atomic positions were recorded every step of the trajectory. MD protocol was structured in two stages: an initial equilibration phase and a subsequent force-application phase. In both phases, temperature was controlled using a CSVR[8] thermostat set at 300 with a time constant of 20.0 fs.
Equilibration was started from an optimised geometry by assigning random atomic velocities were initialized at 300 K. The system was equilibrated for 2000 steps with a timestep of 0.5 fs. The desired temperature was reached after ~1000 steps (see Fig. S38).
In the force application phase, a mechanical force, acting on the selected atoms defined above (see Fig. S39a), was introduced. Force was applied through a spring constant that mimics a constant force pulling experiment acting on a collective variable defined as the distance between the selected atoms. The MD simulation then proceeded for an additional 10 000 steps at the given force.

In the MD simulations, external mechanical force was applied to the rotaxanes using harmonic restraints to selected atomic coordinates. The potential energy values reported in the output files include contributions from both intrinsic molecular interactions and the externally applied harmonic bias. To analyse the internal energy of the system independently of the external work, a correction was applied to remove the artificial potential energy associated with the applied force. The corrected energy was computed as:

$$E(t) = \left[E_{pot}(t) - \frac{1}{2}k\big(x_r - x(t)\big)^2\right]$$

Where: $E_{pot}(t)$ is the total potential energy at time (t) including both internal and restraint energy terms; k is the harmonic force constant used for the restraint; x_r is the target value of the restraint; and $x(t)$ is the value of the collective variable.
This correction removes the contribution of the harmonic restraint energy (the external mechanical work) from the potential energy. To facilitate comparison across simulations and improve visualisation, we set the energy baseline to the average of the second half of the equilibration phase, when the

desired temperature was reached (see above). This standardisation allows the relative energy changes to be plotted (ΔE) over time.

7.2 Computational modelling of $\mathbf{1}_{cis\text{-OMe}}$'

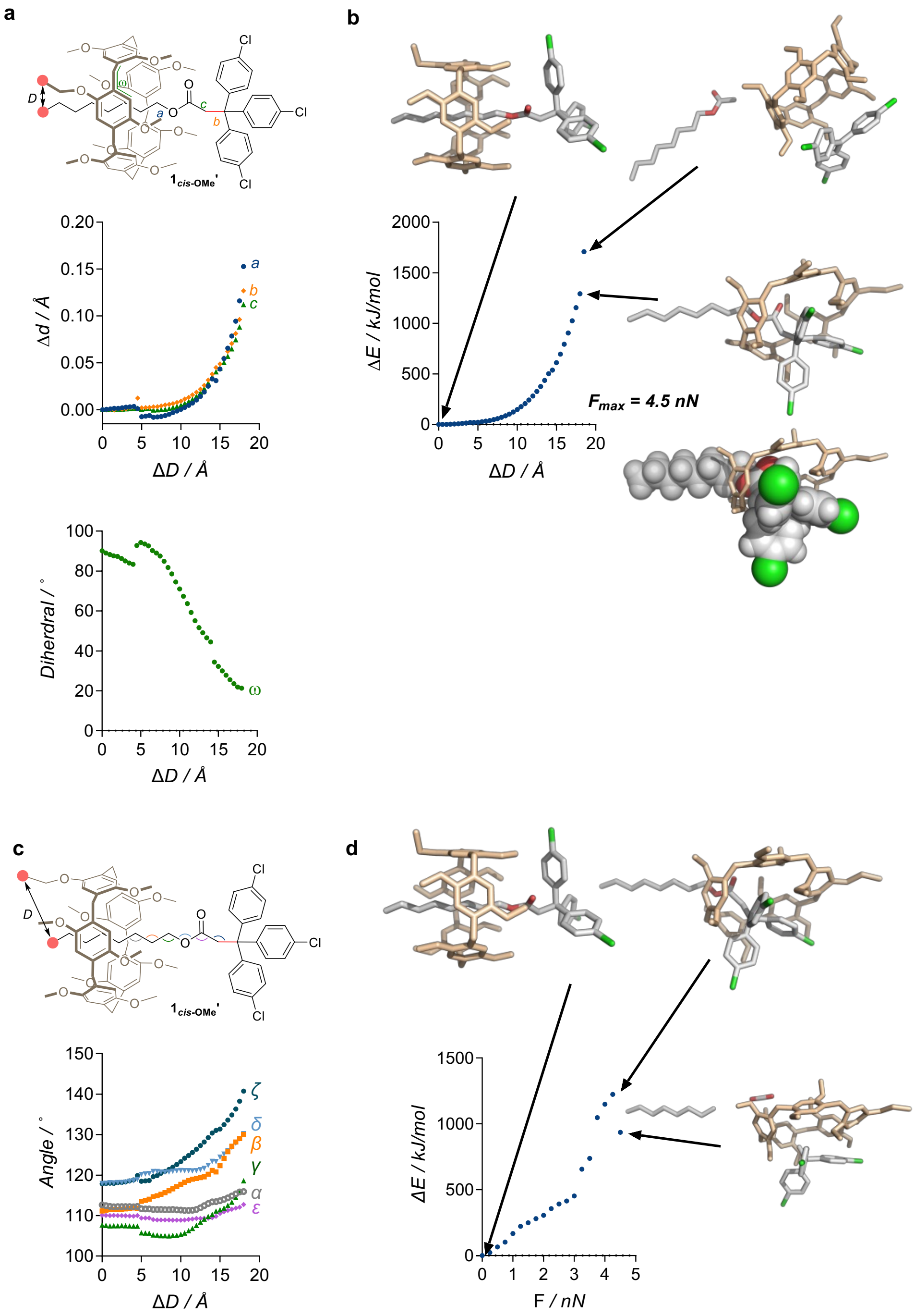

Figure S32. Evolution of bonds *a*, *b*, *c,* dihedral angle ω (a), energy (b), and angles *α*, *β*, *γ*, *δ*, *ε* and *ζ* (c) upon simulated elongation (CoGEF, DFT B3LYP/6-31G, vac) of model $\mathbf{1}_{cis\text{-OMe}}$'. Predicted scissile bonds are shown in red and anchor atoms are indicated by pink disks. The axle of the rotaxane structure just before scission is shown in both sticks and space-filling representation. Hydrogen atoms omitted for clarity in the sticks representation. (d) Evolution of energy of $\mathbf{1}_{cis\text{-OMe}}$' in function of the force applied (EFEI, GFN2-xTB, vac).

7.3 Computational Calculations of Model $1_{cis\text{-OEt}}'$

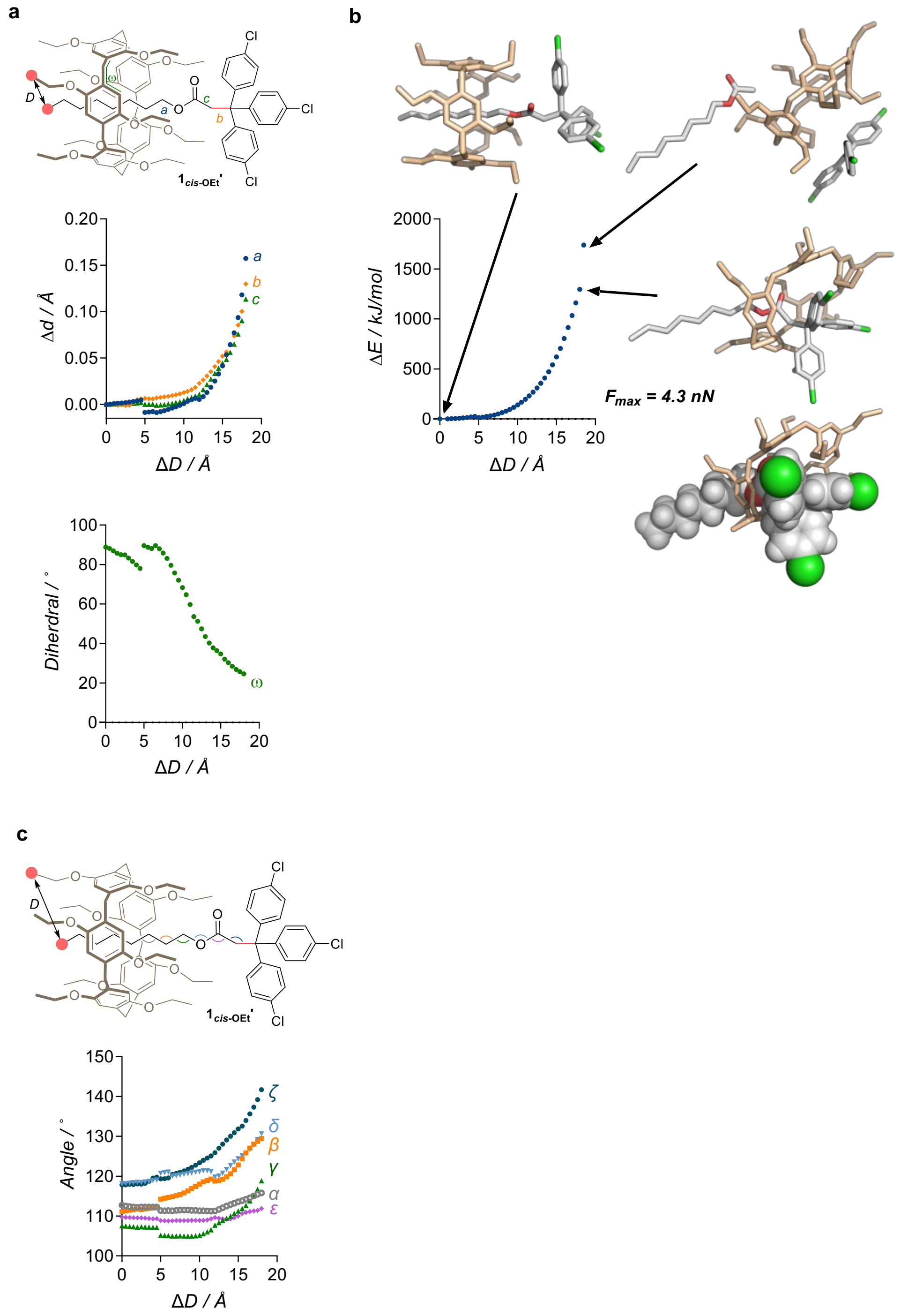

Figure S33. Evolution of bonds *a*, *b*, *c,* dihedral angle ω (a), and energy (b), and angles *α*, *β*, *γ*, *δ*, *ε* and *ζ* (c) upon simulated elongation (CoGEF, DFT B3LYP/6-31G, vac) of model $\mathbf{1}_{cis\text{-OEt}}$**'**. Predicted scissile bond is shown in red and anchor atoms are indicated by pink disks. The axle of the rotaxane structure just before scission is shown in both sticks and space-filling representation. Hydrogen atoms omitted for clarity in the sticks representation.

7.4 Computational Calculations of Model 3_{cis}'

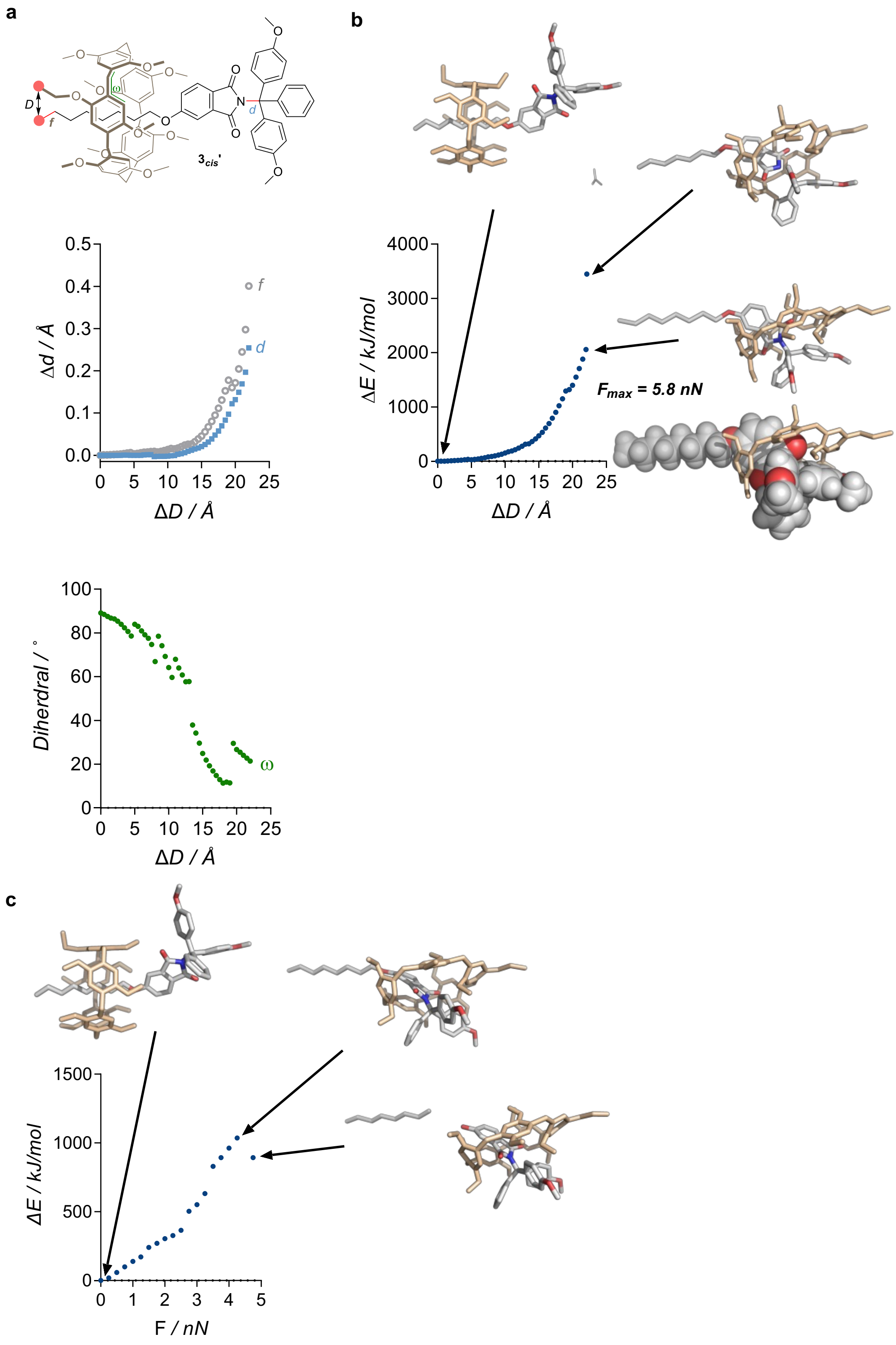

Figure S34. Evolution of bonds *f* and *d*, dihedral angle ω (a), and energy (b) upon simulated elongation (CoGEF, DFT B3LYP/6-31G, vac) of model $\mathbf{3_{cis}}$**'**. Predicted scissile bond is shown in red and anchor atoms are indicated by pink disks. The axle of the rotaxane structure just before scission is shown in both sticks and space-filling representation. Hydrogen atoms omitted for clarity in the sticks representation.

7.5 Computational Calculations of Model $\mathbf{1}_{trans\text{-}OMe}'$

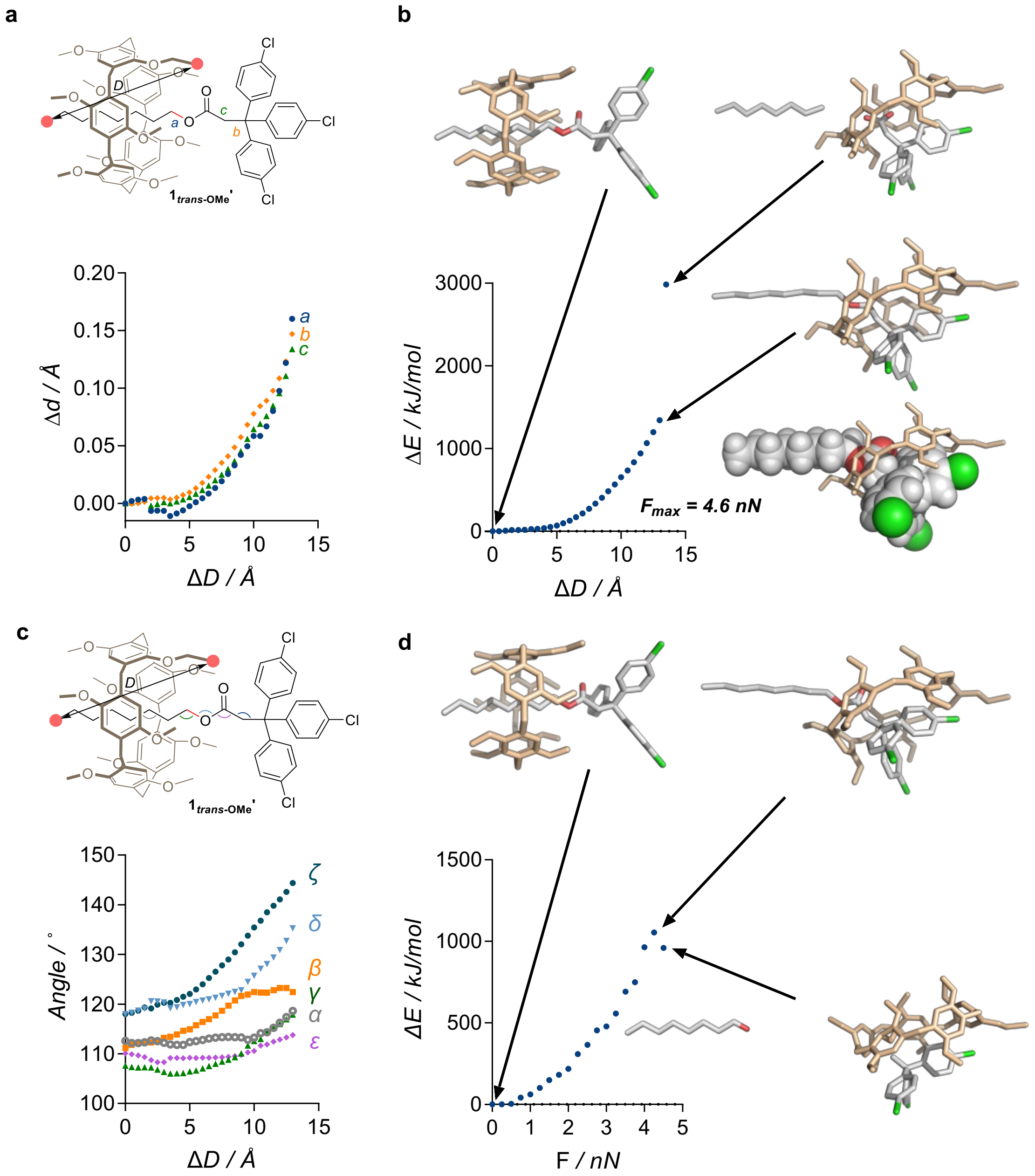

Figure S35. Evolution of bonds *a*, *b*, *c,* dihedral angle ω (a), energy (b), and angles *α*, *β*, *γ*, *δ*, *ε* and *ζ* (c) upon simulated elongation (CoGEF, DFT B3LYP/6-31G, vac) of model $\mathbf{1}_{trans\text{-}OMe}$**'**. Predicted scissile bonds are shown in red and anchor atoms are indicated by pink disks. The axle of the rotaxane structure just before scission is shown in both sticks and space-filling representation. Hydrogen atoms omitted for clarity in the sticks representation. (d) Evolution of energy of $\mathbf{1}_{trans\text{-}OMe}$**'** in function of the force applied (EFEI, GFN2-xTB, vac).

7.6 Computational Calculations of Model $2_{cis}'$

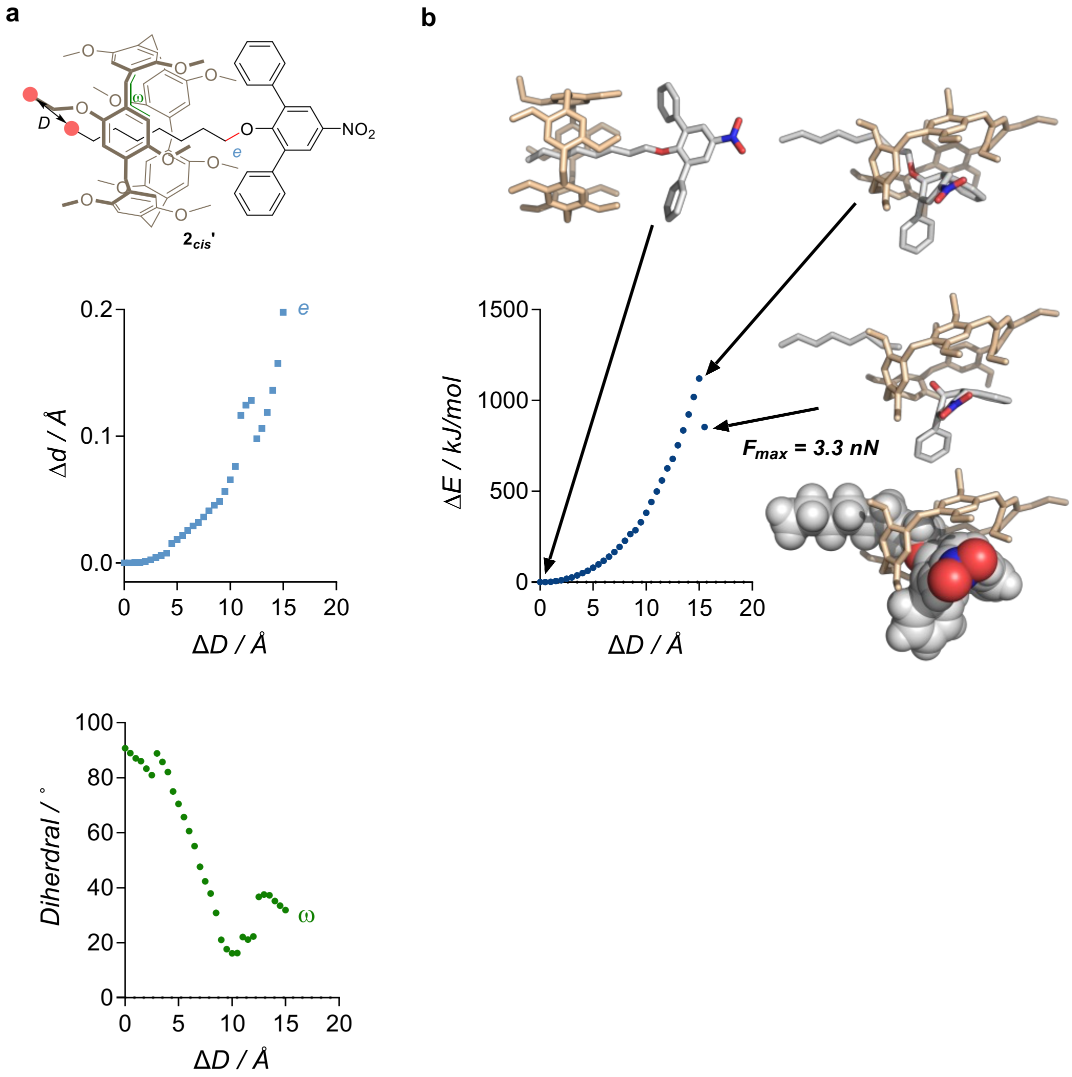

Figure S36. Evolution of bond *e*, dihedral angle ω (a), and energy (b) upon simulated elongation (CoGEF, DFT B3LYP/6-31G, vac) of model $\mathbf{2_{cis}'}$. Predicted scissile bond is shown in red and anchor atoms are indicated by pink disks. The axle of the rotaxane structure just before scission is shown in both sticks and space-filling representation. Hydrogen atoms omitted for clarity in the sticks representation.

7.7 Computational Calculations of Model $1_{flip\text{-}OMe}$'

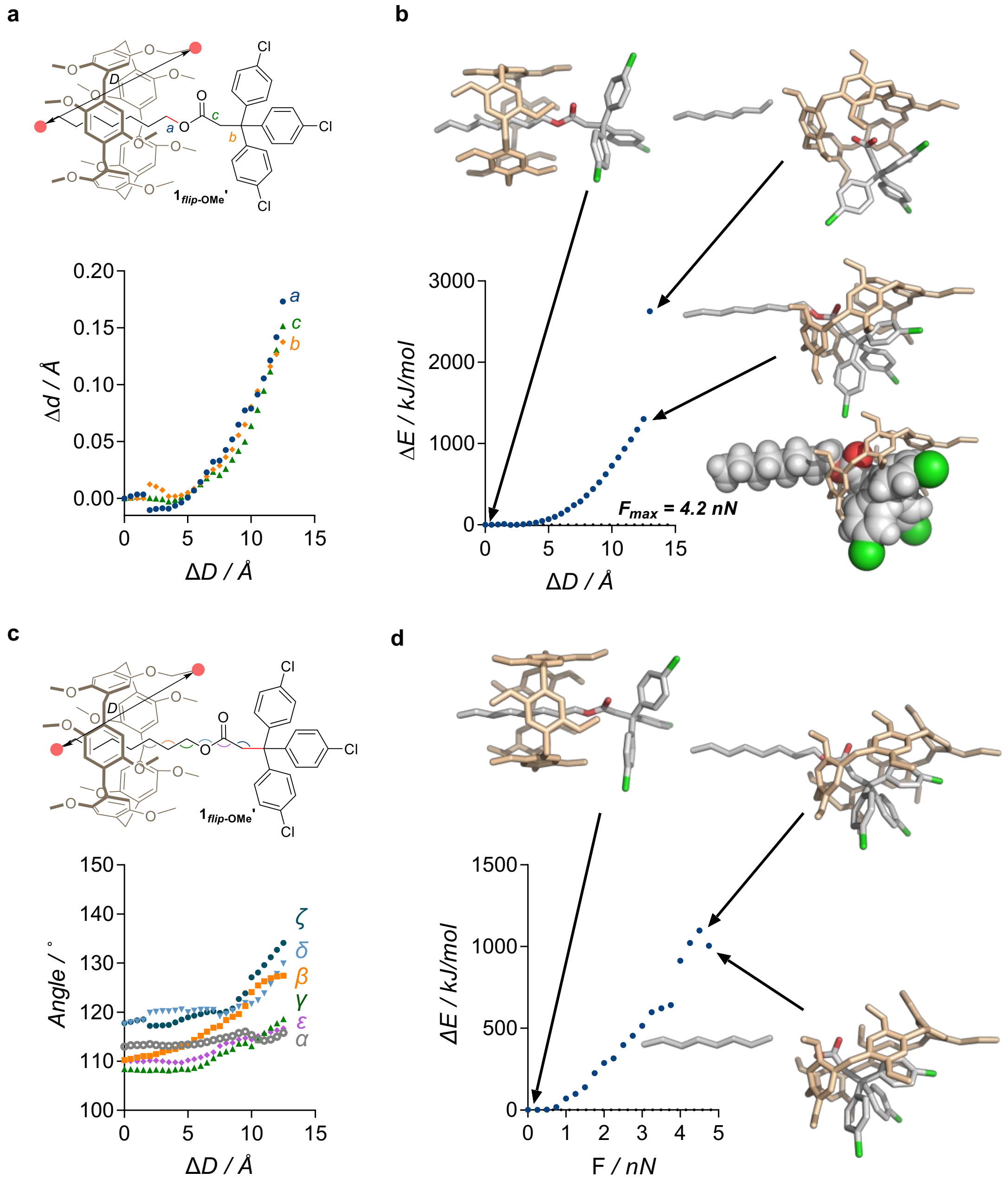

Figure S37. Evolution of bonds *a, b, c*, dihedral angle ω (a), and energy (b) upon simulated elongation (CoGEF, DFT B3LYP/6-31G, vac) of model $\mathbf{1}_{flip\text{-}OMe}$'. Predicted scissile bond is shown in red and anchor atoms are indicated by pink disks. The axle of the rotaxane structure just before scission is shown in both sticks and space-filling representation. Hydrogen atoms omitted for clarity in the sticks representation. (c) Evolution of energy of $\mathbf{1}_{flip\text{-}OMe}$' in function of the force applied (EFEI, GFN2-xTB, vac).

7.8 Comparison of Unstoppering Activation of Model $1_{trans\text{-OMe}}'$, $1_{flip\text{-OMe}}'$, $1_{cis\text{-OMe}}'$ and $1_{cis\text{-OEt}}'$ in Computational Simulation

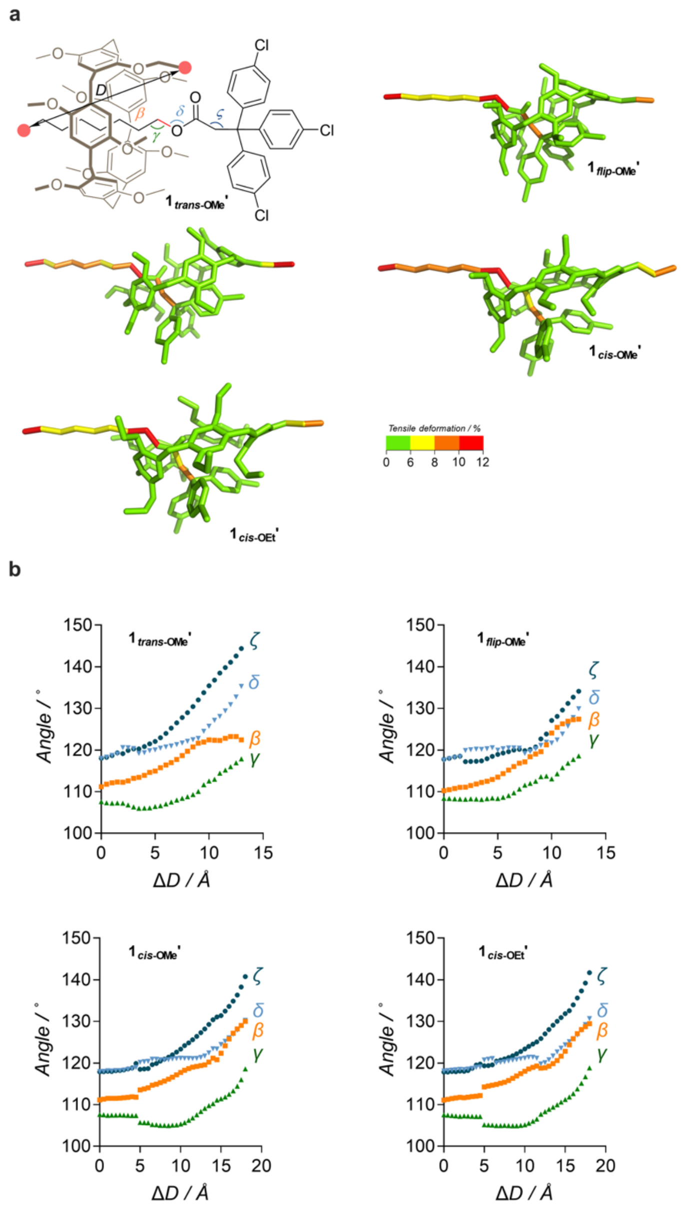

Figure S38. Computational investigation of unstoppering activation in *trans* and *cis* rotaxane. (CoGEF, DFT B3LYP/6-31G, vac). (a) Tensile deformation mapped on E_{max} structures of $\mathbf{1}_{trans\text{-OMe}}'$ (i), $\mathbf{1}_{flip\text{-OMe}}'$ (ii), $\mathbf{1}_{cis\text{-OMe}}'$ (iii) and $\mathbf{1}_{cis\text{-OEt}}'$ (iv). Anchor atoms are indicated by the pink disks. Hydrogen atoms omitted for clarity. (b) Angular deformation of the axle with the evolution of angles *β*, *γ*, *δ* and *ζ* in models $\mathbf{1}_{trans\text{-OMe}}'$ (i), $\mathbf{1}_{flip\text{-OMe}}'$ (ii), $\mathbf{1}_{cis\text{-OMe}}'$ (iii) and $\mathbf{1}_{cis\text{-OEt}}'$ (iv) upon simulated elongation. Angle labels in *cis* rotaxane are same as that in *trans*.

7.9 Comparison of Activation Energies upon External Force of Model $\mathbf{1}_{cis\text{-}OMe}$', $\mathbf{1}_{cis\text{-}OEt}$', $\mathbf{1}_{flip\text{-}OMe}$' and $\mathbf{1}_{trans\text{-}OMe}$'

We performed geometry optimisations using the External Force is Explicitly Included method (EFEI),[9] where external force (from 0 to 5 nN, in 250 pN increments) was applied to the anchor atoms indicated by pink disks in **Figure S39** for each rotaxane geometry. All geometry optimizations and calculations were performed at the GFN2-xTB level of theory. This EFEI method enabled us to detect the largest difference in energy (ΔE) between $\mathbf{1}_{flip\text{-}OMe}$', $\mathbf{1}_{trans\text{-}OMe}$', $\mathbf{1}_{cis\text{-}OMe}$', and $\mathbf{1}_{cis\text{-}OEt}$' at 3.75 nN. Rotaxane $\mathbf{1}_{flip\text{-}OMe}$' were found to be more energetically stabilized which could give an idea on the convenience of the flipping in terms of conformational stability.

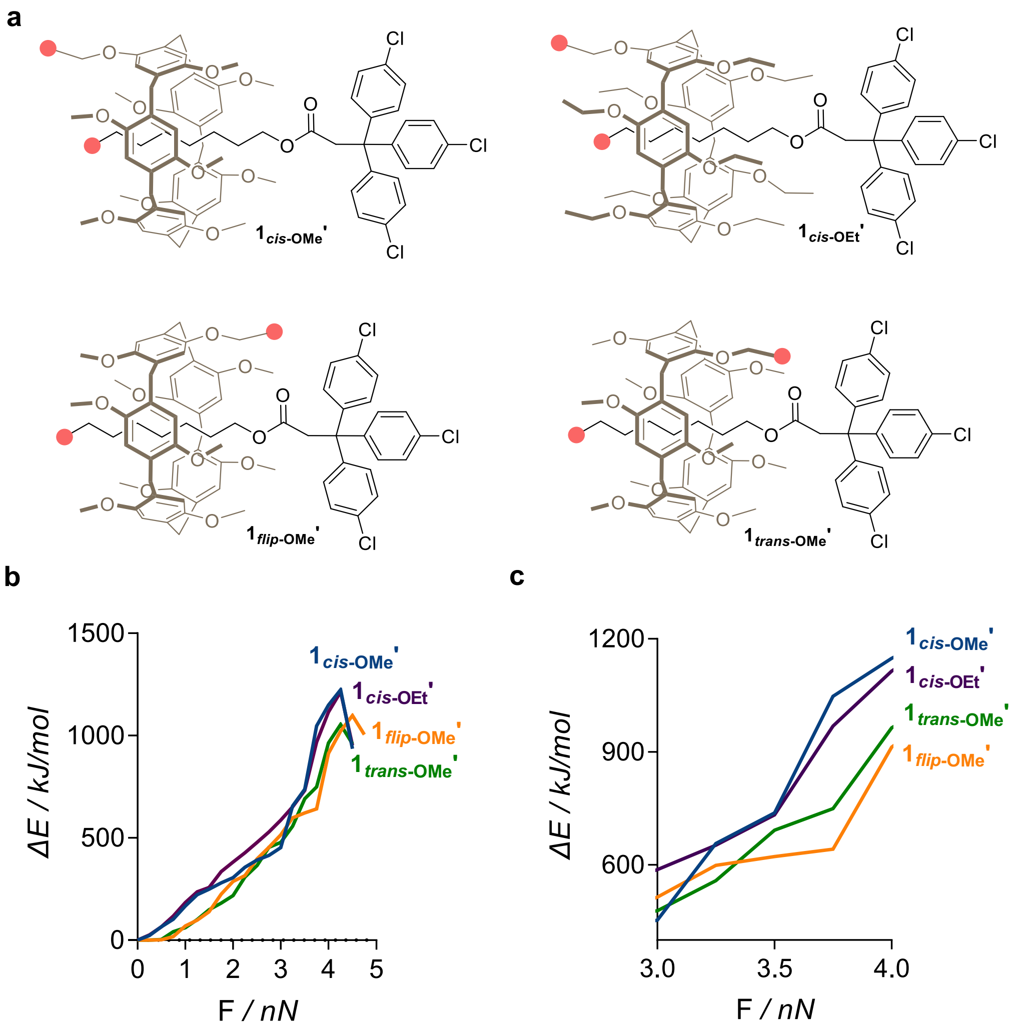

Figure S39. (a) Modelled structures $\mathbf{1}_{cis\text{-}OMe}$', $\mathbf{1}_{cis\text{-}OEt}$', $\mathbf{1}_{flip\text{-}OMe}$' and $\mathbf{1}_{trans\text{-}OMe}$'. (b) Comparison of the EFEI energy landscapes upon external force (GFN2-xTB) between $\mathbf{1}_{cis\text{-}OMe}$' (blue), $\mathbf{1}_{cis\text{-}OEt}$' (purple), $\mathbf{1}_{flip\text{-}OMe}$' (orange), and $\mathbf{1}_{trans\text{-}OMe}$' (green). (c) Zoom-in of panel (b) in the 3.0-4.0 nN range.

7.10 Molecular Dynamics Simulations

Molecular dynamics simulations (20 trajectories) starting from randomized velocities at 3.75 nN for model rotaxanes $\mathbf{1}_{cis\text{-}OMe}$', $\mathbf{1}_{cis\text{-}OEt}$', $\mathbf{1}_{flip\text{-}OMe}$' and $\mathbf{1}_{trans\text{-}OMe}$', were performed. From these calculations we extracted the evolution of the free energy and of the dihedral angle ω (depicted in Sections 7.2-6).

7.10.1 Molecular Dynamics Simulations of Model $\mathbf{1}_{\mathit{cis}\text{-}\mathbf{OMe}}'$

Out of 20 molecular dynamics trajectories of model $\mathbf{1}_{\mathit{cis}\text{-}\mathbf{OMe}}'$ at 3.75 nN, flipping was observed in 50% of the trajectories (1, 2, 6, 9, 10, 11, 13, 15, 16, and 19). All the other trajectories lead to unstoppering without flipping. Additionally, trajectories 1, 2, 6, 10, 13, and 15 lead to unstoppering after flipping.

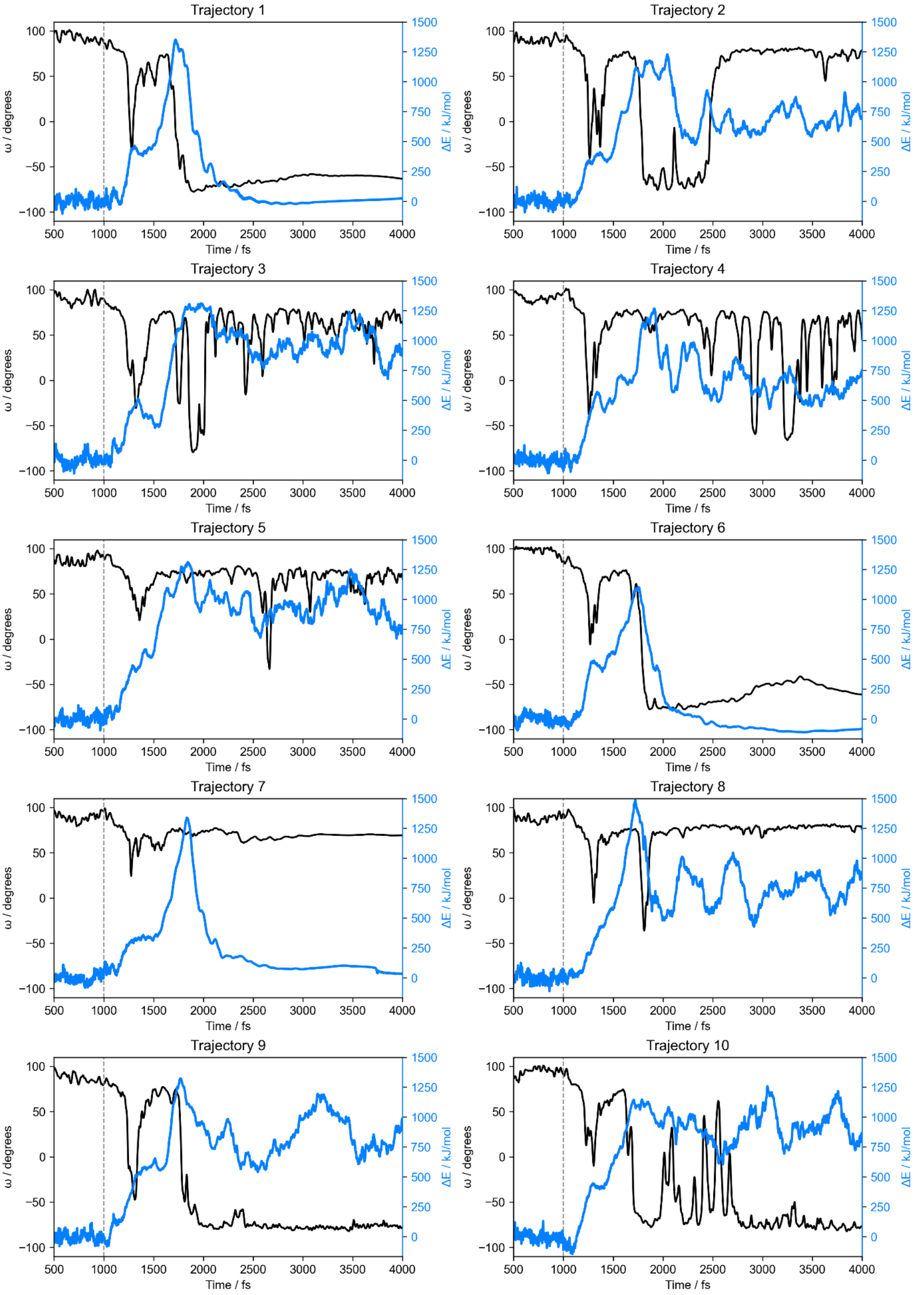

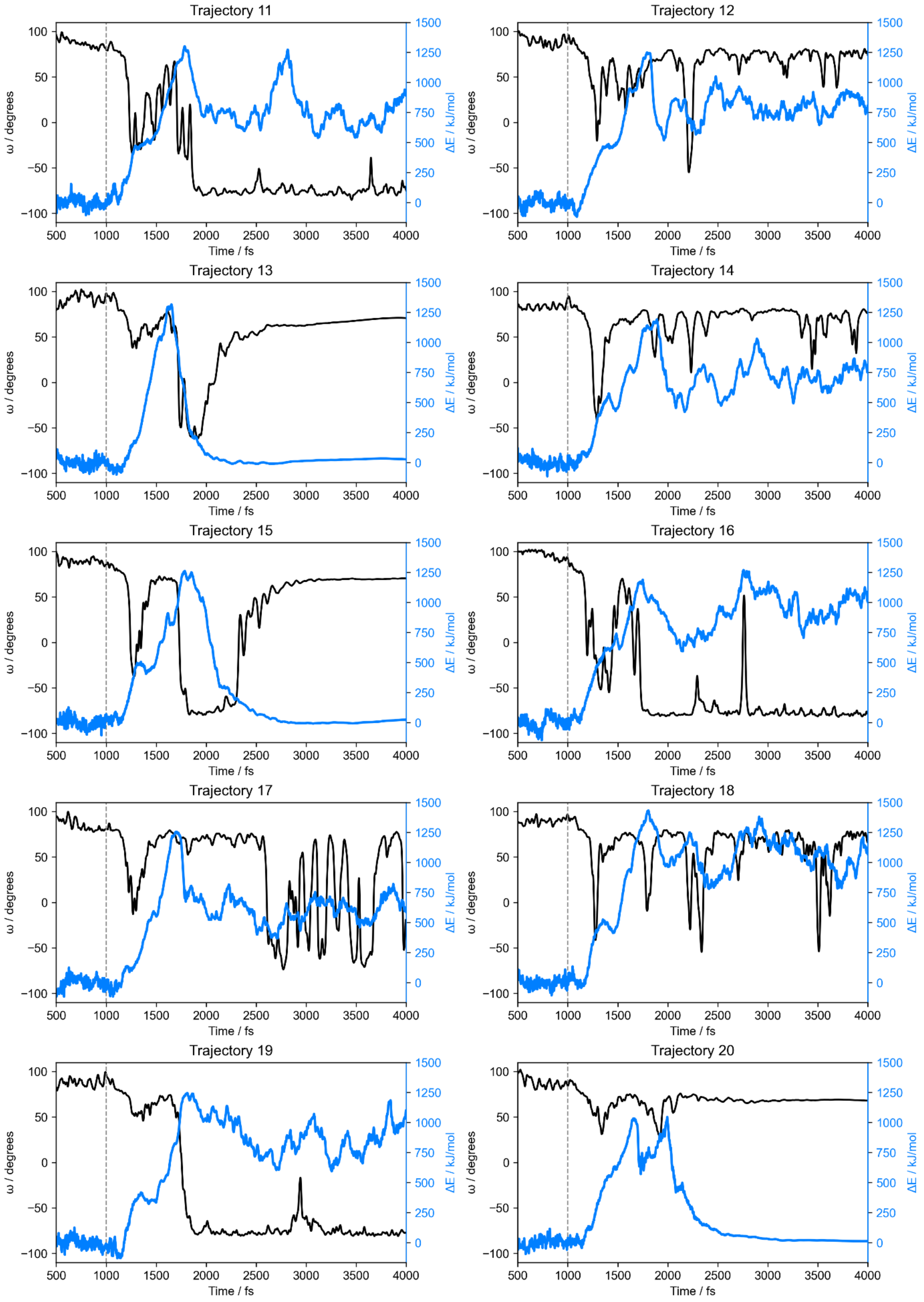

Figure S40. Evolution of dihedral angle *ω* (black trace) and free energy (blue trace) over time for each trajectory of model rotaxane $\mathbf{1}_{\textit{cis}\text{-OMe}}$' at a constant force of 3.75 nN. The vertical grey dashed line at 1000 fs indicates the onset of force application.

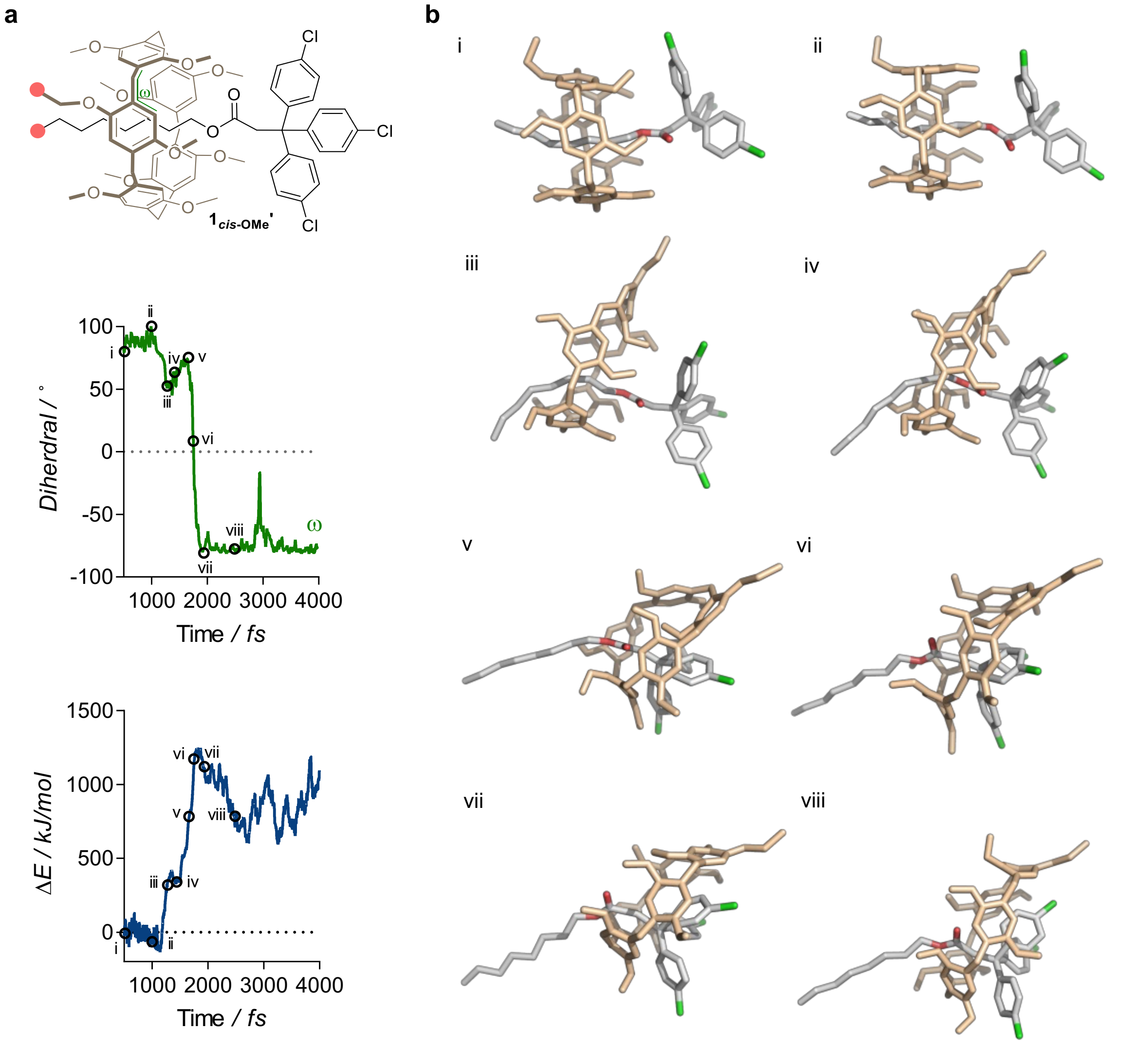

Figure S41. Computational investigation of mechanically triggered local flipping in mechanophore $\mathbf{1}_{cis\text{-}OMe}'$. (GFN2-xTB@3.75 nN, vac). (a) Structure used in the computation showing dihedral angle ω. Anchor atoms are indicated by the pink disks. Evolution of dihedral angle ω (green trace) and free energy (dark blue trace) over time at a constant force of 3.75 nN of a representative trajectory (19 in **Figure S40**). (b) Side views of representative structures along the trajectory showing the flipping of the hydroquinone group via an oxygen-through-the-annulus force-induced rotation. Hydrogen atoms omitted for clarity.

7.10.2 Molecular Dynamics Simulations of Model $\mathbf{1}_{cis\text{-OEt}}$’

All 20 molecular dynamics trajectories of model $\mathbf{1}_{cis\text{-OEt}}$’ at 3.75 nN lead to unstoppering without flipping.

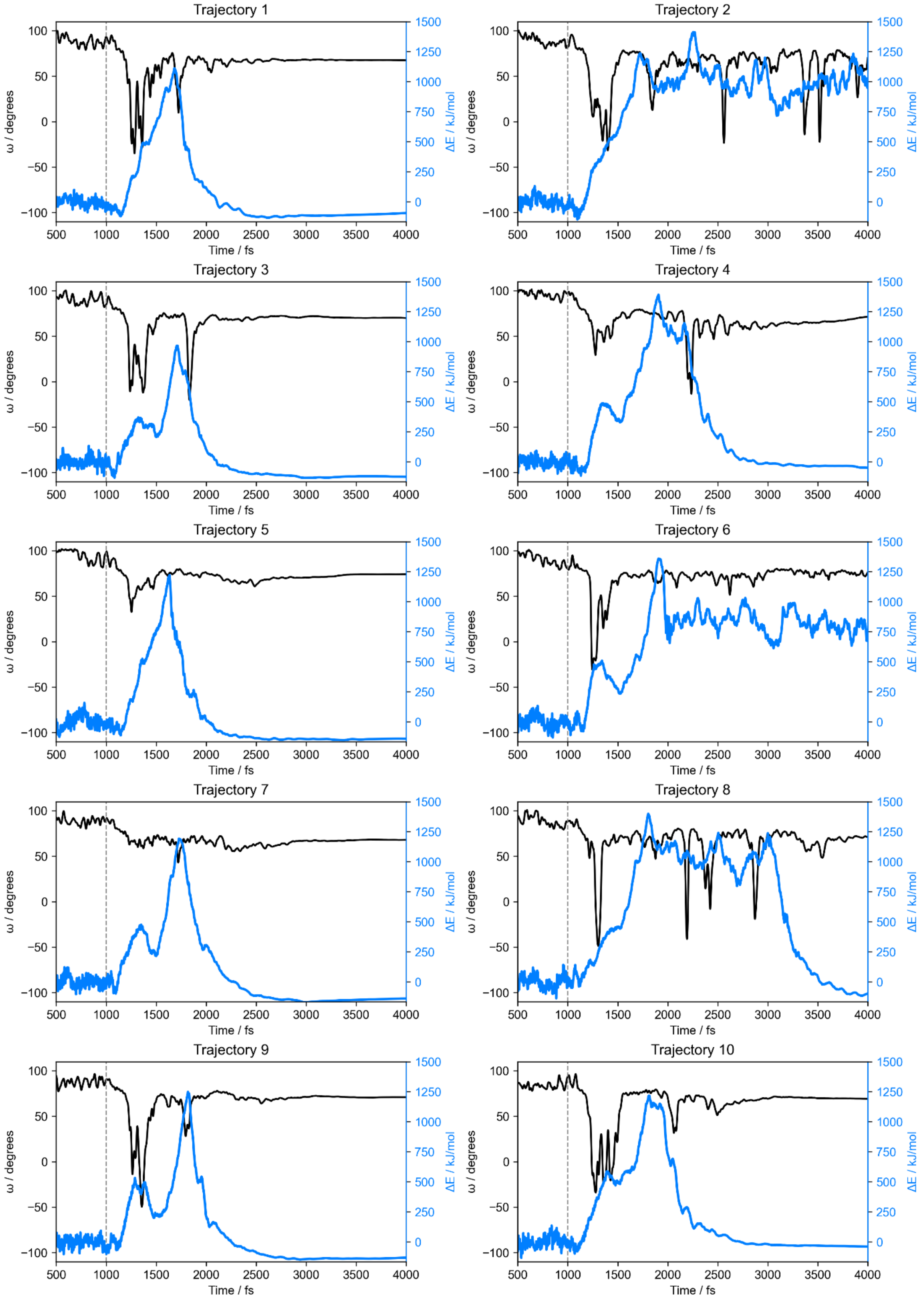

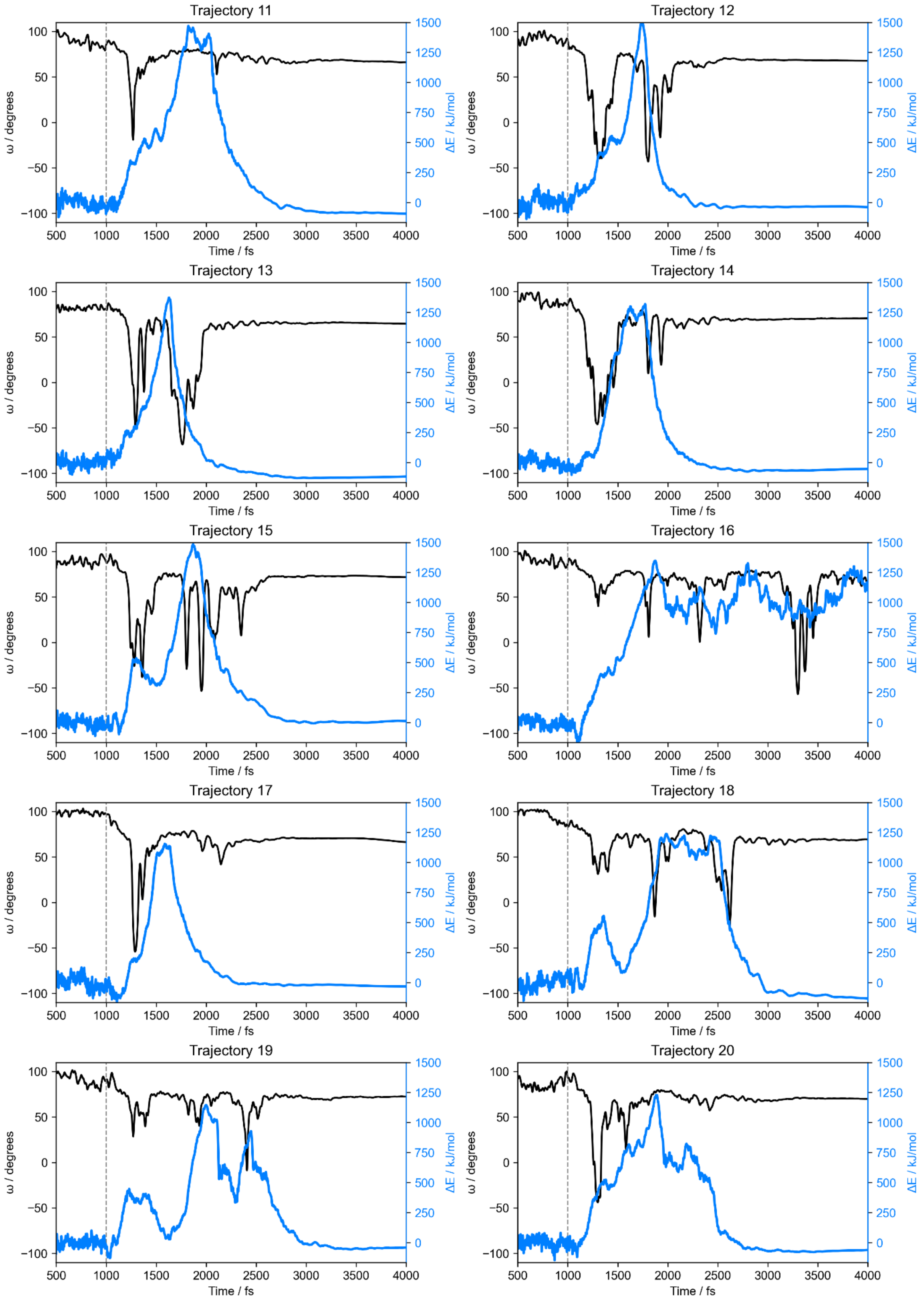

Figure S42. Evolution of dihedral angle *ω* (black trace) and free energy (blue trace) over time for each trajectory of model rotaxane **1***cis*-OEt**'** at a constant force of 3.75 nN. The vertical grey dashed line at 1000 fs indicates the onset of force application.

7.10.3 Molecular Dynamics Simulations of Model $\mathbf{1}_{\textit{flip}\text{-OMe}}$'

All 10 molecular dynamics trajectories of model $\mathbf{1}_{\textit{flip}\text{-OMe}}$' at 3.75 nN lead to unstoppering without flipping.

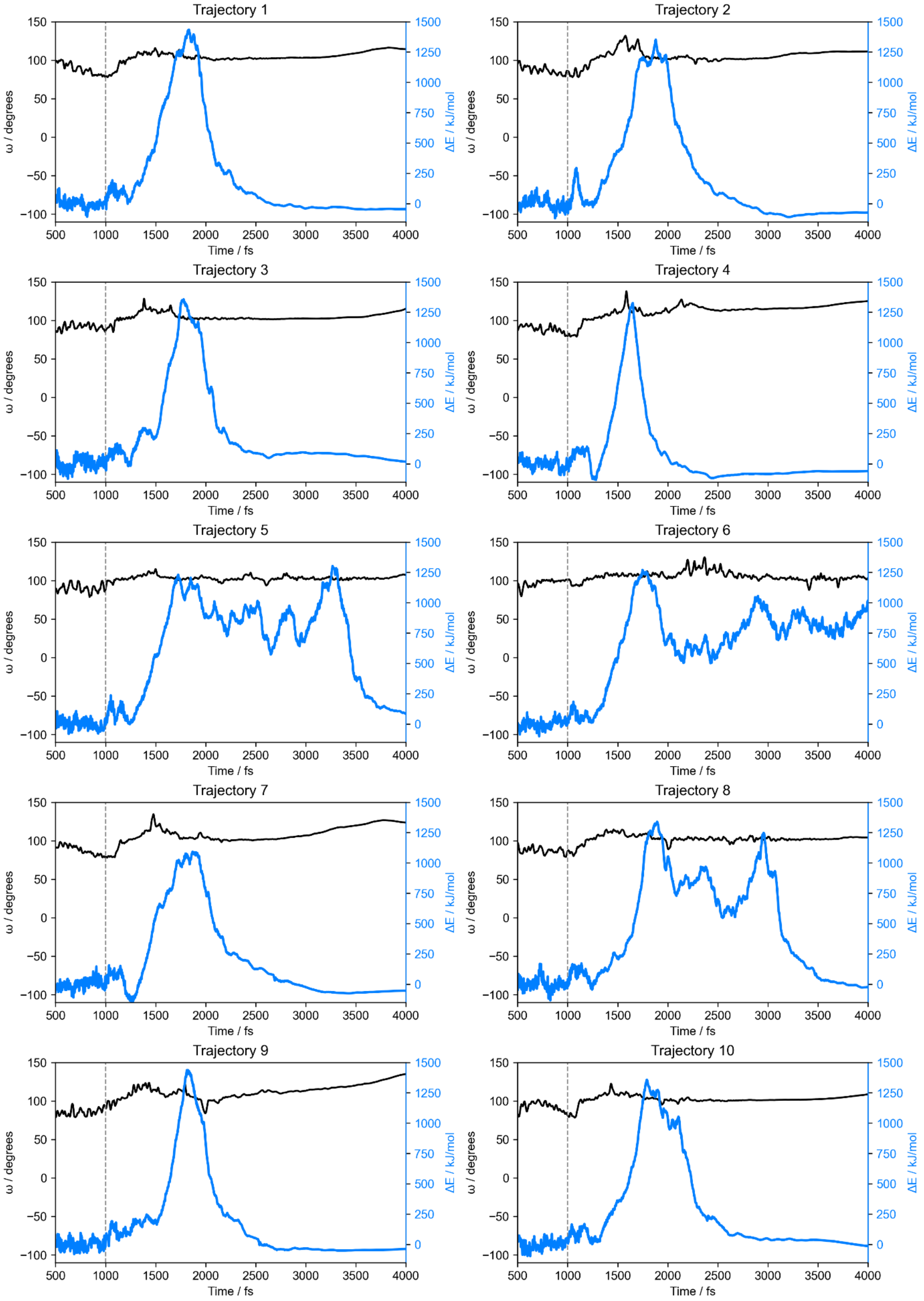

Figure S43. Evolution of dihedral angle ω (black trace) and free energy (blue trace) over time for each trajectory of model rotaxane $\mathbf{1}_{\textit{flip}\text{-OMe}}$' at a constant force of 3.75 nN. The vertical grey dashed line at 1000 fs indicates the onset of force application.

7.10.4 Molecular Dynamics Simulations of Model $\mathbf{1}_{\textit{trans}\text{-OMe}}'$

All 10 molecular dynamics trajectories of model $\mathbf{1}_{\textit{trans}\text{-OMe}}'$ at 3.75 nN lead to unstoppering without flipping.

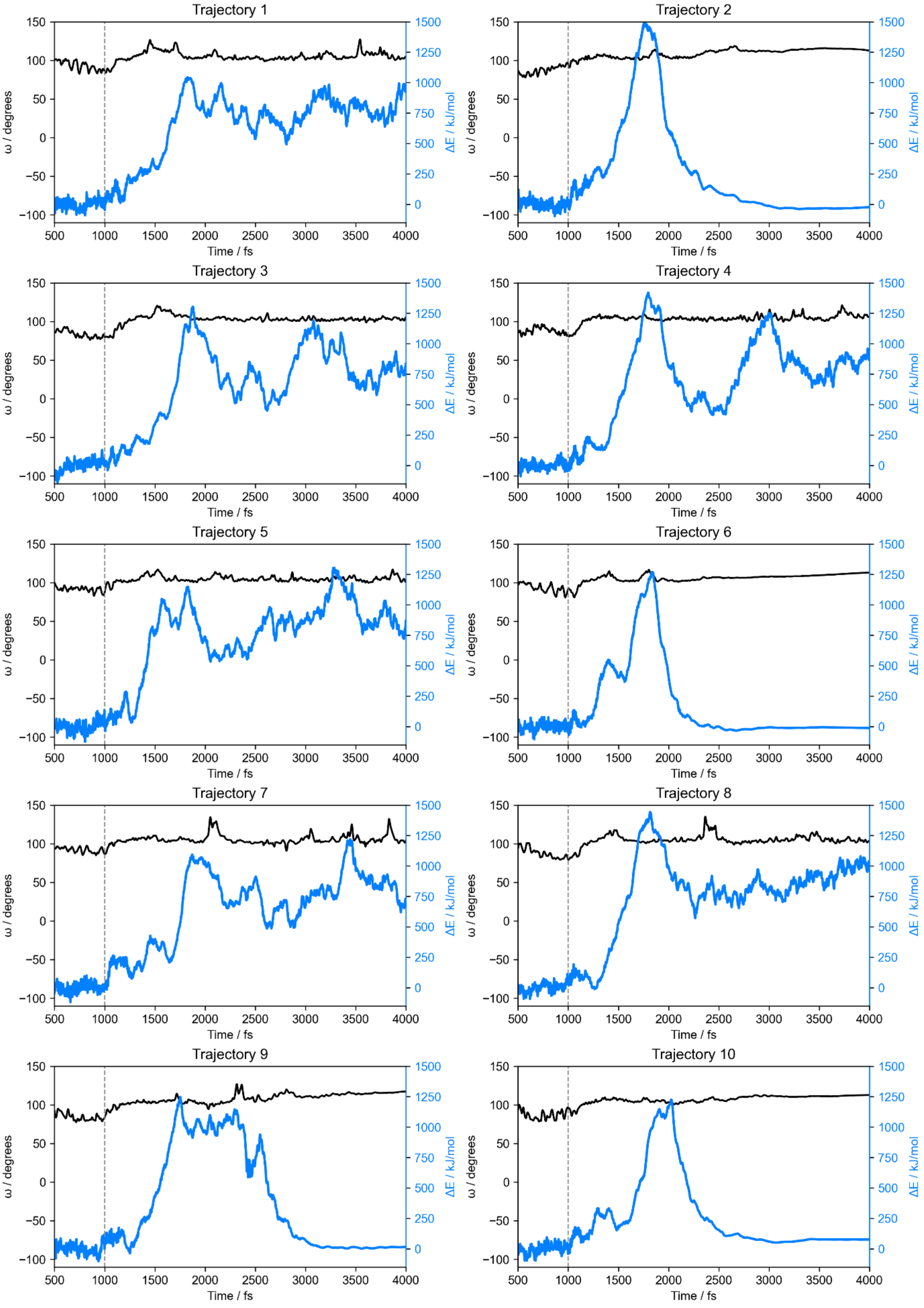

Figure S44. Evolution of dihedral angle ω (black trace) and free energy (blue trace) over time for each trajectory of model rotaxane $\mathbf{1}_{\textit{trans}\text{-OMe}}'$ at a constant force of 3.75 nN. The vertical grey dashed line at 1000 fs indicates the onset of force application.

8 NMR Spectra

8.1 Small Molecule NMR Spectra

8.1.1 Spectra of S2

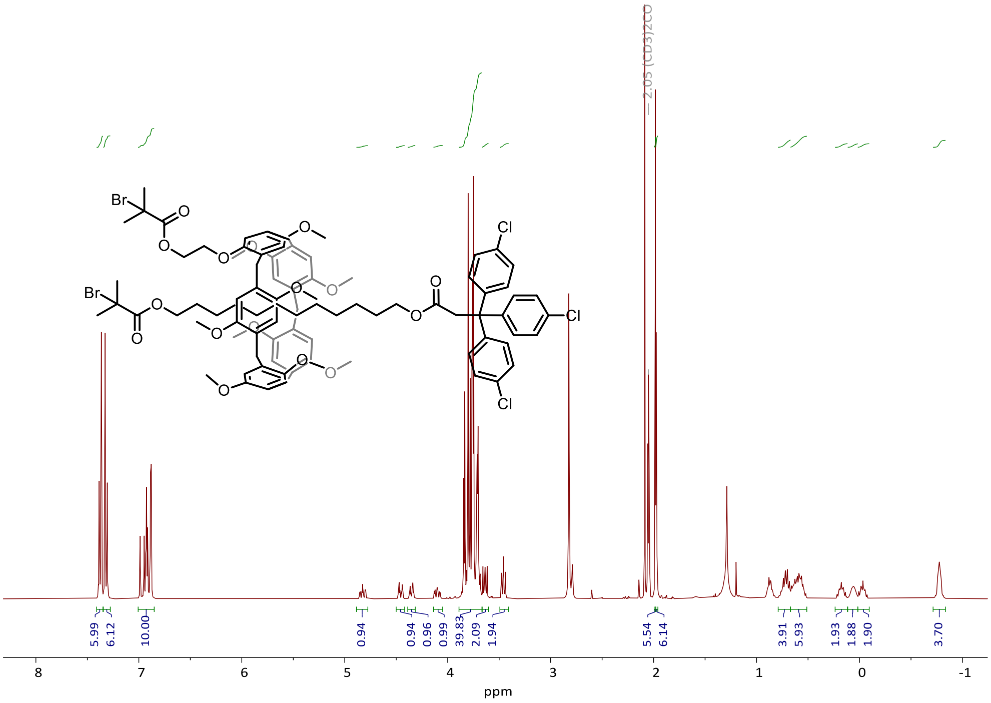

Spectrum S1. ^{1}H NMR (400 MHz, Acetone-d_6, 298 K) spectrum of compound **S2**.

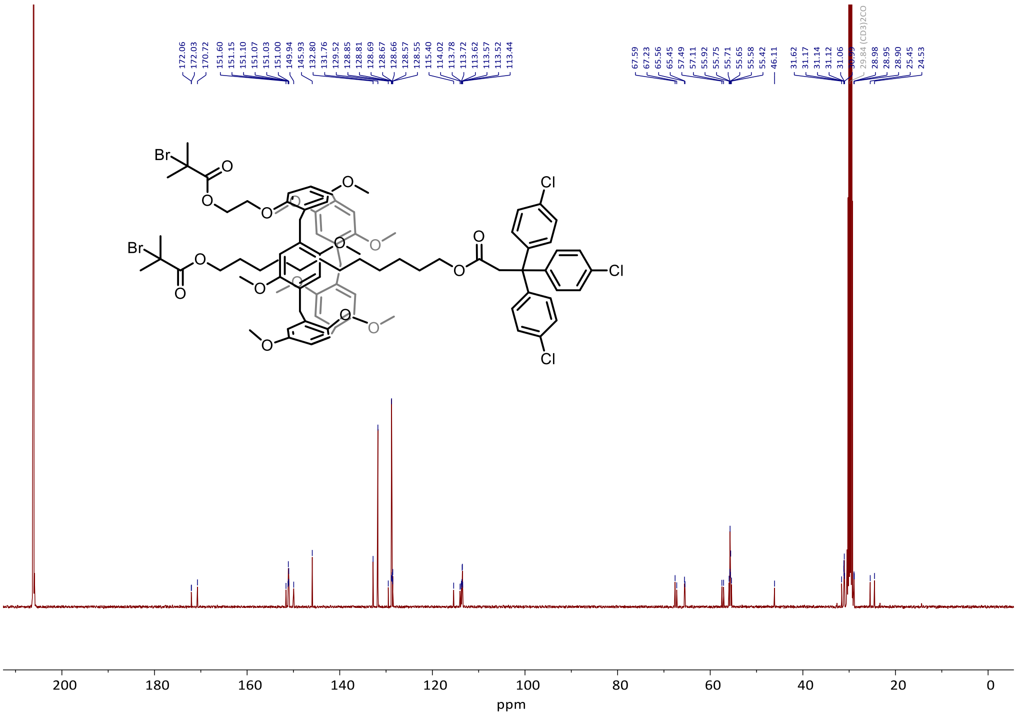

Spectrum S2. ^{13}C NMR (101 MHz, Acetone-d_6, 298 K) spectrum of compound **S2**.

8.1.2 Spectra of S4

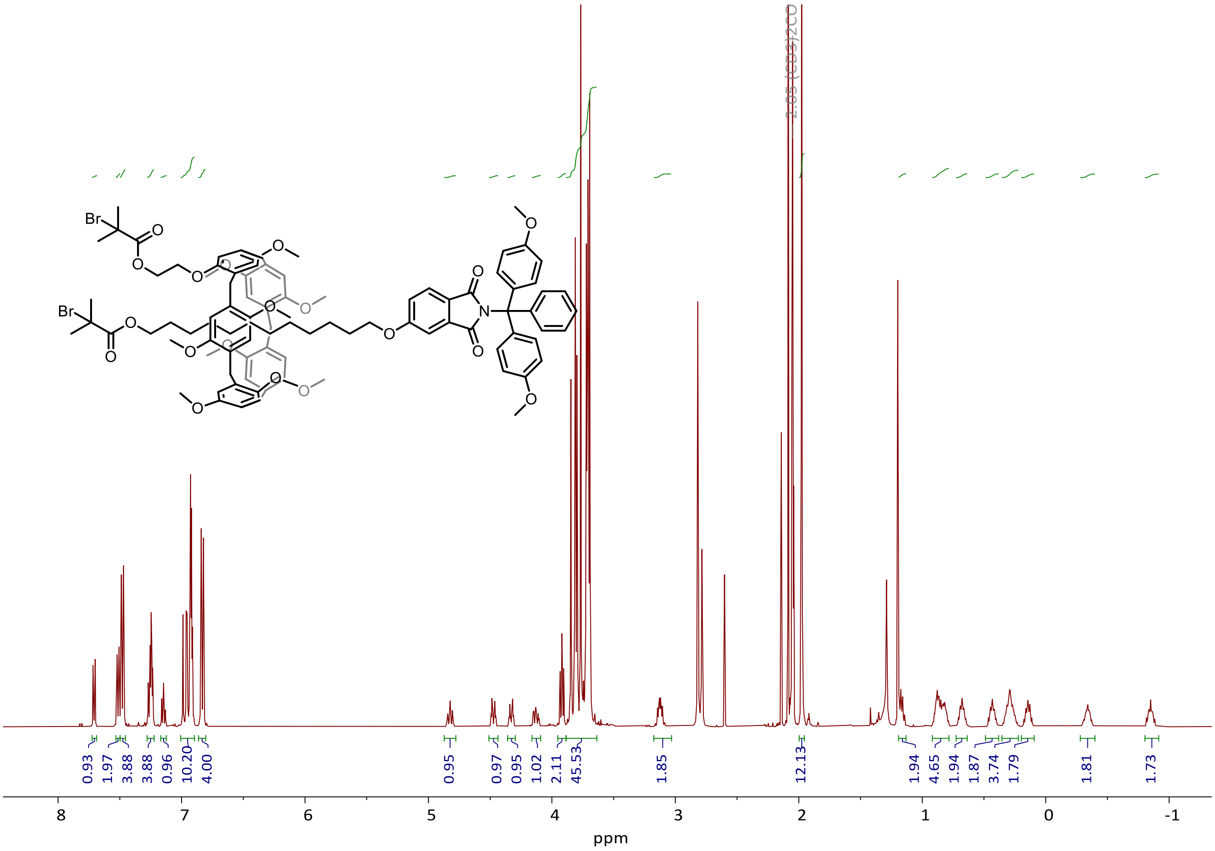

Spectrum S3. ^{1}H NMR (500 MHz, Acetone-d_6, 298 K) spectrum of compound **S4**.

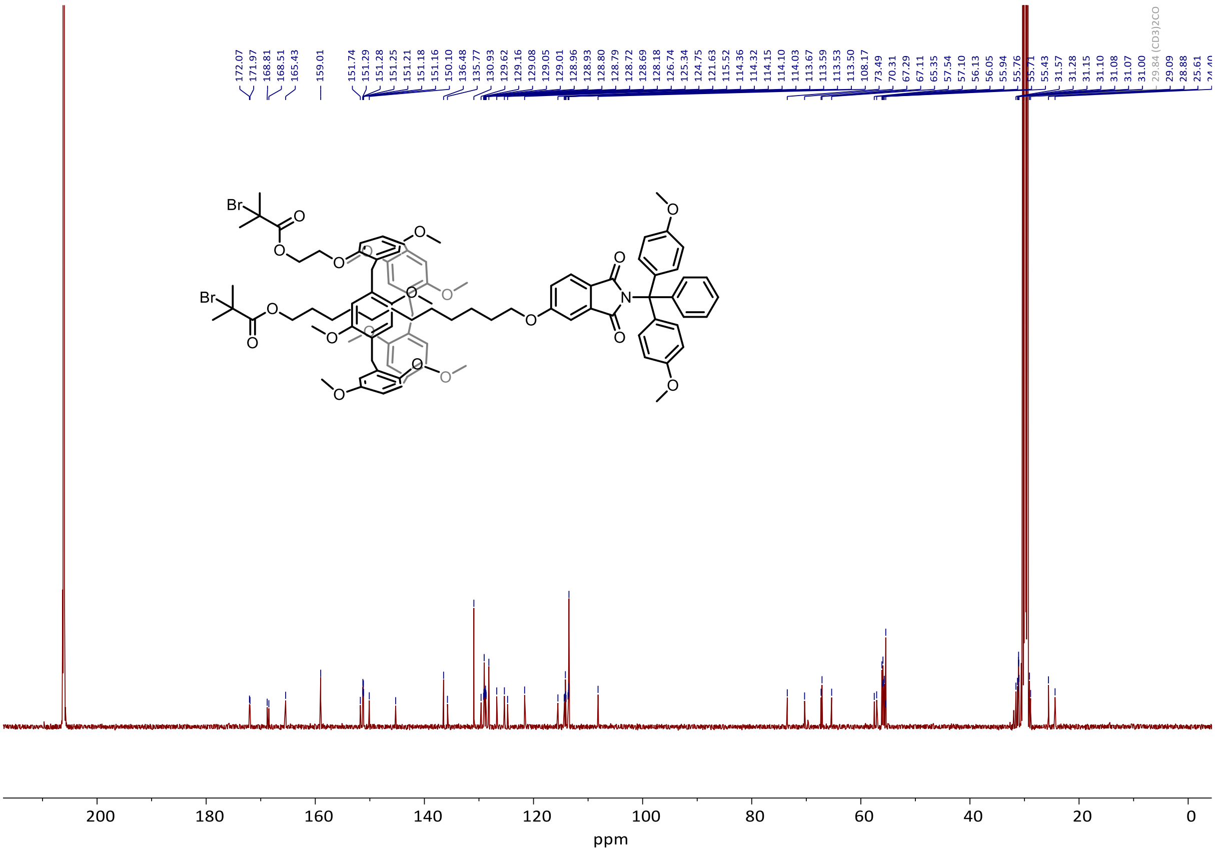

Spectrum S4. ^{13}C NMR (126 MHz, Acetone-d_6, 298 K) spectrum of compound **S4**.

8.1.3 Spectra of S6

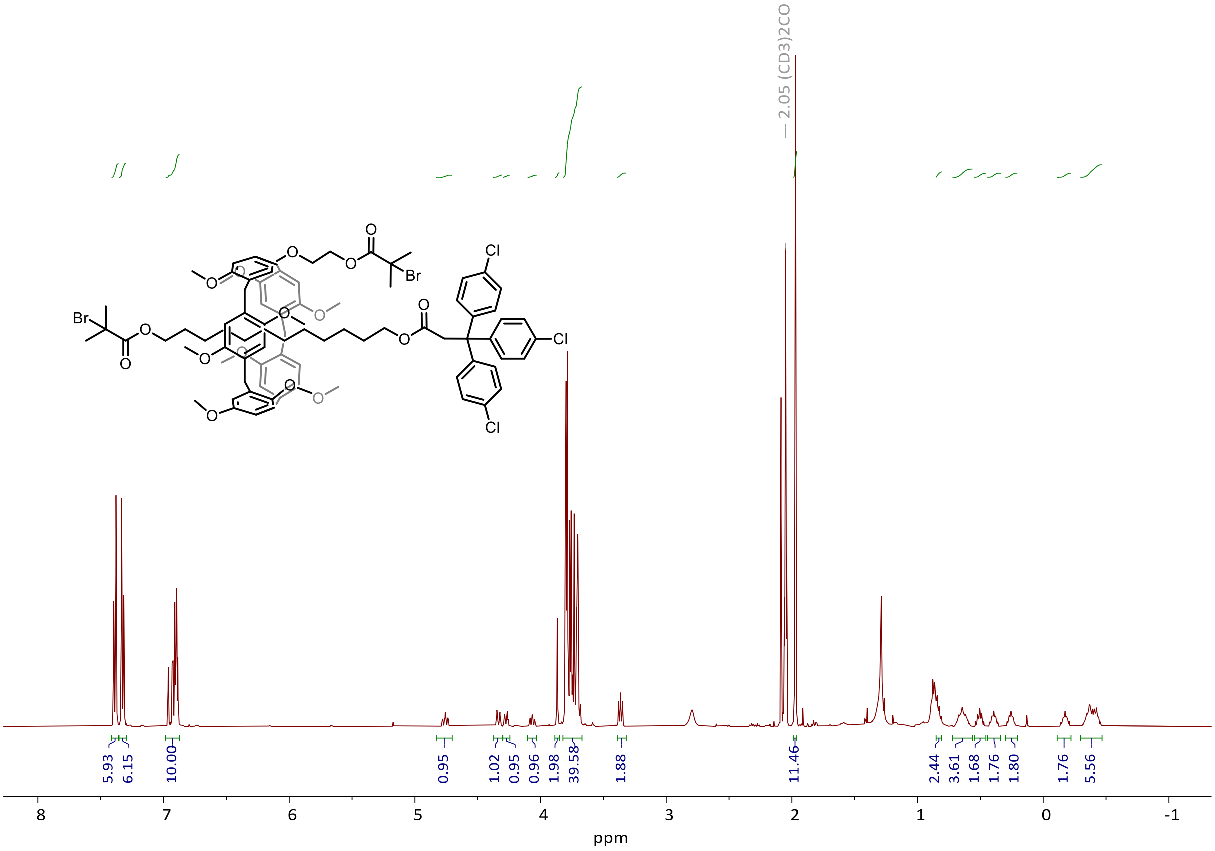

Spectrum S5. ^{1}H NMR (500 MHz, Acetone-d_6, 298 K) spectrum of compound **S6**.

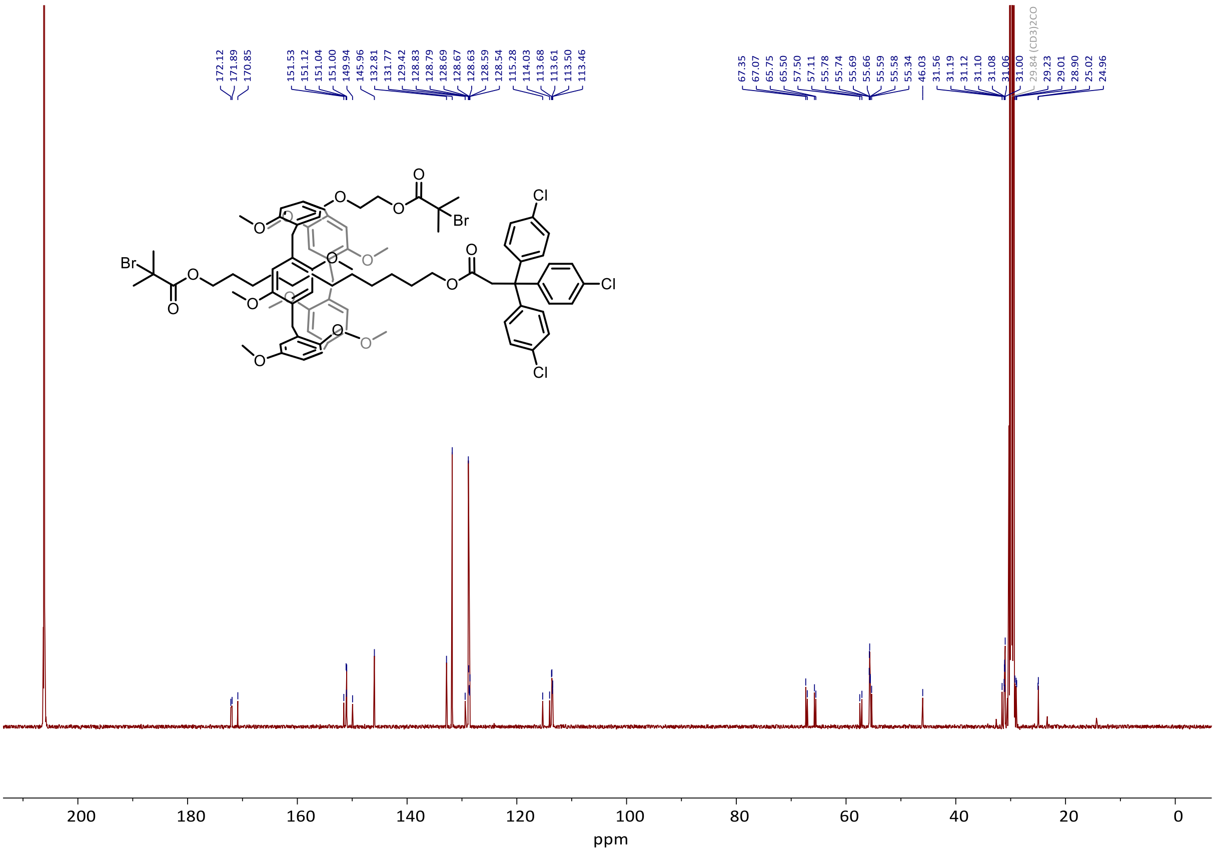

Spectrum S6. ^{13}C NMR (126 MHz, Acetone-d_6, 298 K) spectrum of compound **S6**.

8.1.4 Spectra of S16

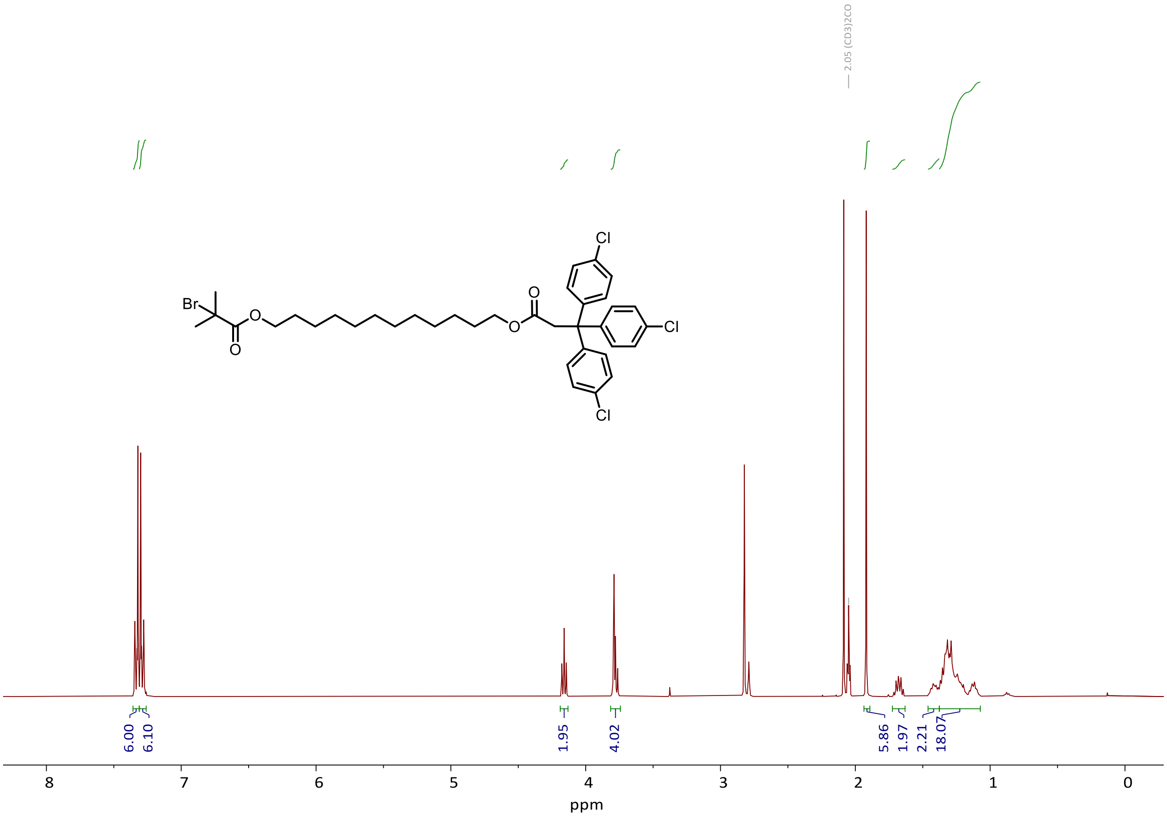

Spectrum S7. ^{1}H NMR (400 MHz, Acetone-d_6, 298 K) spectrum of compound **S16**.

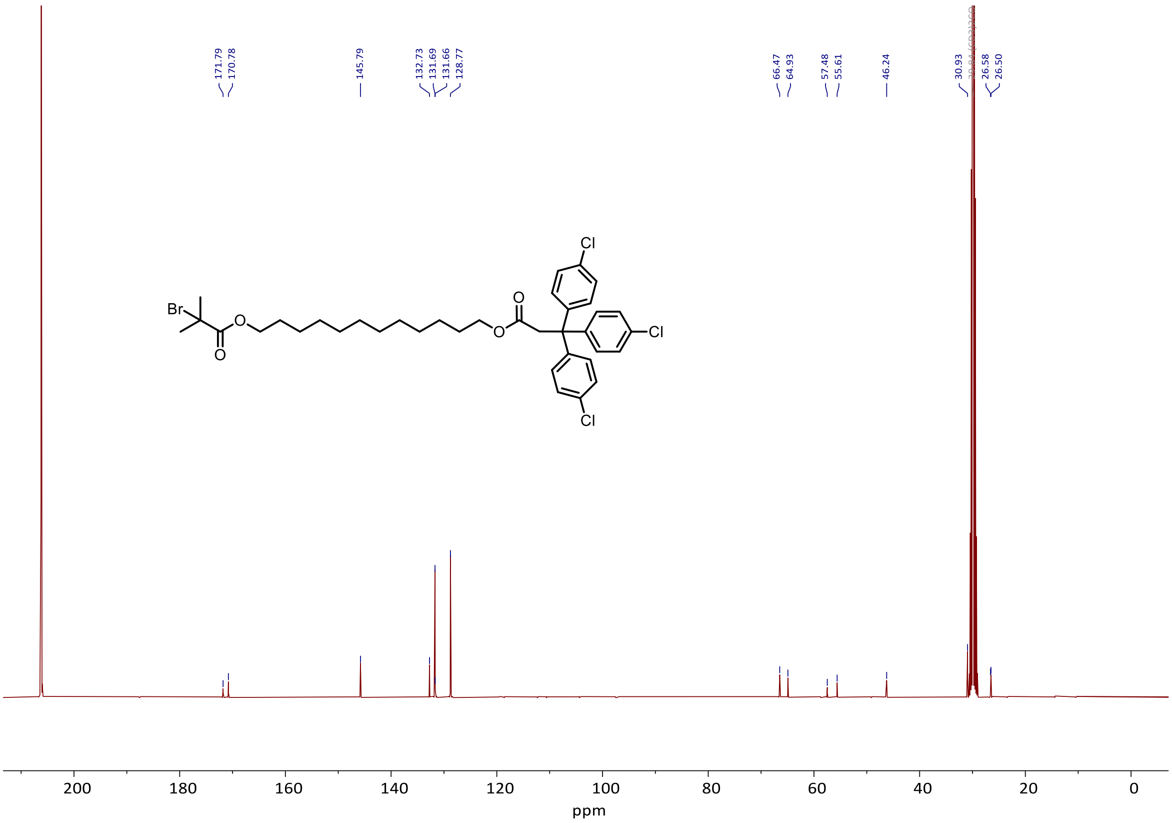

Spectrum S8. ^{13}C NMR (101 MHz, Acetone-d_6, 298 K) spectrum of compound **S16**.

8.1.5 Spectra of S8

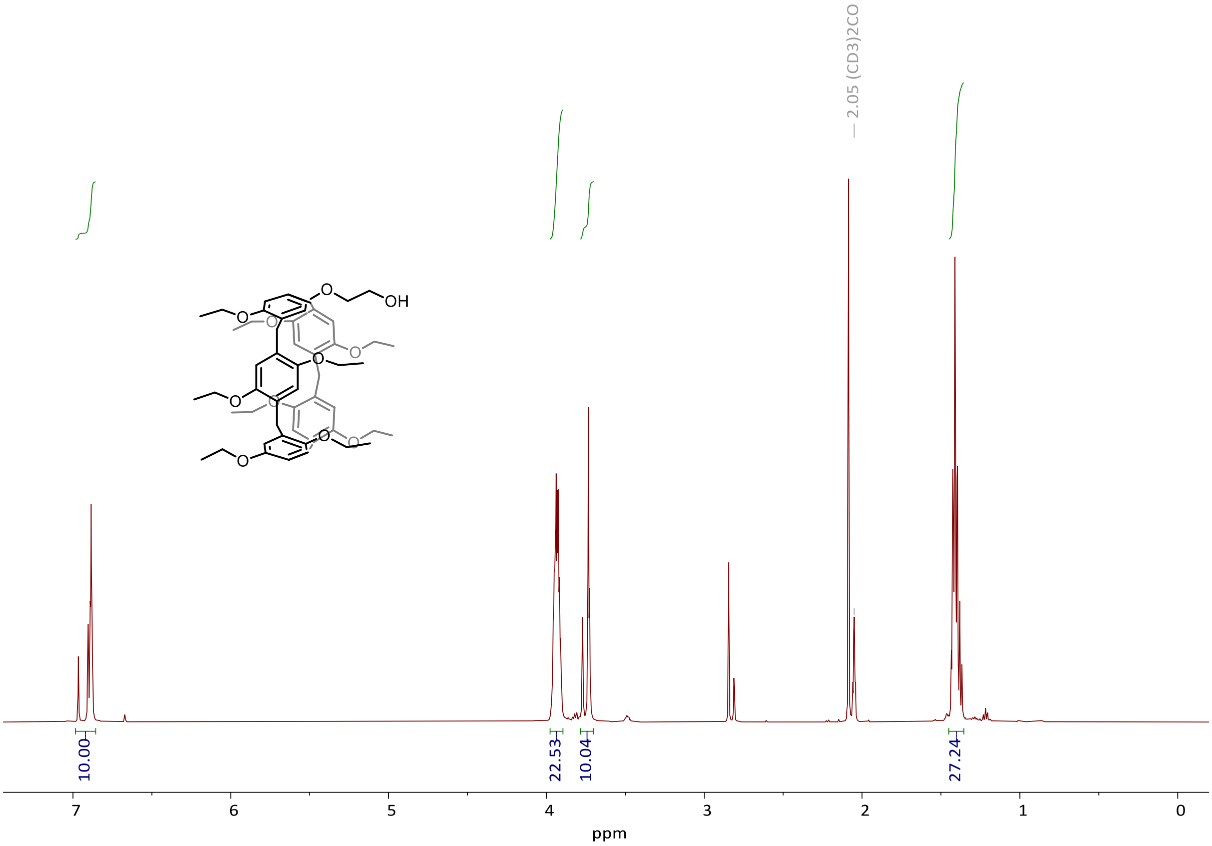

Spectrum S9. ^{1}H NMR (500 MHz, Acetone-d_6, 298 K) spectrum of compound **S8**.

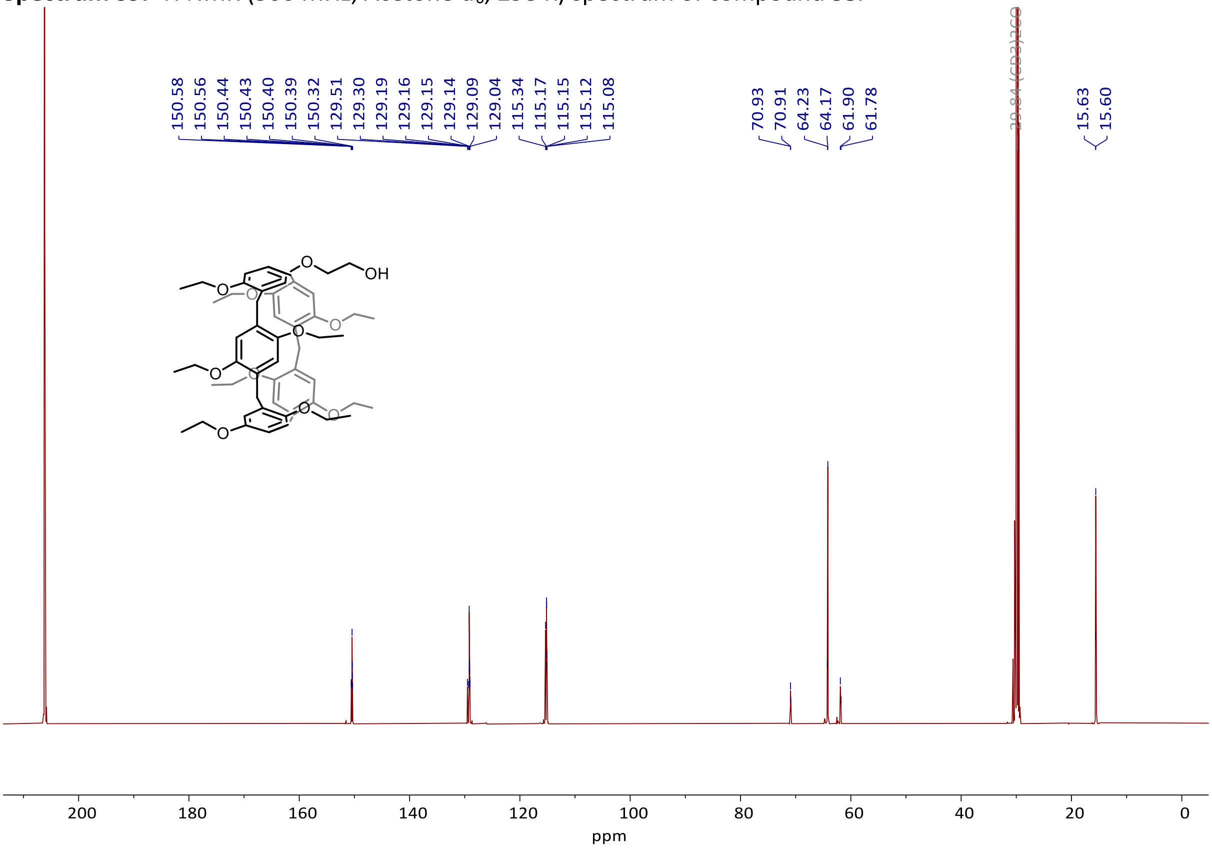

Spectrum S10. ^{13}C NMR (126 MHz, Acetone-d_6, 298 K) spectrum of compound **S8**.

8.1.6 Spectra of S9

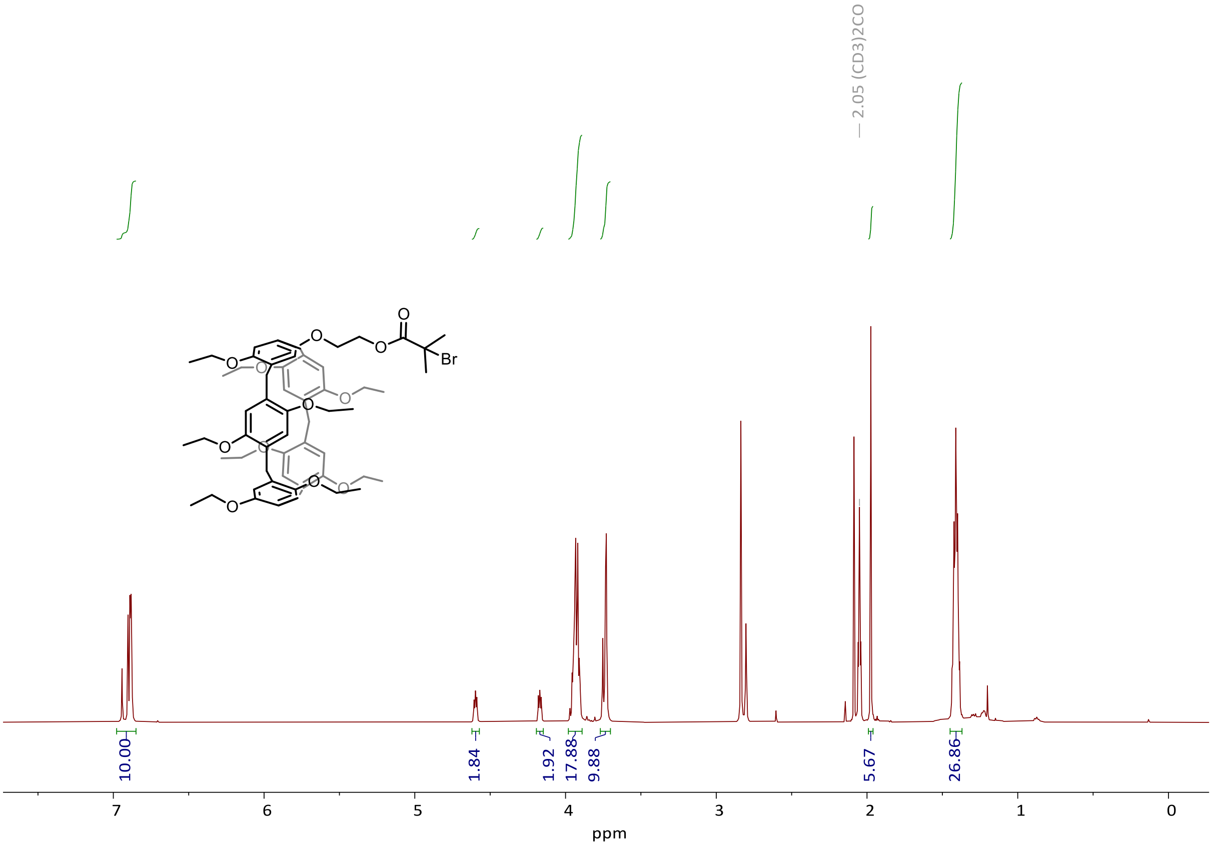

Spectrum S11. ^{1}H NMR (500 MHz, Acetone-d_6, 298 K) spectrum of compound **S9**.

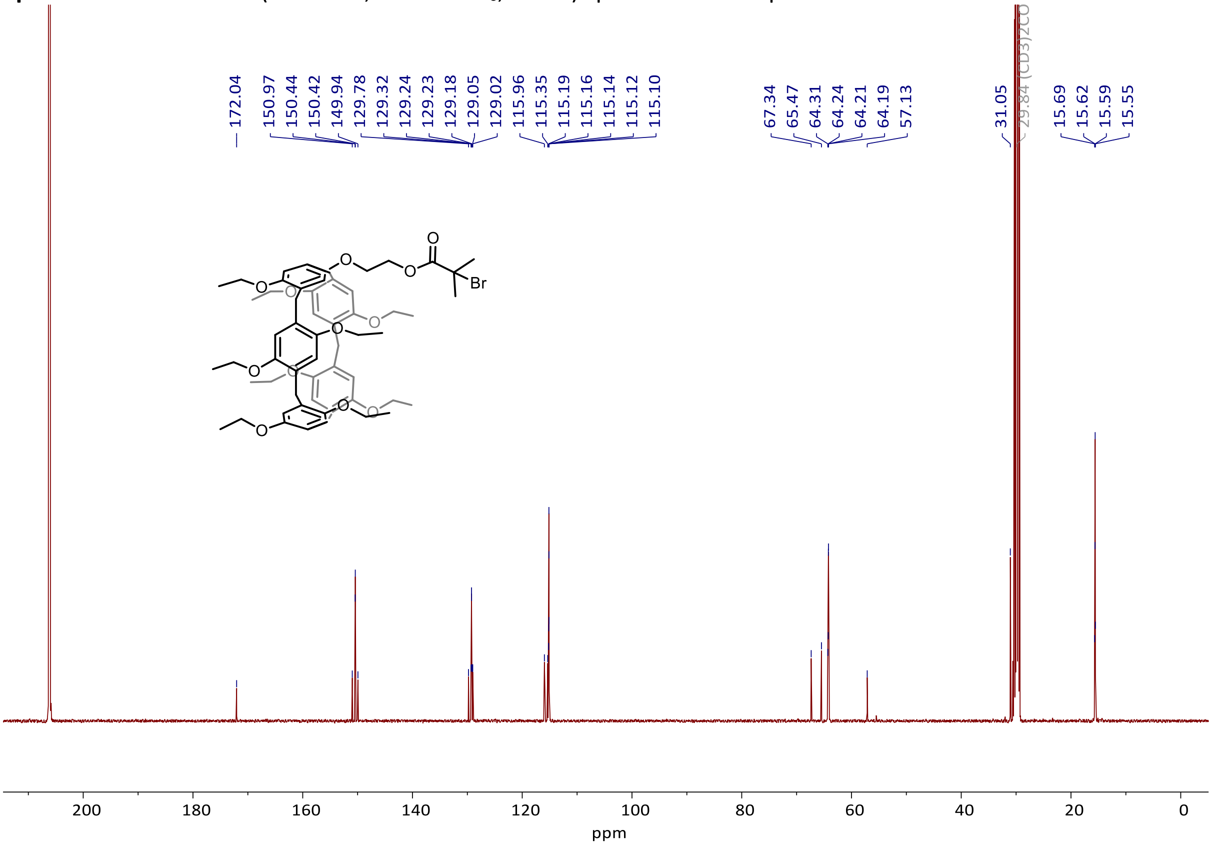

Spectrum S12. ^{13}C NMR (126 MHz, Acetone-d_6, 298 K) spectrum of compound **S9**.

8.1.7 Spectra of S11

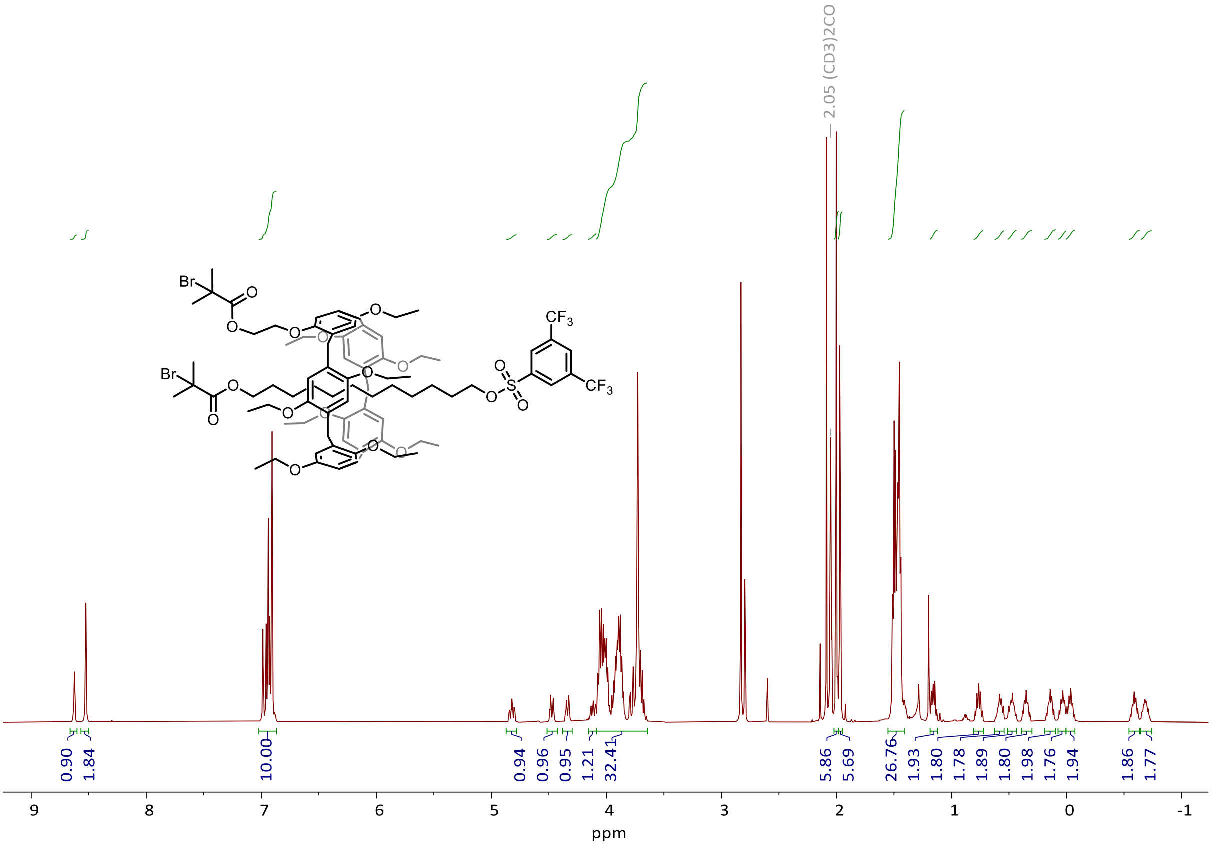

Spectrum S13. ^{1}H NMR (500 MHz, Acetone-d_6, 298 K) spectrum of compound **S11**.

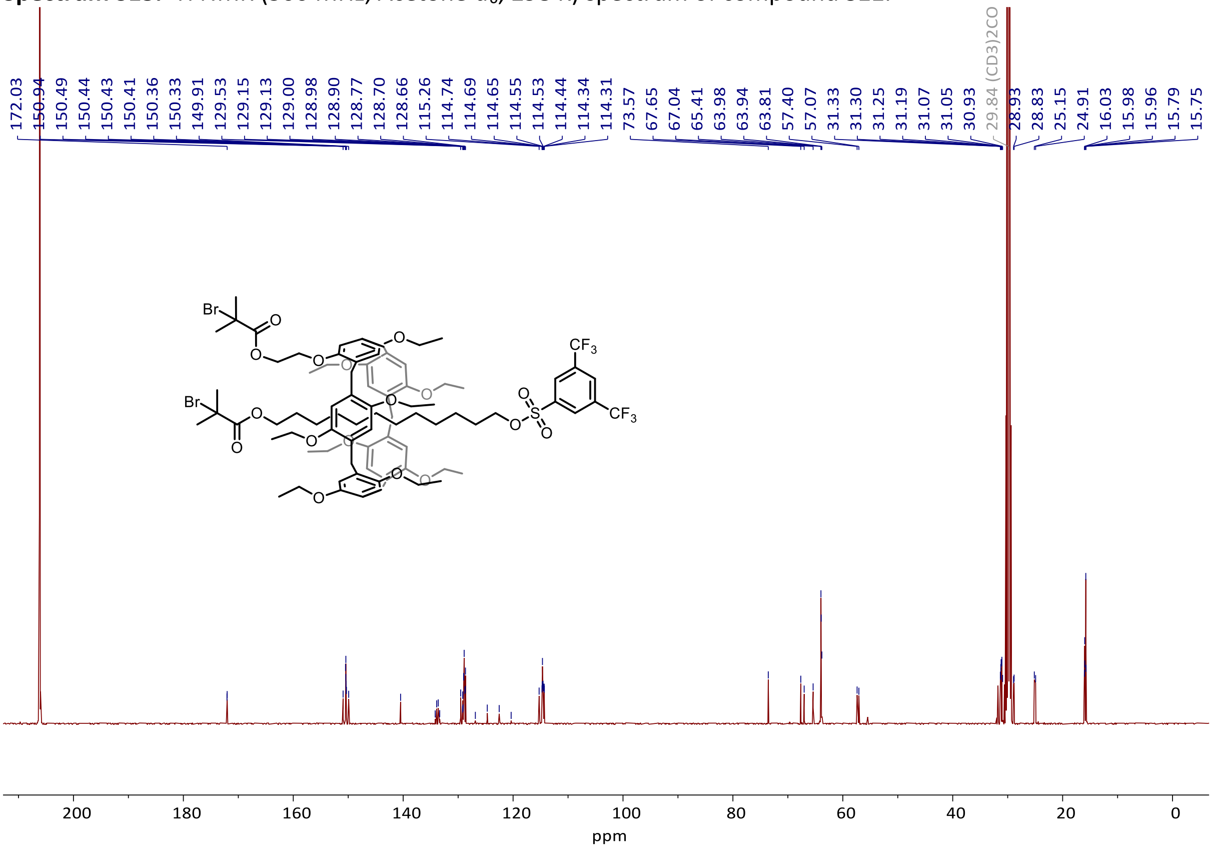

Spectrum S14. ^{13}C NMR (126 MHz, Acetone-d_6, 298 K) spectrum of compound **S11**.

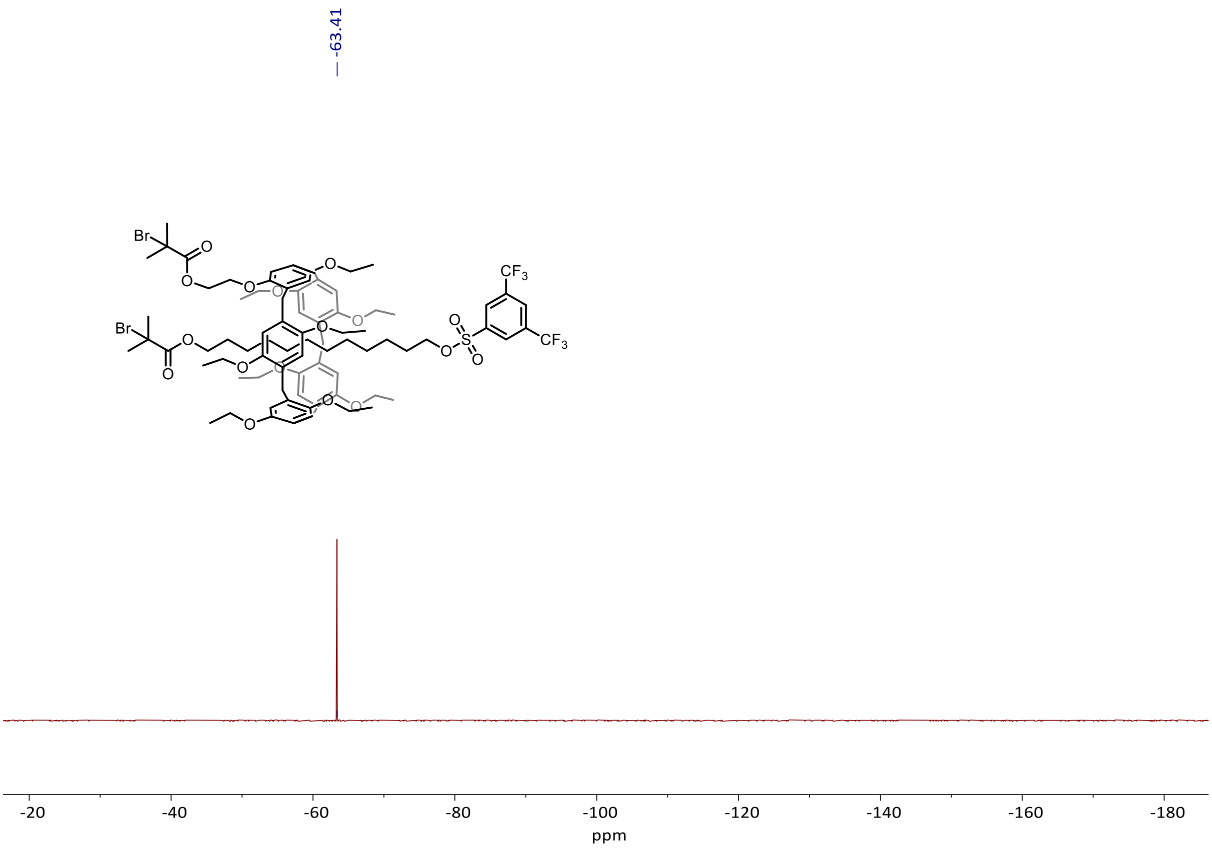

Spectrum S15. ^{19}F NMR (471 MHz, Acetone-d_6, 298 K) spectrum of compound **S11**.

8.1.8 Spectra of S12

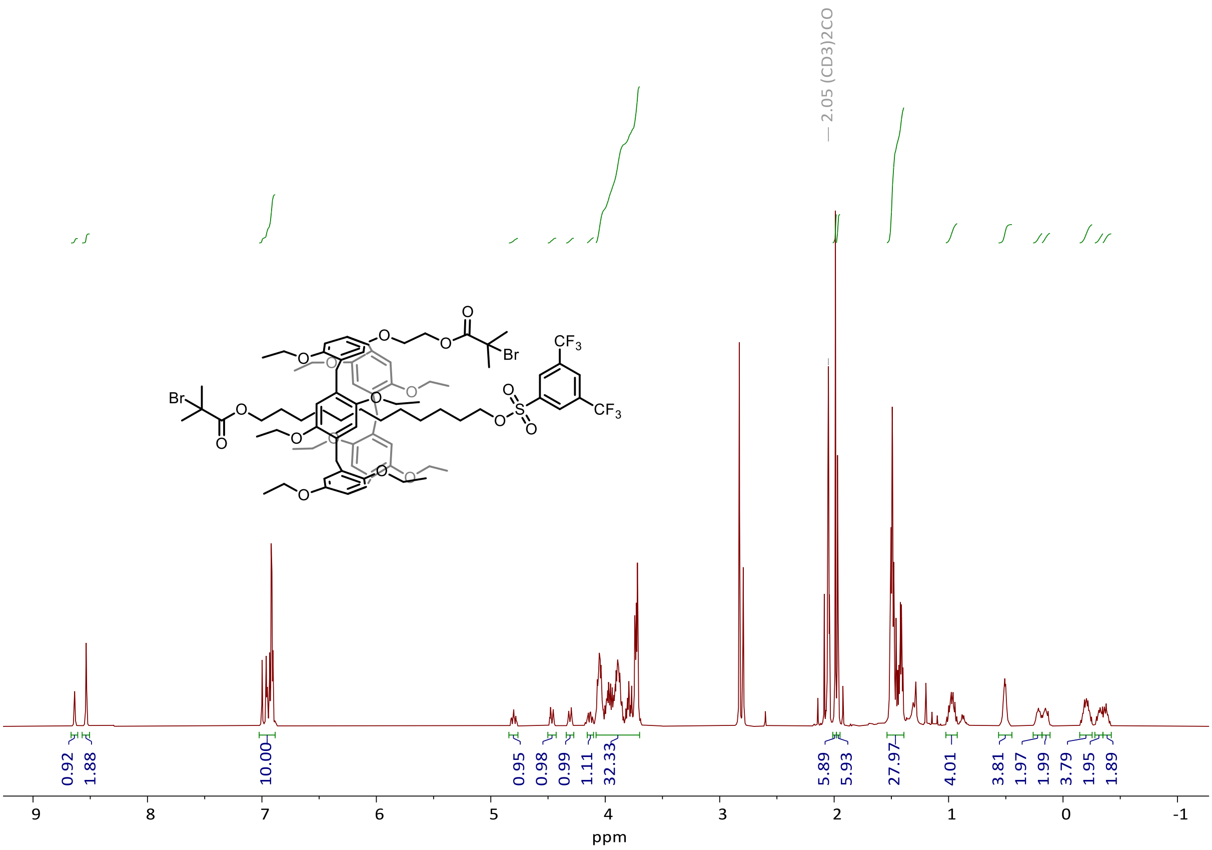

Spectrum S16. ^{1}H NMR (500 MHz, Acetone-d_6, 298 K) spectrum of compound **S12**.

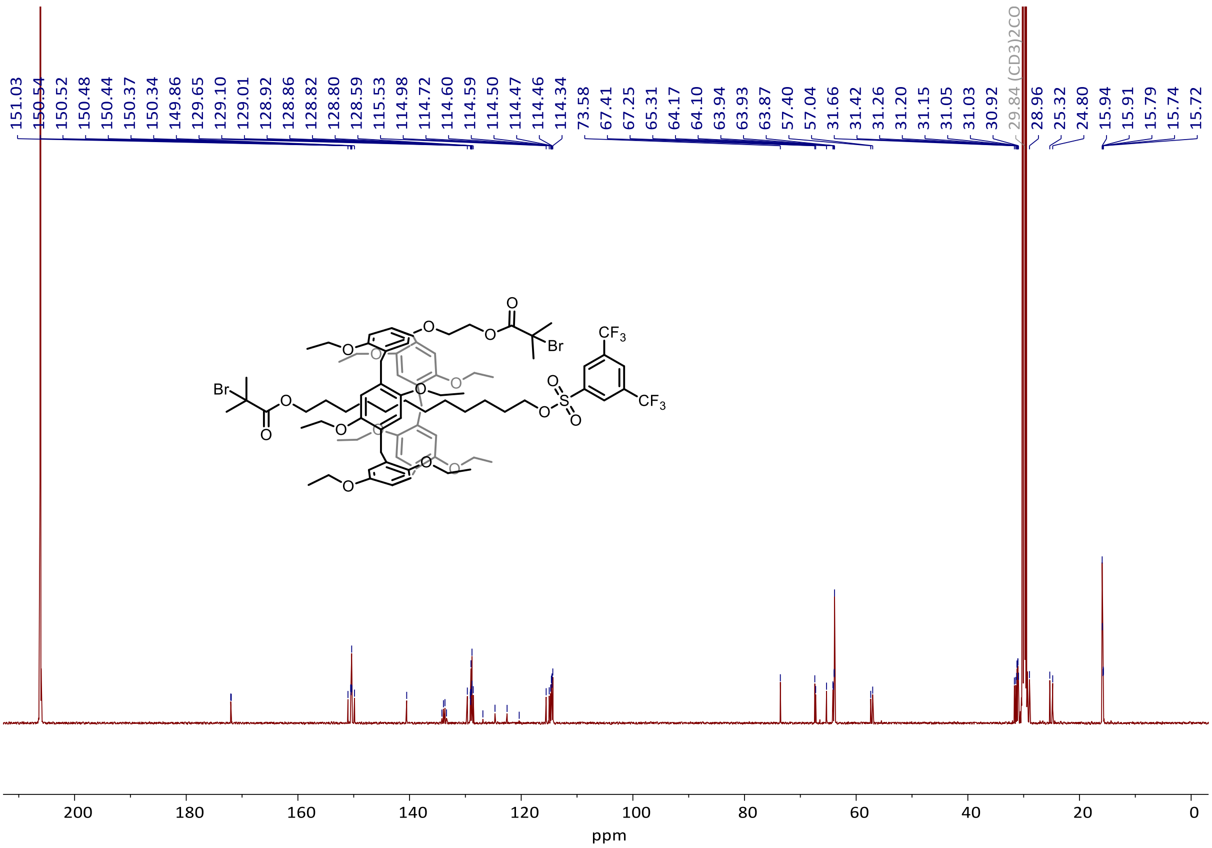

Spectrum S17. ^{13}C NMR (126 MHz, Acetone-d_6, 298 K) spectrum of compound **S12**.

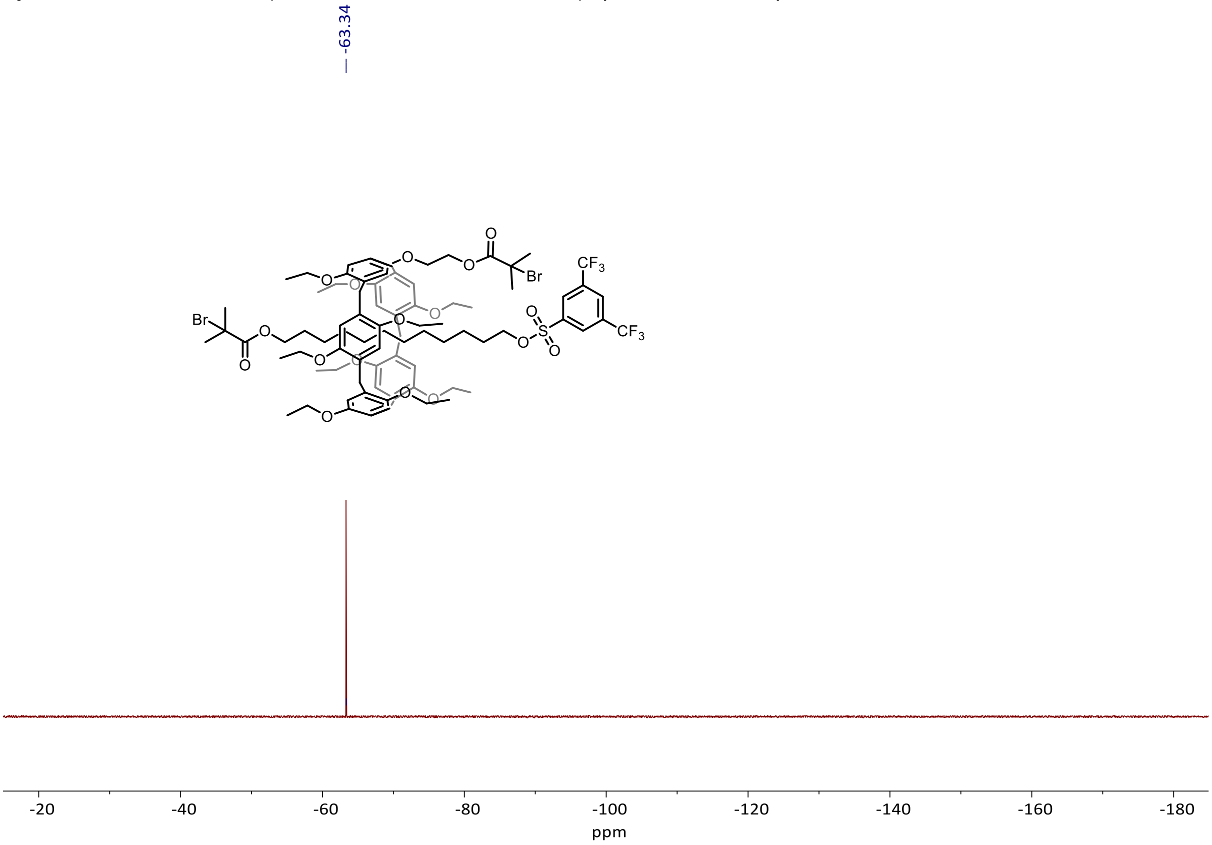

Spectrum S18. ^{19}F NMR (471 MHz, Acetone-d_6, 298 K) spectrum of compound **S12**.

8.1.9 Spectra of S13

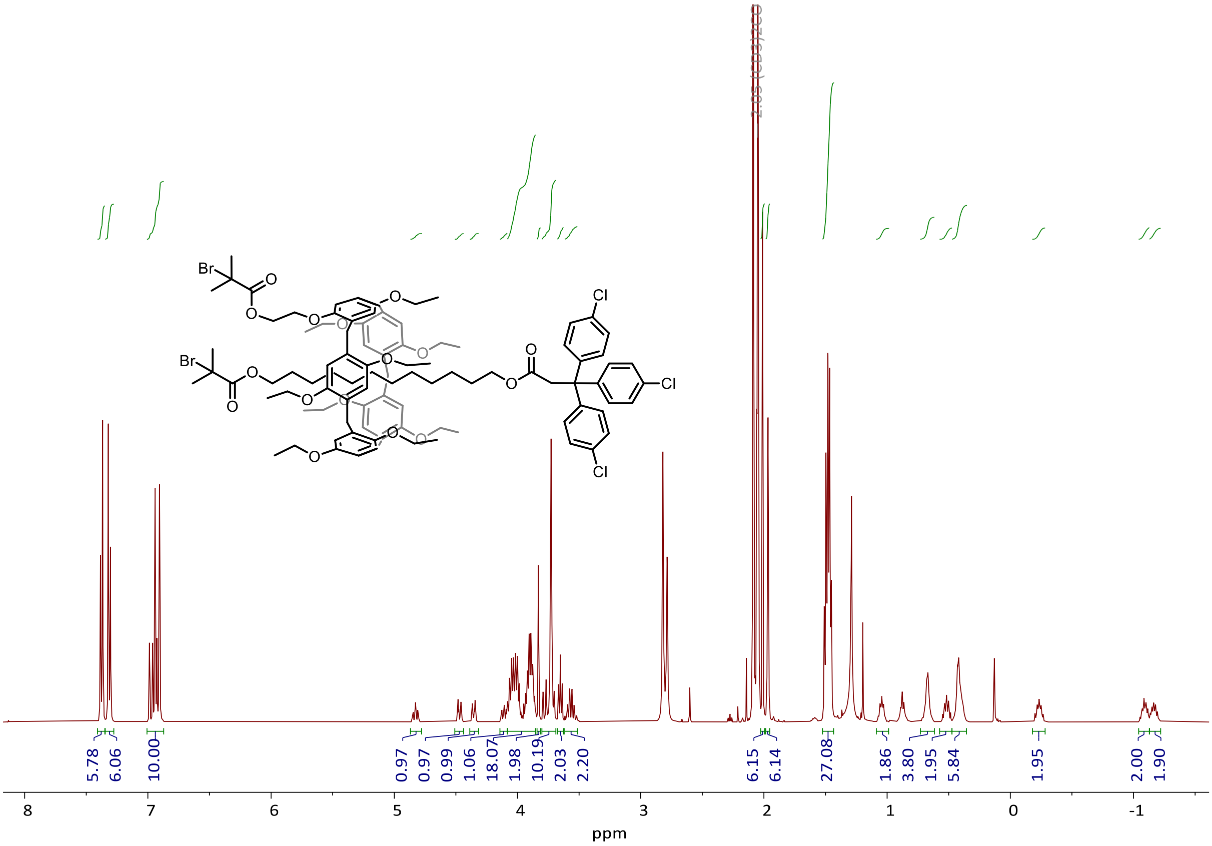

Spectrum S19. ^{1}H NMR (500 MHz, Acetone-d_6, 298 K) spectrum of compound **S13**.

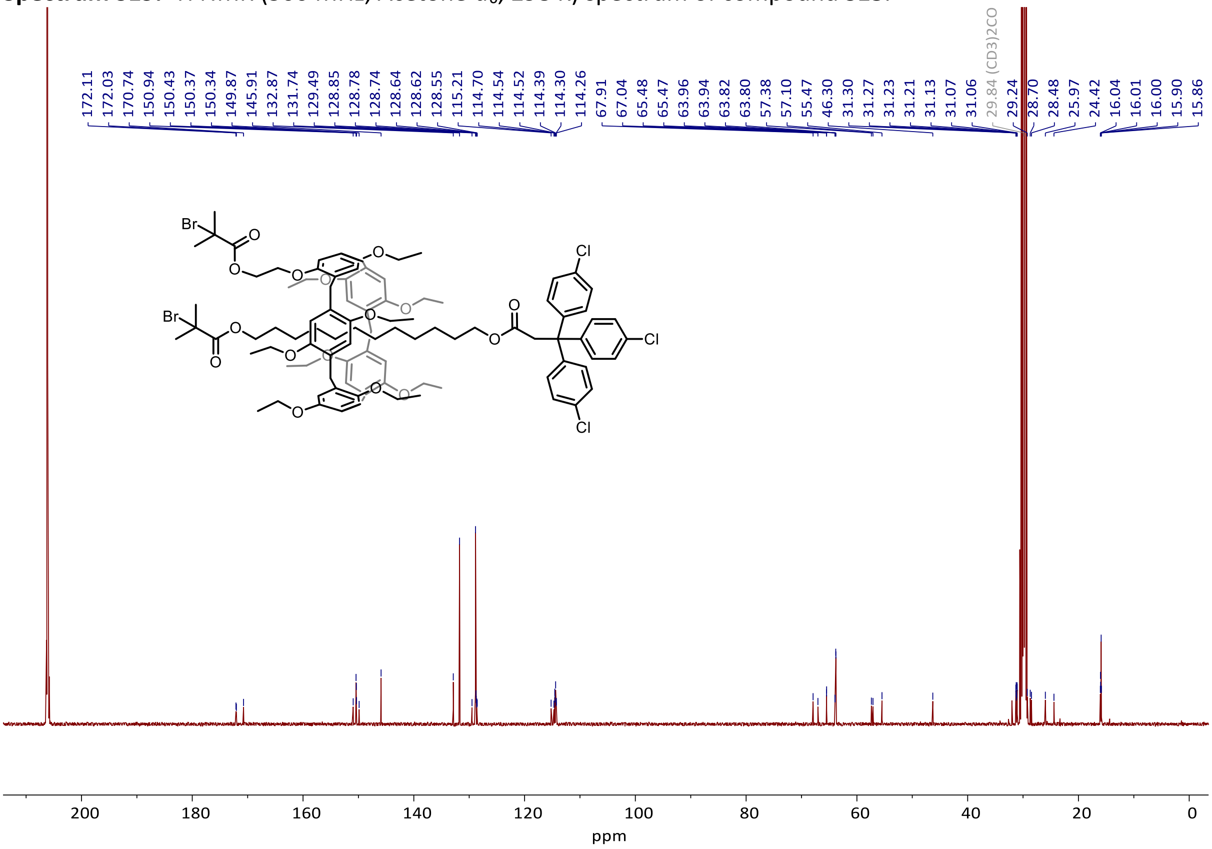

Spectrum S20. ^{13}C NMR (126 MHz, Acetone-d_6, 298 K) spectrum of compound **S13**.

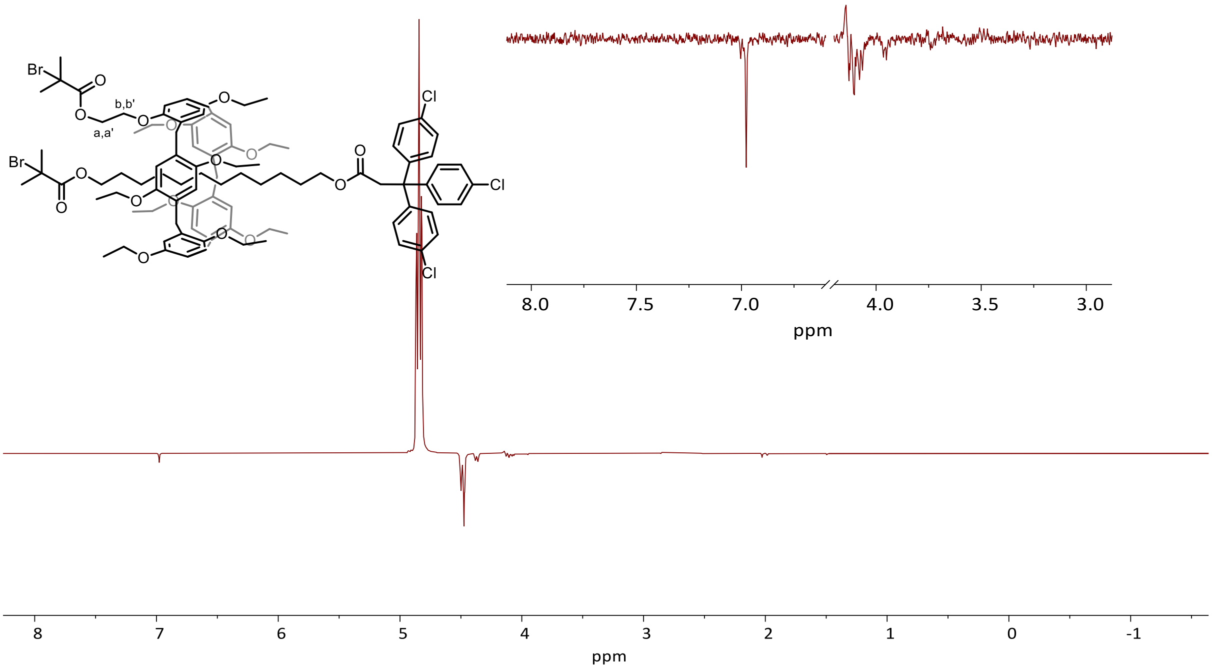

Spectrum S21. 1D selective NOESY ^{1}H NMR (500 MHz, Acetone-d_6, 298 K) spectrum of compound **S13** upon selective excitation of proton H_a.

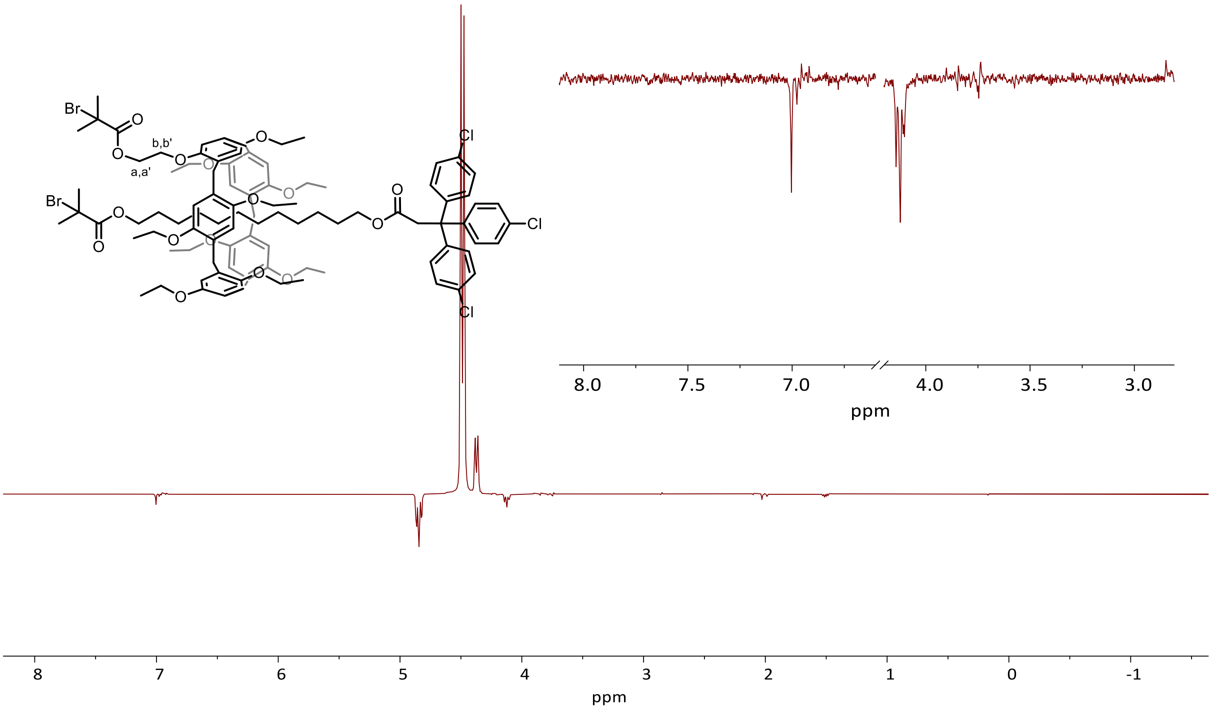

Spectrum S22. 1D selective NOESY ^{1}H NMR (500 MHz, Acetone-d_6, 298 K) spectrum of compound **S13** upon selective excitation of proton $H_{a'}$.

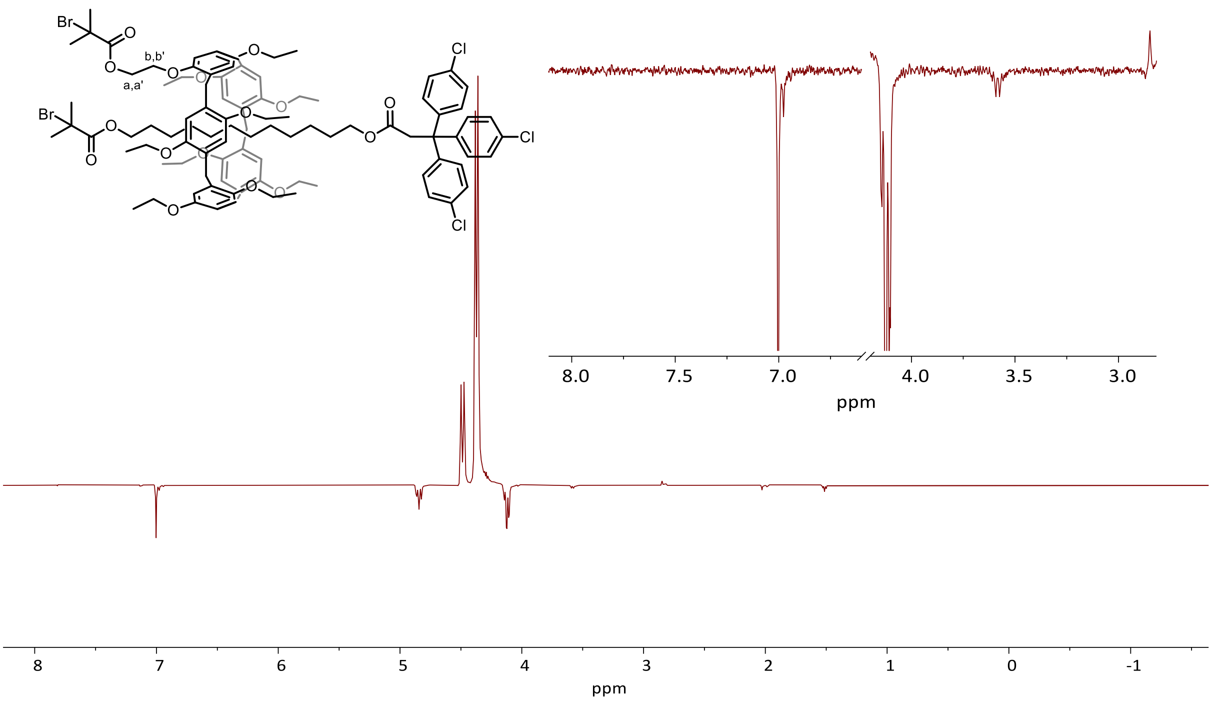

Spectrum S23. 1D selective NOESY ^{1}H NMR (500 MHz, Acetone-d_6, 298 K) spectrum of compound **S13** upon selective excitation of proton H_b.

8.1.10 Spectra of S14

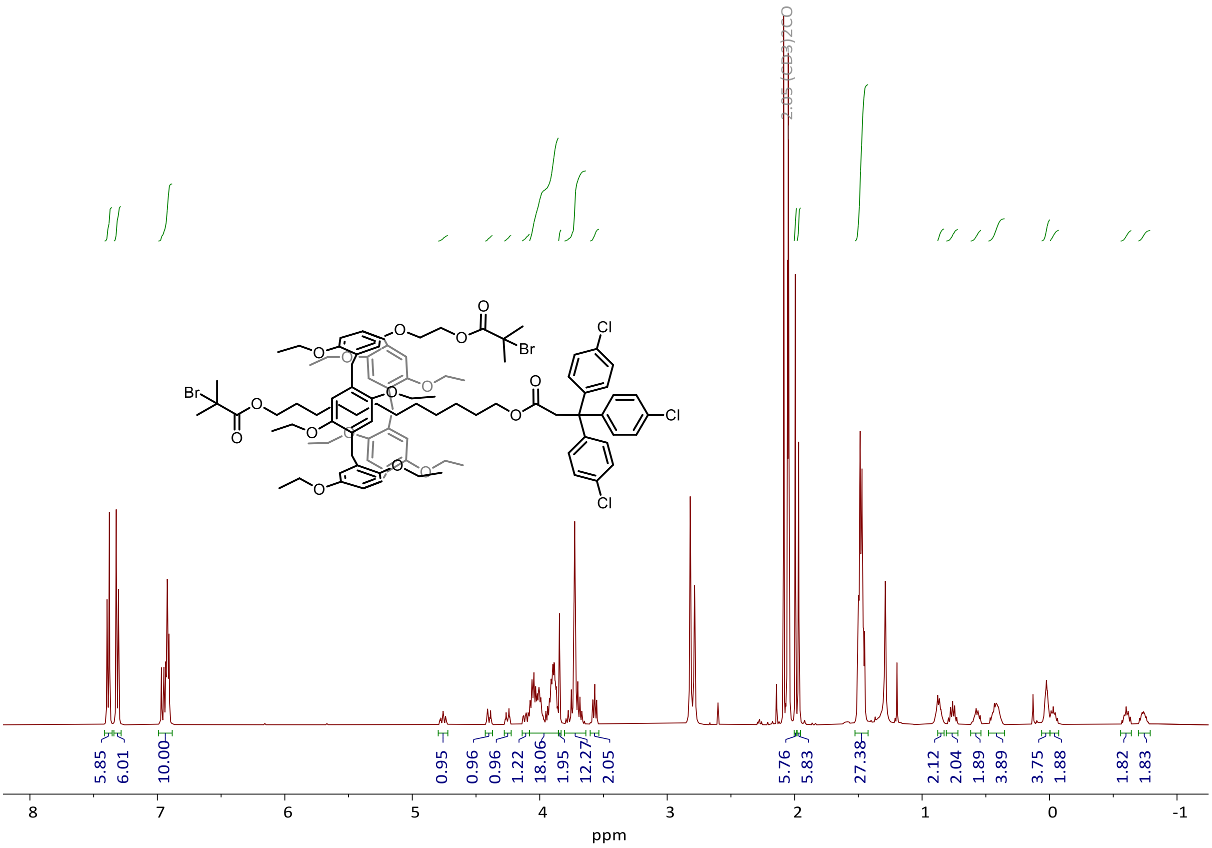

Spectrum S24. ^{1}H NMR (500 MHz, Acetone-d_6, 298 K) spectrum of compound **S14**.

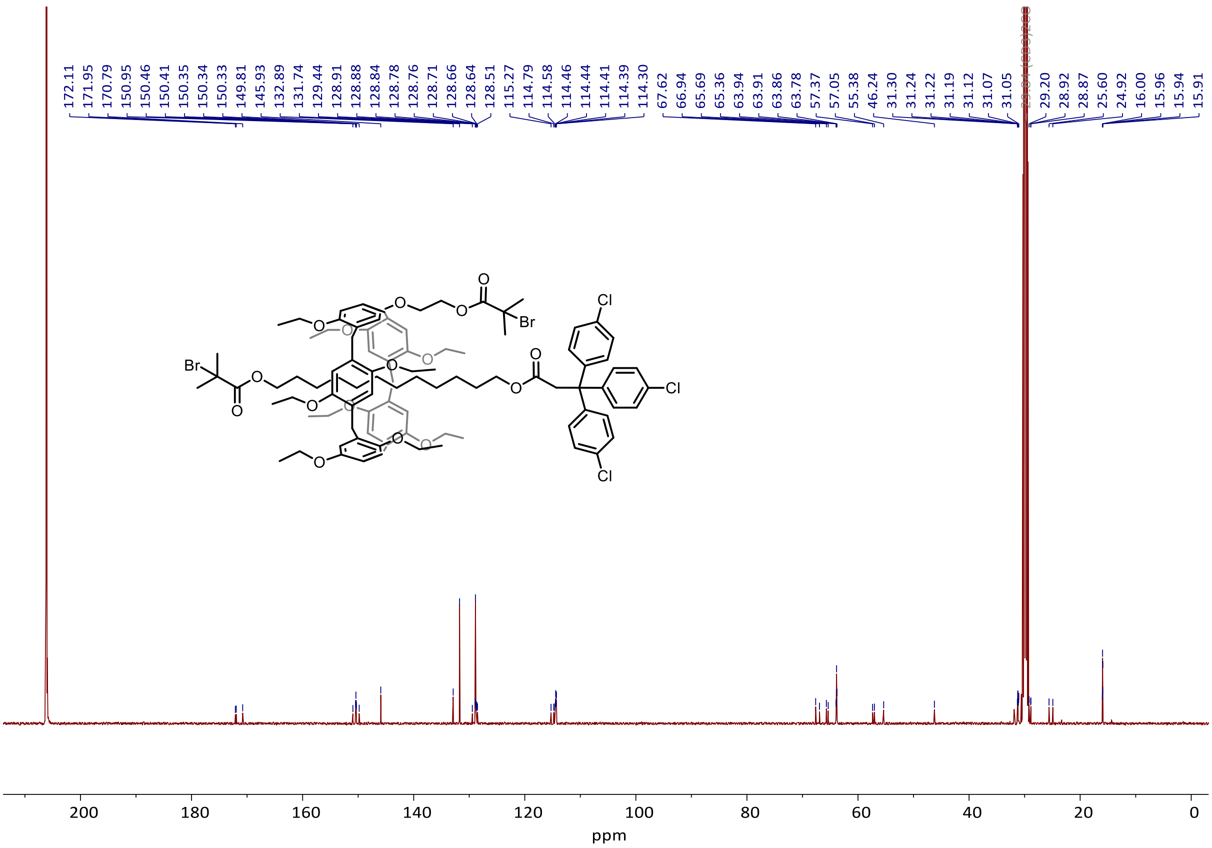

Spectrum S25. ^{13}C NMR (126 MHz, Acetone-d_6, 298 K) spectrum of compound **S14**.

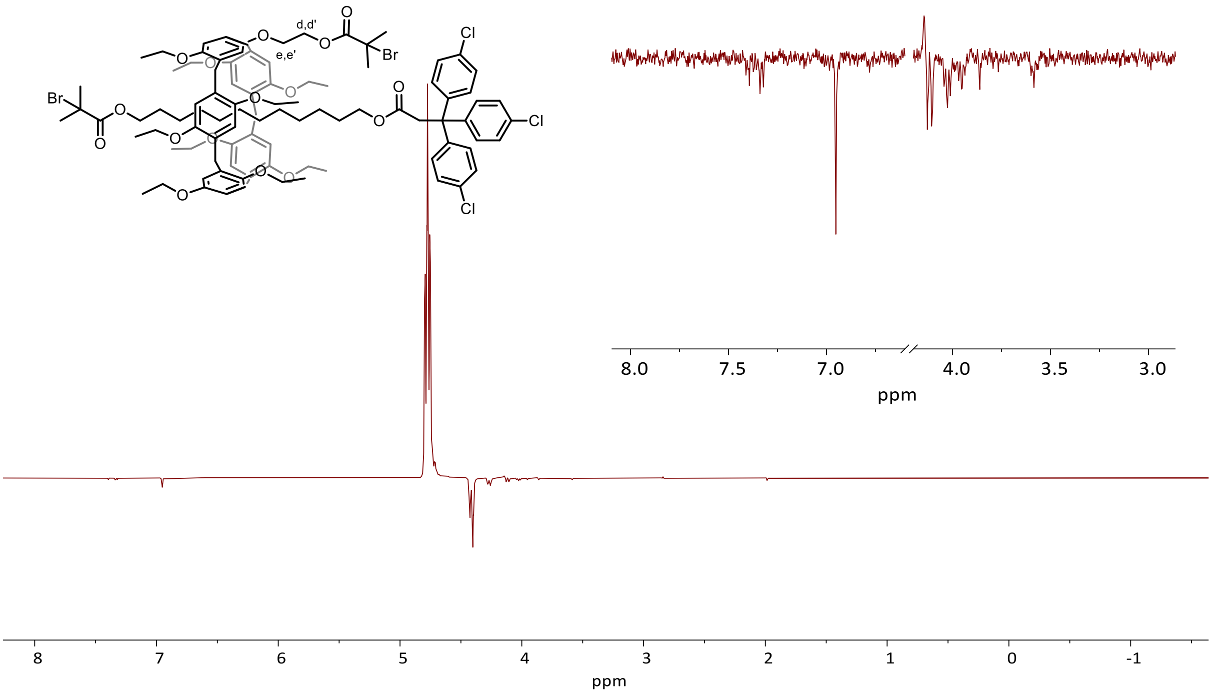

Spectrum S26. 1D selective NOESY ^{1}H NMR (500 MHz, Acetone-d_6, 298 K) spectrum of compound **S14** upon selective excitation of proton H_d.

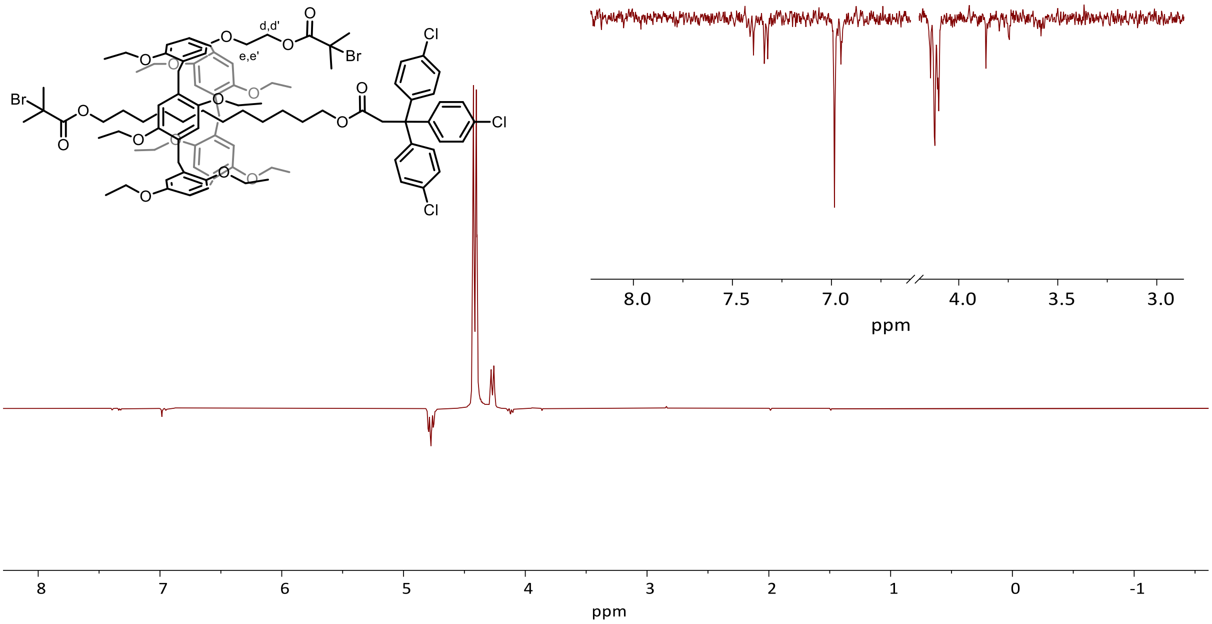

Spectrum S27. 1D selective NOESY ^{1}H NMR (500 MHz, Acetone-d_6, 298 K) spectrum of compound **S14** upon selective excitation of proton $H_{d'}$.

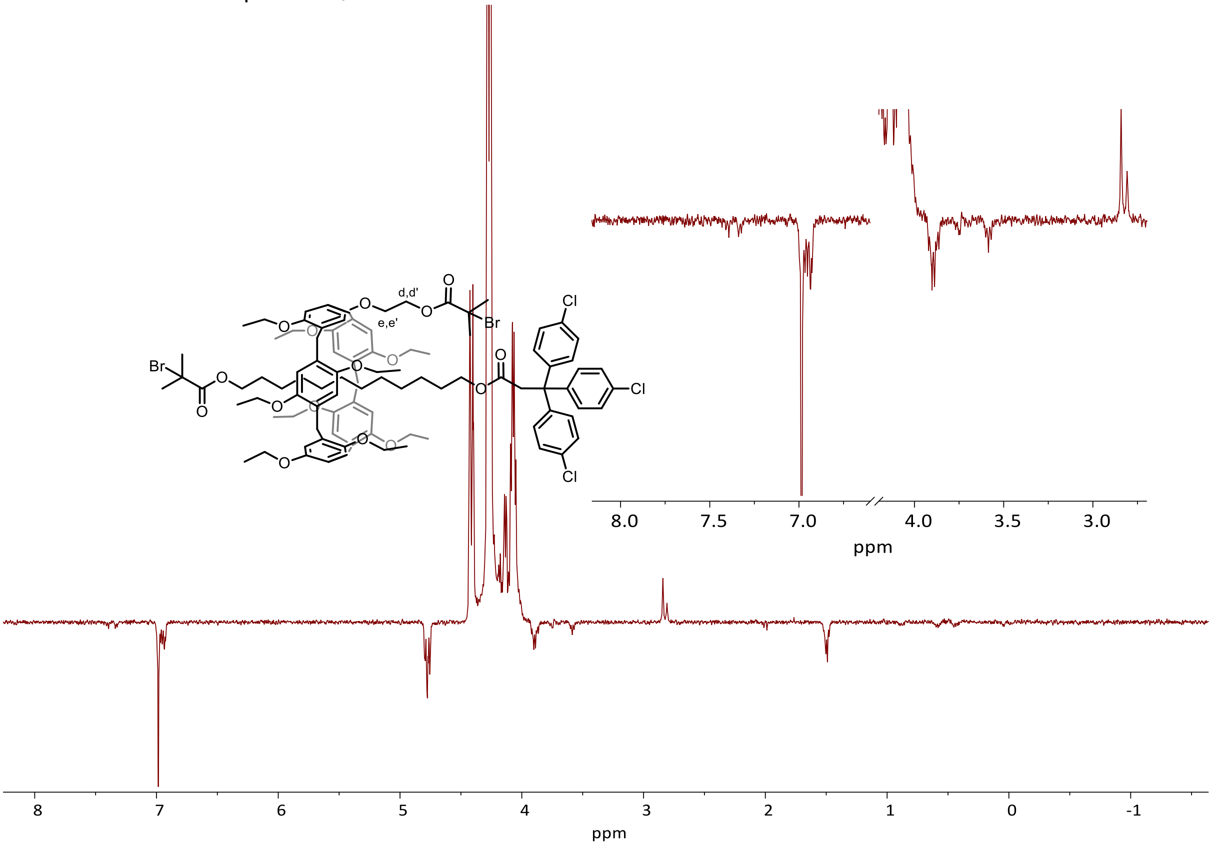

Spectrum S28. 1D selective NOESY ^{1}H NMR (500 MHz, Acetone-d_6, 298 K) spectrum of compound **S14** upon selective excitation of proton H_e.

8.2 Polymer NMR Spectra

8.2.1 Spectra of polymer $1_{cis\text{-OMe-145}}$

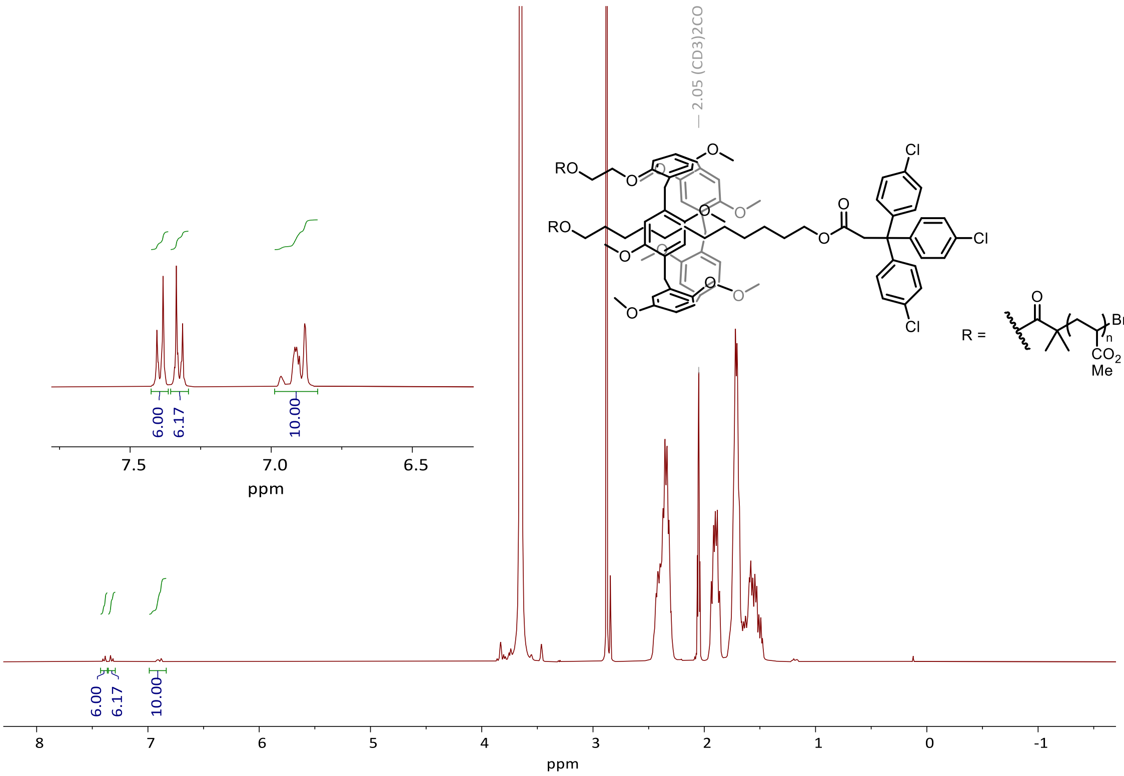

Spectrum S29. ^{1}H NMR (400 MHz, Acetone-d_6, 298 K) spectrum of polymer $\mathbf{1}_{cis\text{-OMe-145}}$.

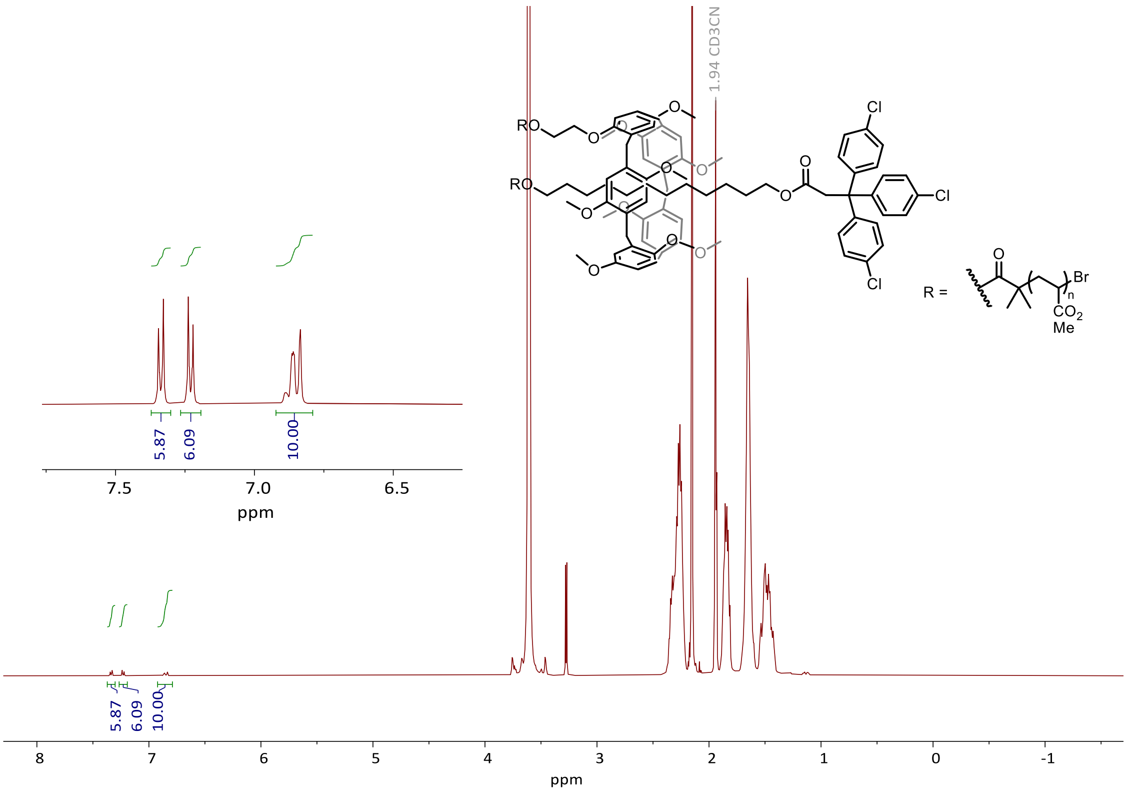

Spectrum S30. ^{1}H NMR (500 MHz, Acetonitrile-d_6, 298 K) spectrum of polymer $\mathbf{1}_{cis\text{-OMe-145}}$.

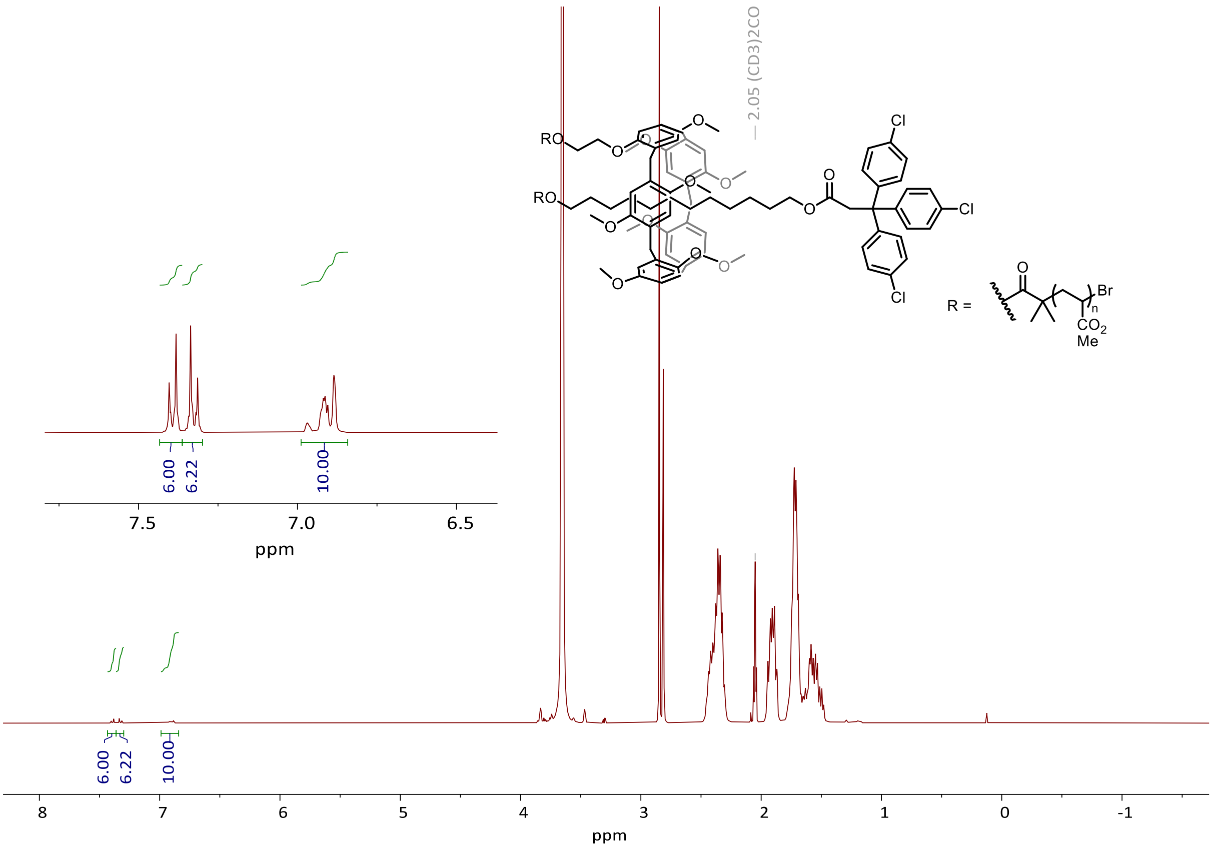

Spectrum S31. ^{1}H NMR (400 MHz, Acetone-d_6, 298 K) spectrum of polymer $\mathbf{1}_{\mathit{cis}\text{-OMe-166}}$.

8.2.2 Spectra of polymer $\mathbf{1}_{\mathit{cis}\text{-OEt}}$

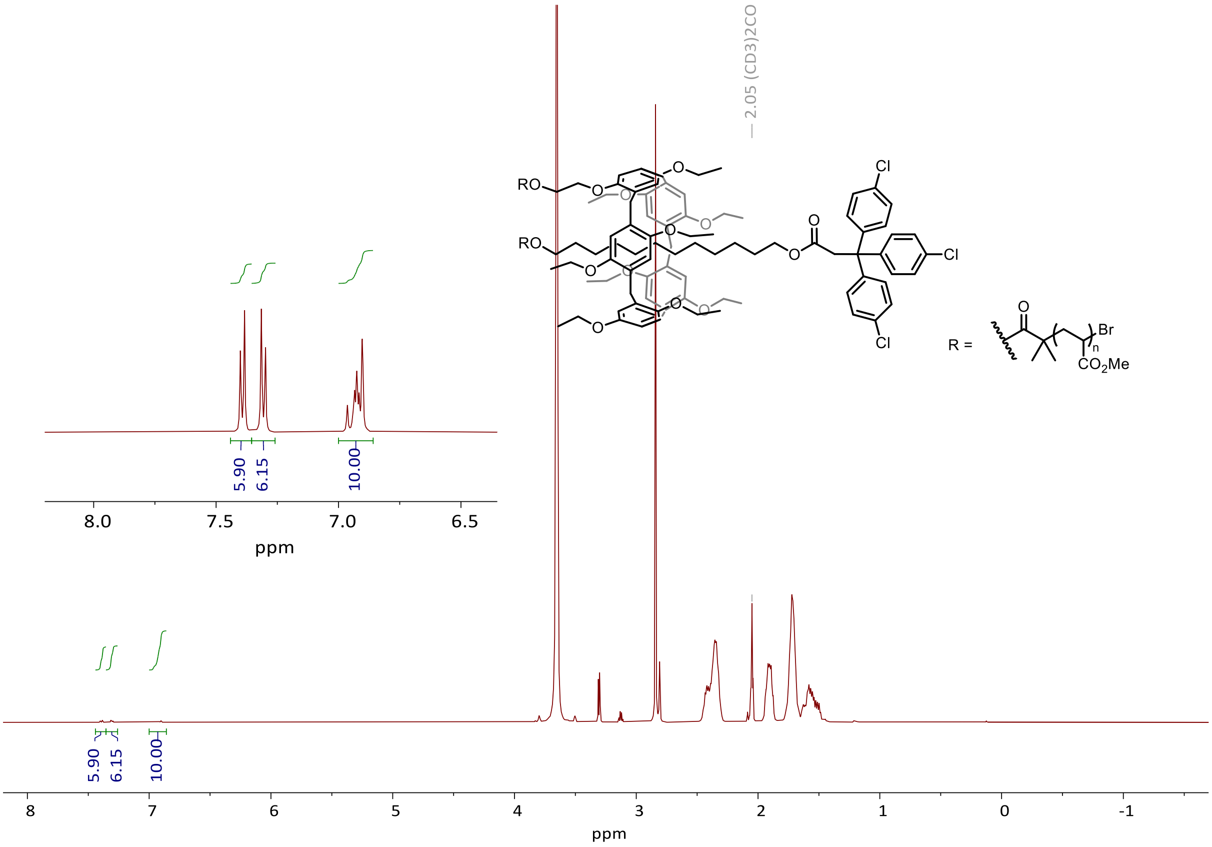

Spectrum S32. ^{1}H NMR (500 MHz, Acetone-d_6, 298 K) spectrum of polymer $\mathbf{1}_{\mathit{cis}\text{-OEt}}$.

8.2.3 Spectra of polymer S19

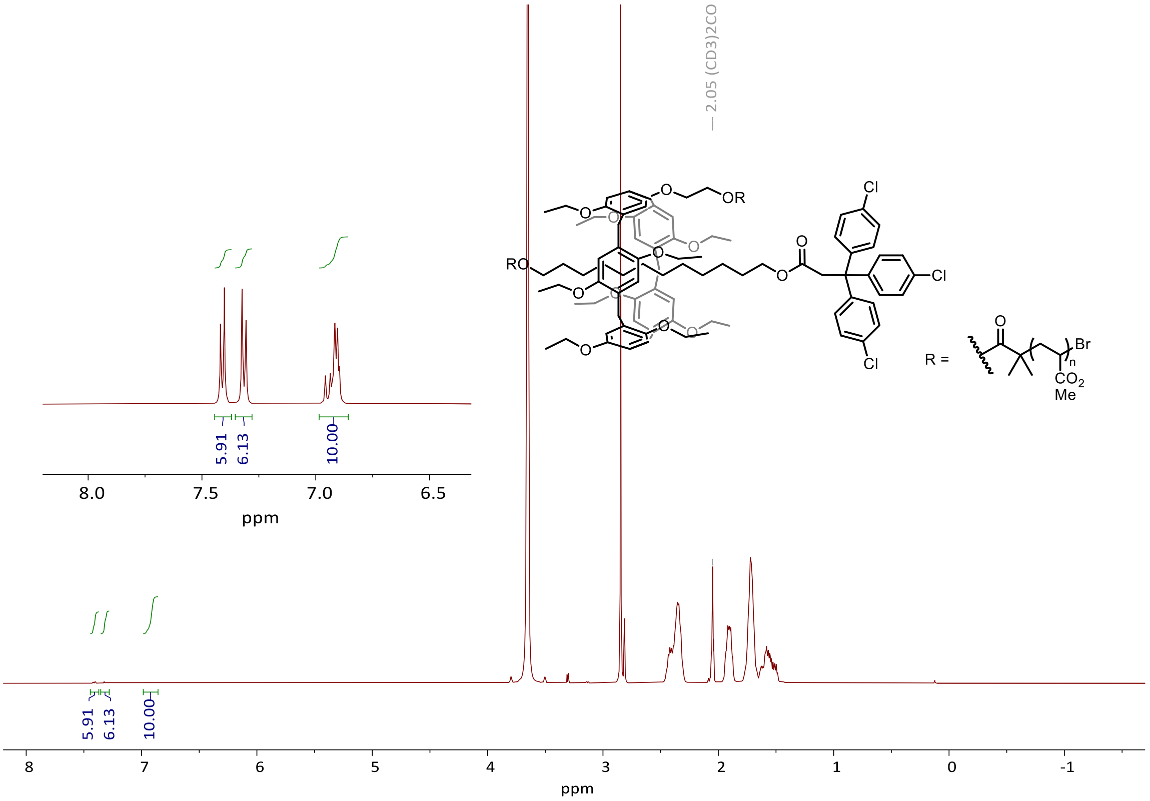

Spectrum S33. ^{1}H NMR (500 MHz, Acetone-d_6, 298 K) spectrum of polymer **S19**.

8.2.4 Spectra of polymer 3_{cis}

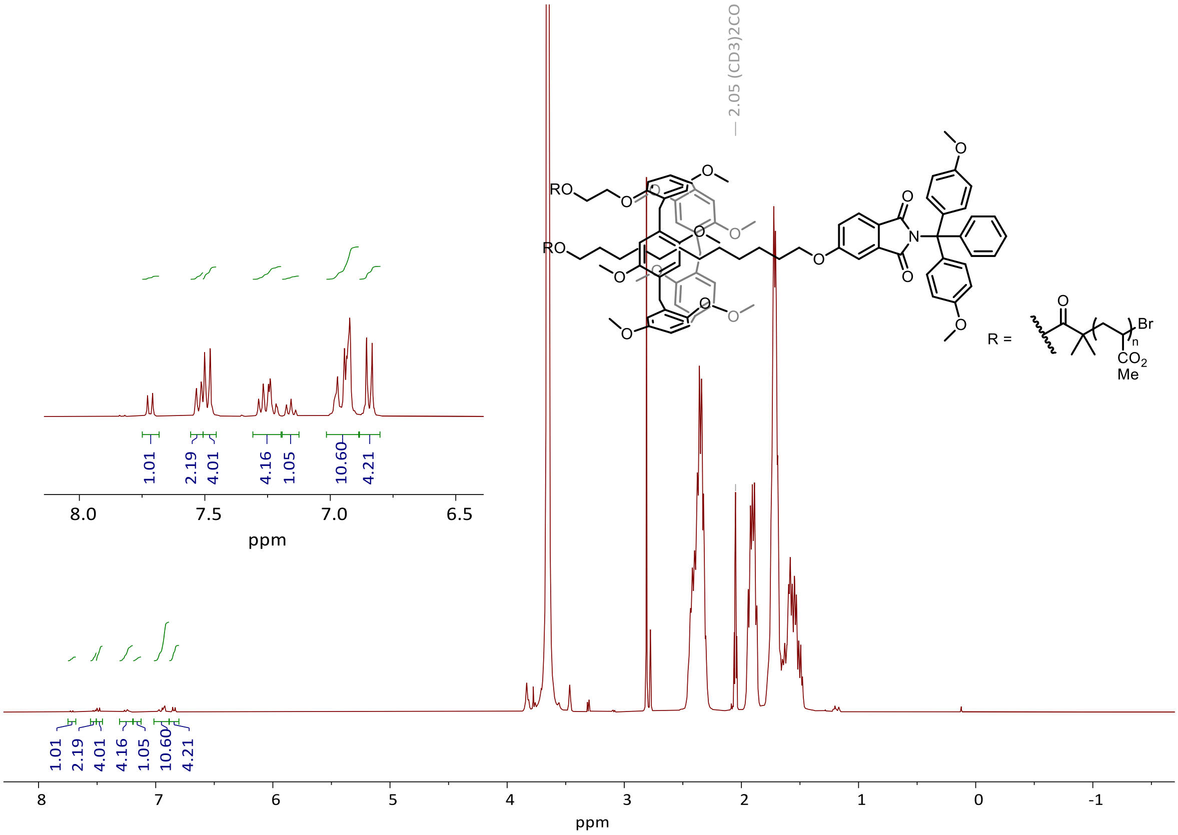

Spectrum S34. ^{1}H NMR (400 MHz, Acetone-d_6, 298 K) spectrum of polymer **3_{cis}**.

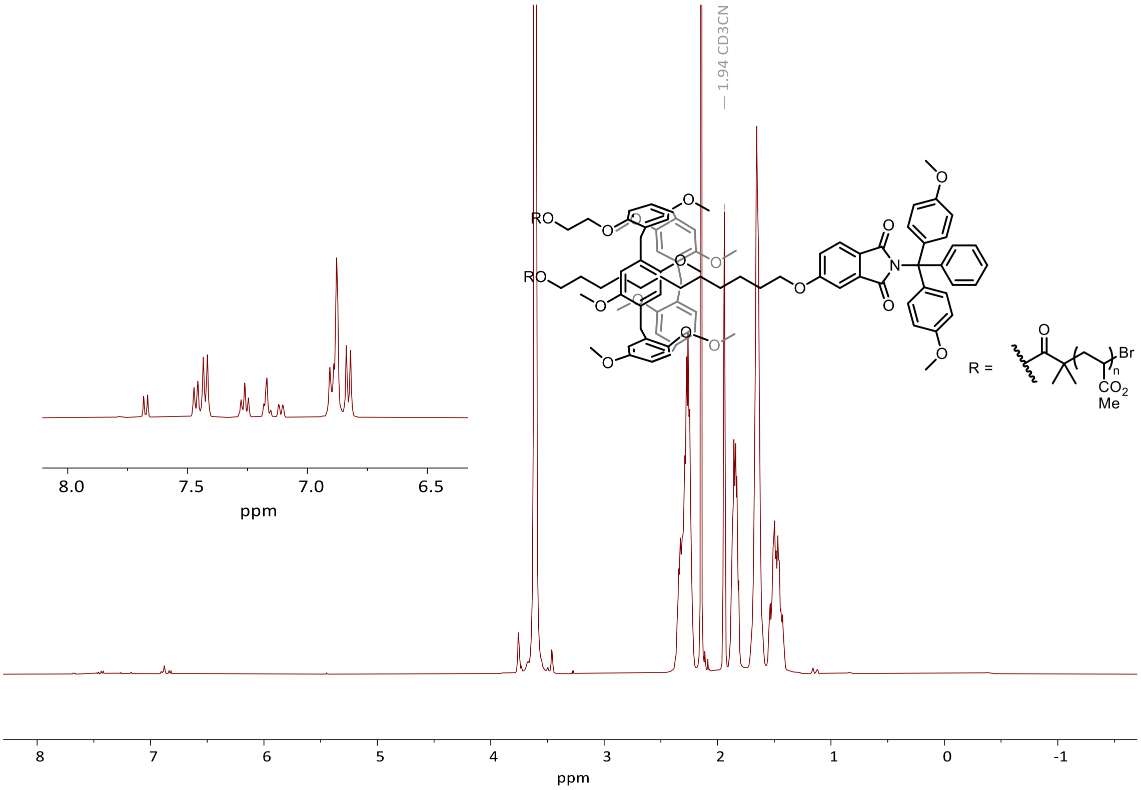

Spectrum S35. ^{1}H NMR (500 MHz, Acetonitrile-d_6, 298 K) spectrum of polymer **3*cis***.

8.2.5 Spectra of polymer S23

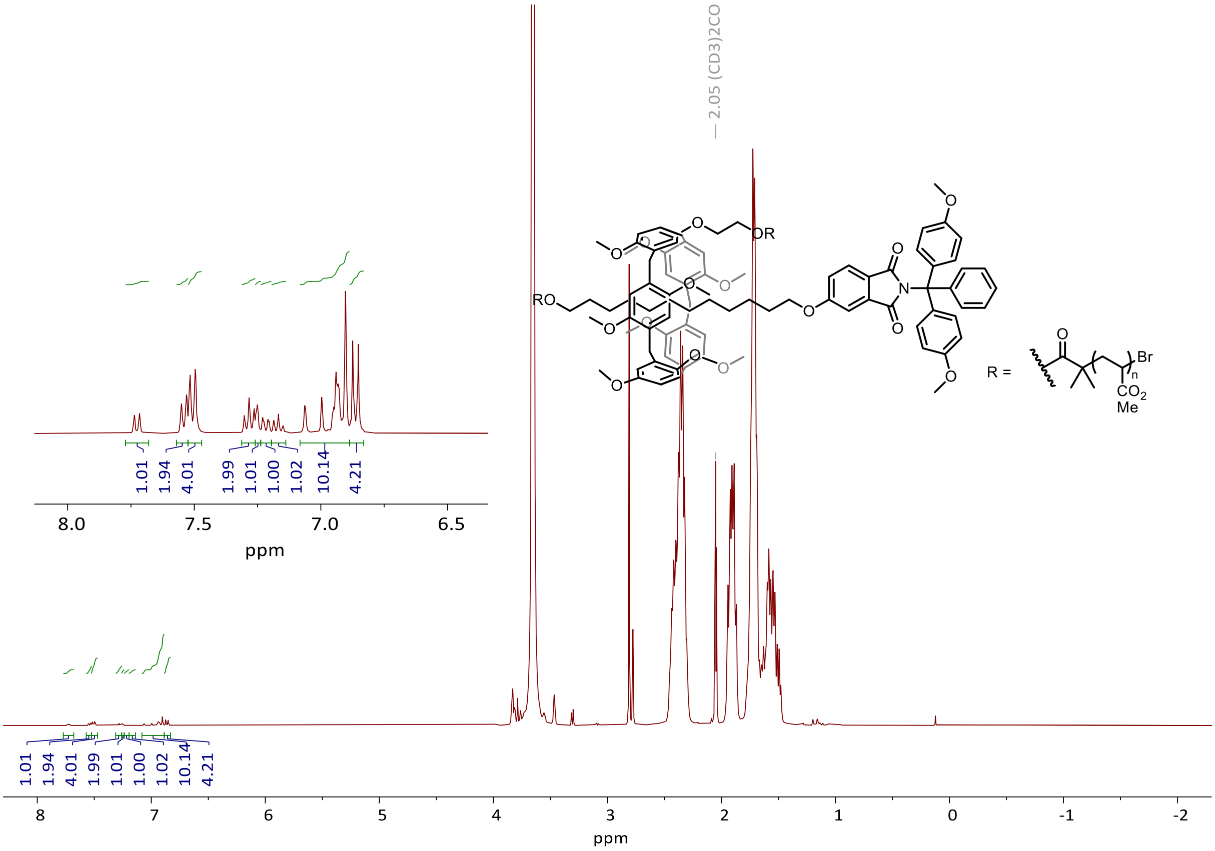

Spectrum S36. ^{1}H NMR (400 MHz, Acetone-d_6, 298 K) spectrum of polymer **S23**.

8.2.6 Spectra of polymer $1_{trans\text{-OMe}}$

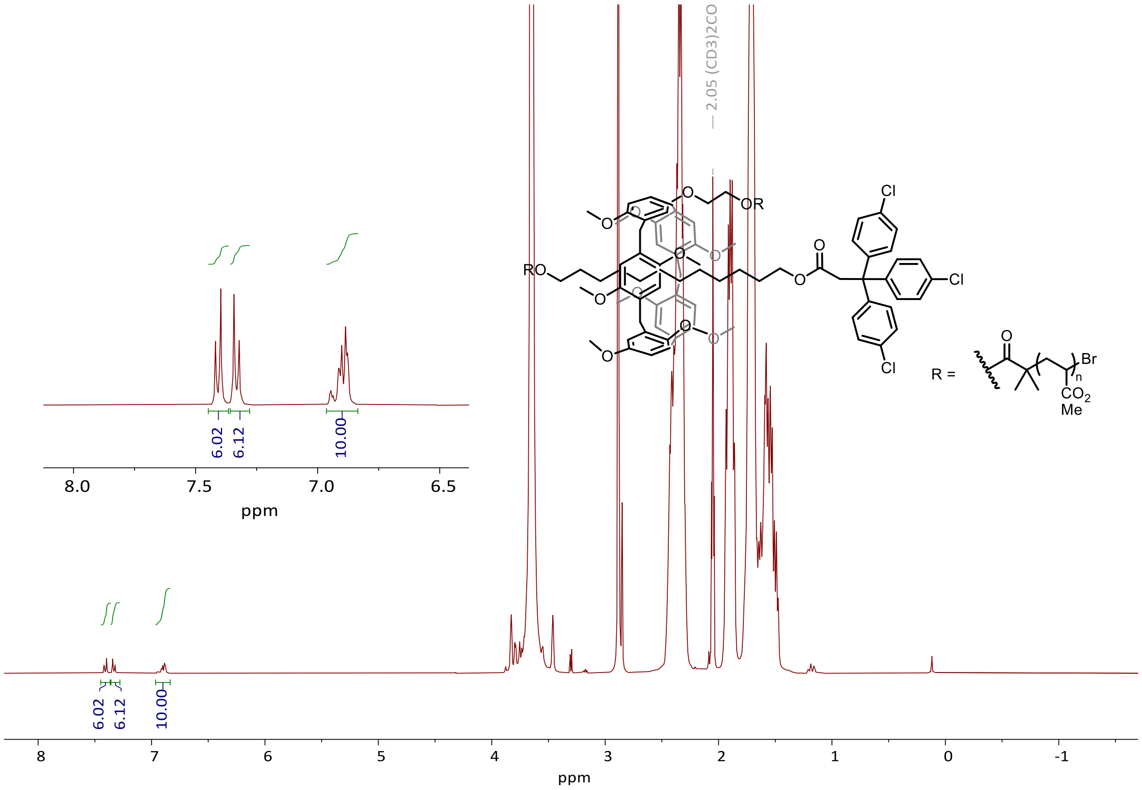

Spectrum S37. ^{1}H NMR (400 MHz, Acetone-d_6, 298 K) spectrum of polymer $\mathbf{1}_{trans\text{-OMe}}$.

8.2.7 Spectra of polymer 2_{cis}

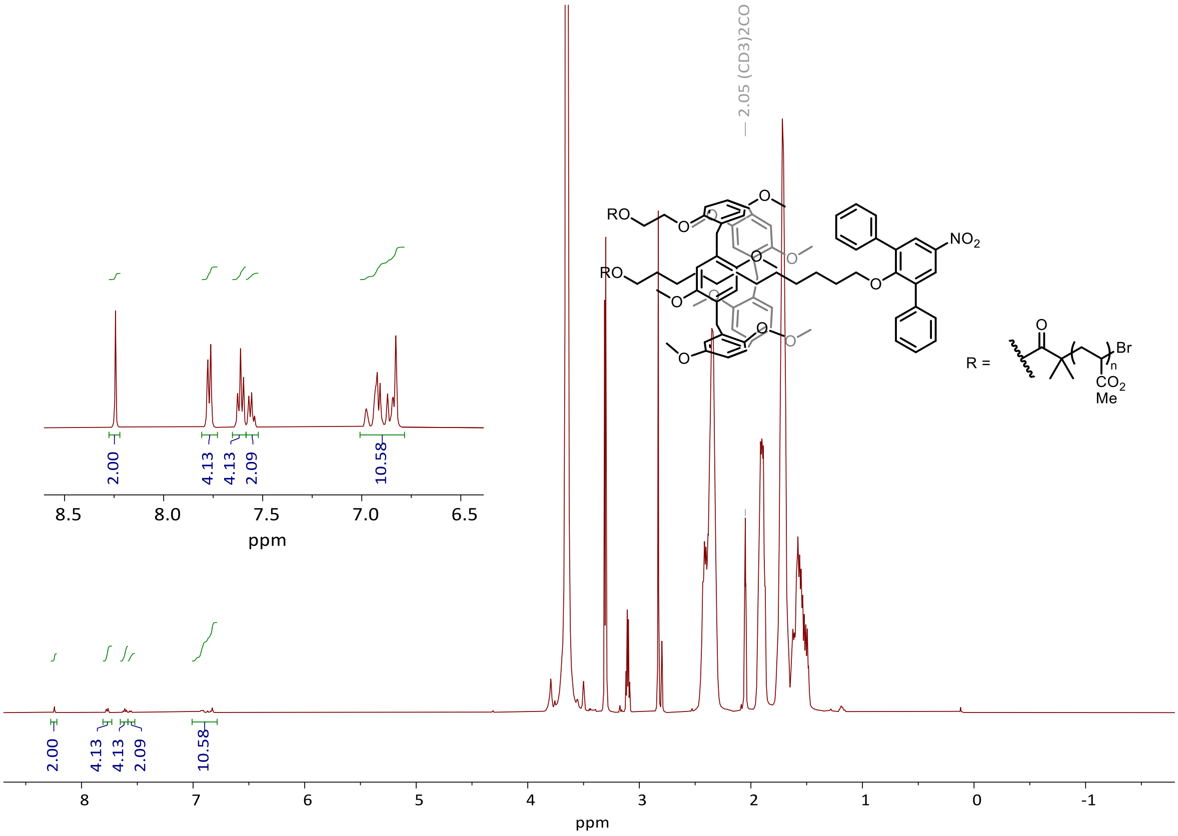

Spectrum S38. ^{1}H NMR (500 MHz, Acetone-d_6, 298 K) spectrum of polymer $\mathbf{2}_{cis}$.

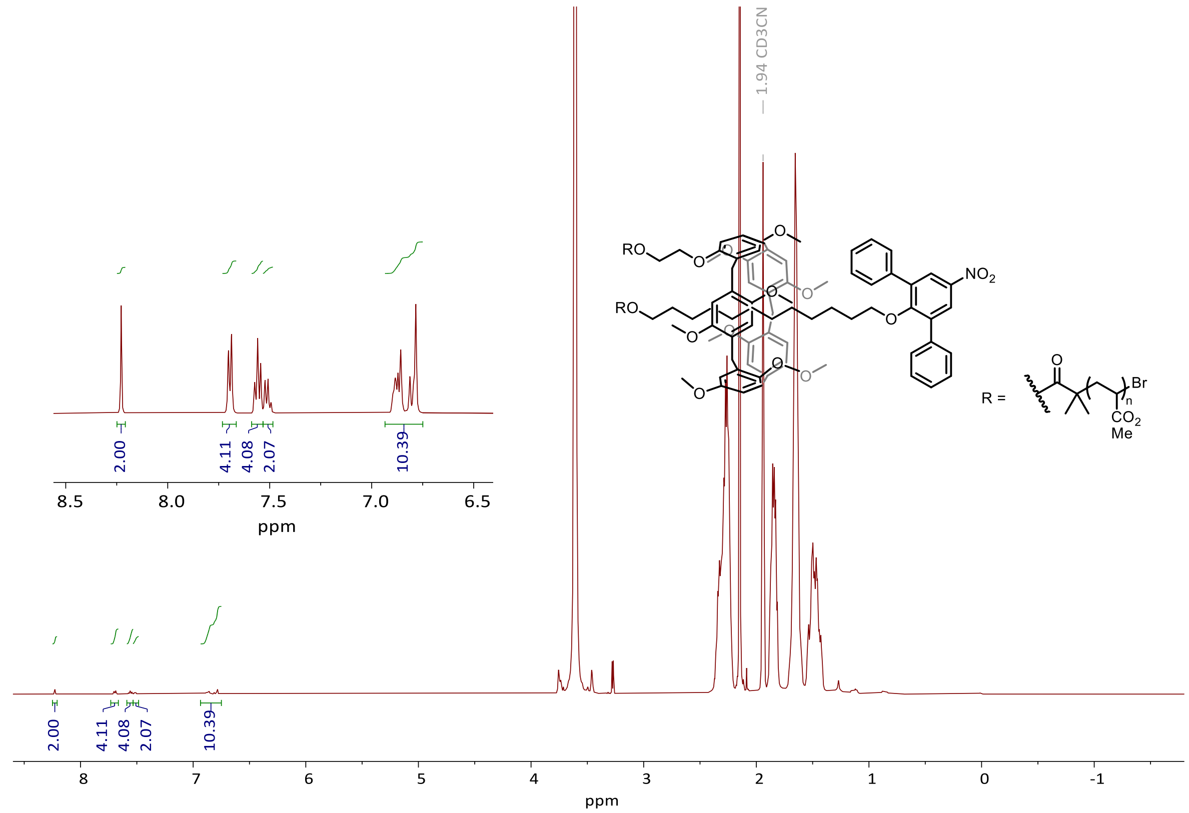

Spectrum S39. ^{1}H NMR (500 MHz, Acetonitrile-d_6, 298 K) spectrum of polymer $\mathbf{2}_{cis}$.

8.2.8 Spectra of polymer S20

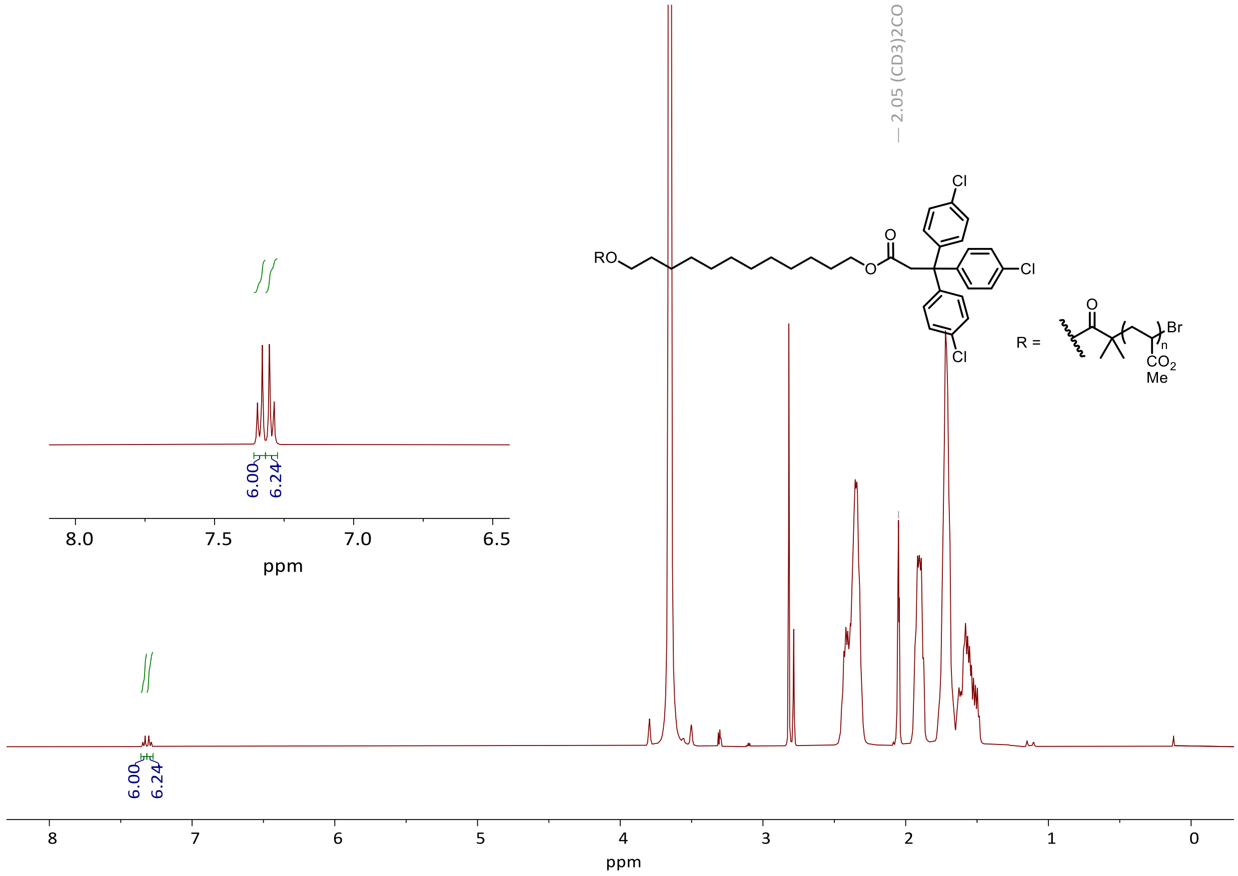

Spectrum S40. ^{1}H NMR (500 MHz, Acetone-d_6, 298 K) spectrum of polymer **S20**.

8.2.9 Spectra of polymer S21

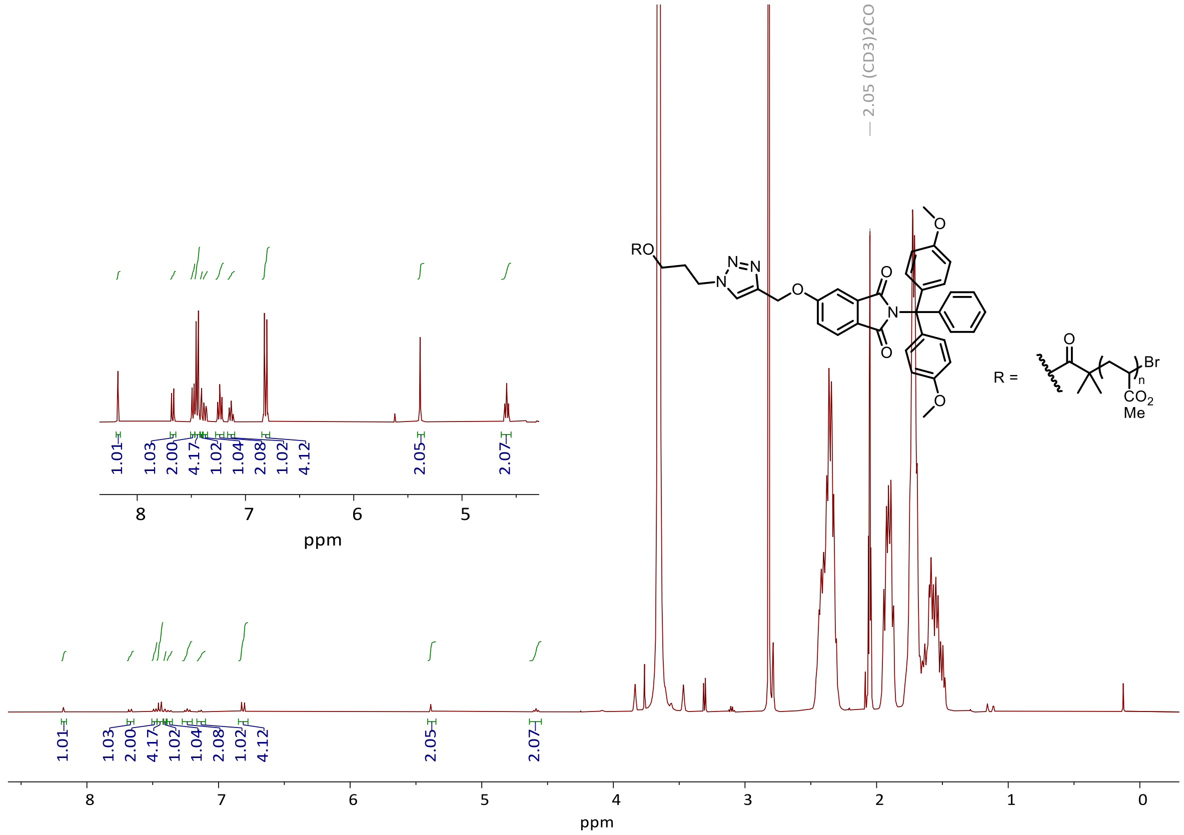

Spectrum S41. ^{1}H NMR (400 MHz, Acetone-d_6, 298 K) spectrum of polymer **S21**

8.2.10 Spectra of polymer 6_{OMe}

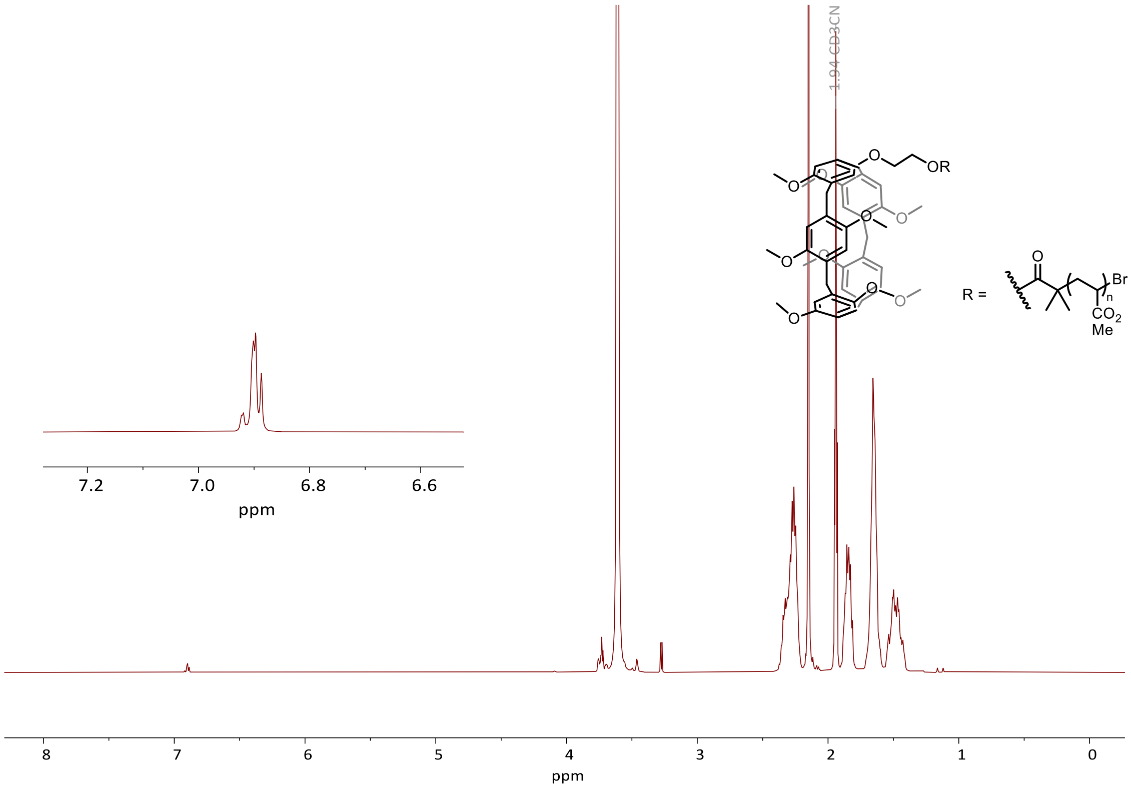

Spectrum S42. ^{1}H NMR (500 MHz, Acetonitrile-d_6, 298 K) spectrum of polymer $\mathbf{6_{OMe}}$.

8.3 Post-Sonication NMR Spectra

8.3.1 Post-Sonication ^{1}H NMR Spectra of Polymer $1_{cis\text{-}OMe\text{-}145}$ (Run 1)

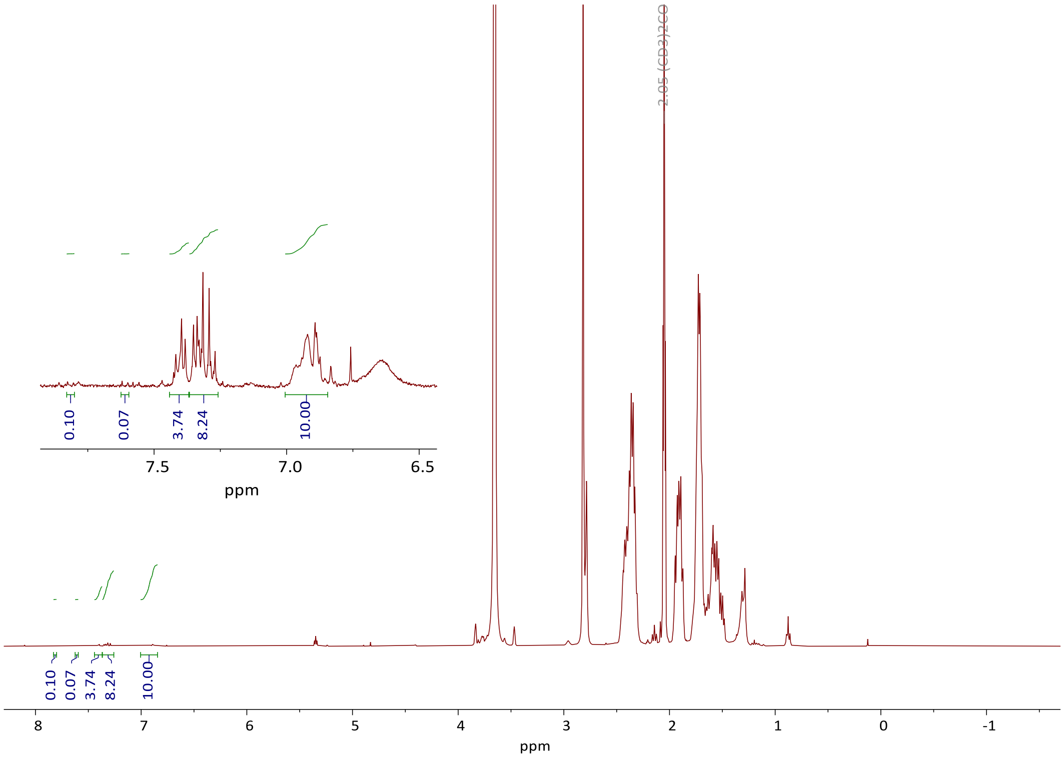

Spectrum S43. ^{1}H NMR (400 MHz, Acetone-d_6, 298 K) spectrum of post-sonication polymer **$1_{cis\text{-}OMe\text{-}145}$** before being washed with methanol.

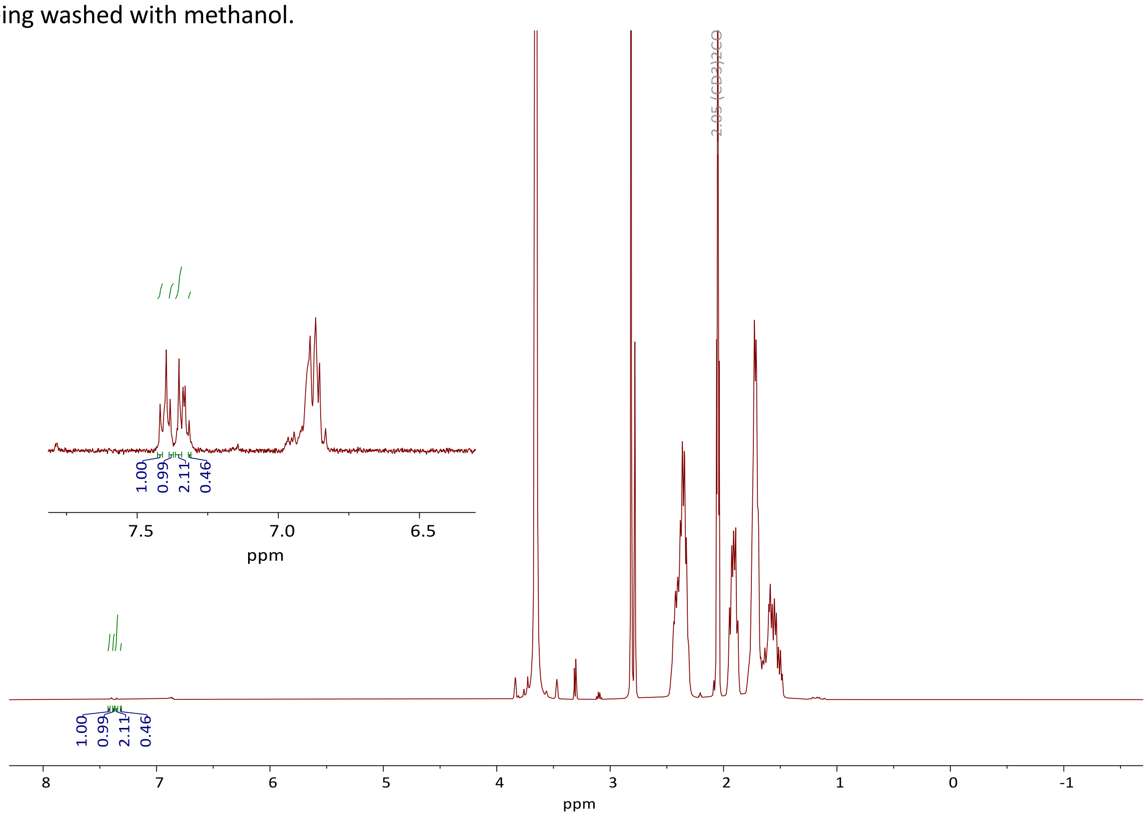

Spectrum S44. ^{1}H NMR (400 MHz, Acetone-d_6, 298 K) spectrum of post-sonication polymer **$1_{cis\text{-}OMe\text{-}145}$** after being washed with methanol.

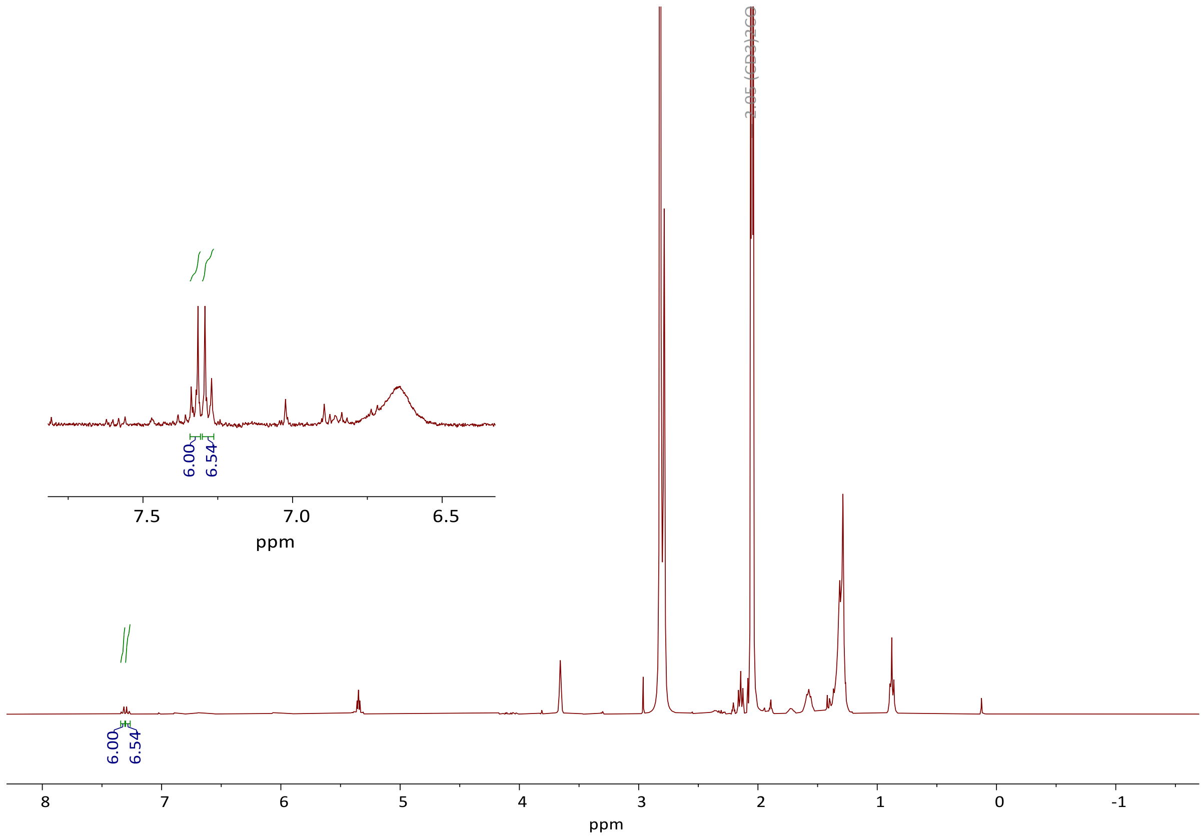

Spectrum S45. ^{1}H NMR (400 MHz, Acetone-d_6, 298 K) spectrum of the MeOH extract from post-sonication polymer $\mathbf{1}_{\boldsymbol{cis}\text{-}\mathbf{OMe}\text{-}\mathbf{145}}$.

8.3.2 Post-Sonication ^{1}H NMR Spectra of Polymer $\mathbf{1}_{\boldsymbol{cis}\text{-}\mathbf{OMe}\text{-}\mathbf{145}}$ (Run 2)

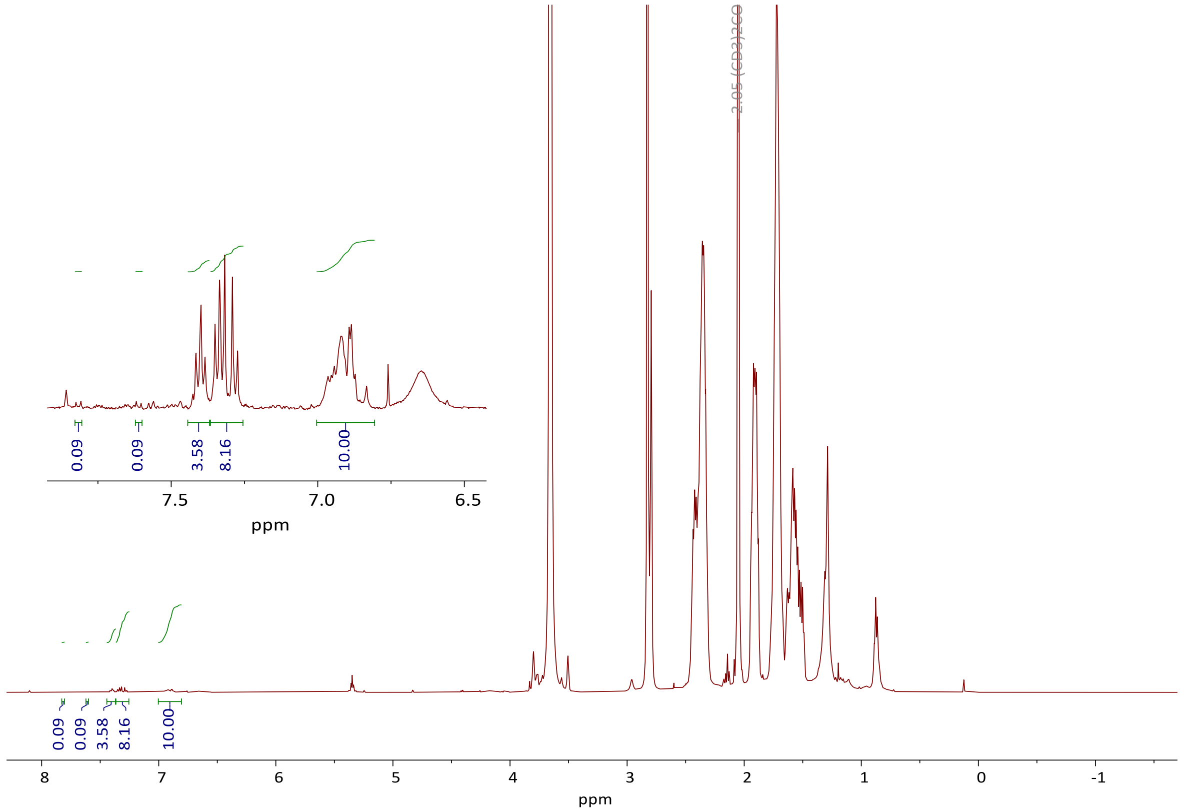

Spectrum S46. ^{1}H NMR (400 MHz, Acetone-d_6, 298 K) spectrum of post-sonication polymer $\mathbf{1}_{\boldsymbol{cis}\text{-}\mathbf{OMe}\text{-}\mathbf{145}}$ before being washed with methanol.

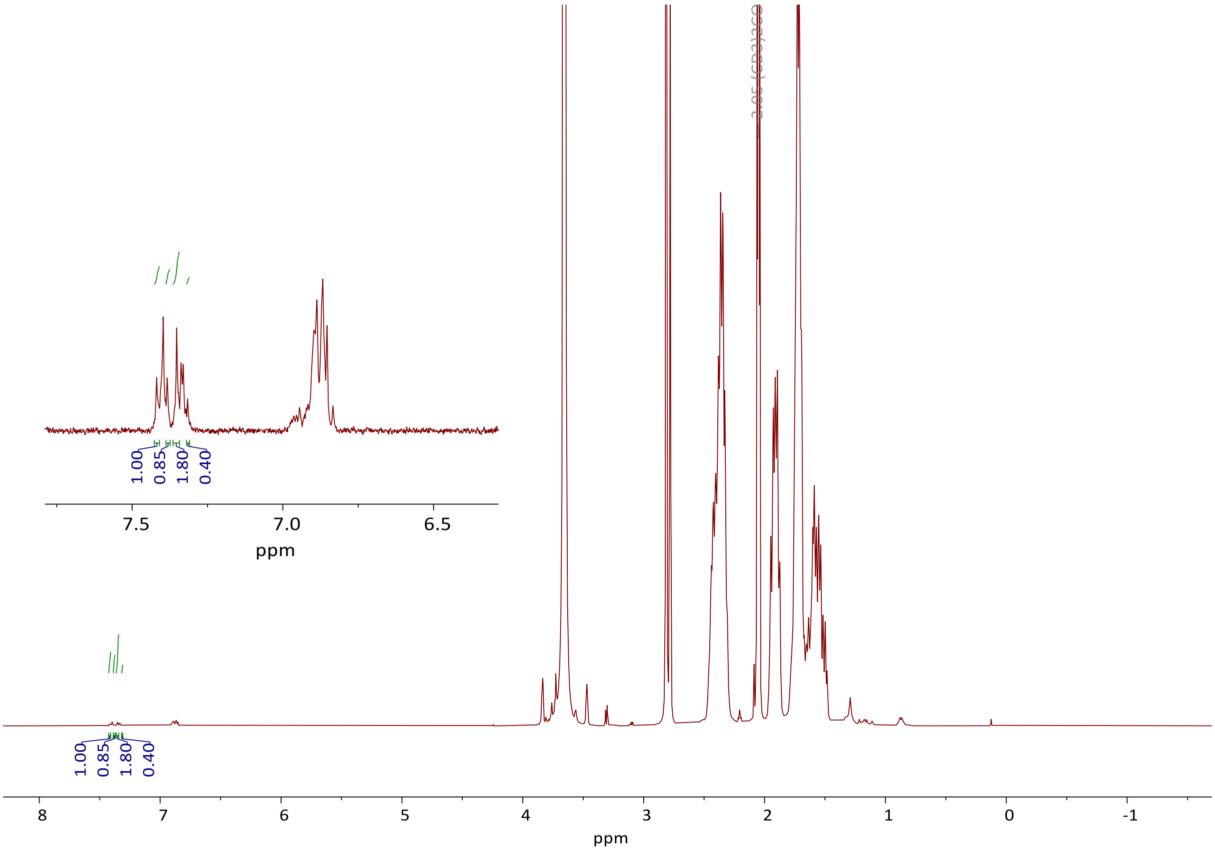

Spectrum S47. ^{1}H NMR (400 MHz, Acetone-d_6, 298 K) spectrum of post-sonication polymer $\mathbf{1}_{\textit{cis}\text{-OMe-145}}$ after being washed with methanol.

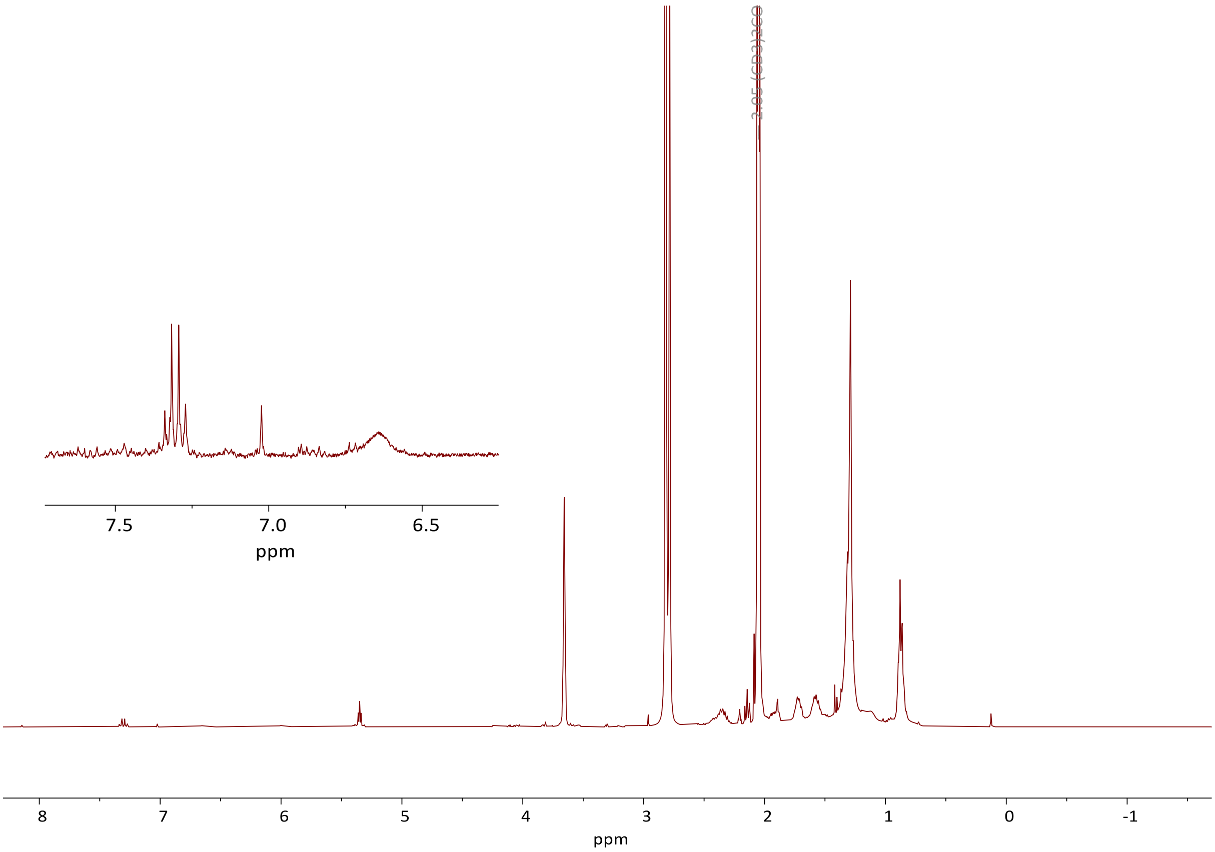

Spectrum S48. ^{1}H NMR (400 MHz, Acetone-d_6, 298 K) spectrum of the MeOH extract from post-sonication polymer $\mathbf{1}_{\textit{cis}\text{-OMe-145}}$.

8.3.3 Post-Sonication ^{1}H NMR Spectra of Polymer $\mathbf{1}_{cis\text{-OMe-145}}$ (Run 1 and run 2)

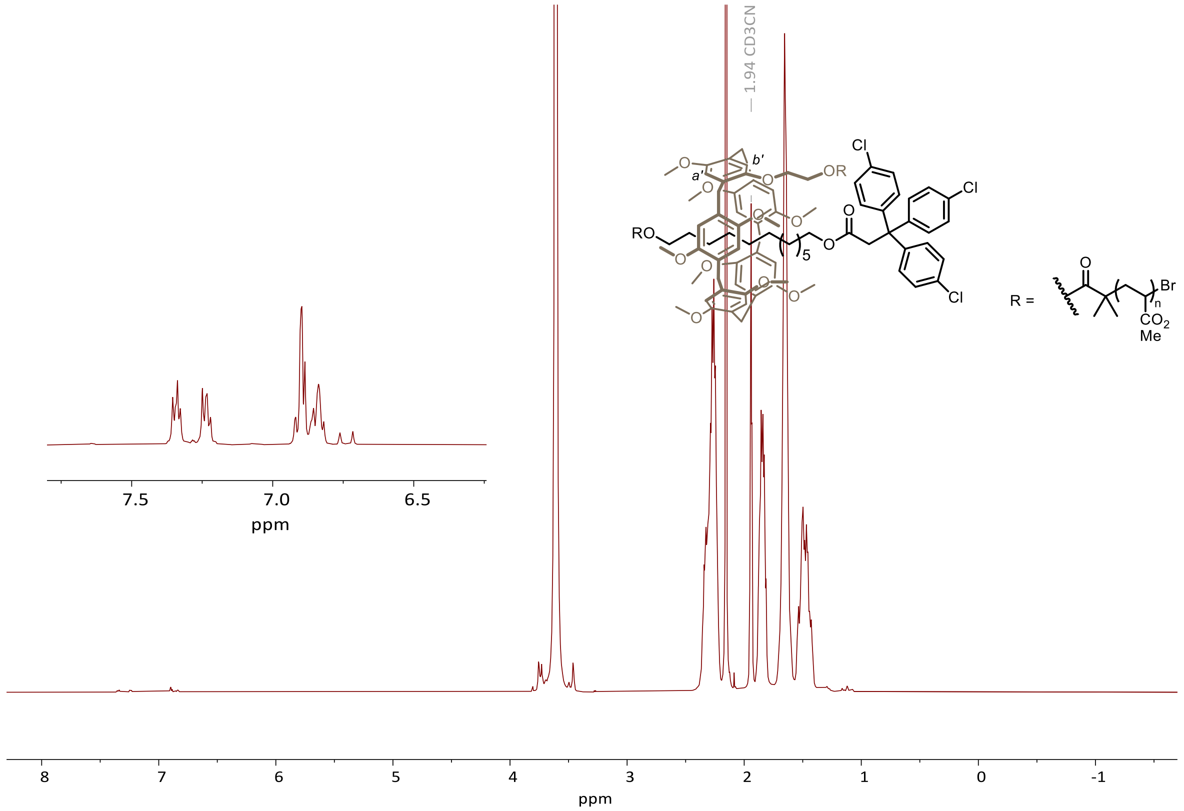

Spectrum S49. ^{1}H NMR (500 MHz, Acetonitrile-d_6, 298 K) spectrum of post-sonication polymer $\mathbf{1}_{cis\text{-OMe-145}}$ after being washed with methanol.

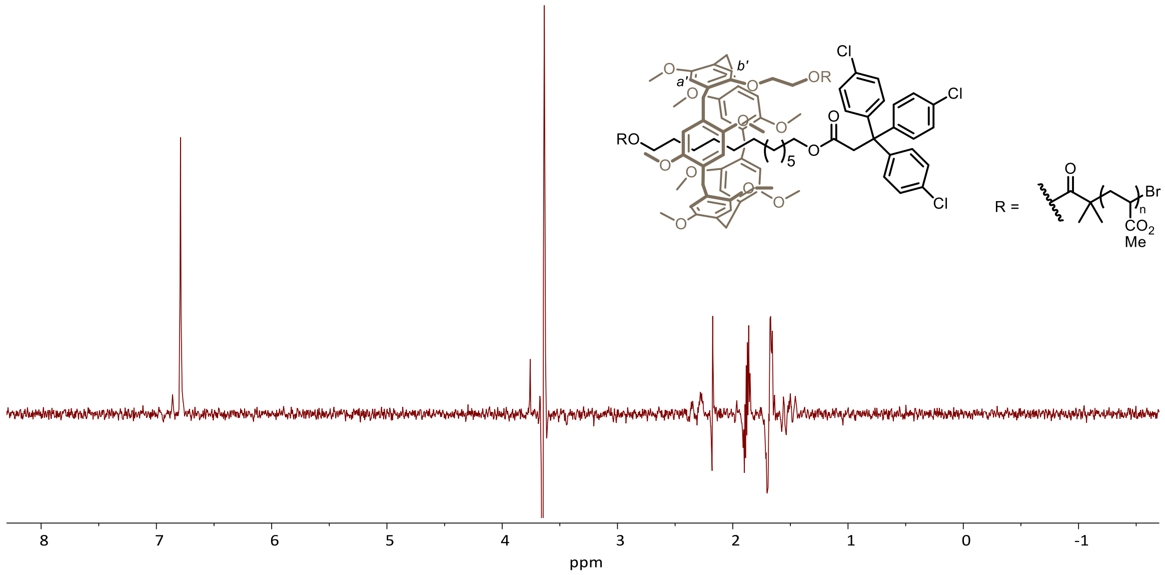

Spectrum S50. 1D selective NOESY ^{1}H NMR (700 MHz, Acetonitrile-d_6, 298 K) spectrum of post-sonication polymer $\mathbf{1}_{cis\text{-OMe-145}}$ after being washed with methanol upon selective excitation of proton $H_{a'}$.

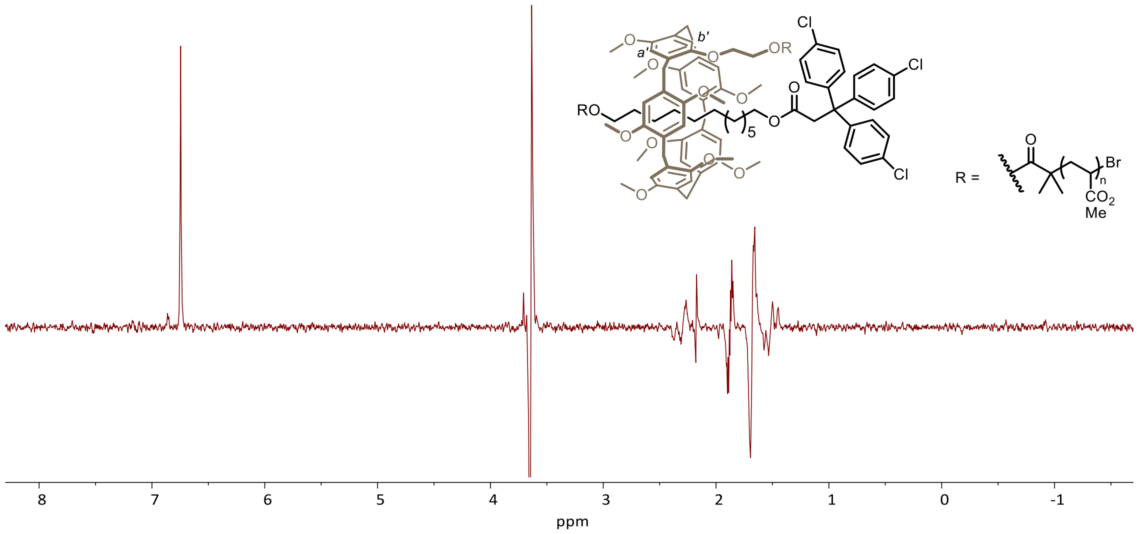

Spectrum S51. 1D selective NOESY ^{1}H NMR (700 MHz, Acetonitrile-d_6, 298 K) spectrum of post-sonication polymer $\mathbf{1}_{\mathit{cis}\text{-OMe-145}}$ after being washed with methanol upon selective excitation of proton $H_{b'}$.

8.3.4 Post-Sonication ^{1}H NMR Spectra of Polymer $\mathbf{1}_{\mathit{cis}\text{-OMe-145}}$ (Run 3)

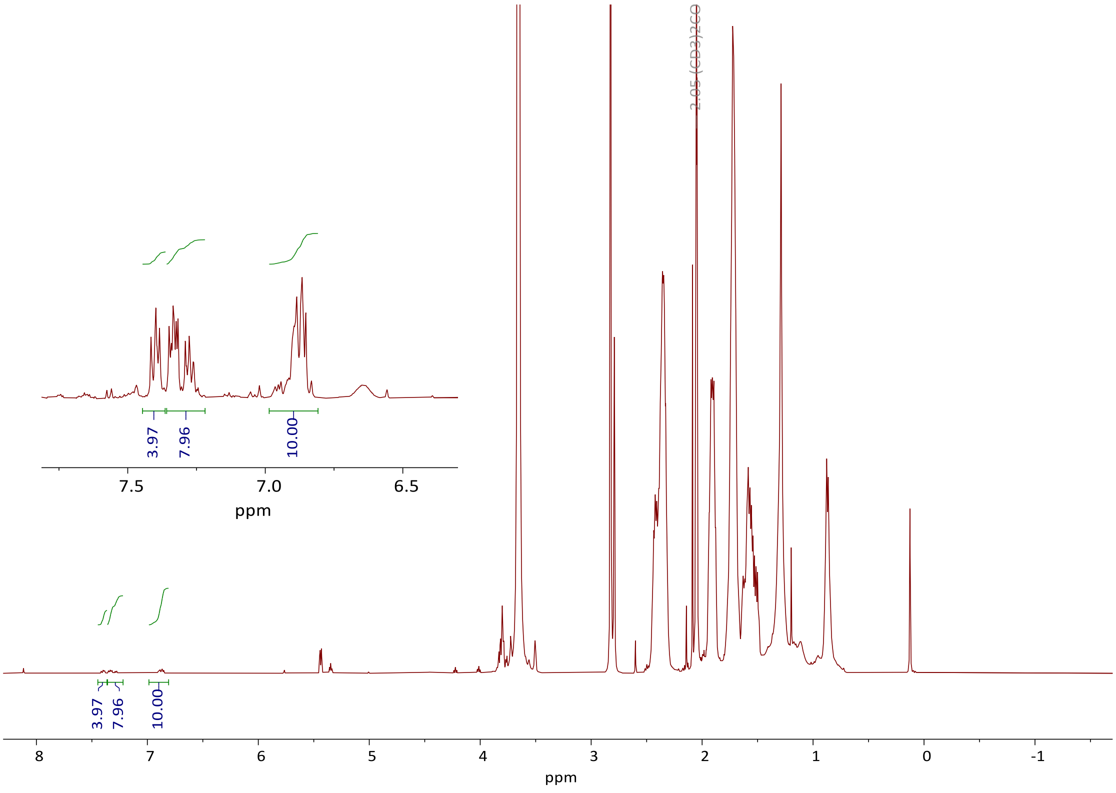

Spectrum S52. ^{1}H NMR (400 MHz, Acetone-d_6, 298 K) spectrum of post-sonication polymer $\mathbf{1}_{\mathit{cis}\text{-OMe-145}}$ before being washed with methanol.

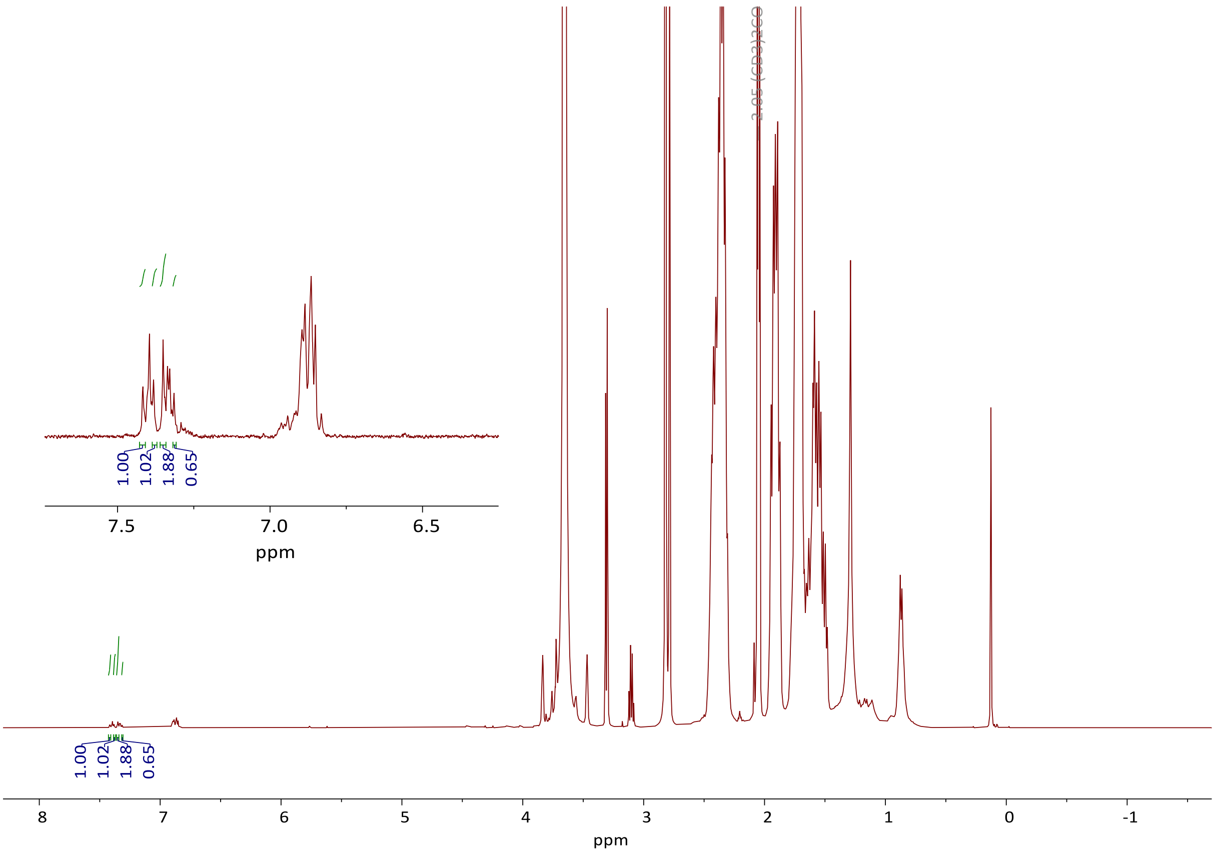

Spectrum S53. ^{1}H NMR (400 MHz, Acetone-d_6, 298 K) spectrum of post-sonication polymer $\mathbf{1}_{cis\text{-OMe-145}}$ after being washed with methanol.

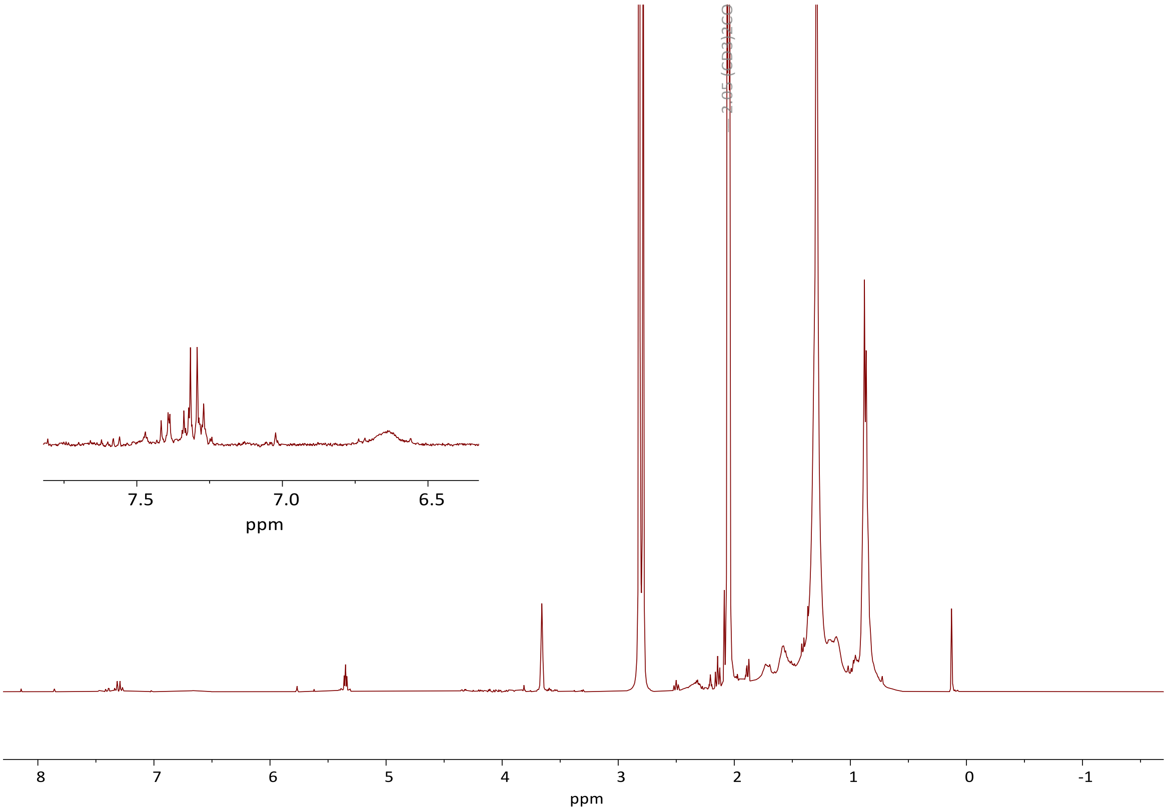

Spectrum S54. ^{1}H NMR (400 MHz, Acetone-d_6, 298 K) spectrum of the MeOH extract from post-sonication polymer $\mathbf{1}_{cis\text{-OMe-145}}$.

8.3.5 Post-Sonication ^{1}H NMR Spectra of Polymer $\mathbf{1}_{cis\text{-OMe-145}}$ (2 min sonication, Run 1)

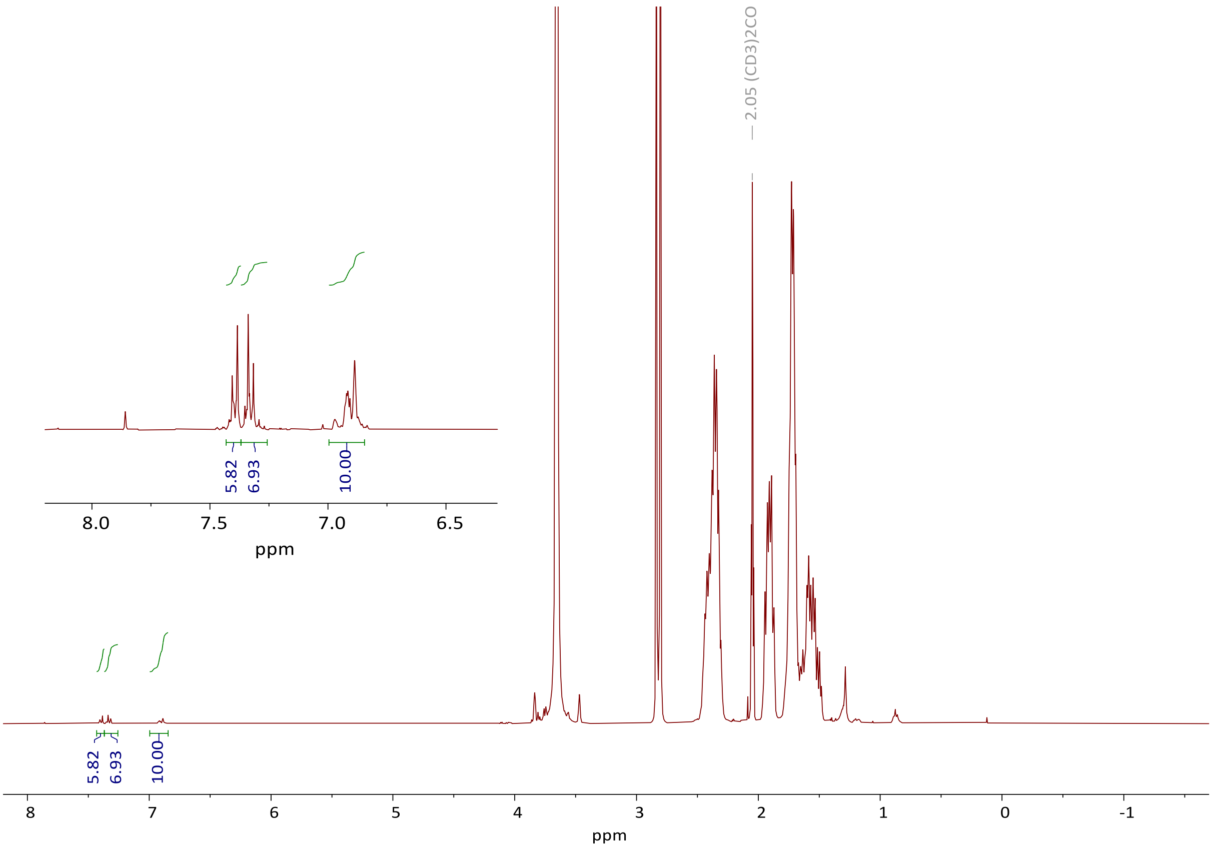

Spectrum S55. ^{1}H NMR (400 MHz, Acetone-d_6, 298 K) spectrum of post-sonication polymer $\mathbf{1}_{\mathit{cis}\text{-OMe-145}}$ before being washed with methanol.

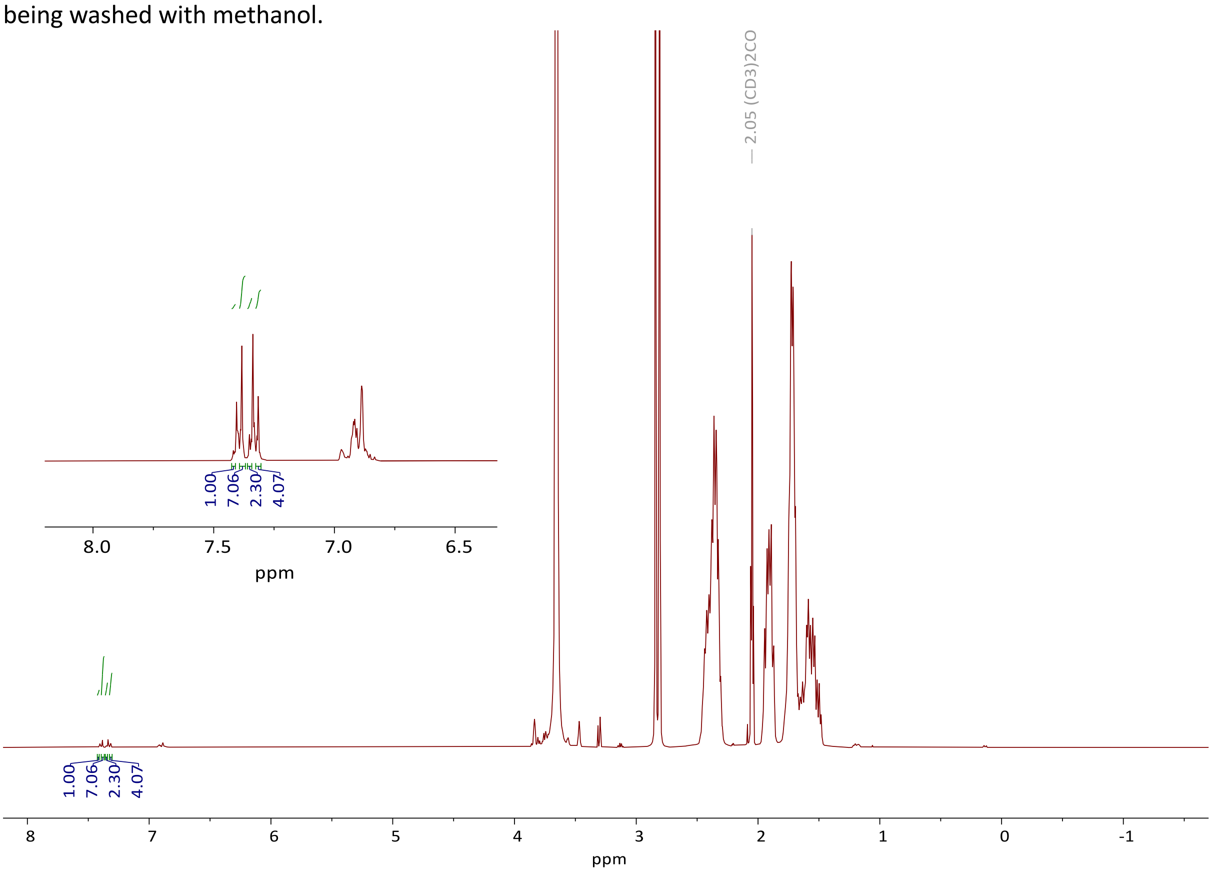

Spectrum S56. ^{1}H NMR (400 MHz, Acetone-d_6, 298 K) spectrum of post-sonication polymer $\mathbf{1}_{\mathit{cis}\text{-OMe-145}}$ after being washed with methanol.

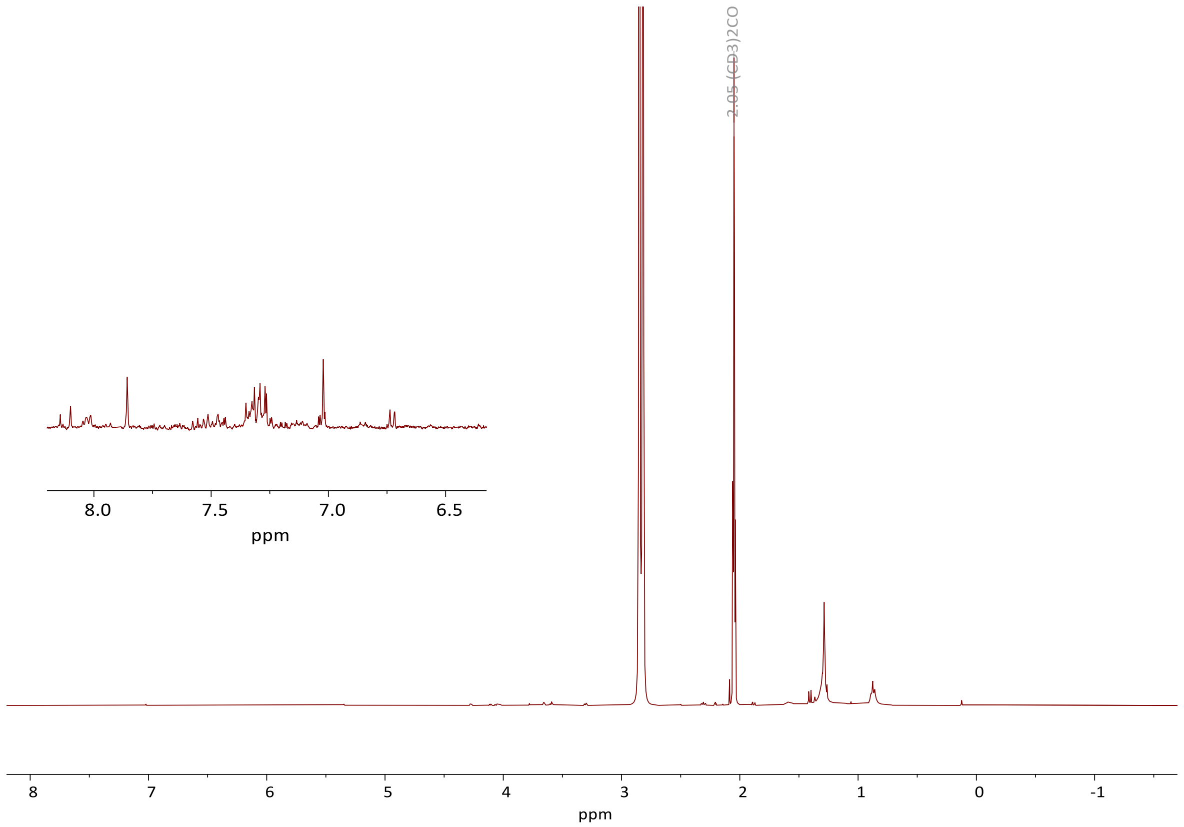

Spectrum S57. ^{1}H NMR (400 MHz, Acetone-d_6, 298 K) spectrum of the MeOH extract from post-sonication polymer $\mathbf{1}_{\mathit{cis}\text{-OMe-145}}$.

8.3.6 Post-Sonication ^{1}H NMR Spectra of Polymer $\mathbf{1}_{\mathit{cis}\text{-OMe-145}}$ (2 min sonication, Run 2)

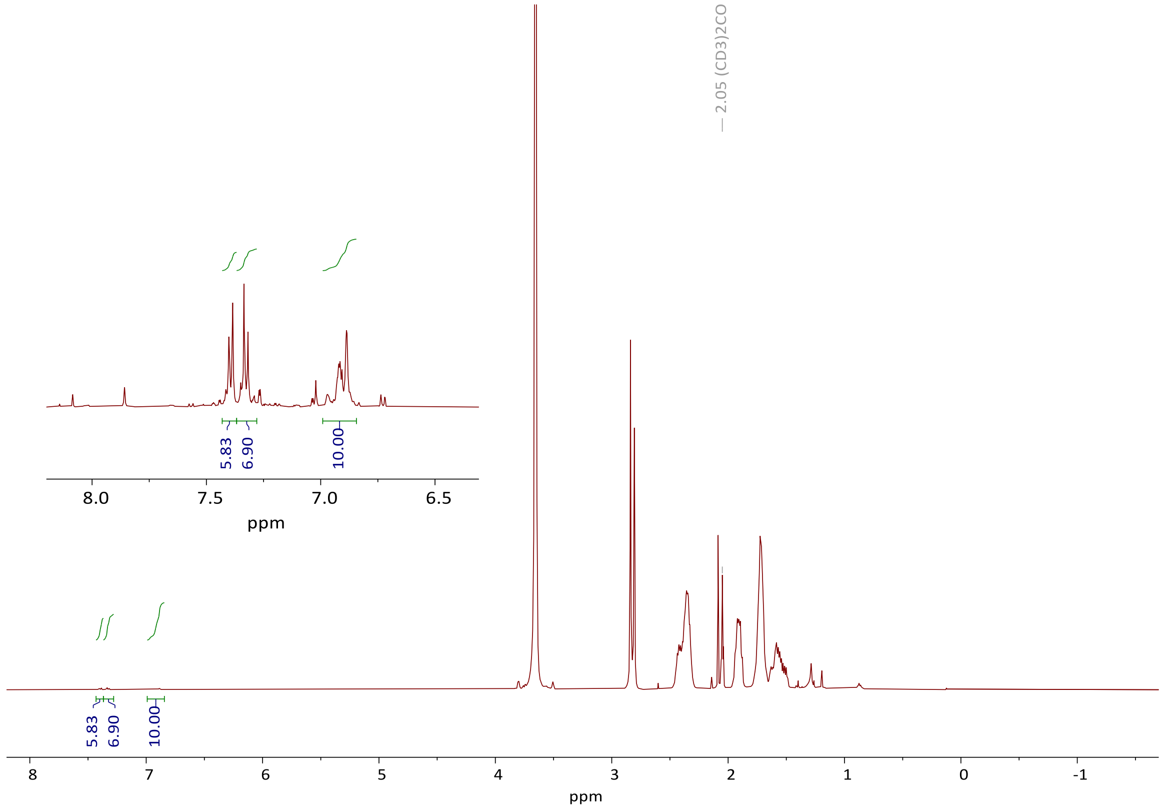

Spectrum S58. ^{1}H NMR (400 MHz, Acetone-d_6, 298 K) spectrum of post-sonication polymer $\mathbf{1}_{\mathit{cis}\text{-OMe-145}}$ before being washed with methanol.

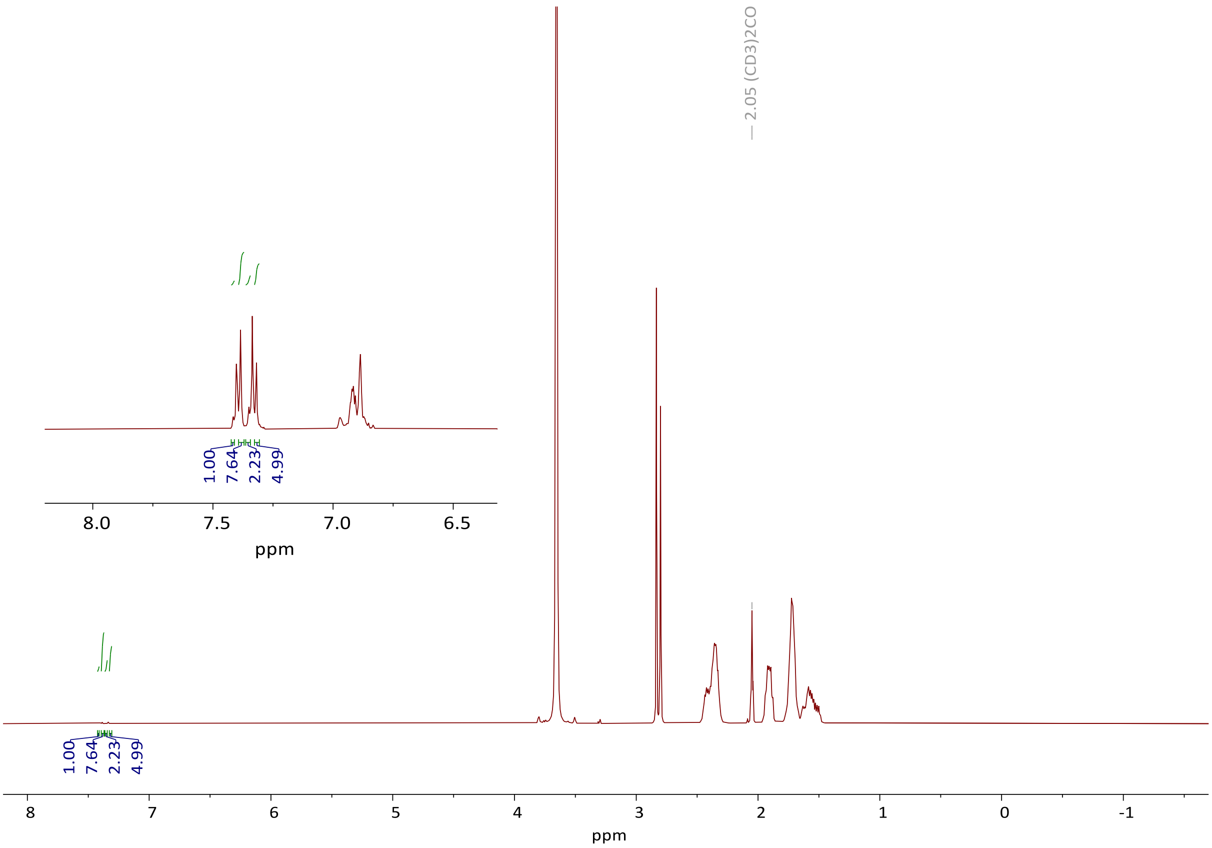

Spectrum S59. ^{1}H NMR (400 MHz, Acetone-d_6, 298 K) spectrum of post-sonication polymer $\mathbf{1}_{\textit{cis}\text{-OMe-145}}$ after being washed with methanol.

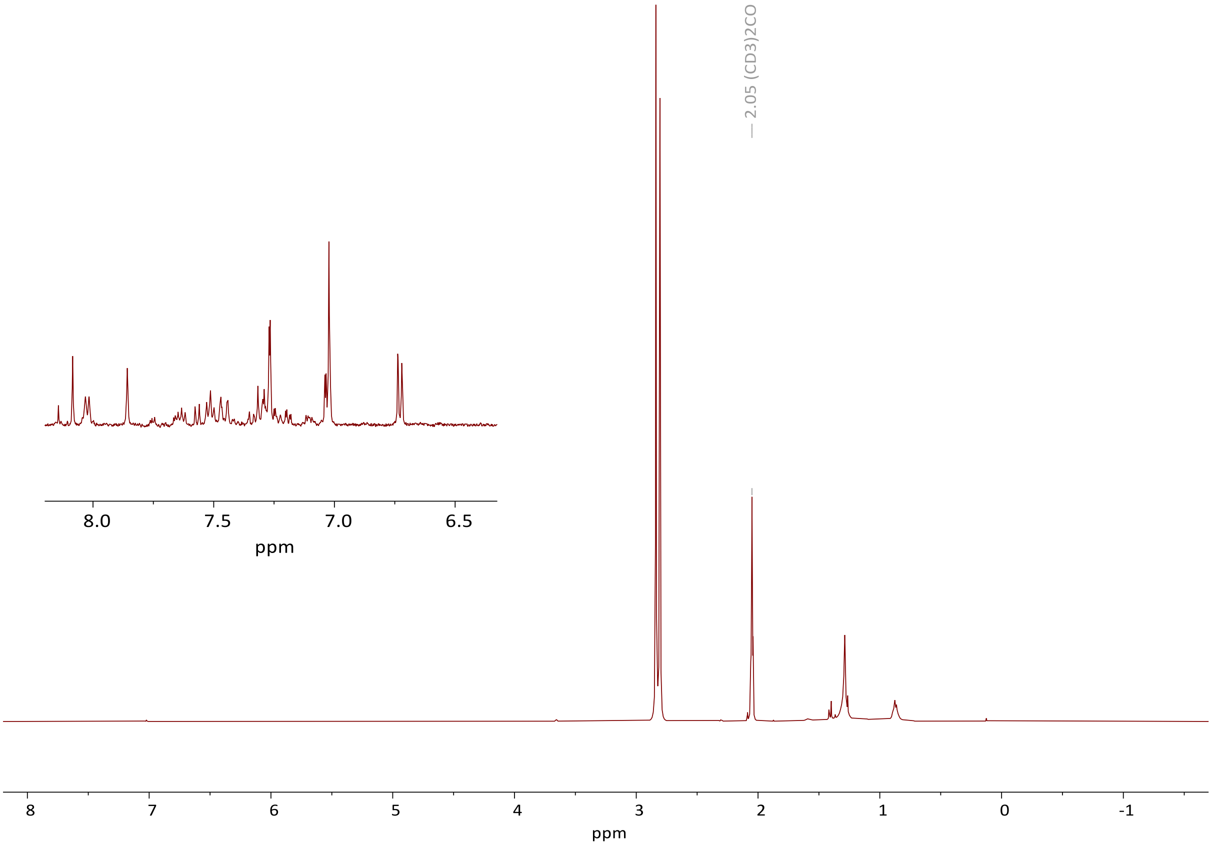

Spectrum S60. ^{1}H NMR (400 MHz, Acetone-d_6, 298 K) spectrum of the MeOH extract from post-sonication polymer $\mathbf{1}_{\textit{cis}\text{-OMe-145}}$.

8.3.7 Post-Sonication ^{1}H NMR Spectra of Polymer $\mathbf{1}_{cis\text{-}OMe\text{-}145}$ (5 min sonication, Run 1)

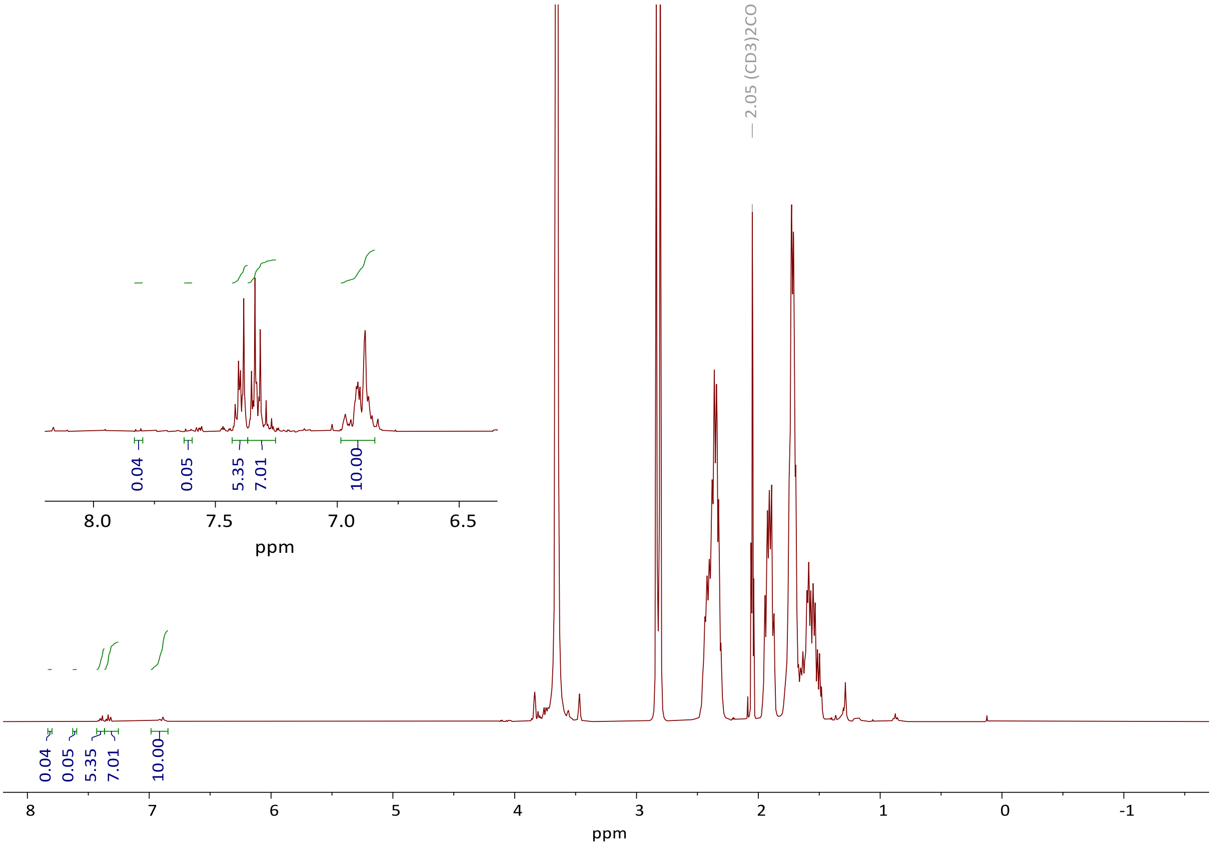

Spectrum S61. ^{1}H NMR (400 MHz, Acetone-d_6, 298 K) spectrum of post-sonication polymer $\mathbf{1}_{cis\text{-}OMe\text{-}145}$ before being washed with methanol.

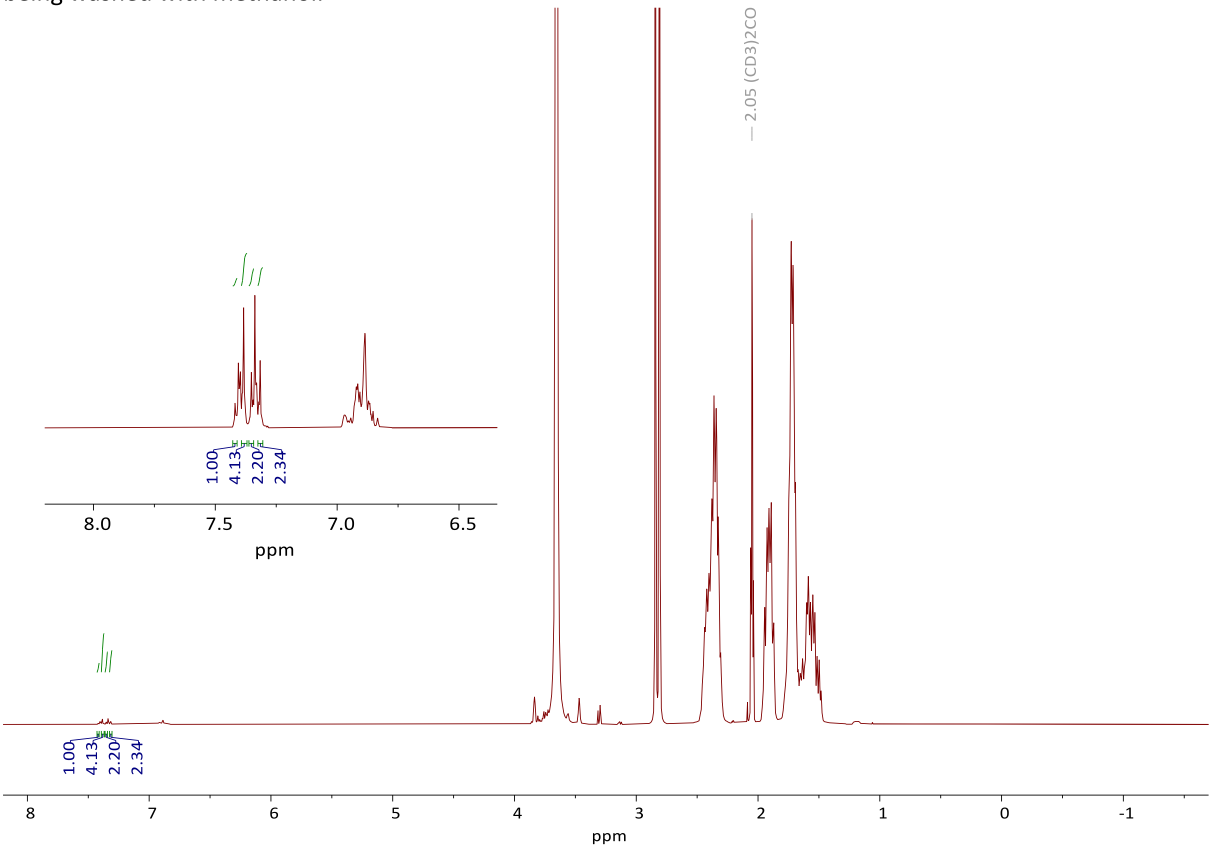

Spectrum S62. ^{1}H NMR (400 MHz, Acetone-d_6, 298 K) spectrum of post-sonication polymer $\mathbf{1}_{cis\text{-}OMe\text{-}145}$ after being washed with methanol.

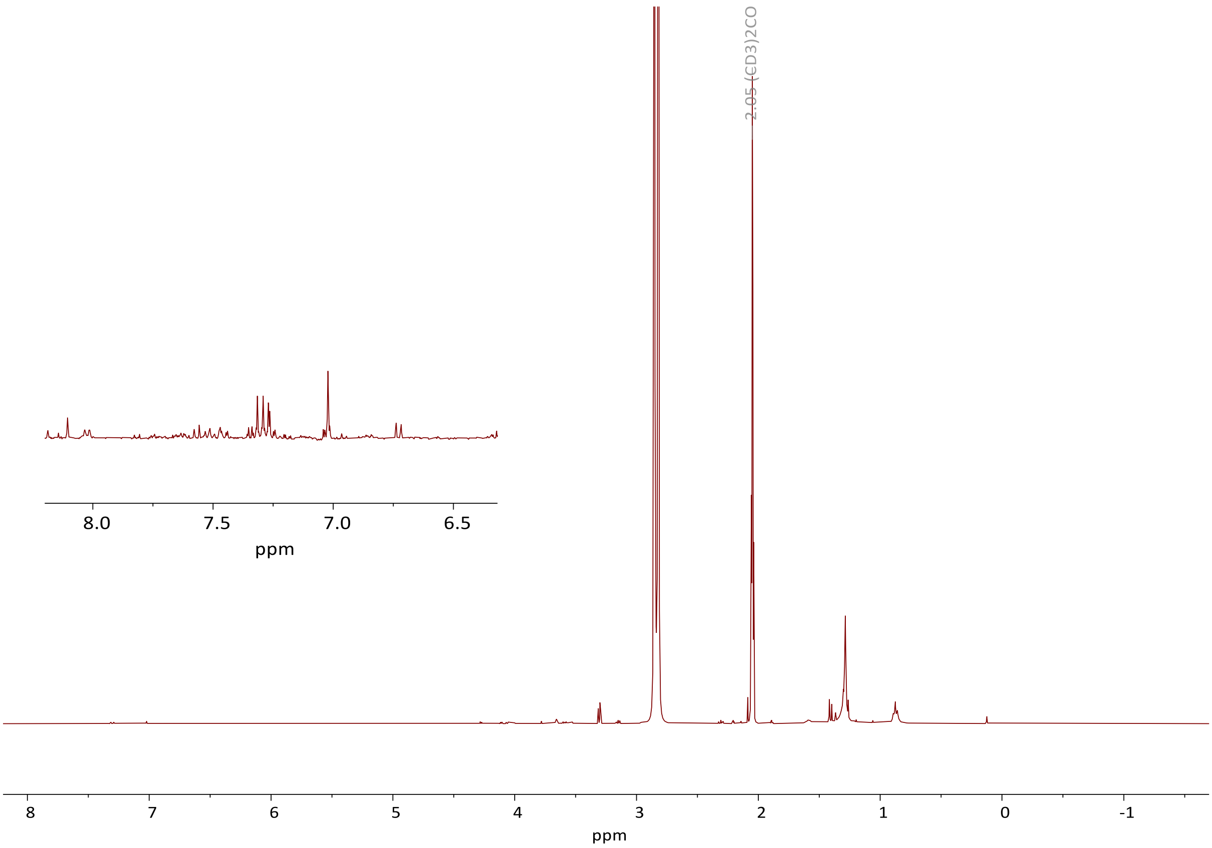

Spectrum S63. ^1H NMR (400 MHz, Acetone-d_6, 298 K) spectrum of the MeOH extract from post-sonication polymer $\mathbf{1}_{\textit{cis}\text{-OMe-145}}$.

8.3.8 Post-Sonication ^1H NMR Spectra of Polymer $\mathbf{1}_{\textit{cis}\text{-OMe-145}}$ (5 min sonication, Run 2)

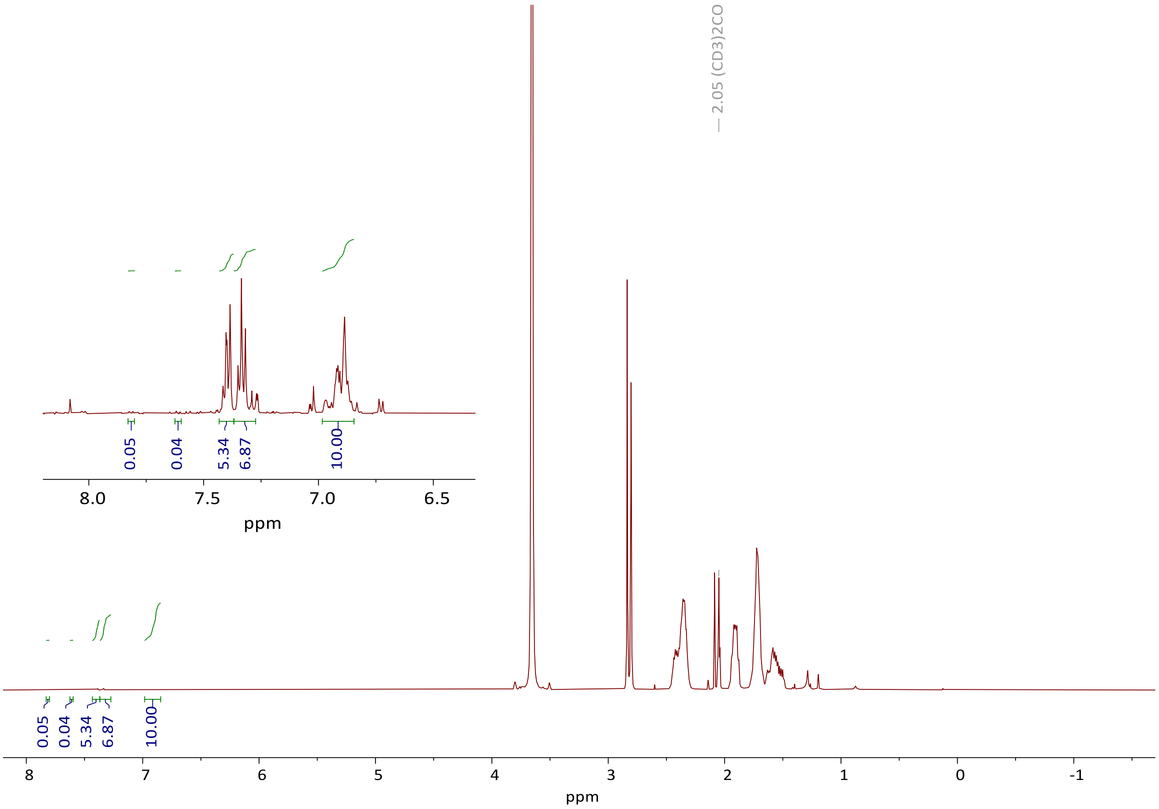

Spectrum S64. ^1H NMR (400 MHz, Acetone-d_6, 298 K) spectrum of post-sonication polymer $\mathbf{1}_{\textit{cis}\text{-OMe-145}}$ before being washed with methanol.

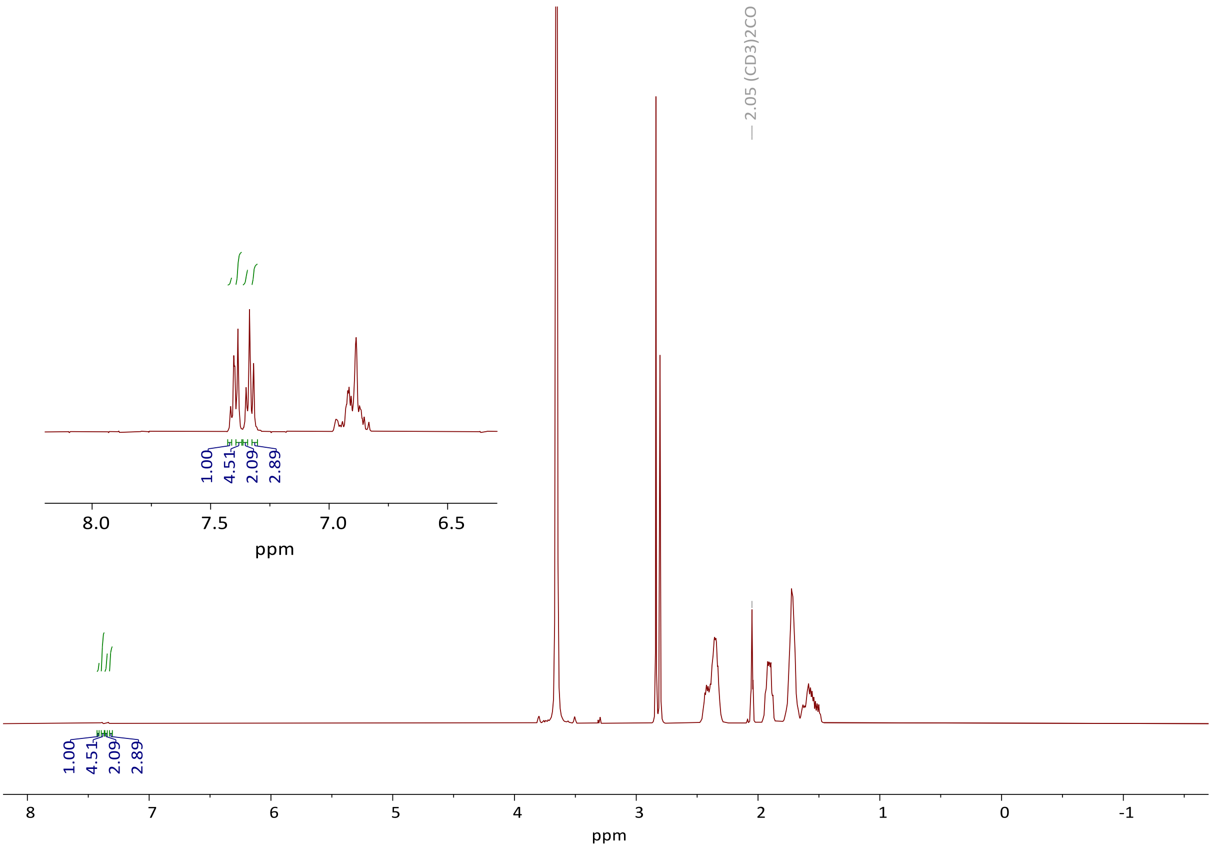

Spectrum S65. ^{1}H NMR (400 MHz, Acetone-d_6, 298 K) spectrum of post-sonication polymer $\mathbf{1}_{cis\text{-}\mathbf{OMe\text{-}145}}$ after being washed with methanol.

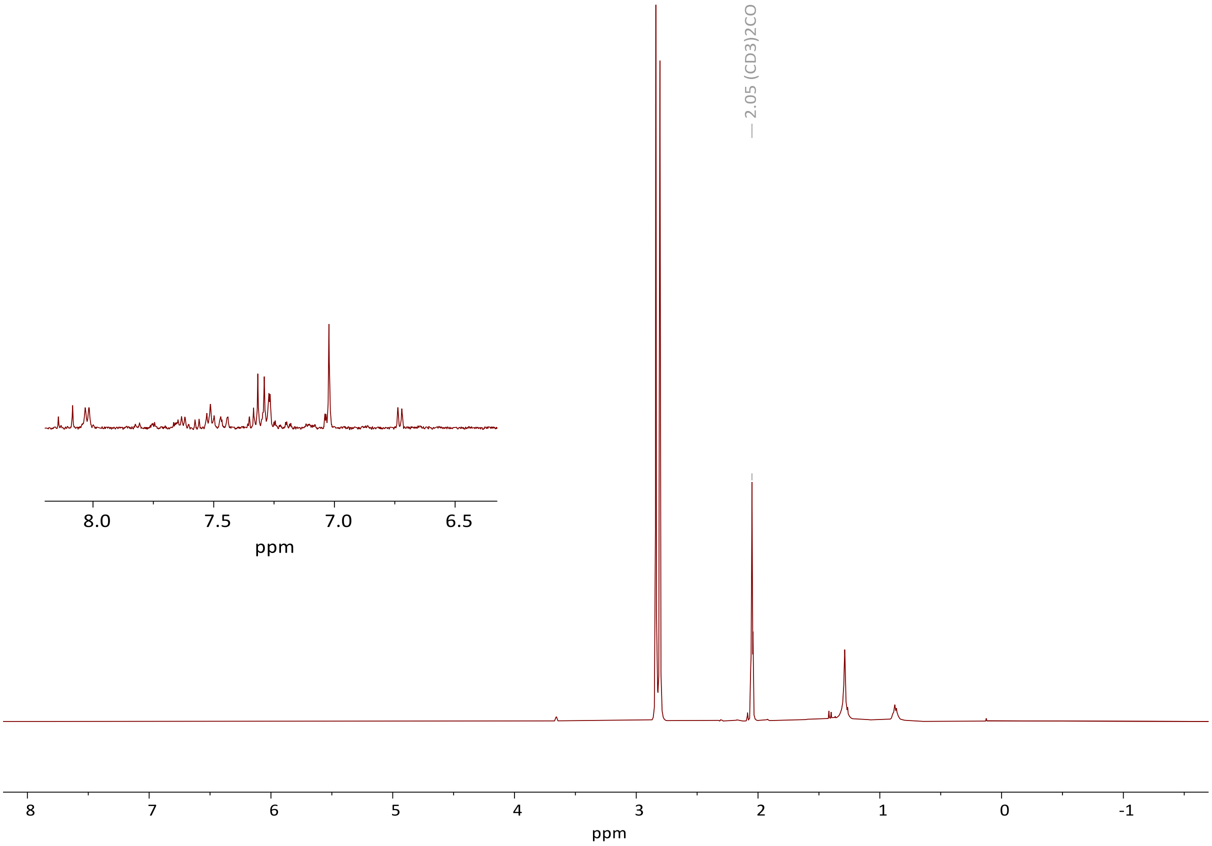

Spectrum S66. ^{1}H NMR (400 MHz, Acetone-d_6, 298 K) spectrum of the MeOH extract from post-sonication polymer $\mathbf{1}_{cis\text{-}\mathbf{OMe\text{-}145}}$.

8.3.9 Post-Sonication ^{1}H NMR Spectra of Polymer $1_{cis\text{-OMe-145}}$ (10 min sonication, Run 1)

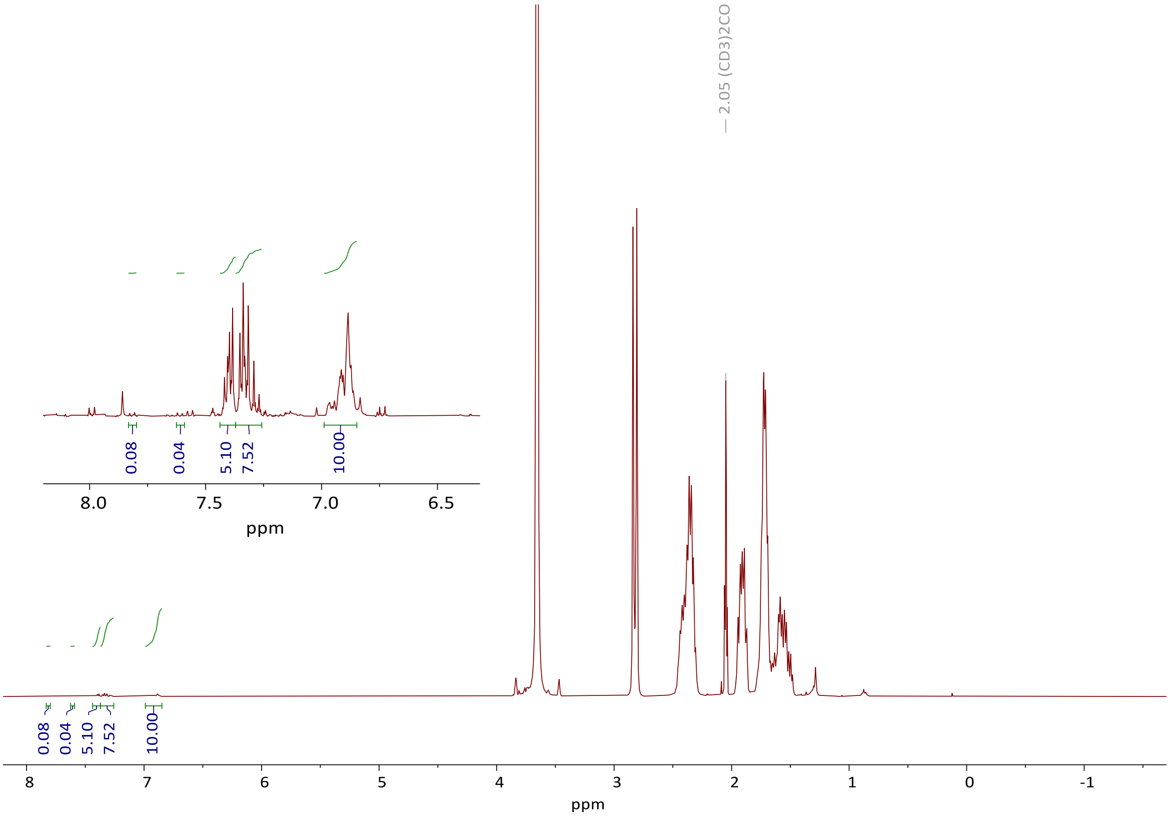

Spectrum S67. ^{1}H NMR (400 MHz, Acetone-d_6, 298 K) spectrum of post-sonication polymer $\mathbf{1}_{cis\text{-OMe-145}}$ before being washed with methanol.

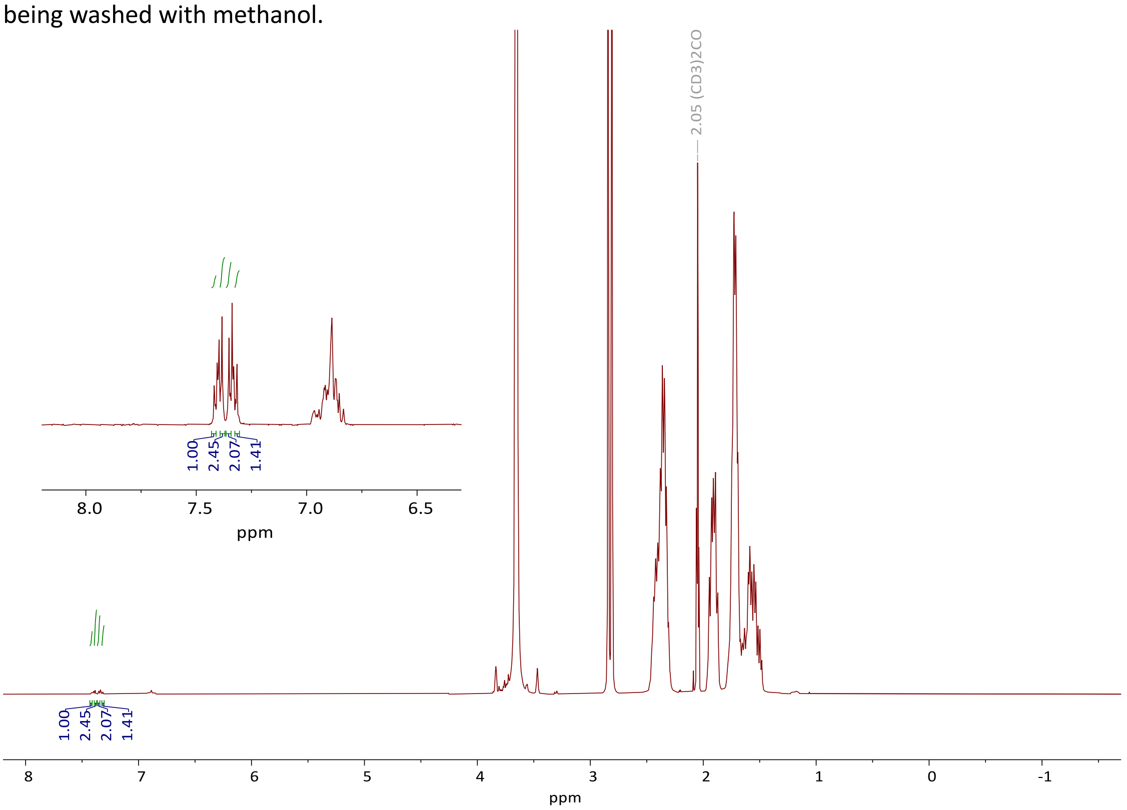

Spectrum S68. ^{1}H NMR (400 MHz, Acetone-d_6, 298 K) spectrum of post-sonication polymer $\mathbf{1}_{cis\text{-OMe-145}}$ after being washed with methanol.

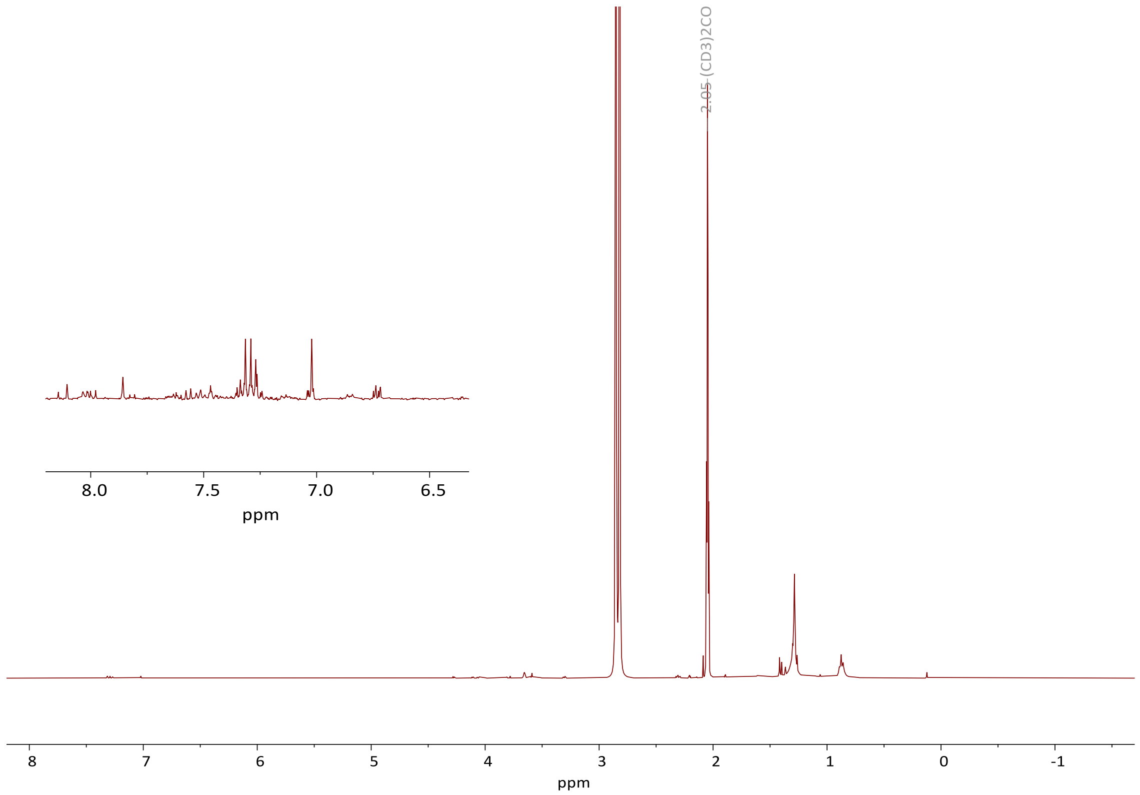

Spectrum S69. ^{1}H NMR (400 MHz, Acetone-d_6, 298 K) spectrum of the MeOH extract from post-sonication polymer **1**$_{\textit{cis}\text{-OMe-145}}$.

8.3.10 Post-Sonication ^{1}H NMR Spectra of Polymer 1$_{\textit{cis}\text{-OMe-145}}$ (10 min sonication, Run 2)

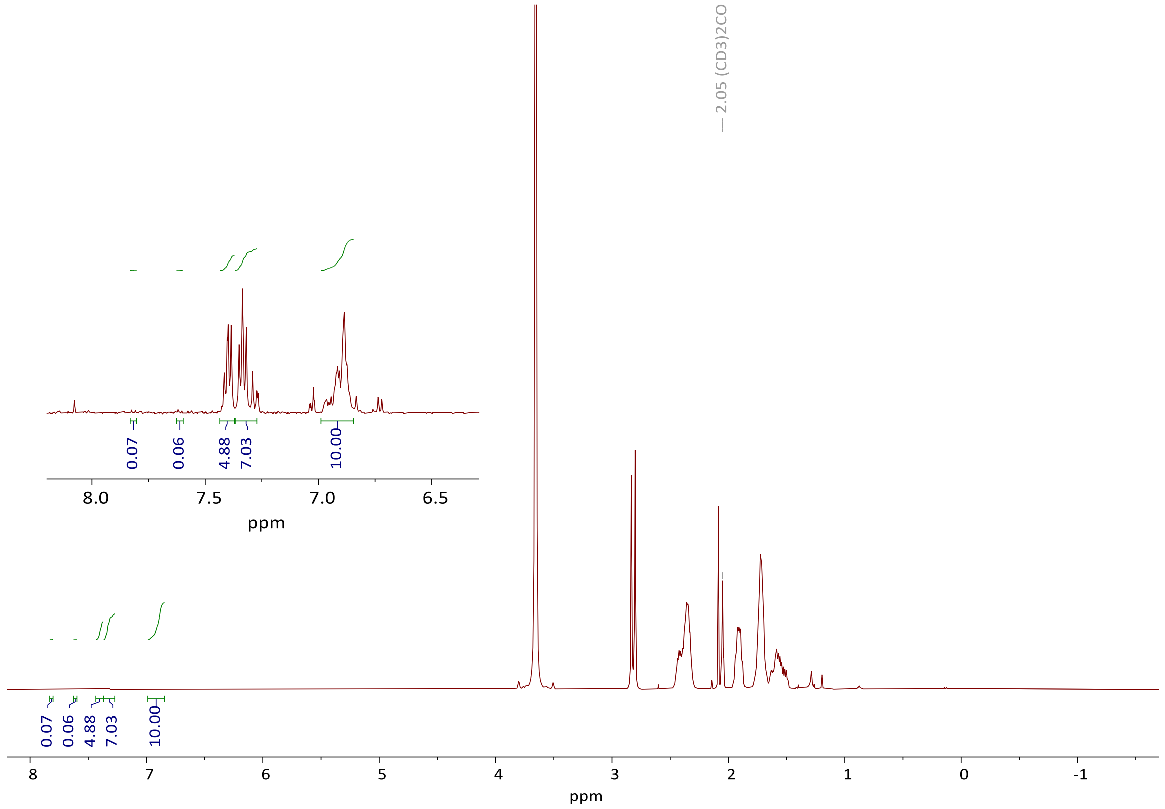

Spectrum S70. ^{1}H NMR (400 MHz, Acetone-d_6, 298 K) spectrum of post-sonication polymer **1**$_{\textit{cis}\text{-OMe-145}}$ before being washed with methanol.

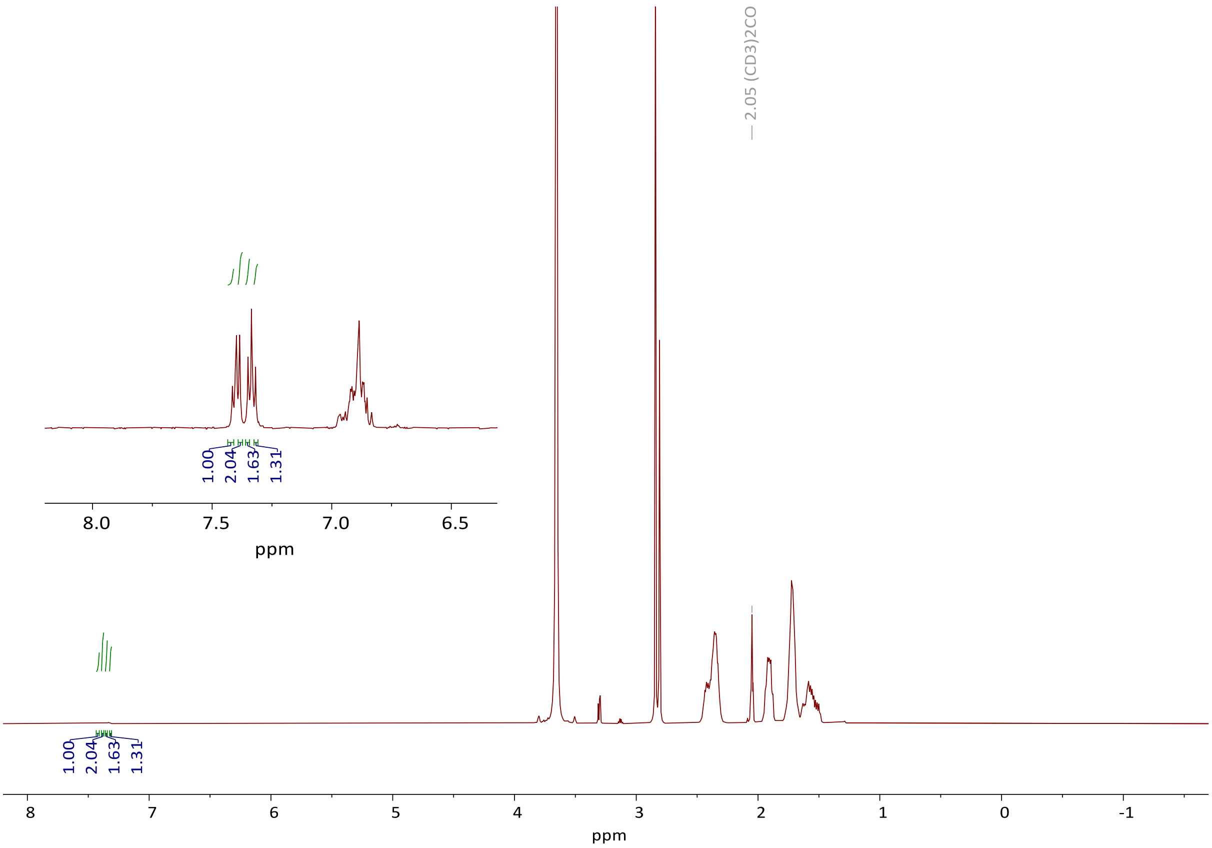

Spectrum S71. ^{1}H NMR (400 MHz, Acetone-d_6, 298 K) spectrum of post-sonication polymer $\mathbf{1}_{\textit{cis}\text{-OMe-145}}$ after being washed with methanol.

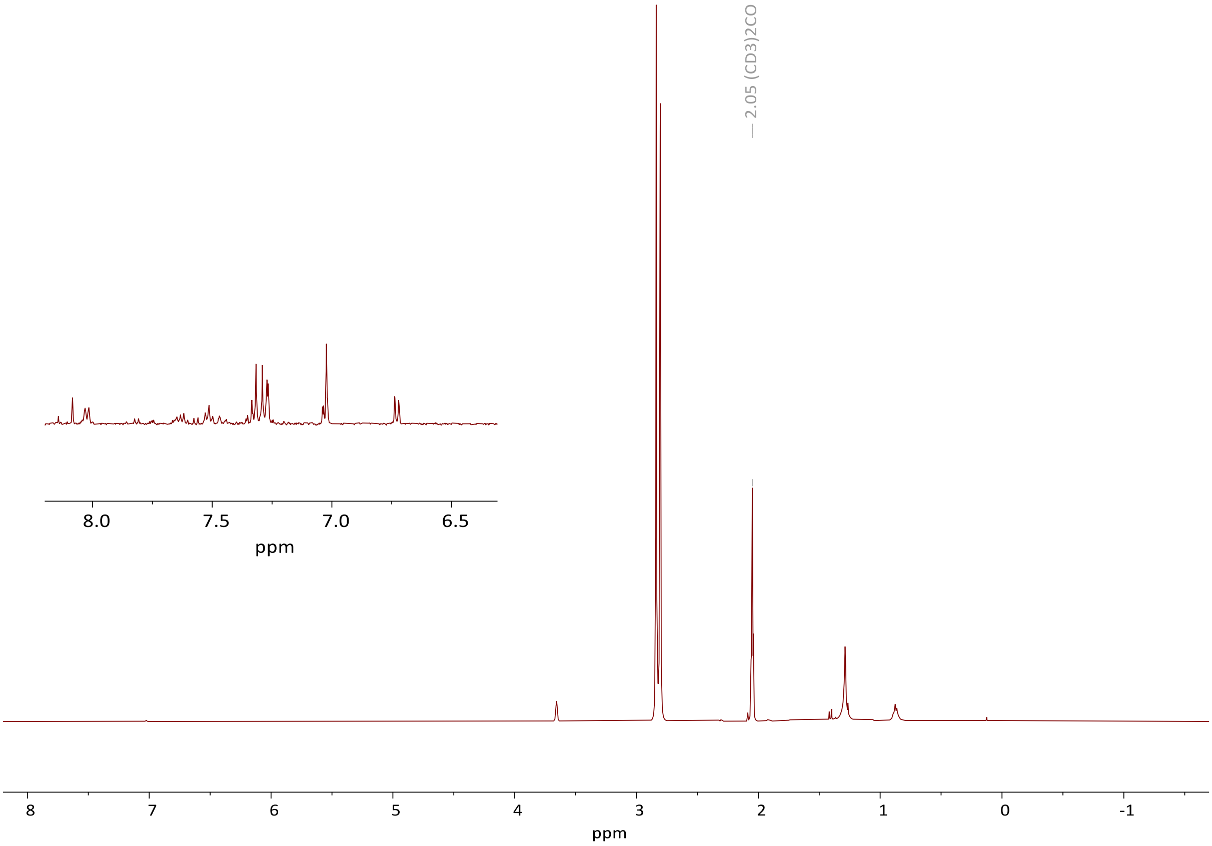

Spectrum S72. ^{1}H NMR (400 MHz, Acetone-d_6, 298 K) spectrum of the MeOH extract from post-sonication polymer $\mathbf{1}_{\textit{cis}\text{-OMe-145}}$.

8.3.11 Post-Sonication ^{1}H NMR Spectra of Polymer $\mathbf{1}_{cis\text{-OMe-145}}$ (20 min sonication, Run 1)

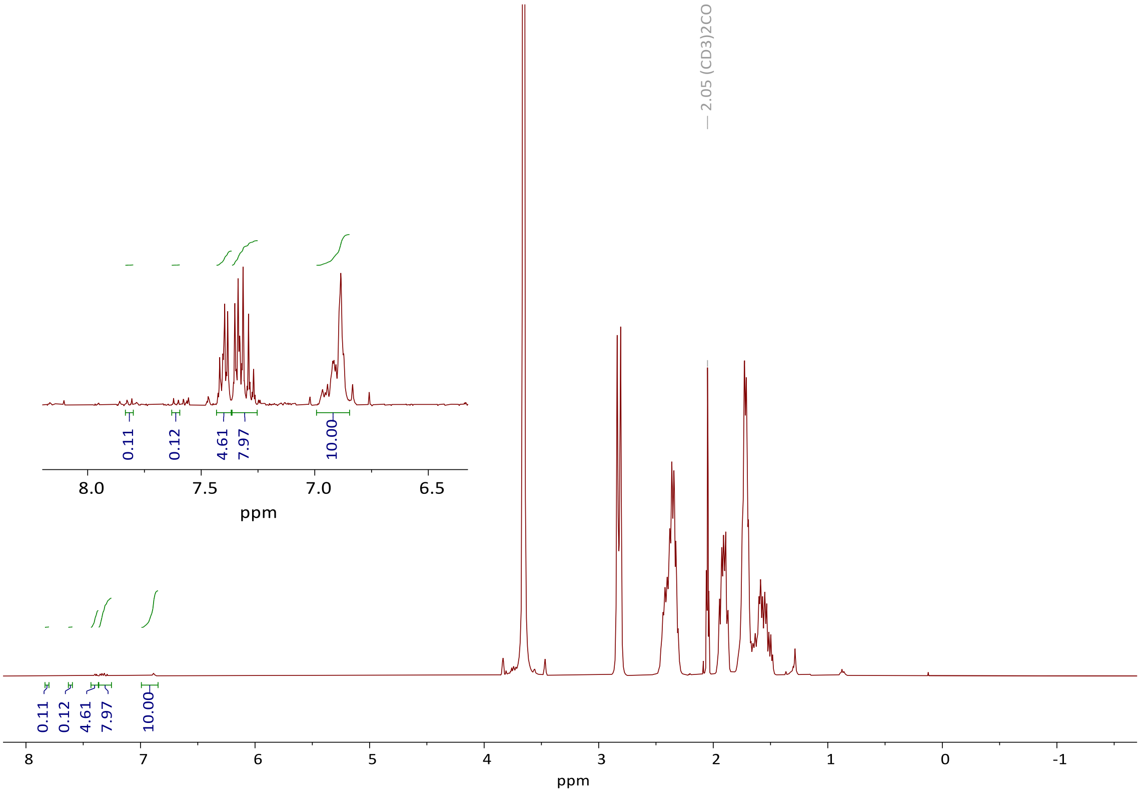

Spectrum S73. ^{1}H NMR (400 MHz, Acetone-d_6, 298 K) spectrum of post-sonication polymer $\mathbf{1}_{cis\text{-OMe-145}}$ before being washed with methanol.

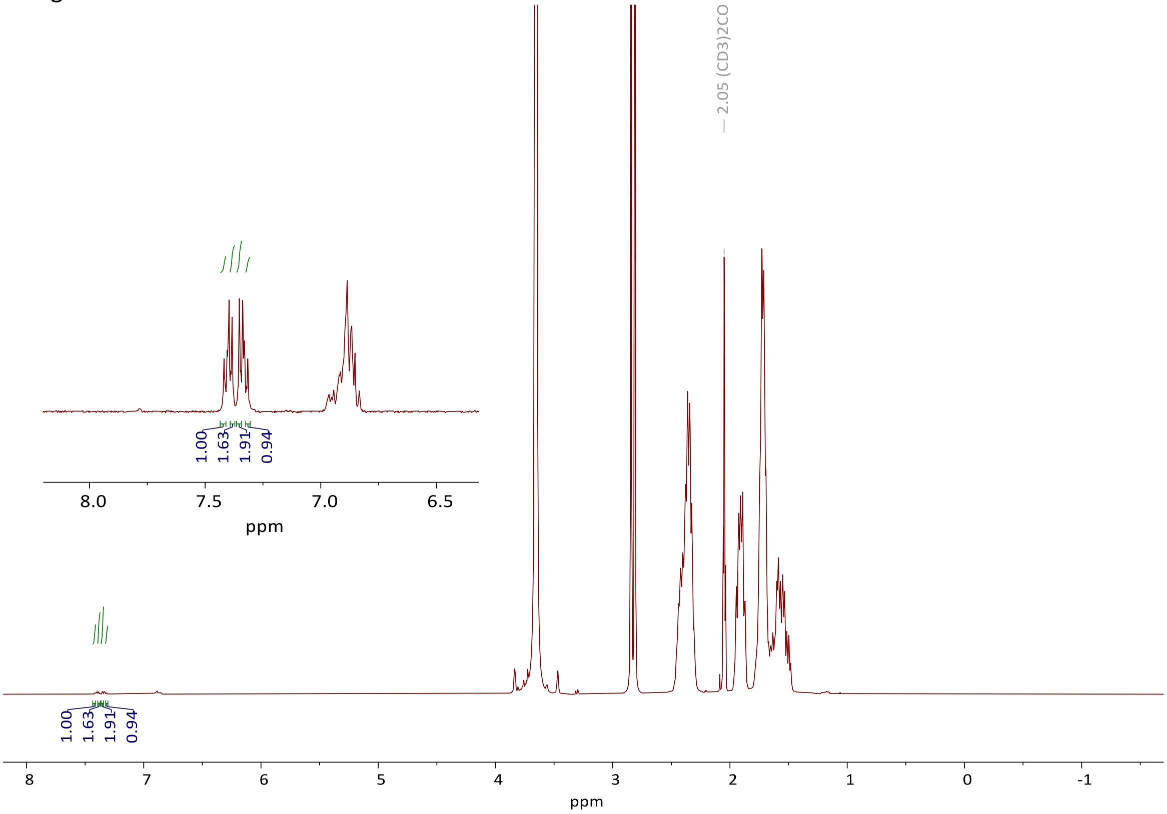

Spectrum S74. ^{1}H NMR (400 MHz, Acetone-d_6, 298 K) spectrum of post-sonication polymer $\mathbf{1}_{cis\text{-OMe-145}}$ after being washed with methanol.

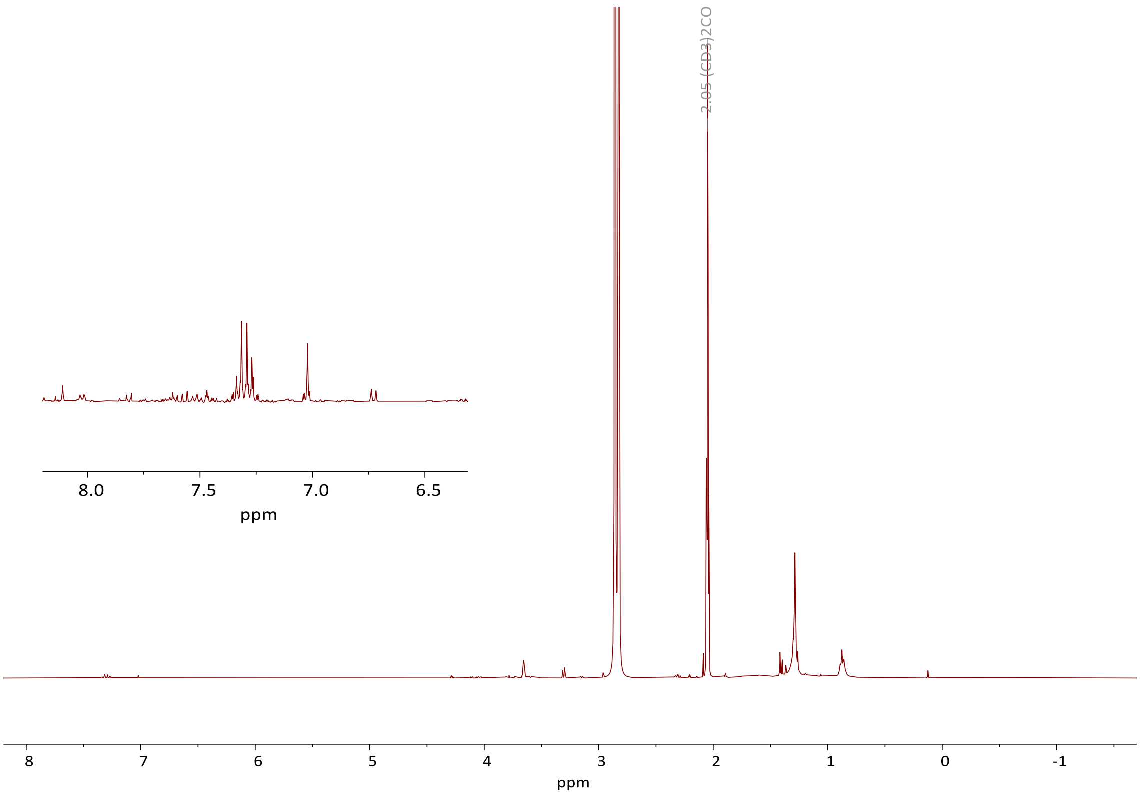

Spectrum S75. ^{1}H NMR (400 MHz, Acetone-d_6, 298 K) spectrum of the MeOH extract from post-sonication polymer $\mathbf{1}_{\textit{cis}\text{-OMe-145}}$.

8.3.12 Post-Sonication ^{1}H NMR Spectra of Polymer $\mathbf{1}_{\textit{cis}\text{-OMe-145}}$ (20 min sonication, Run 2)

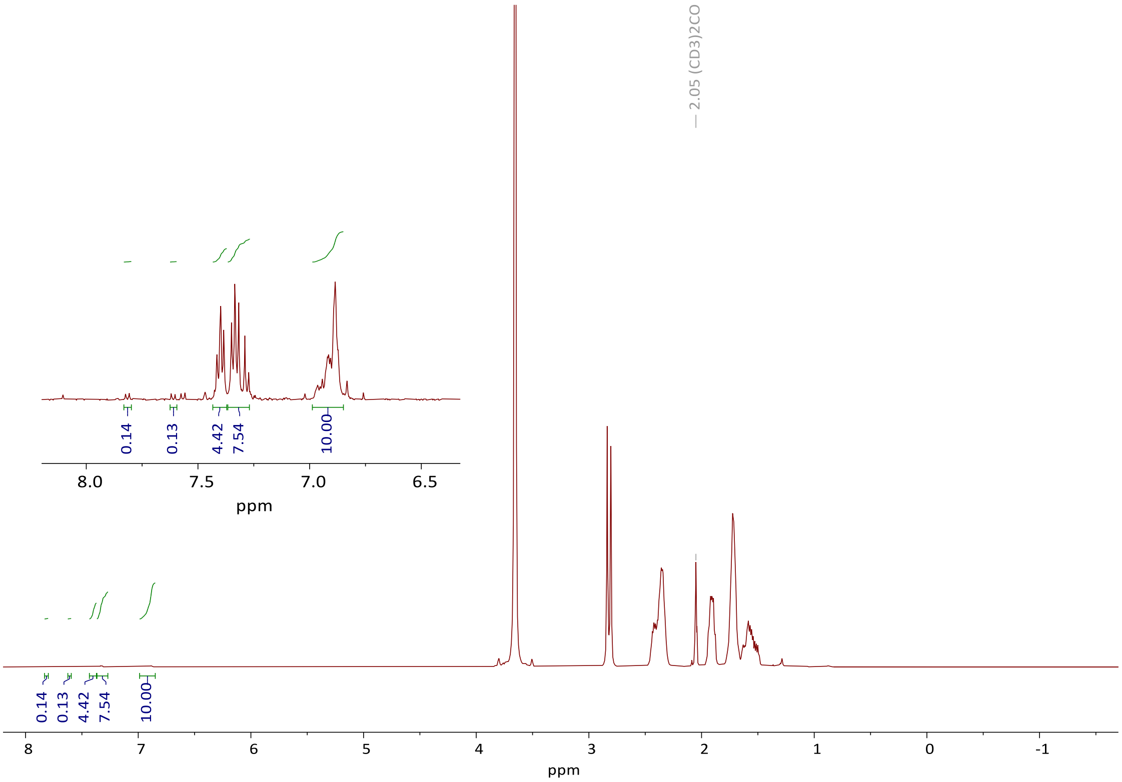

Spectrum S76. ^{1}H NMR (400 MHz, Acetone-d_6, 298 K) spectrum of post-sonication polymer $\mathbf{1}_{\textit{cis}\text{-OMe-145}}$ before being washed with methanol.

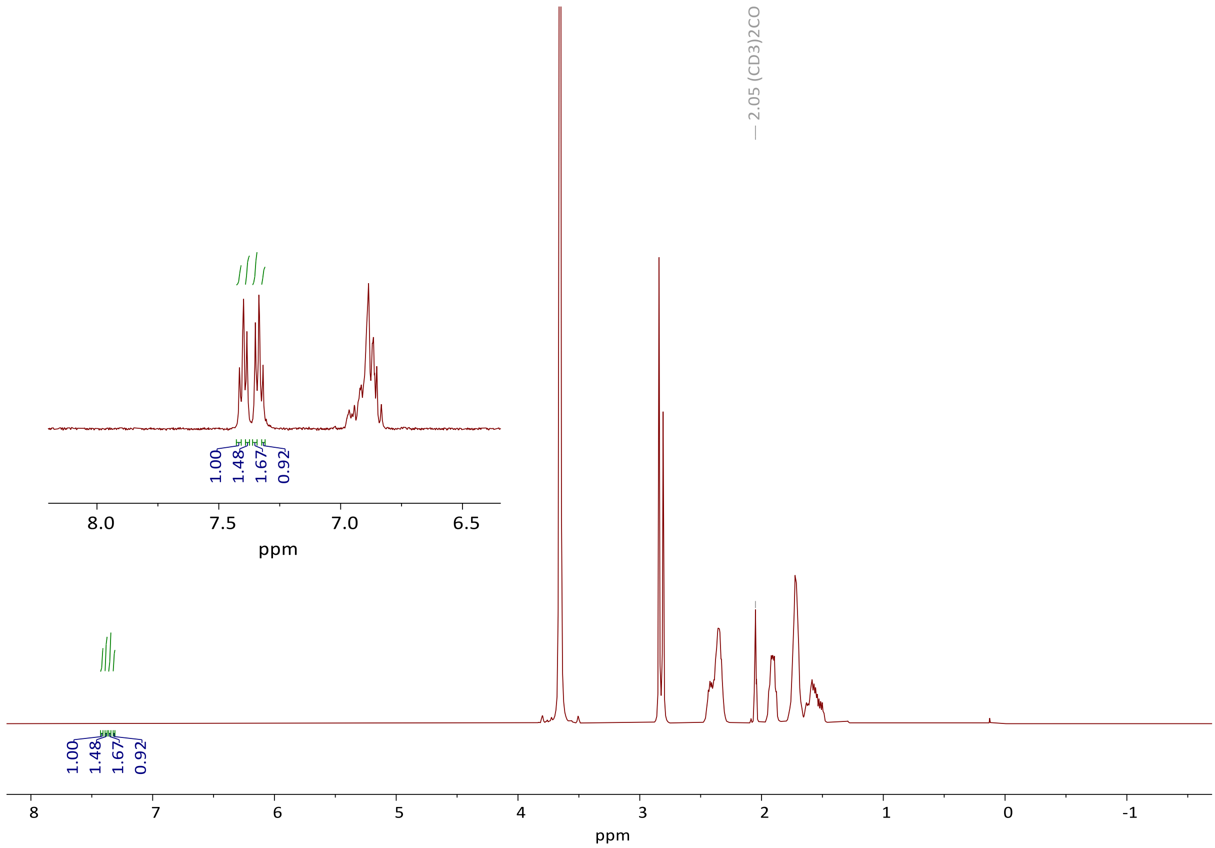

Spectrum S77. ^{1}H NMR (400 MHz, Acetone-d_6, 298 K) spectrum of post-sonication polymer $\mathbf{1}_{\textit{cis}\text{-OMe-145}}$ after being washed with methanol.

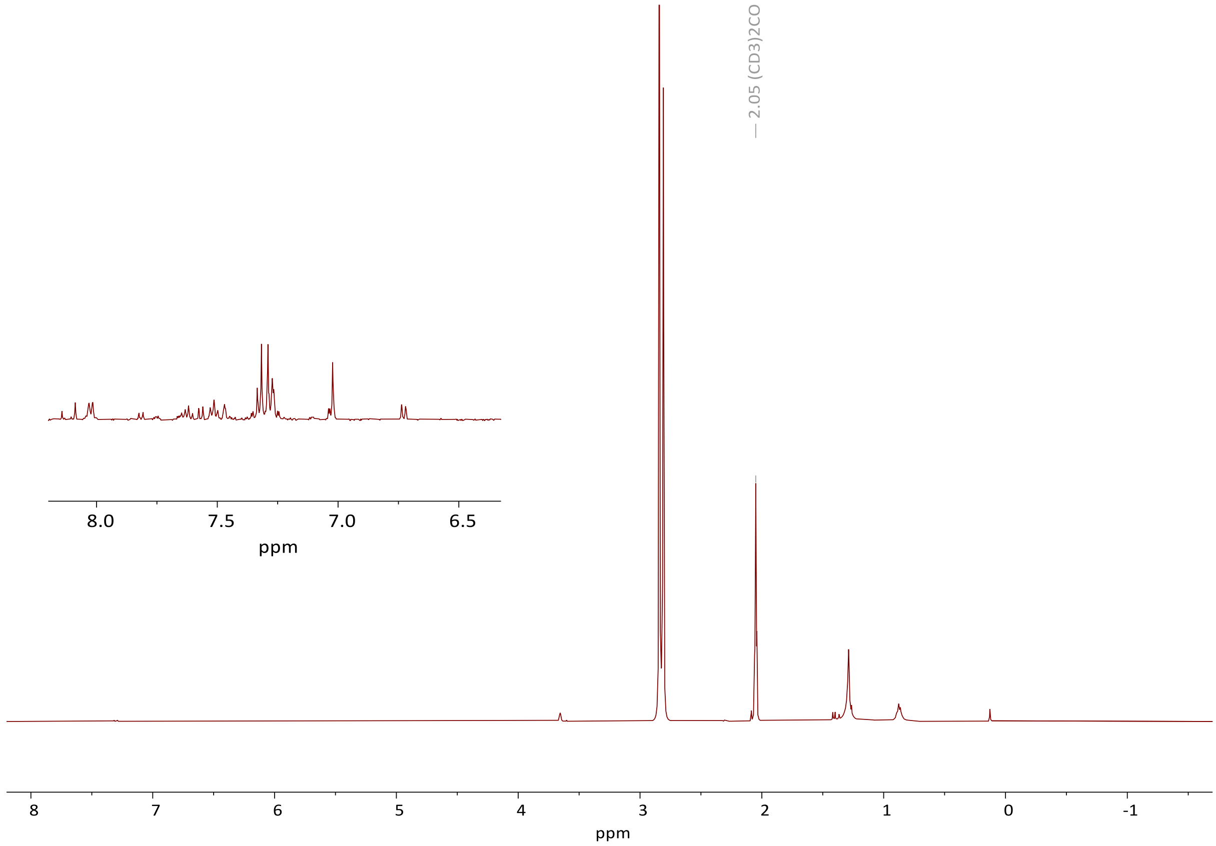

Spectrum S78. ^{1}H NMR (400 MHz, Acetone-d_6, 298 K) spectrum of the MeOH extract from post-sonication polymer $\mathbf{1}_{\textit{cis}\text{-OMe-145}}$.

8.3.13 Post-Sonication ^{1}H NMR Spectra of Polymer $\mathbf{1}_{cis\text{-}OMe\text{-}145}$ (40 min sonication, Run 1)

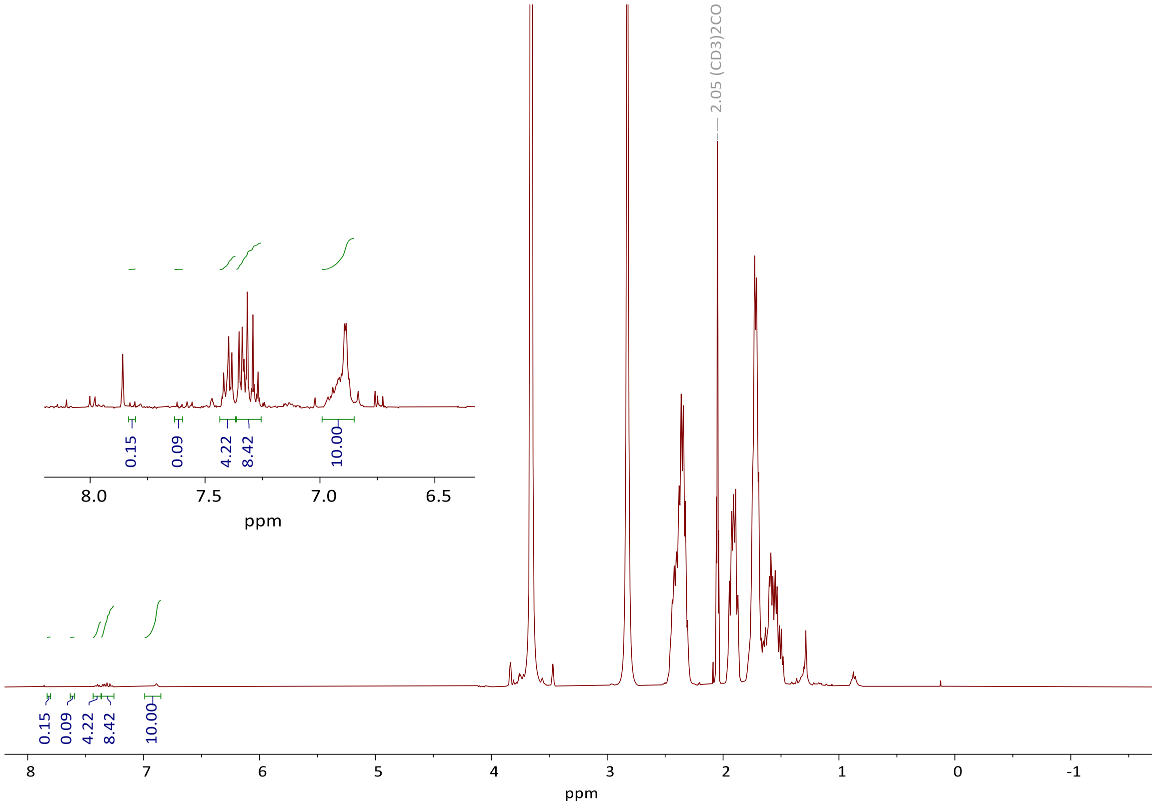

Spectrum S79. ^{1}H NMR (400 MHz, Acetone-d_6, 298 K) spectrum of post-sonication polymer $\mathbf{1}_{cis\text{-}OMe\text{-}145}$ before being washed with methanol.

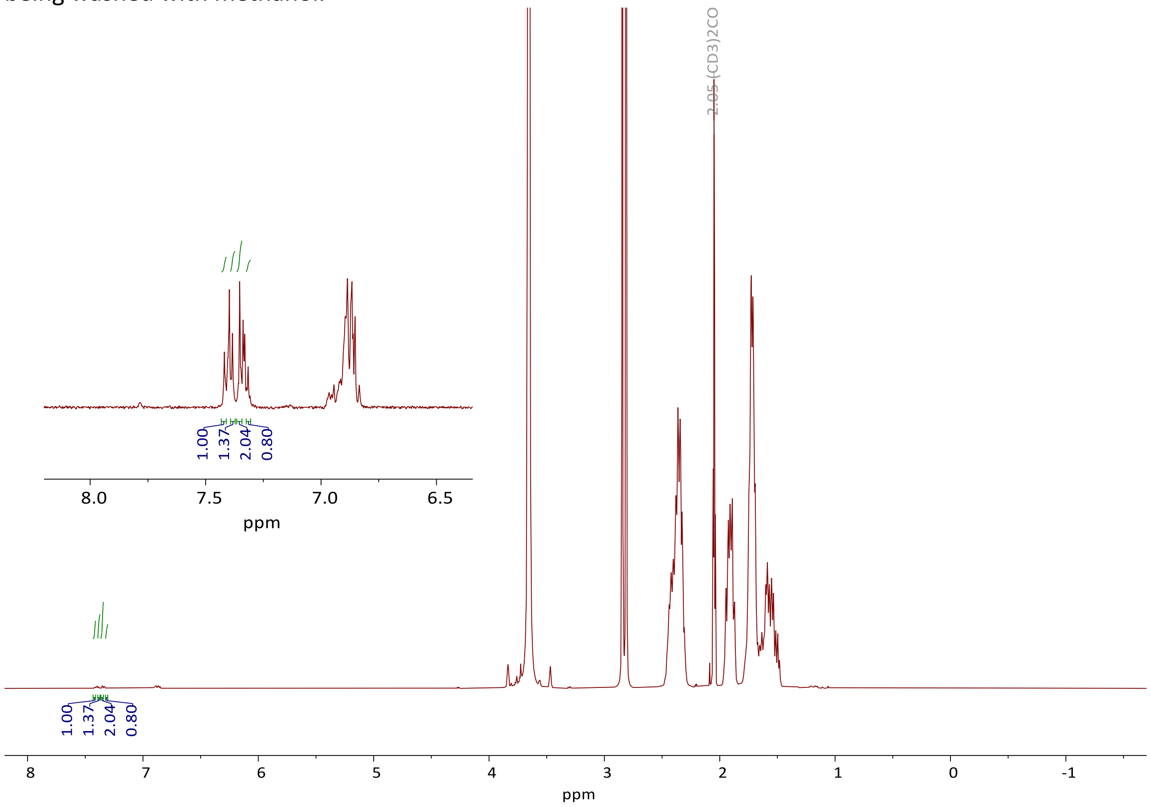

Spectrum S80. ^{1}H NMR (400 MHz, Acetone-d_6, 298 K) spectrum of post-sonication polymer $\mathbf{1}_{cis\text{-}OMe\text{-}145}$ after being washed with methanol.

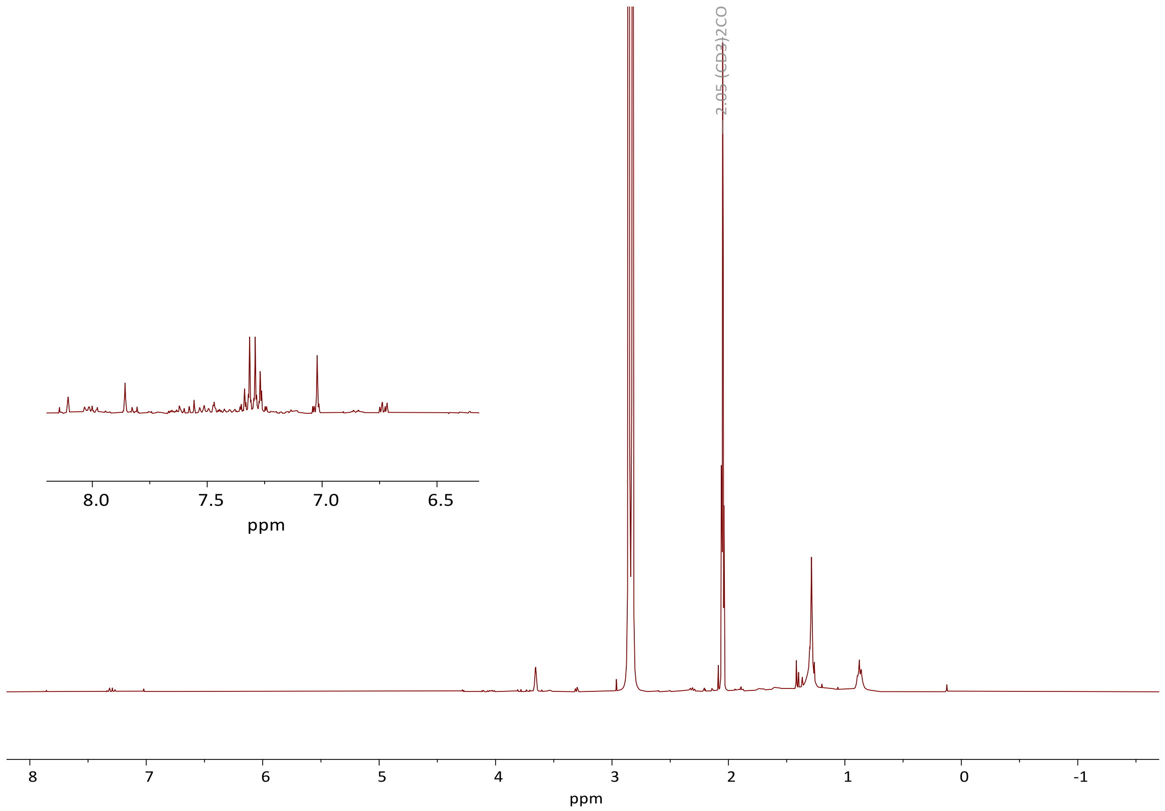

Spectrum S81. ^{1}H NMR (400 MHz, Acetone-d_6, 298 K) spectrum of the MeOH extract from post-sonication polymer **1**$_{\textbf{\textit{cis}}\textbf{-OMe-145}}$.

8.3.14 Post-Sonication ^{1}H NMR Spectra of Polymer 1$_{\textit{cis}\text{-OMe-145}}$ (40 min sonication, Run 2)

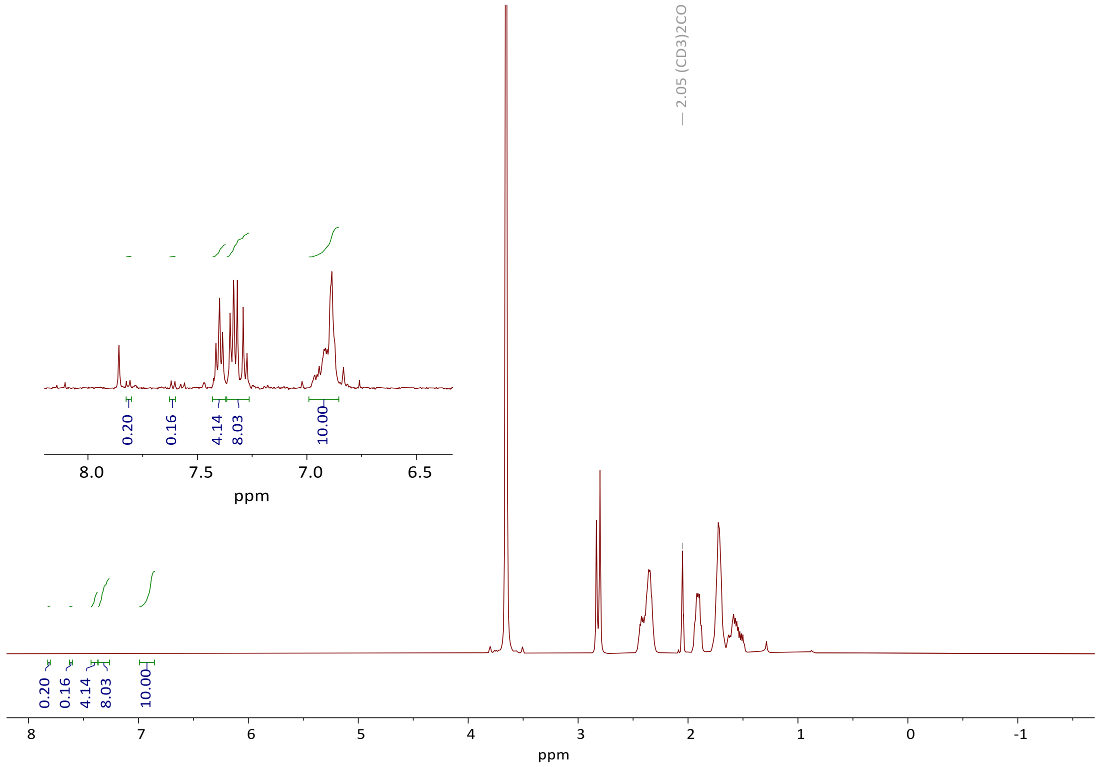

Spectrum S82. ^{1}H NMR (400 MHz, Acetone-d_6, 298 K) spectrum of post-sonication polymer **1**$_{\textbf{\textit{cis}}\textbf{-OMe-145}}$ before being washed with methanol.

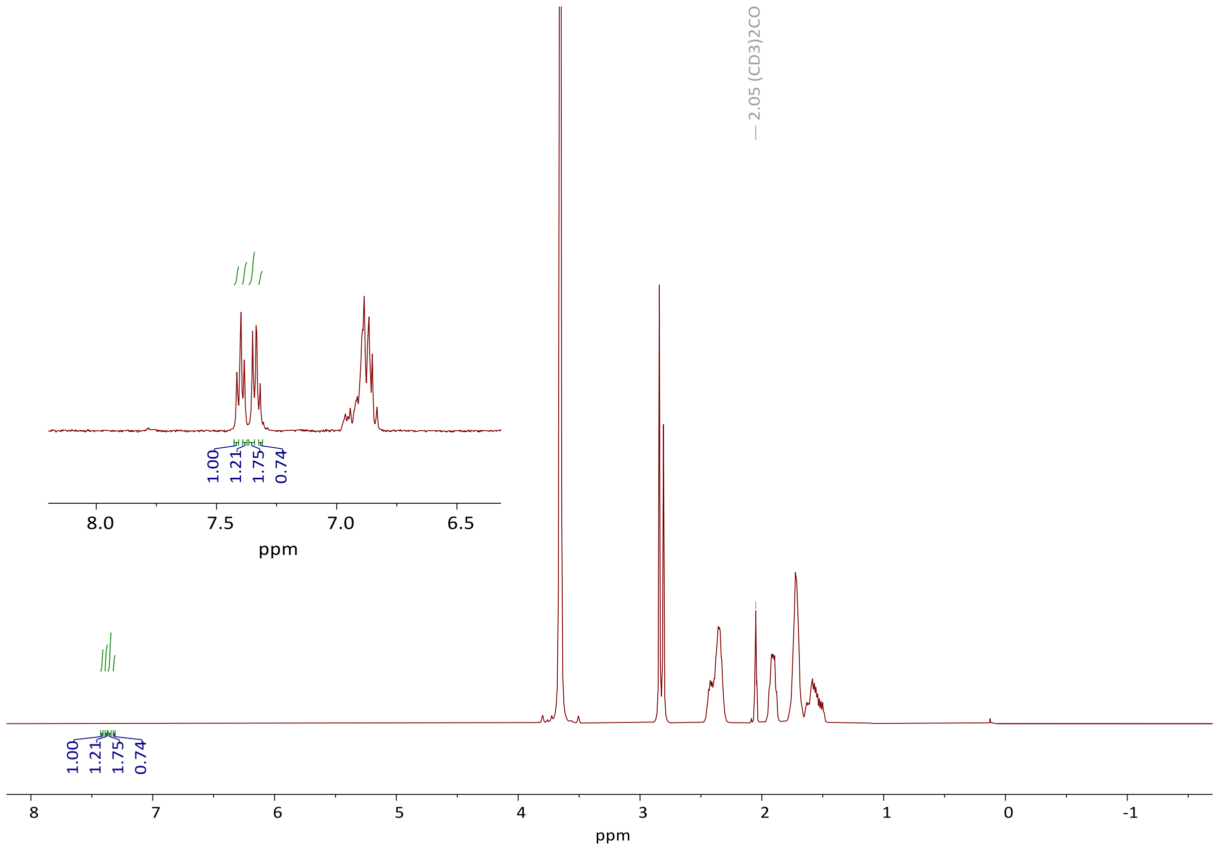

Spectrum S83. ^{1}H NMR (400 MHz, Acetone-d_6, 298 K) spectrum of post-sonication polymer $\mathbf{1}_{cis\text{-OMe-145}}$ after being washed with methanol.

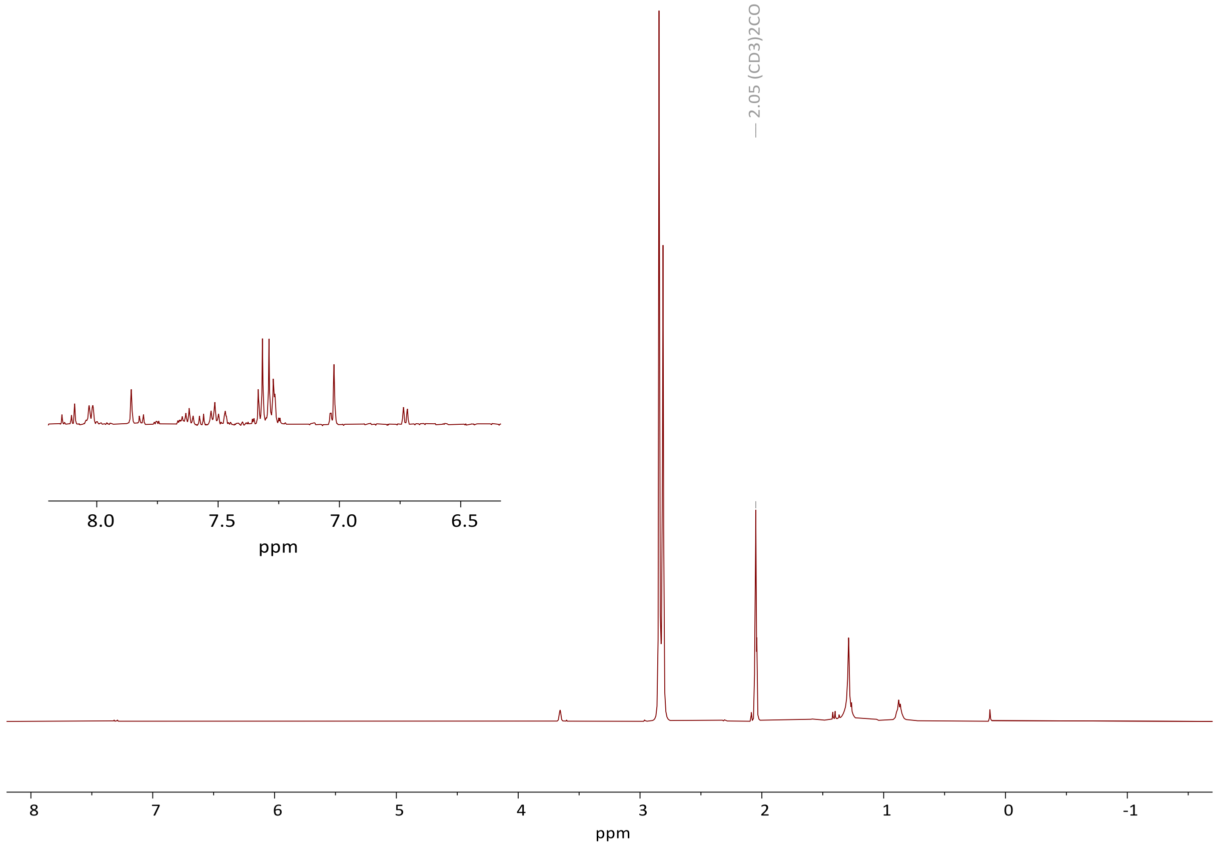

Spectrum S84. ^{1}H NMR (400 MHz, Acetone-d_6, 298 K) spectrum of the MeOH extract from post-sonication polymer $\mathbf{1}_{cis\text{-OMe-145}}$.

8.3.15 Post-Sonication ^{1}H NMR Spectra of Polymer $\mathbf{1}_{cis\text{-OEt}}$ (Run 1)

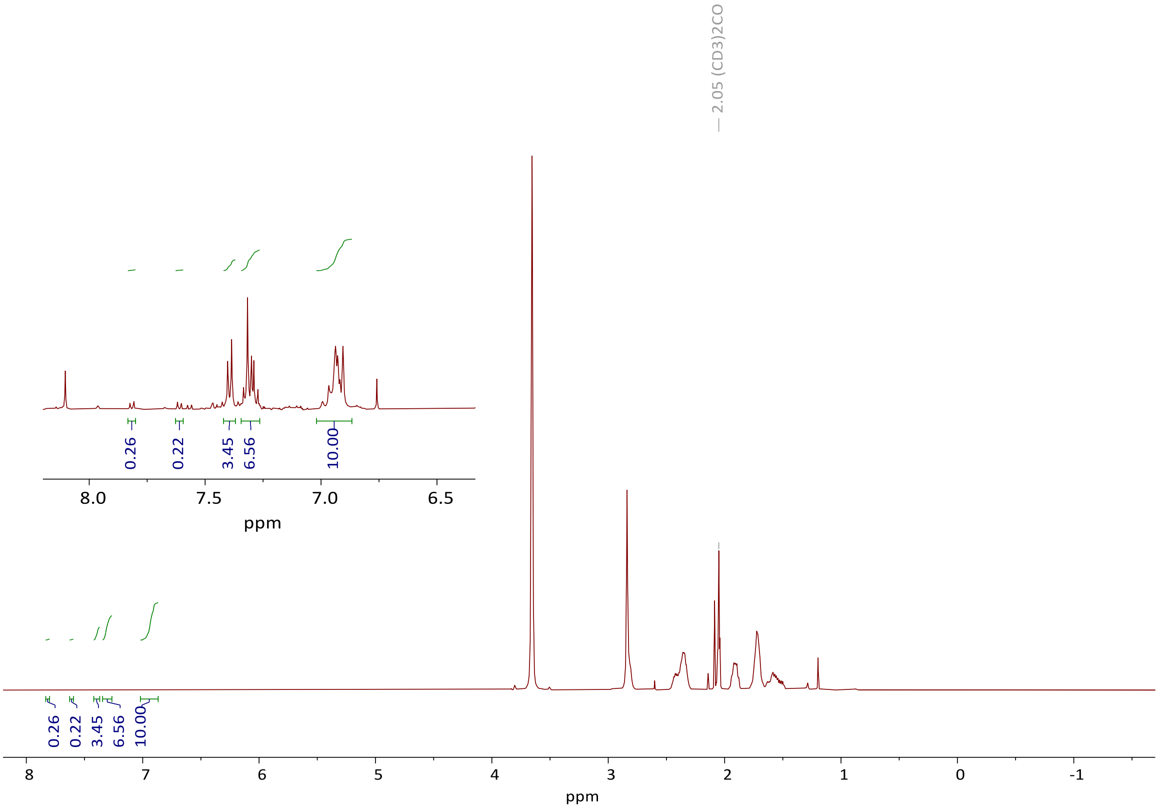

Spectrum S85. ^{1}H NMR (500 MHz, Acetone-d_6, 298 K) spectrum of post-sonication polymer $\mathbf{1}_{cis\text{-OEt}}$ before being washed with methanol.

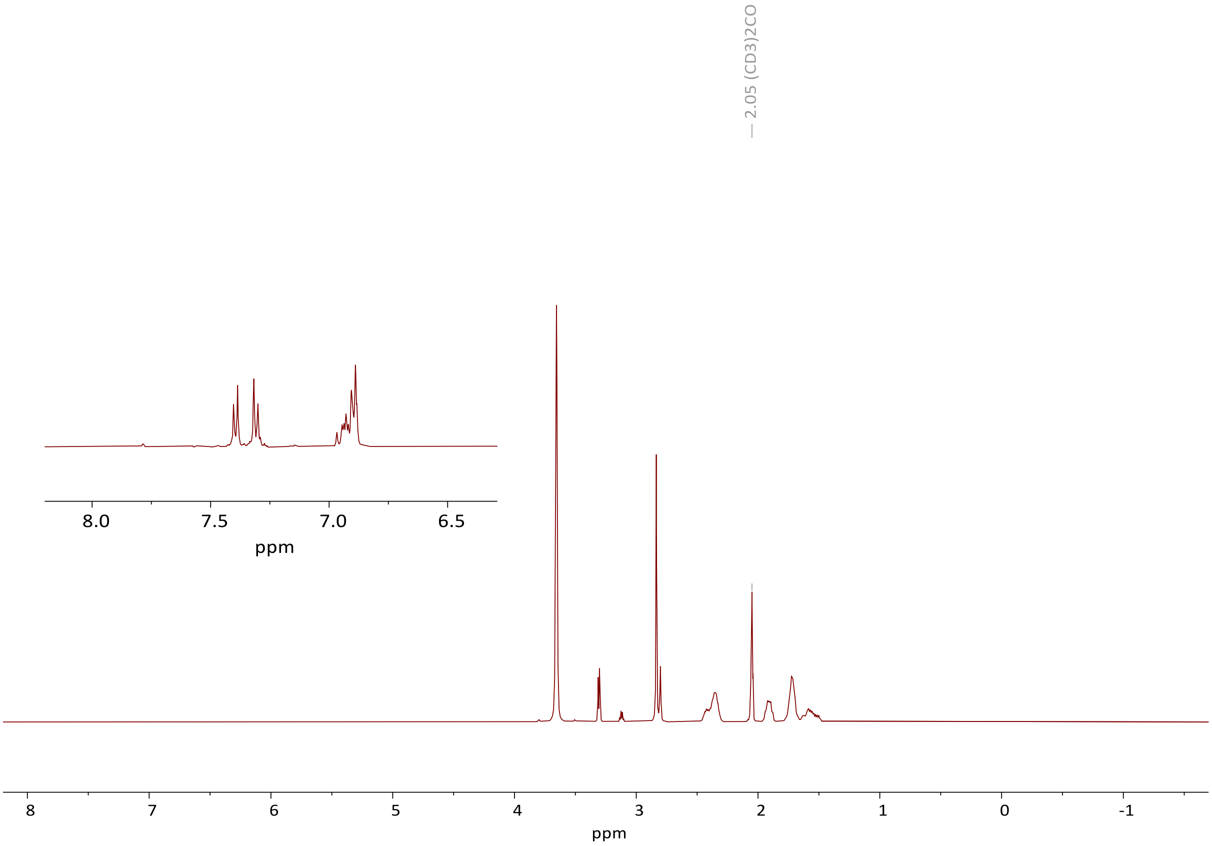

Spectrum S86. ^{1}H NMR (500 MHz, Acetone-d_6, 298 K) spectrum of post-sonication polymer $\mathbf{1}_{cis\text{-OEt}}$ after being washed with methanol.

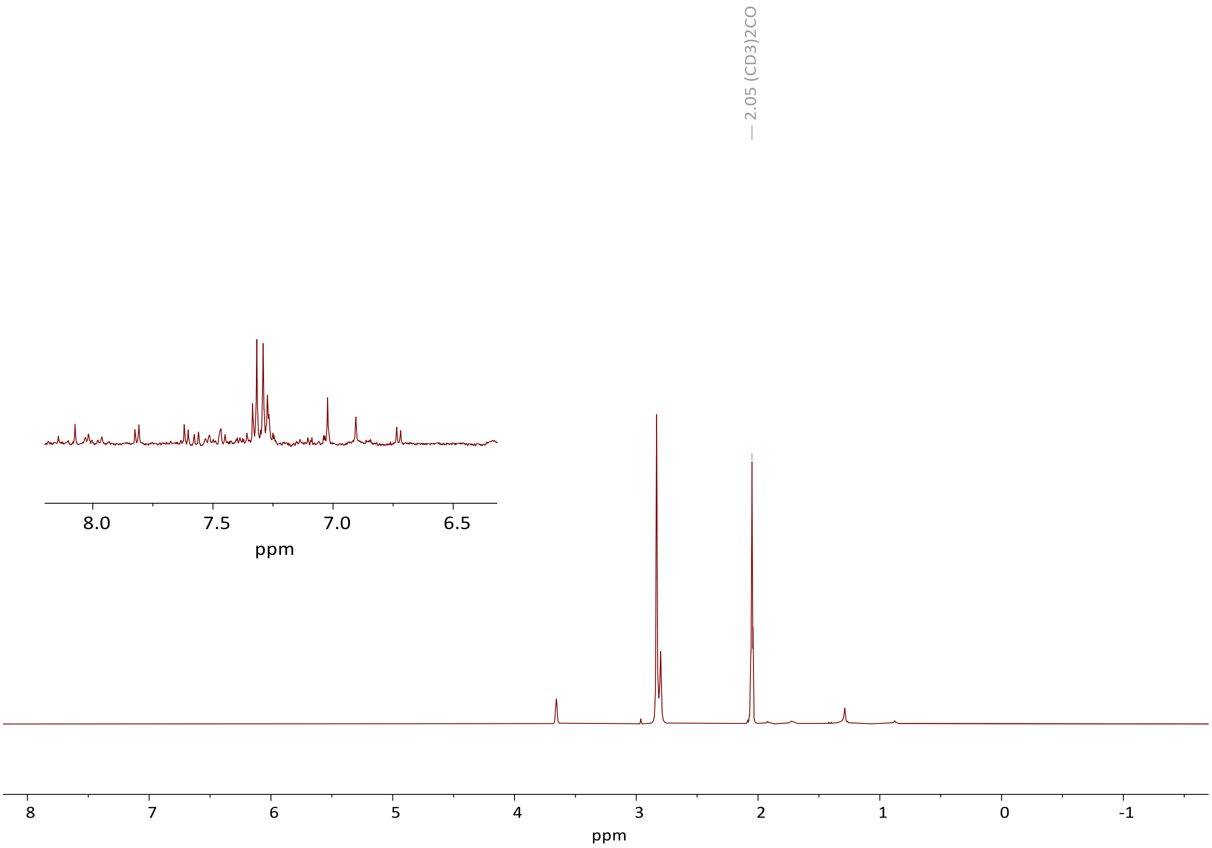

Spectrum S87. ^{1}H NMR (500 MHz, Acetone-d_6, 298 K) spectrum of the MeOH extract from post-sonication polymer **1***cis*-OEt.

8.3.16 Post-Sonication ^{1}H NMR Spectra of Polymer 1*cis*-OEt (Run 2)

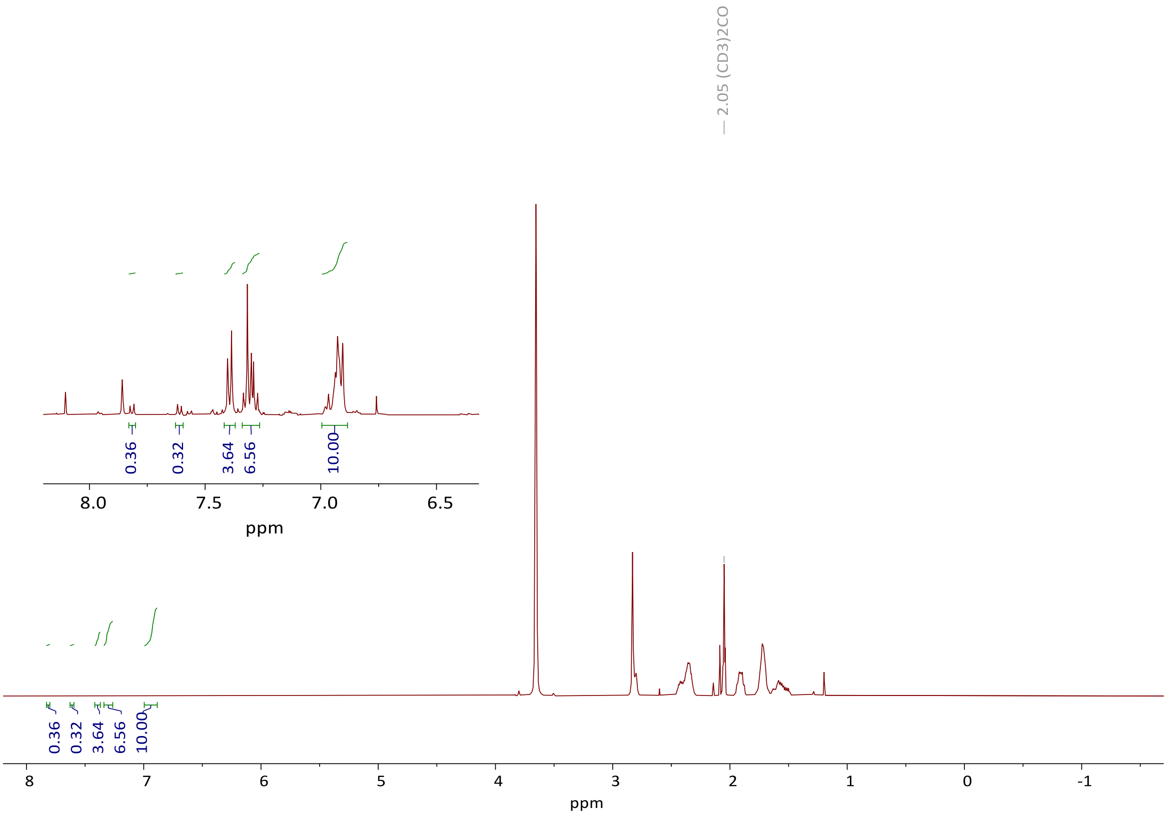

Spectrum S88. ^{1}H NMR (500 MHz, Acetone-d_6, 298 K) spectrum of post-sonication polymer **1***cis*-OEt before being washed with methanol.

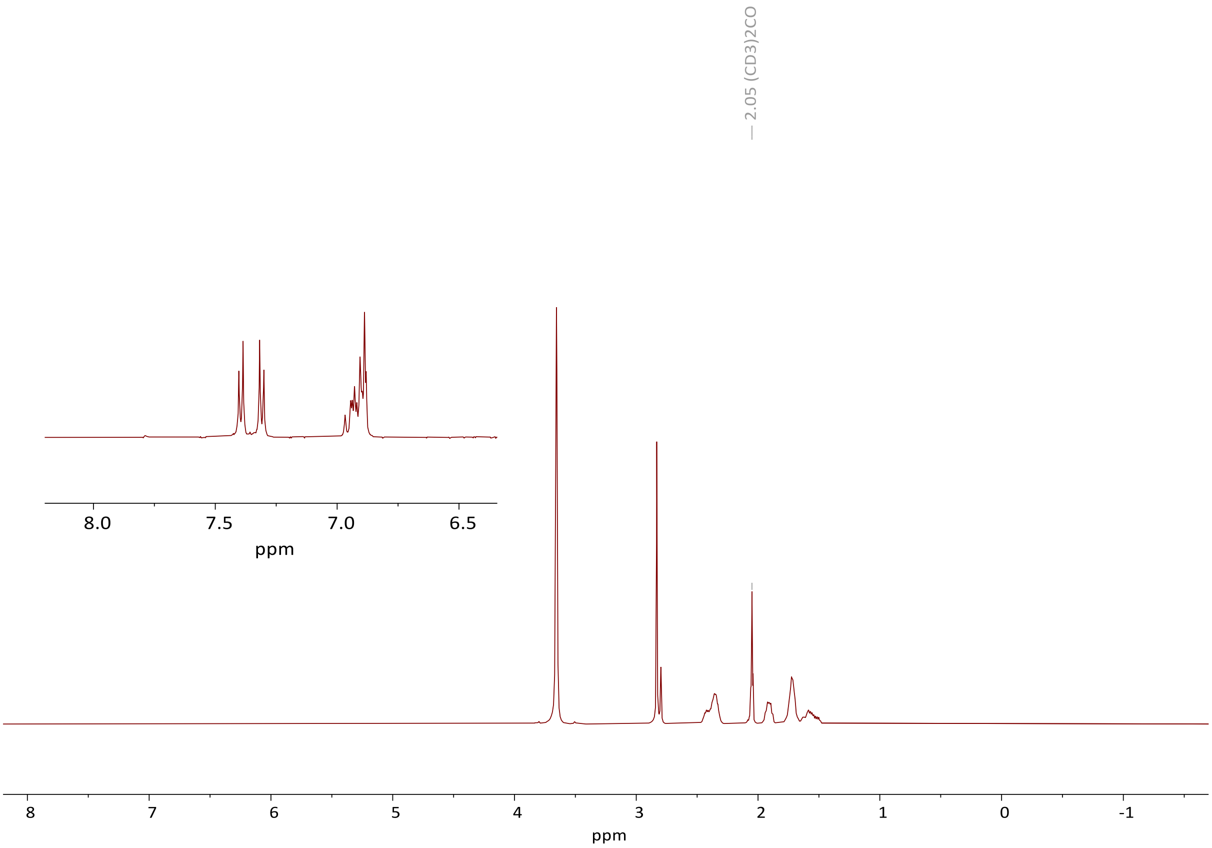

Spectrum S89. ^{1}H NMR (500 MHz, Acetone-d_6, 298 K) spectrum of post-sonication polymer $\mathbf{1}_{\textit{cis}\text{-}\mathbf{OEt}}$ after being washed with methanol.

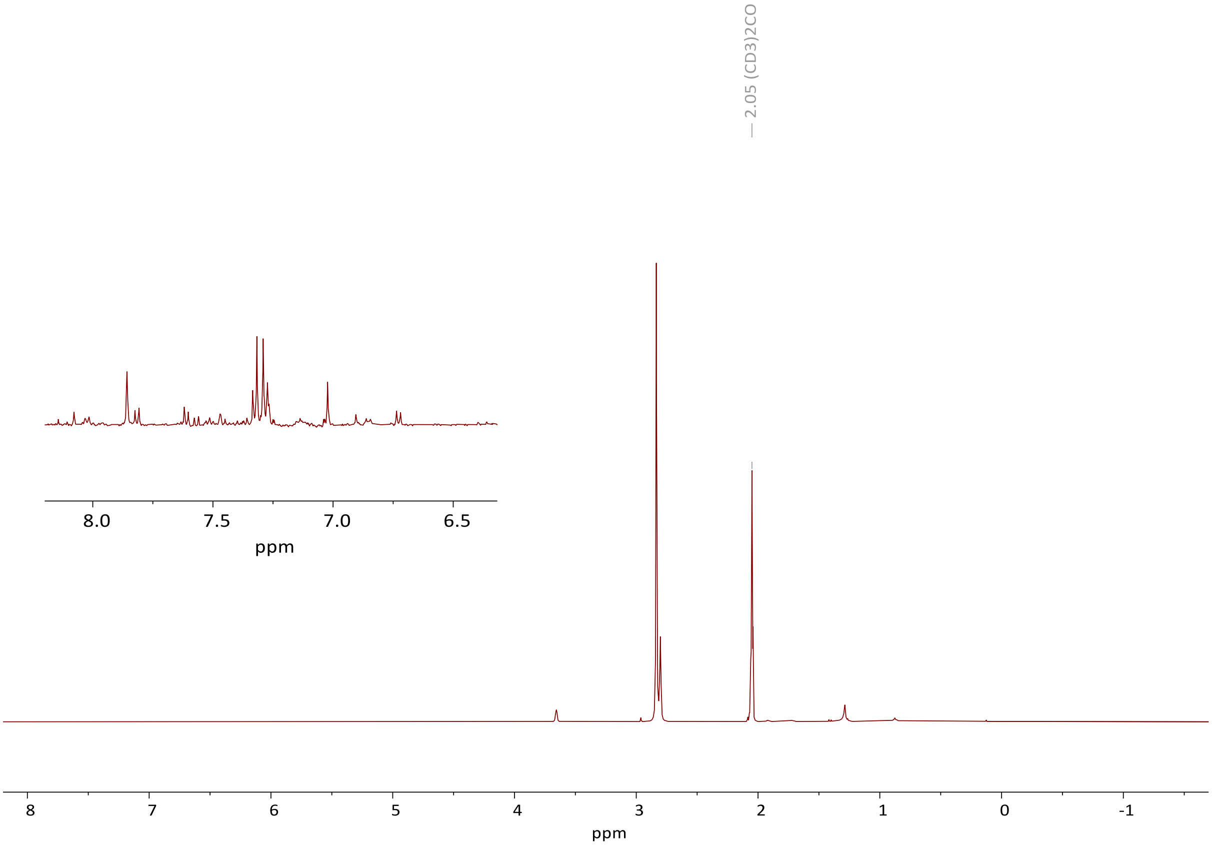

Spectrum S90. ^{1}H NMR (500 MHz, Acetone-d_6, 298 K) spectrum of the MeOH extract from post-sonication polymer $\mathbf{1}_{\textit{cis}\text{-}\mathbf{OEt}}$.

8.3.17 Post-Sonication ^{1}H NMR Spectra of Polymer $\mathbf{3}_{cis}$ (Run 1)

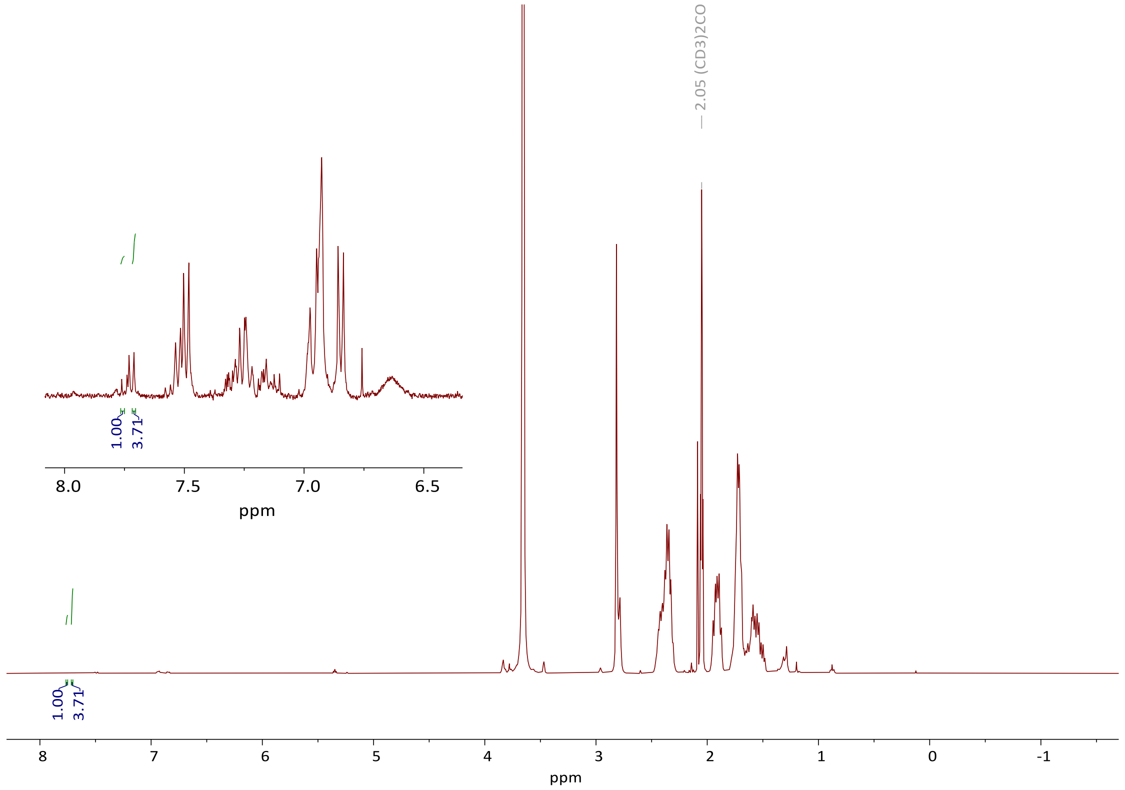

Spectrum S91. ^{1}H NMR (400 MHz, Acetone-d_6, 298 K) spectrum of post-sonication polymer $\mathbf{3}_{cis}$ before being washed with methanol.

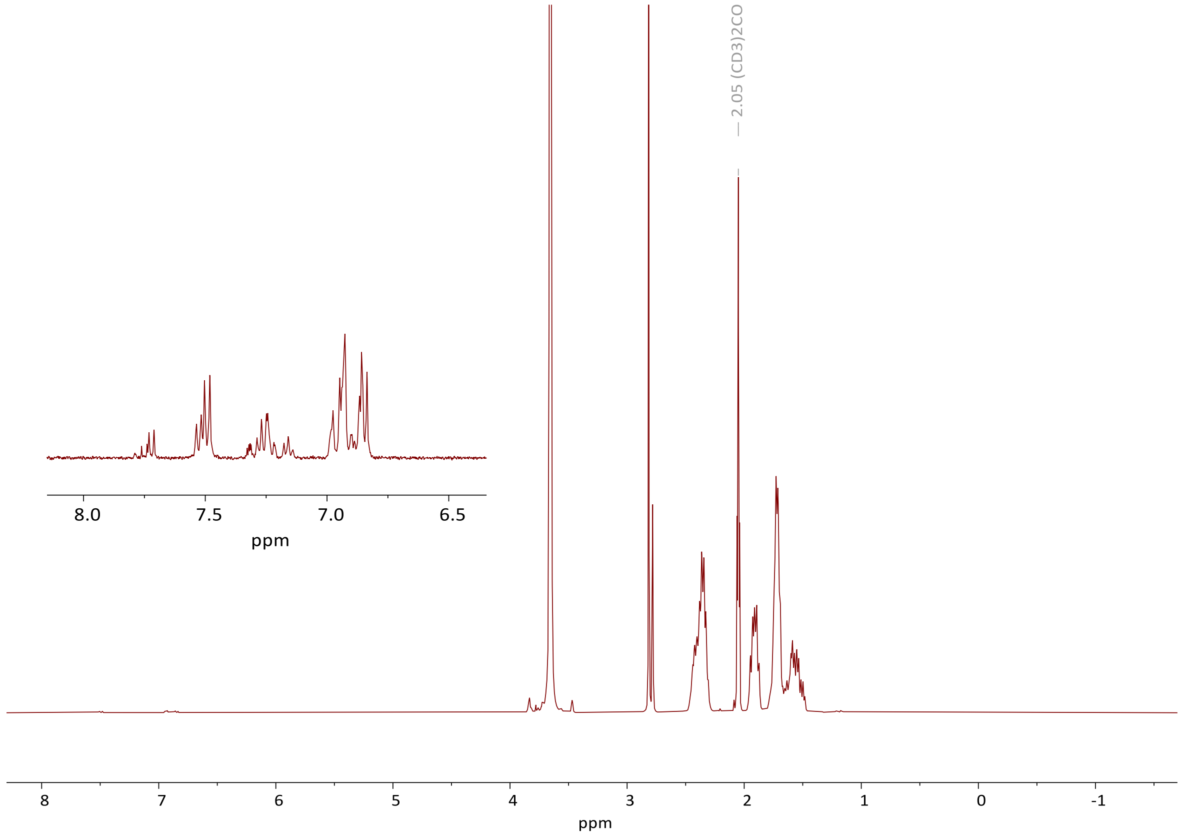

Spectrum S92. ^{1}H NMR (400 MHz, Acetone-d_6, 298 K) spectrum of post-sonication polymer $\mathbf{3}_{cis}$ after being washed with methanol.

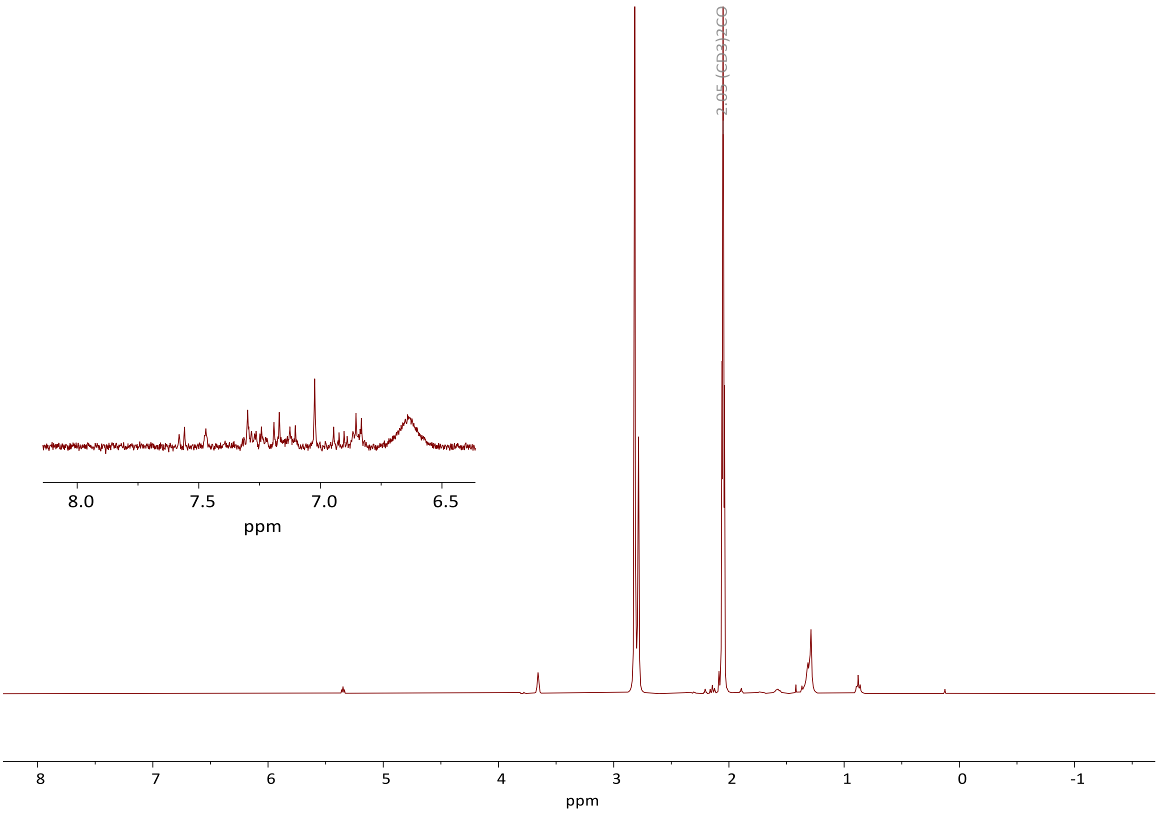

Spectrum S93. ^{1}H NMR (400 MHz, Acetone-d_6, 298 K) spectrum of the MeOH extract from post-sonication polymer **3*cis***.

8.3.18 Post-Sonication ^{1}H NMR Spectra of Polymer 3*cis* (Run 2)

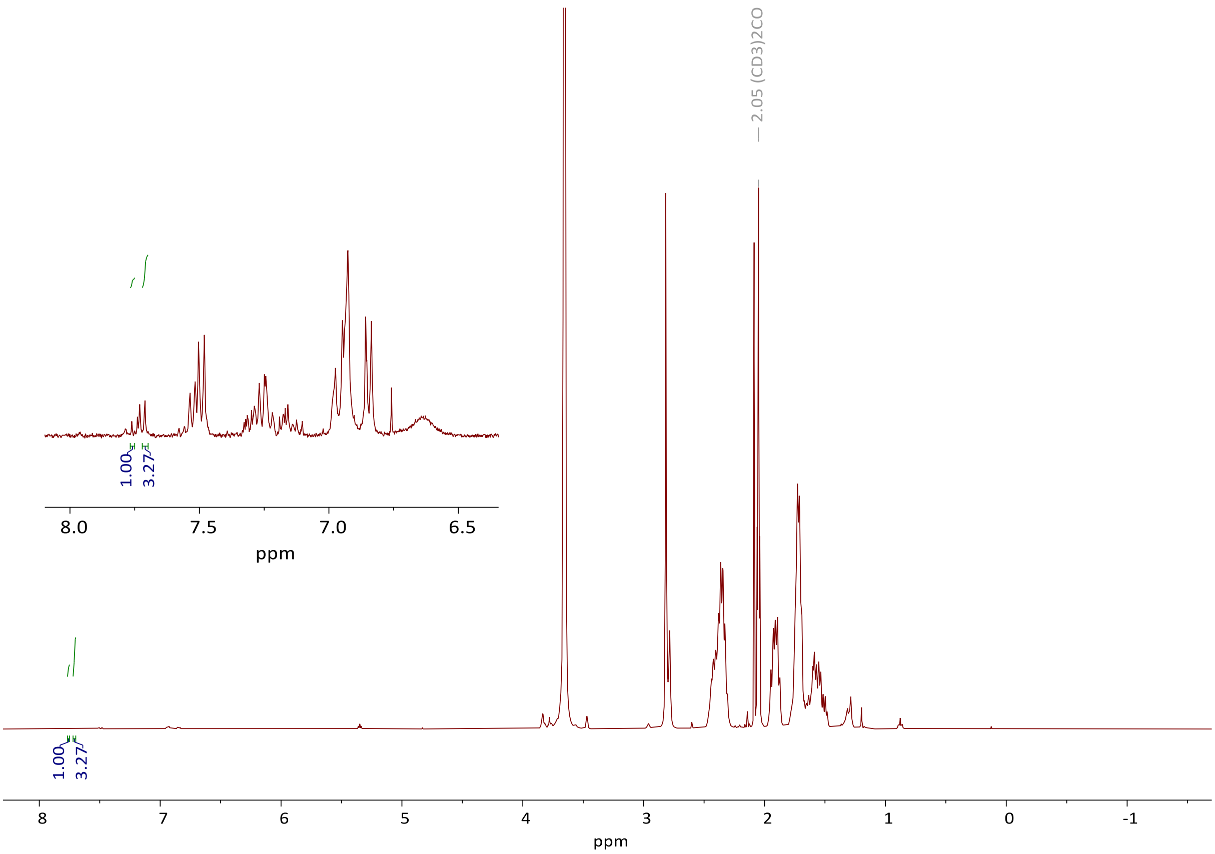

Spectrum S94. ^{1}H NMR (400 MHz, Acetone-d_6, 298 K) spectrum of post-sonication polymer **3*cis*** before being washed with methanol.

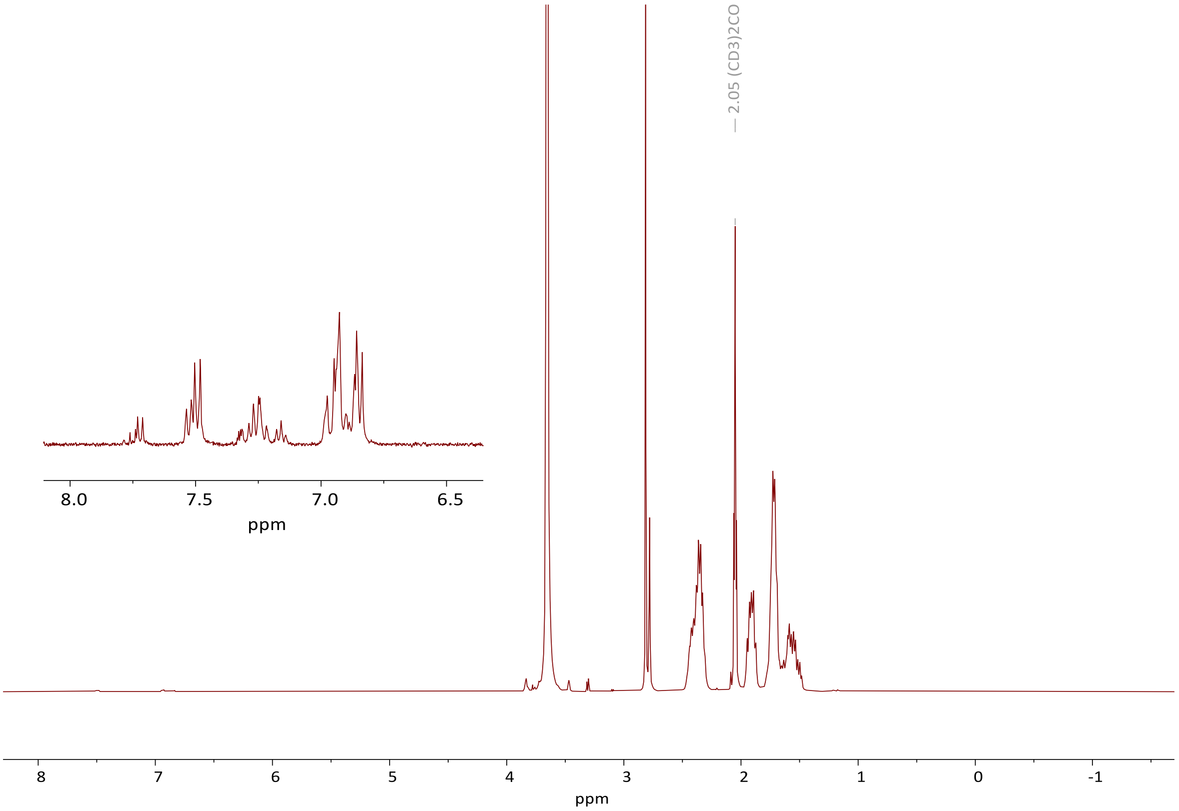

Spectrum S95. ^{1}H NMR (400 MHz, Acetone-d_6, 298 K) spectrum of post-sonication polymer $\mathbf{3}_{\boldsymbol{cis}}$ after being washed with methanol.

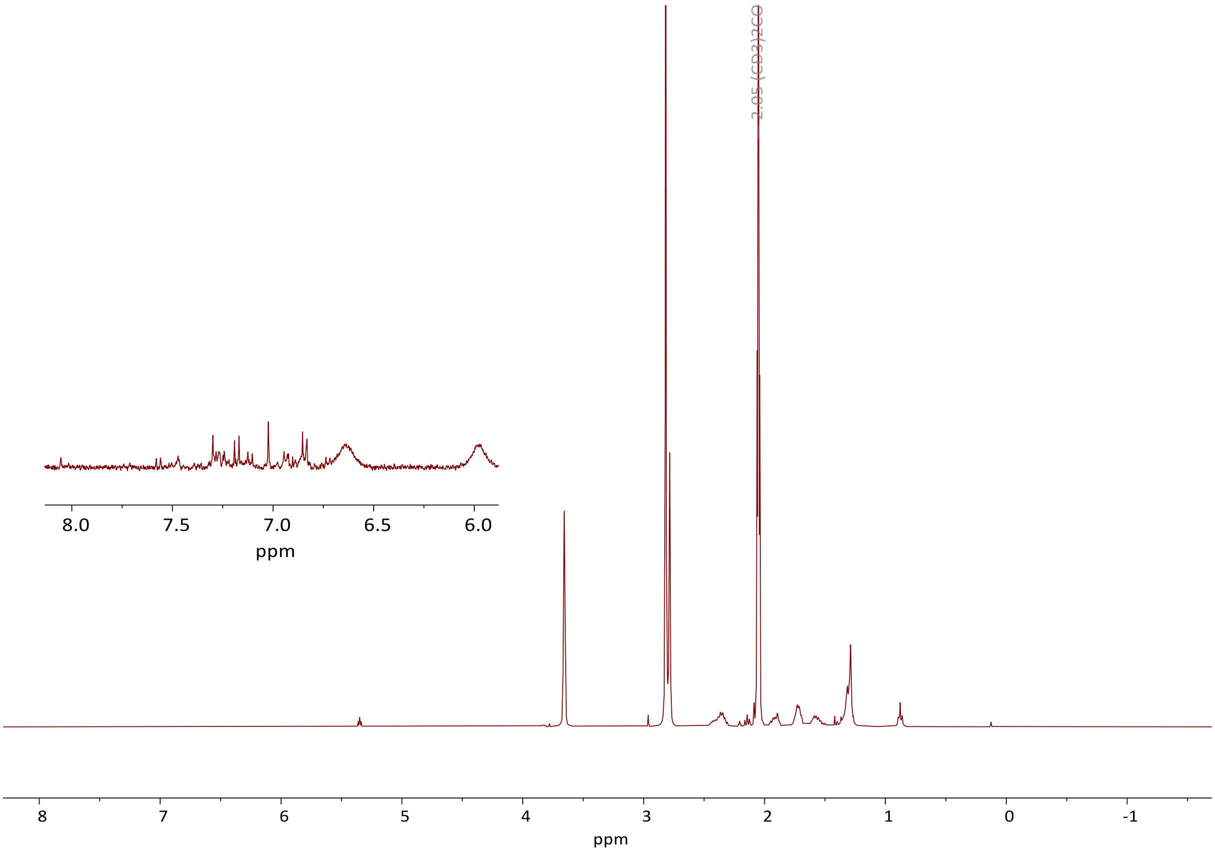

Spectrum S96. ^{1}H NMR (400 MHz, Acetone-d_6, 298 K) spectrum of the MeOH extract from post-sonication polymer $\mathbf{3}_{\boldsymbol{cis}}$.

8.3.19 Post-Sonication ^{1}H NMR Spectra of Polymer $\mathbf{3}_{cis}$ (Run 1 and run 2)

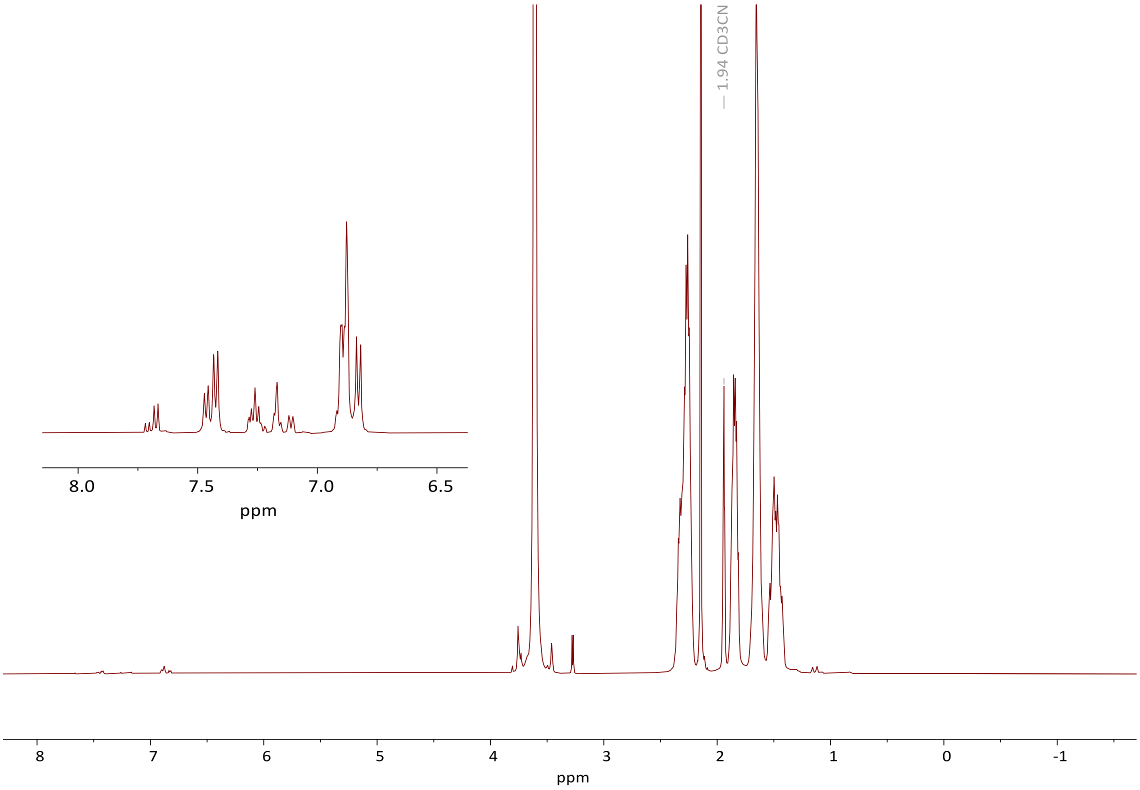

Spectrum S97. ^{1}H NMR (500 MHz, Acetonitrile-d_6, 298 K) spectrum of post-sonication polymer $\mathbf{3}_{cis}$ after being washed with methanol.

8.3.20 Post-Sonication ^{1}H NMR Spectra of Polymer $\mathbf{1}_{trans\text{-OMe}}$ (Run 1)

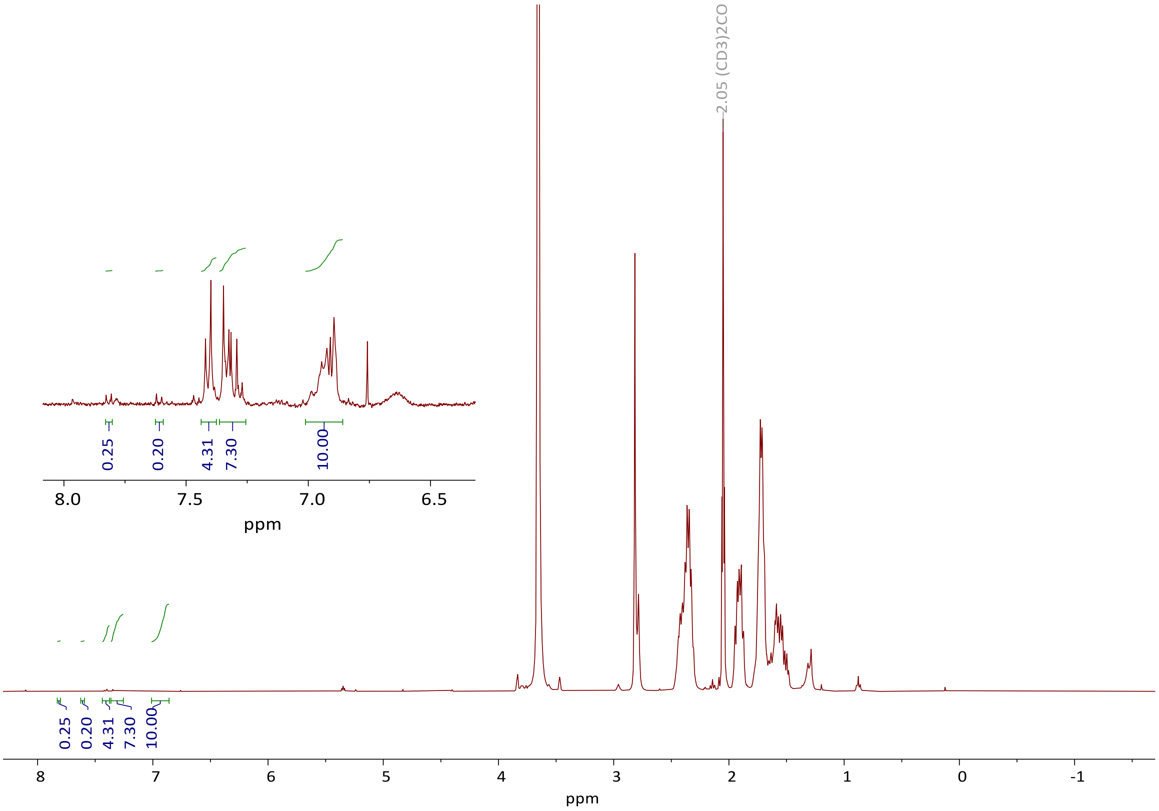

Spectrum S98. ^{1}H NMR (400 MHz, Acetone-d_6, 298 K) spectrum of post-sonication polymer $\mathbf{1}_{trans\text{-OMe}}$ before being washed with methanol.

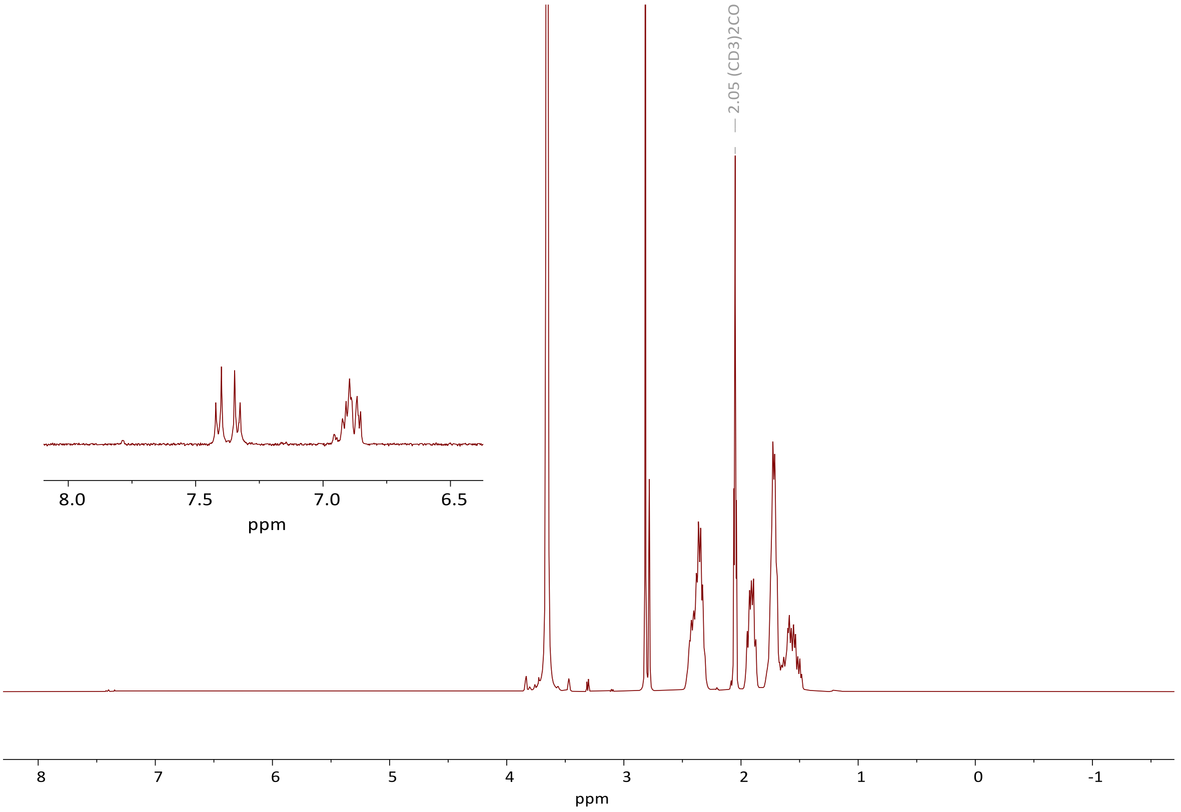

Spectrum S99. ^{1}H NMR (400 MHz, Acetone-d_6, 298 K) spectrum of post-sonication polymer **1***trans*-**OMe** after being washed with methanol.

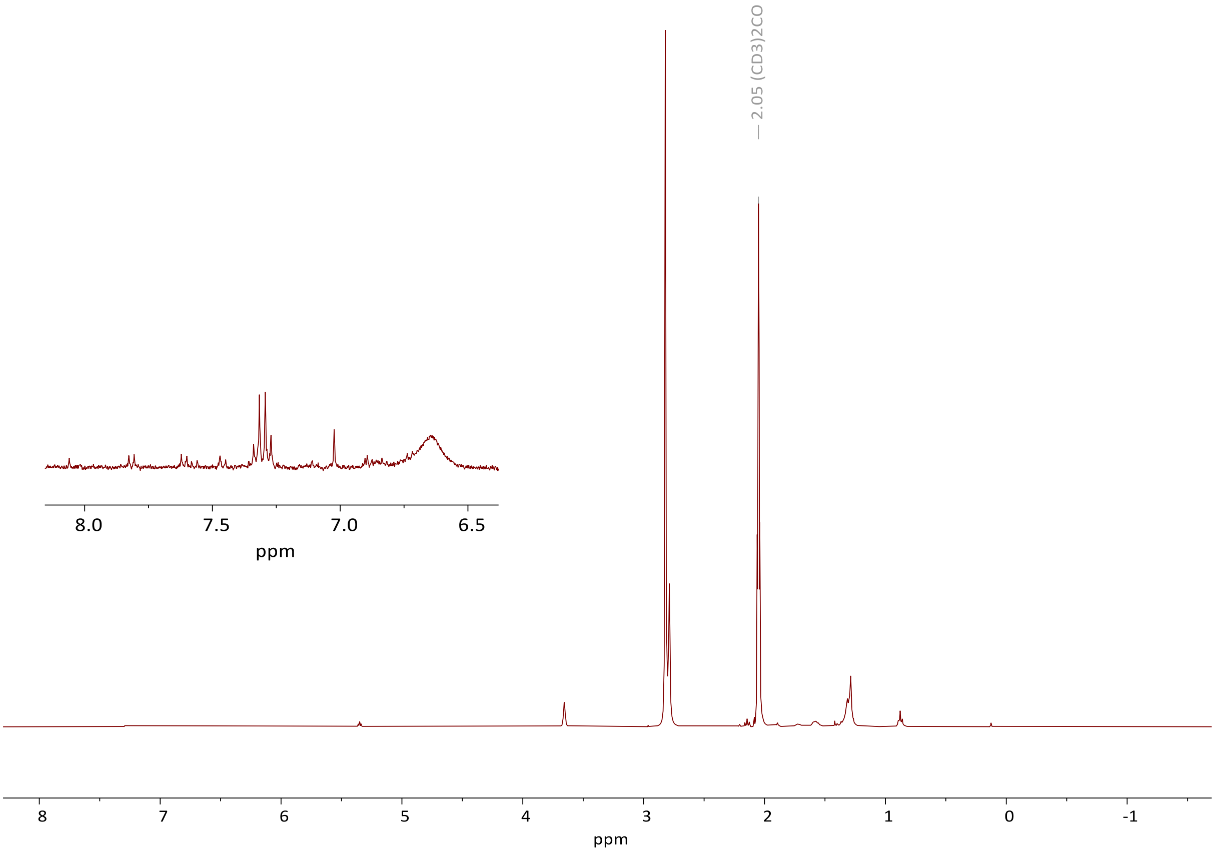

Spectrum S100. ^{1}H NMR (400 MHz, Acetone-d_6, 298 K) spectrum of the MeOH extract from post-sonication polymer **1***trans*-**OMe**.

8.3.21 Post-Sonication ^{1}H NMR Spectra of Polymer $\mathbf{1}_{\textit{trans}\text{-OMe}}$ (Run 2)

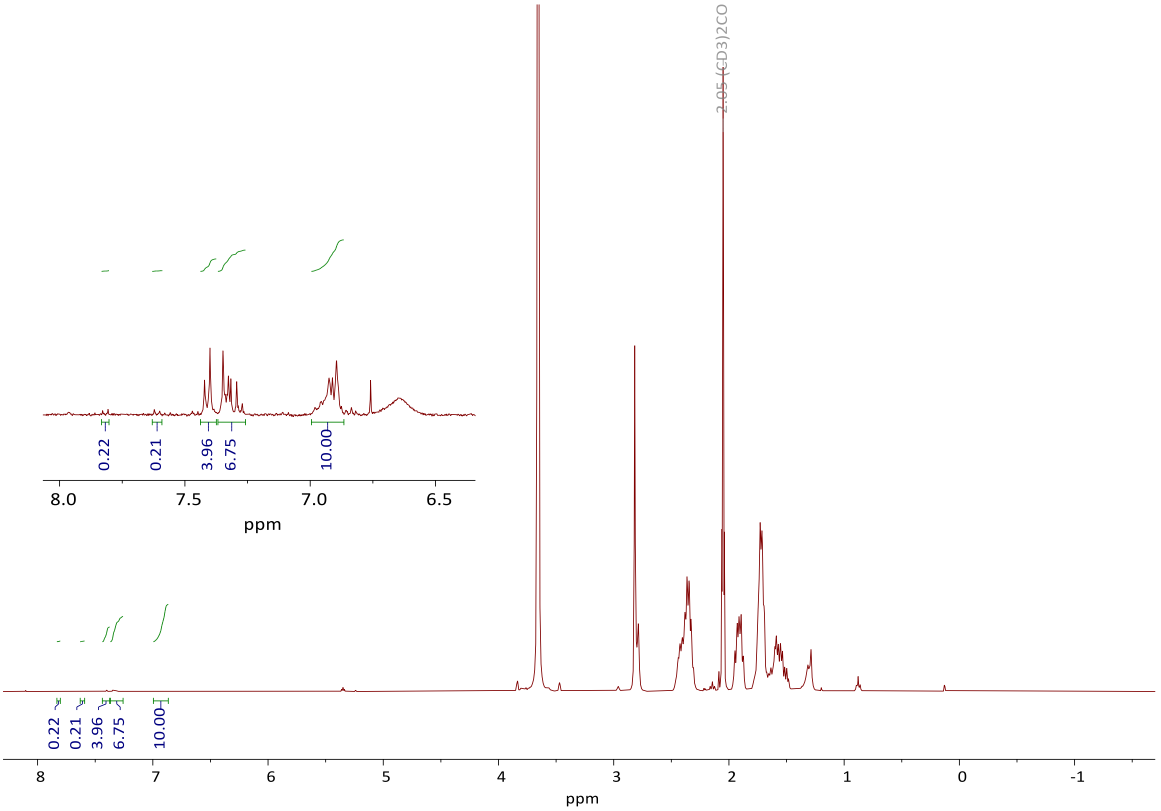

Spectrum S101. ^{1}H NMR (400 MHz, Acetone-d_6, 298 K) spectrum of post-sonication polymer $\mathbf{1}_{\textit{trans}\text{-OMe}}$ before being washed with methanol.

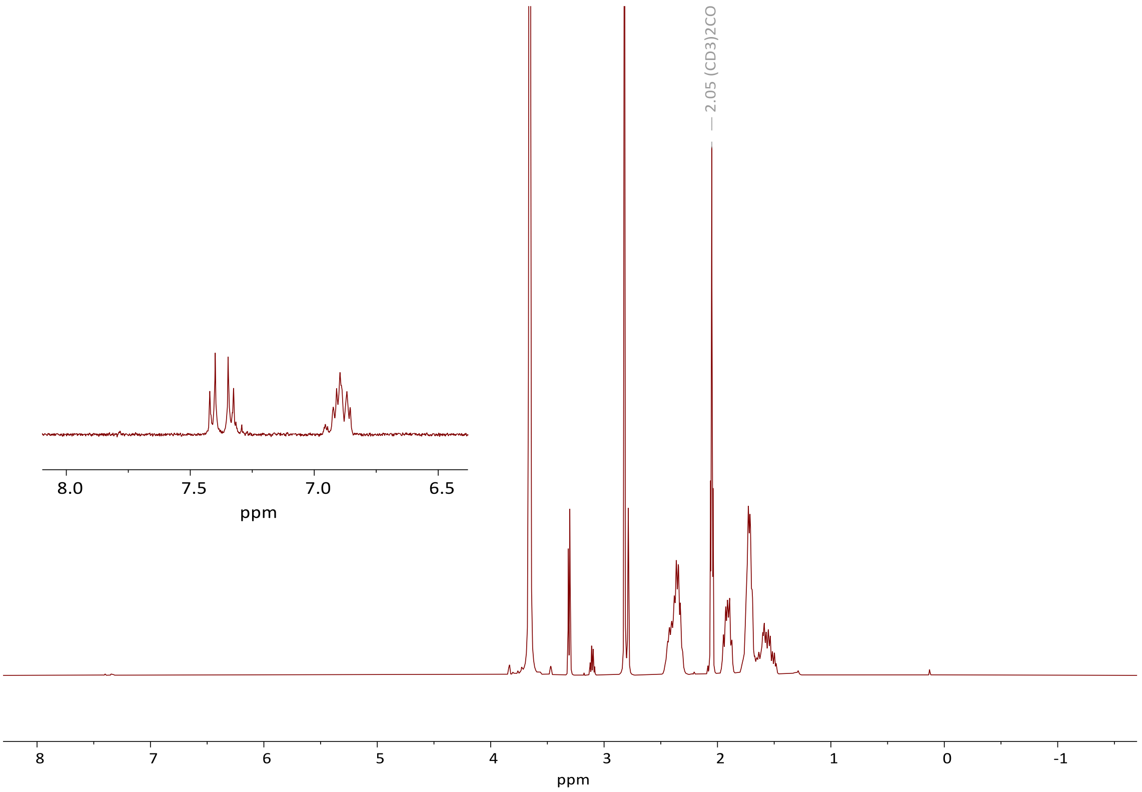

Spectrum S102. ^{1}H NMR (400 MHz, Acetone-d_6, 298 K) spectrum of post-sonication polymer $\mathbf{1}_{\textit{trans}\text{-OMe}}$ after being washed with methanol.

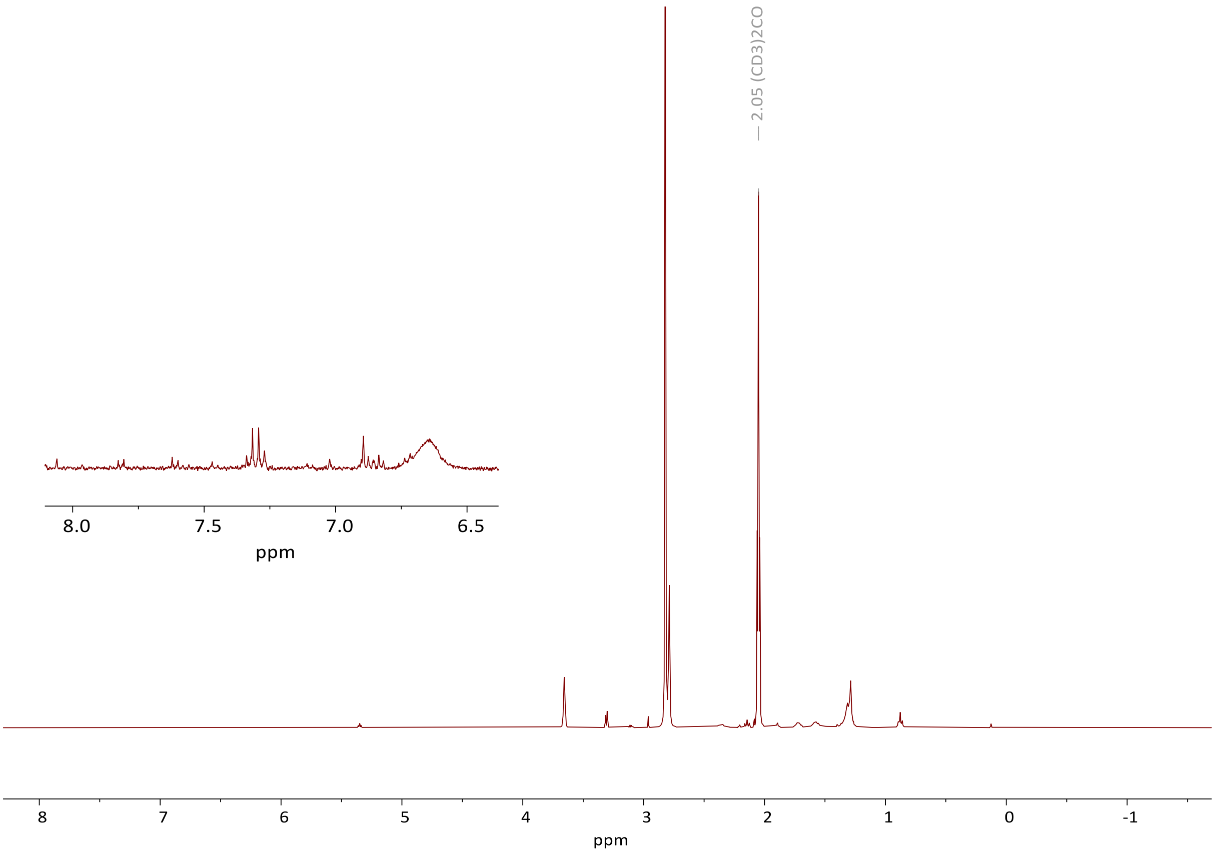

Spectrum S103. ^{1}H NMR (400 MHz, Acetone-d_6, 298 K) spectrum of the MeOH extract from post-sonication polymer $\mathbf{1}_{\textit{trans}\text{-OMe}}$.

8.3.22 Post-Sonication ^{1}H NMR Spectra of Polymer $\mathbf{1}_{\textit{trans}\text{-OMe}}$ (Run 3)

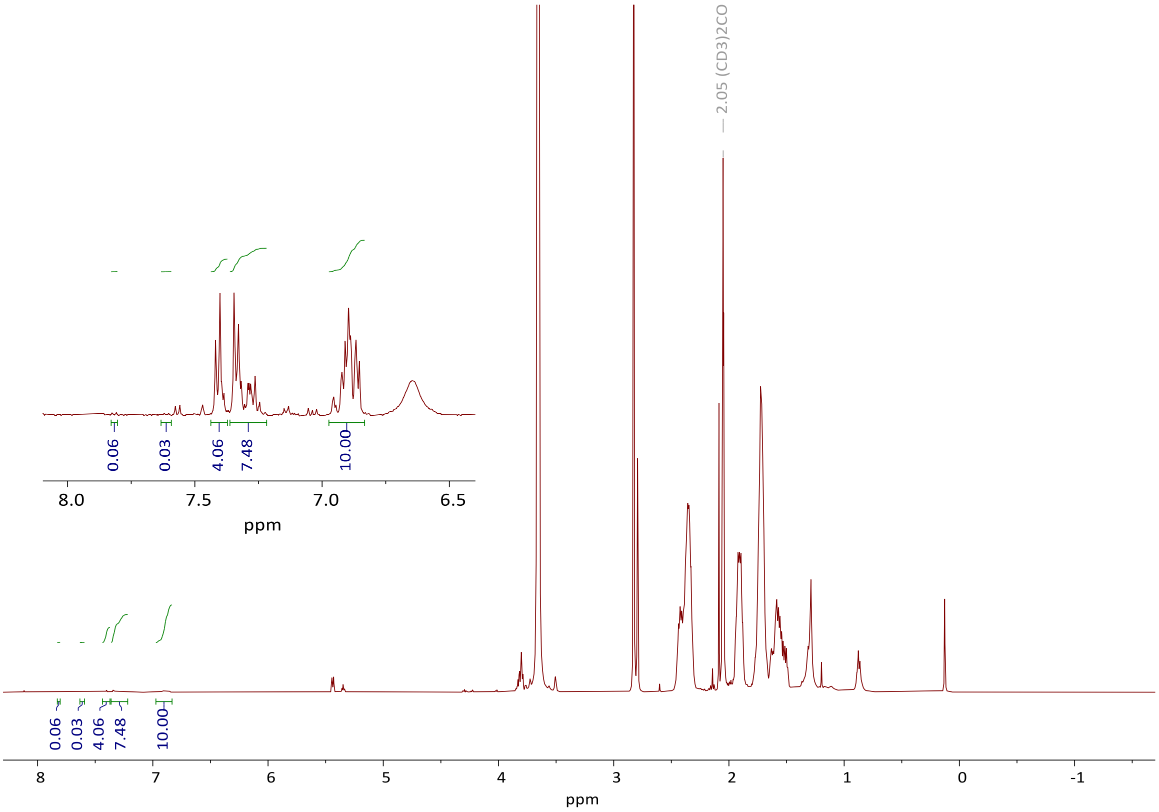

Spectrum S104. ^{1}H NMR (400 MHz, Acetone-d_6, 298 K) spectrum of post-sonication polymer $\mathbf{1}_{\textit{trans}\text{-OMe}}$ before being washed with methanol.

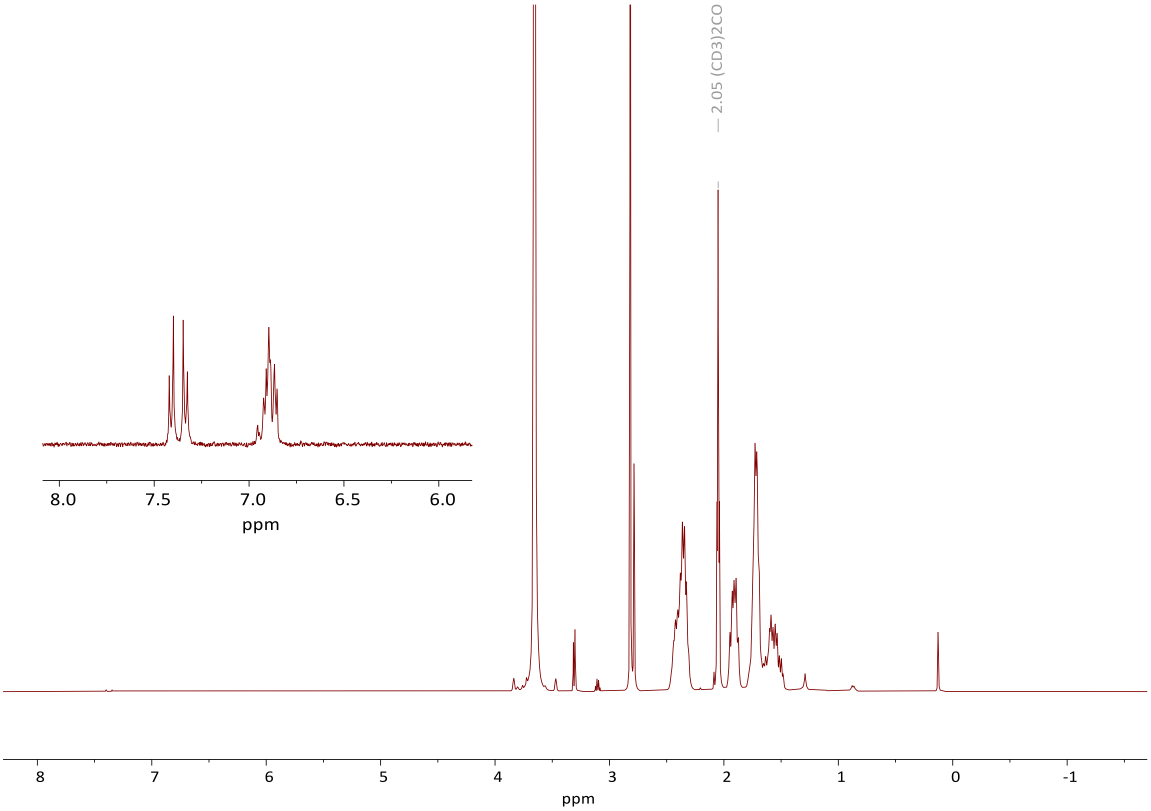

Spectrum S105. ^{1}H NMR (400 MHz, Acetone-d_6, 298 K) spectrum of post-sonication polymer **1***trans***-OMe** after being washed with methanol.

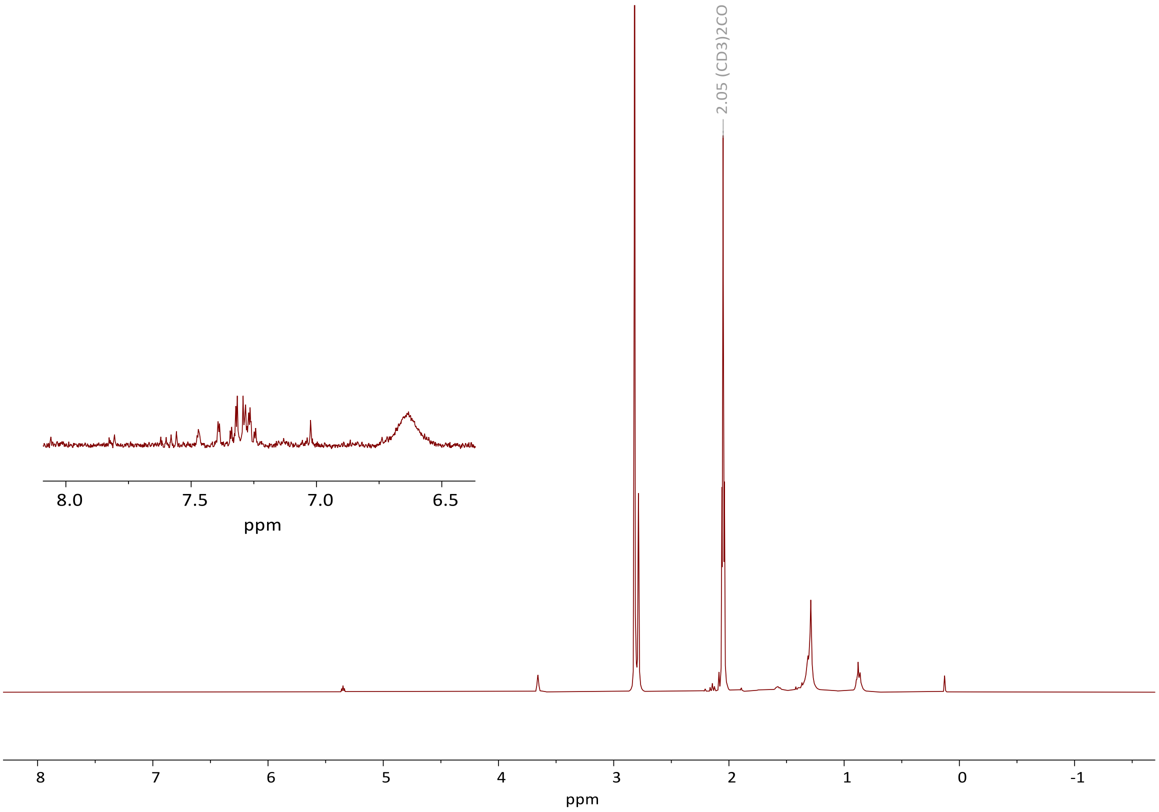

Spectrum S106. ^{1}H NMR (400 MHz, Acetone-d_6, 298 K) spectrum of the MeOH extract from post-sonication polymer **1***trans***-OMe**.

8.3.23 Post-Sonication ^{1}H NMR Spectra of Polymer $\mathbf{2}_{cis}$ (Run 1)

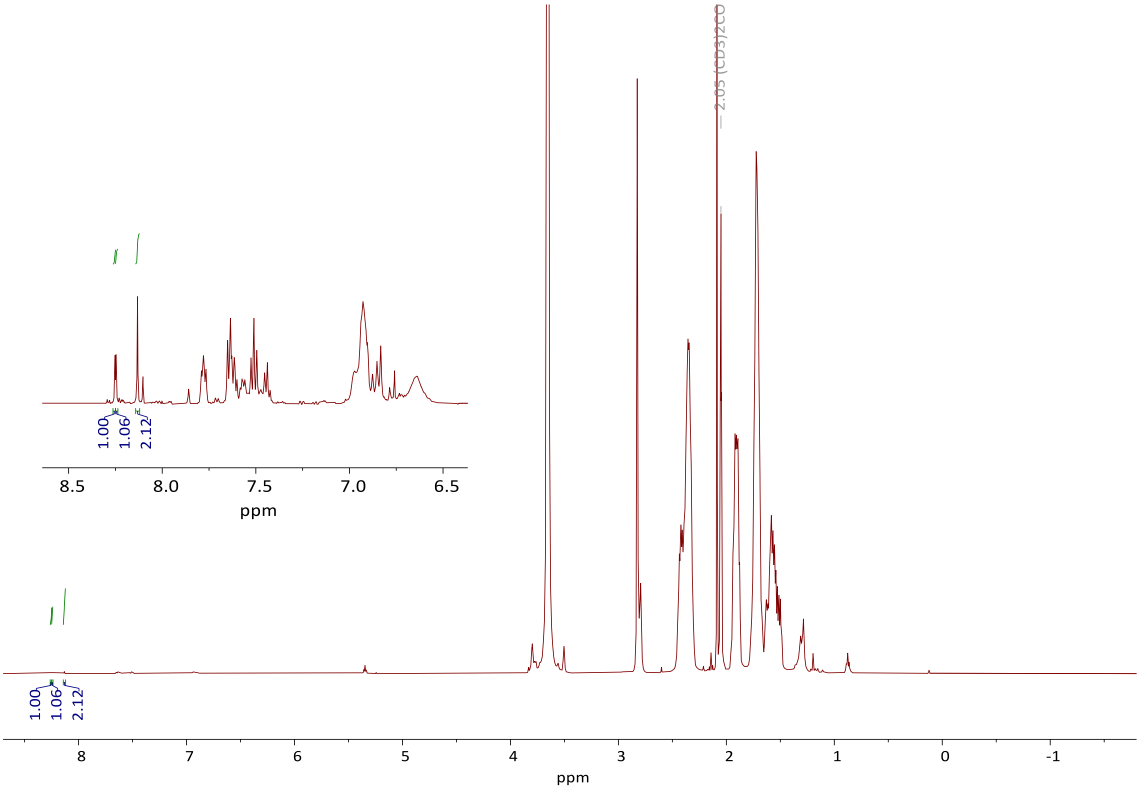

Spectrum S107. ^{1}H NMR (500 MHz, Acetone-d_6, 298 K) spectrum of post-sonication polymer $\mathbf{2}_{cis}$ before being washed with methanol.

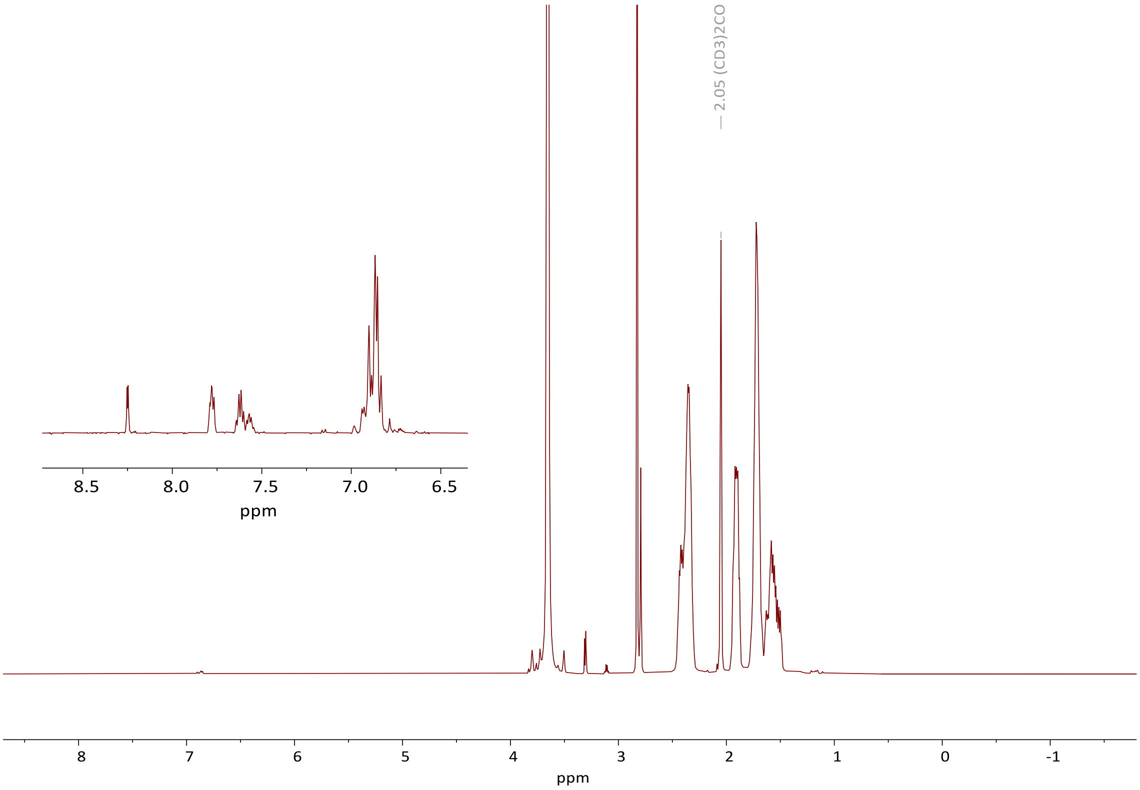

Spectrum S108. ^{1}H NMR (500 MHz, Acetone-d_6, 298 K) spectrum of post-sonication polymer $\mathbf{2}_{cis}$ after being washed with methanol.

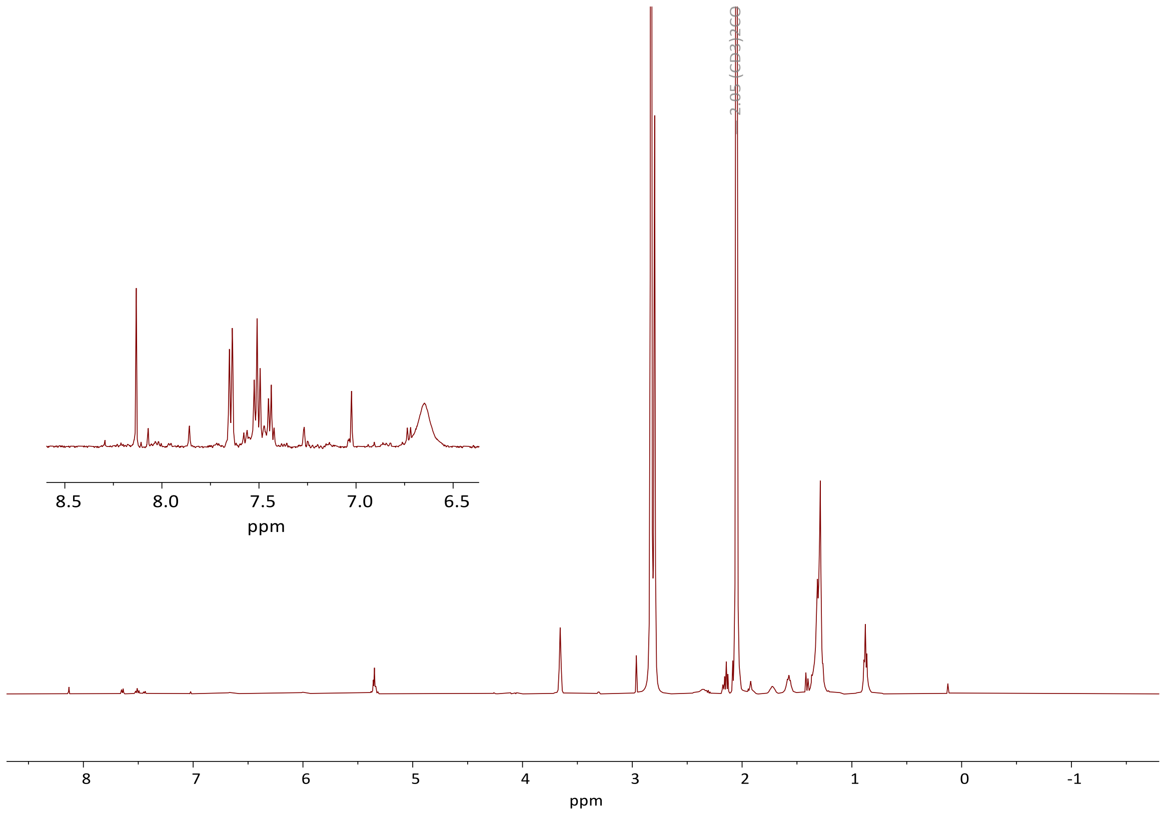

Spectrum S109. ^{1}H NMR (500 MHz, Acetone-d_6, 298 K) spectrum of the MeOH extract from post-sonication polymer $\mathbf{2}_{cis}$.

8.3.24 Post-Sonication ^{1}H NMR Spectra of Polymer $\mathbf{2}_{cis}$ (Run 2)

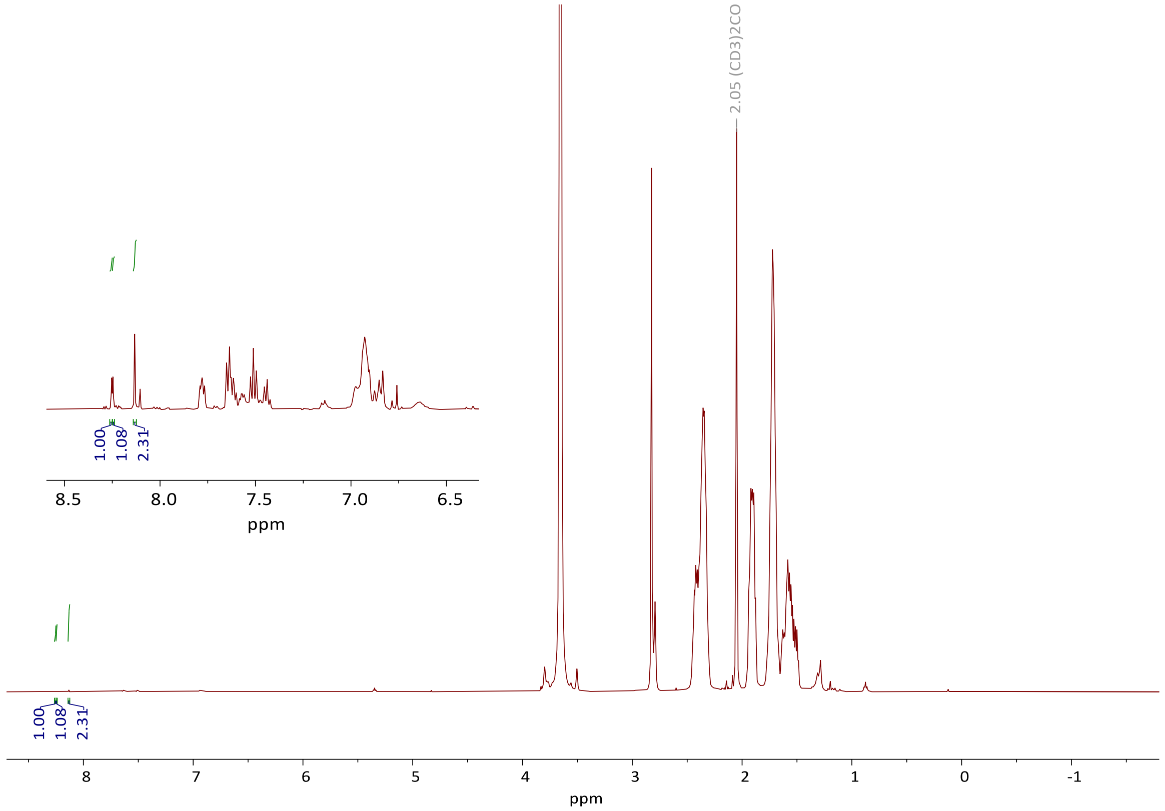

Spectrum S110. ^{1}H NMR (500 MHz, Acetone-d_6, 298 K) spectrum of post-sonication polymer $\mathbf{2}_{cis}$ before being washed with methanol.

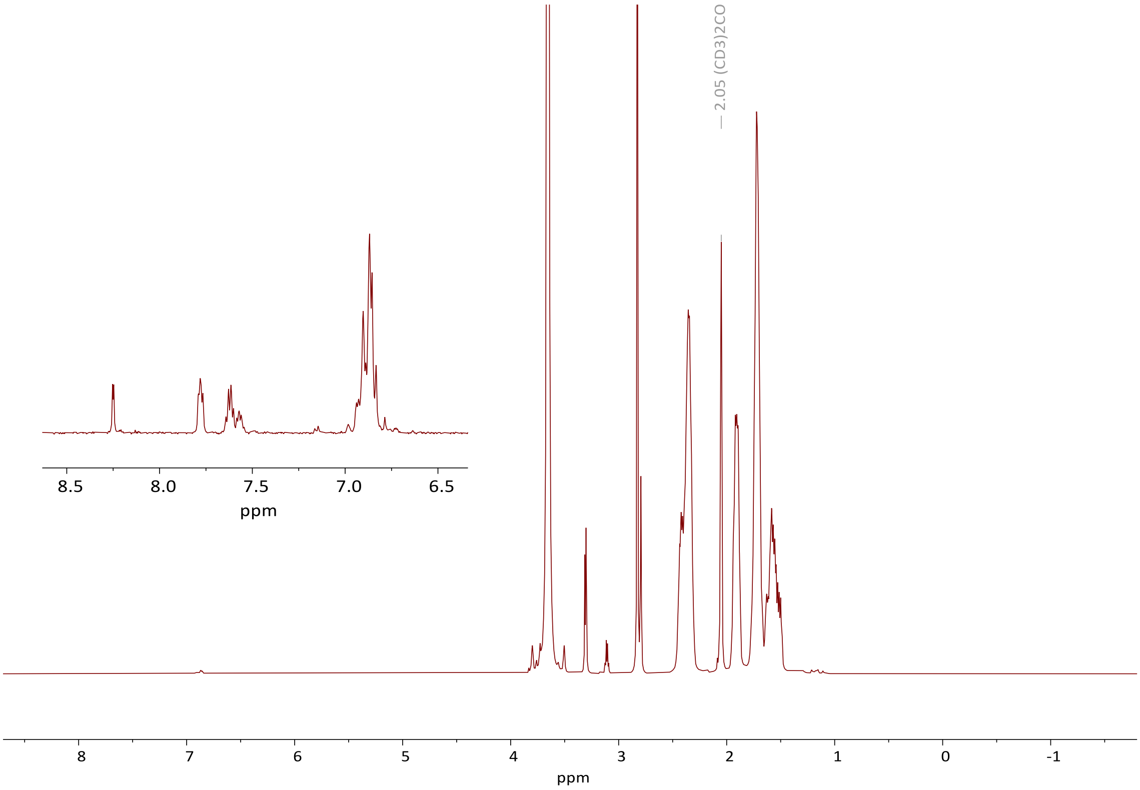

Spectrum S111. ^{1}H NMR (500 MHz, Acetone-d_6, 298 K) spectrum of post-sonication polymer **2**$_{cis}$ after being washed with methanol.

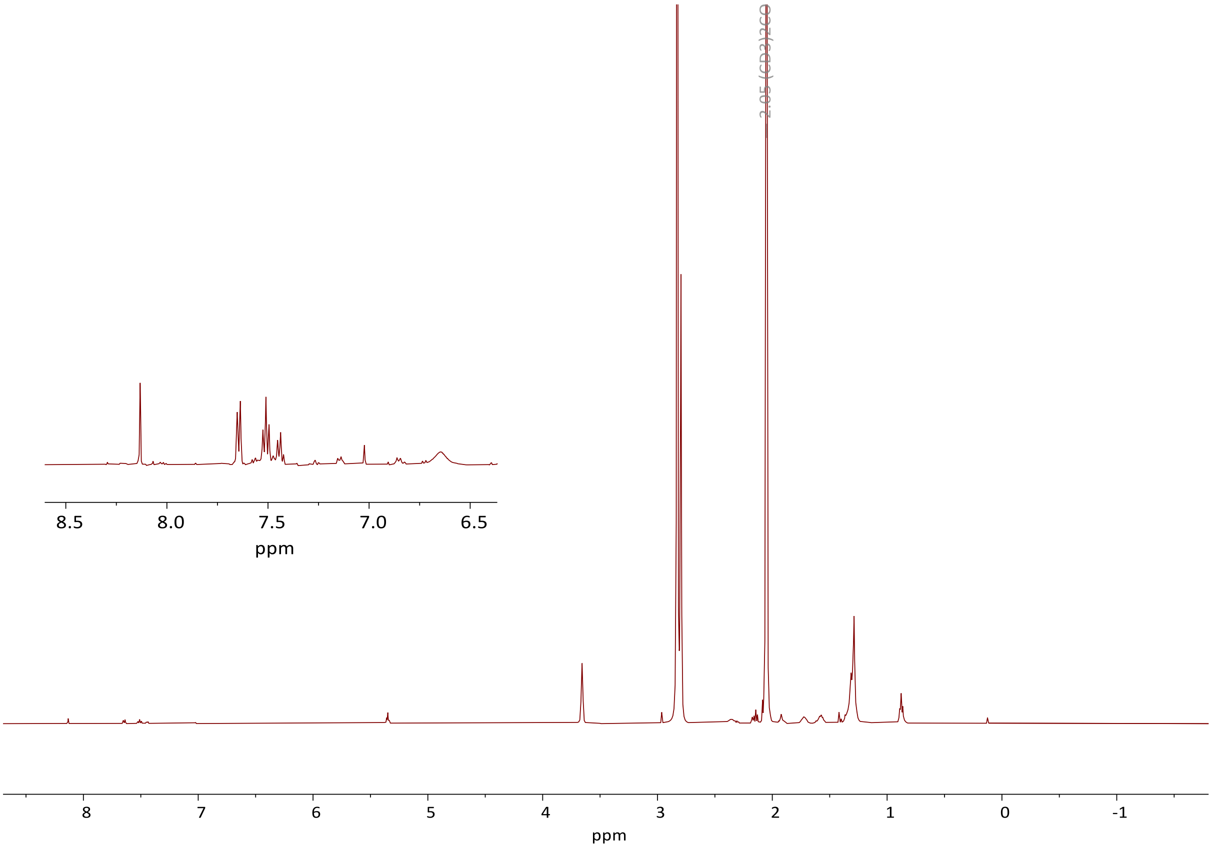

Spectrum S112. ^{1}H NMR (500 MHz, Acetone-d_6, 298 K) spectrum of the MeOH extract from post-sonication polymer **2**$_{cis}$.

8.3.25 Post-Sonication ^{1}H NMR Spectra of Polymer $\mathbf{2}_{cis}$ (Run 1 and run 2)

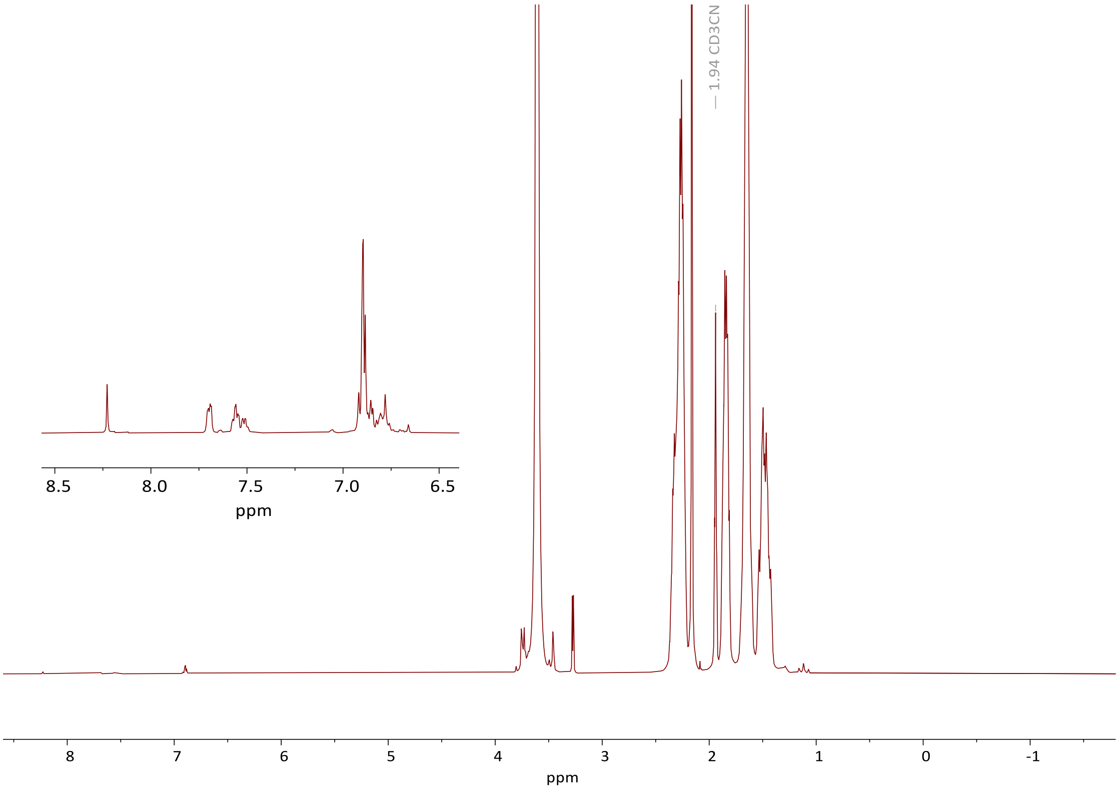

Spectrum S113. ^{1}H NMR (500 MHz, Acetonitrile-d_6, 298 K) spectrum of post-sonication polymer $\mathbf{2}_{cis}$ after being washed with methanol.

8.3.26 Post-Sonication ^{1}H NMR Spectra of Polymer S20

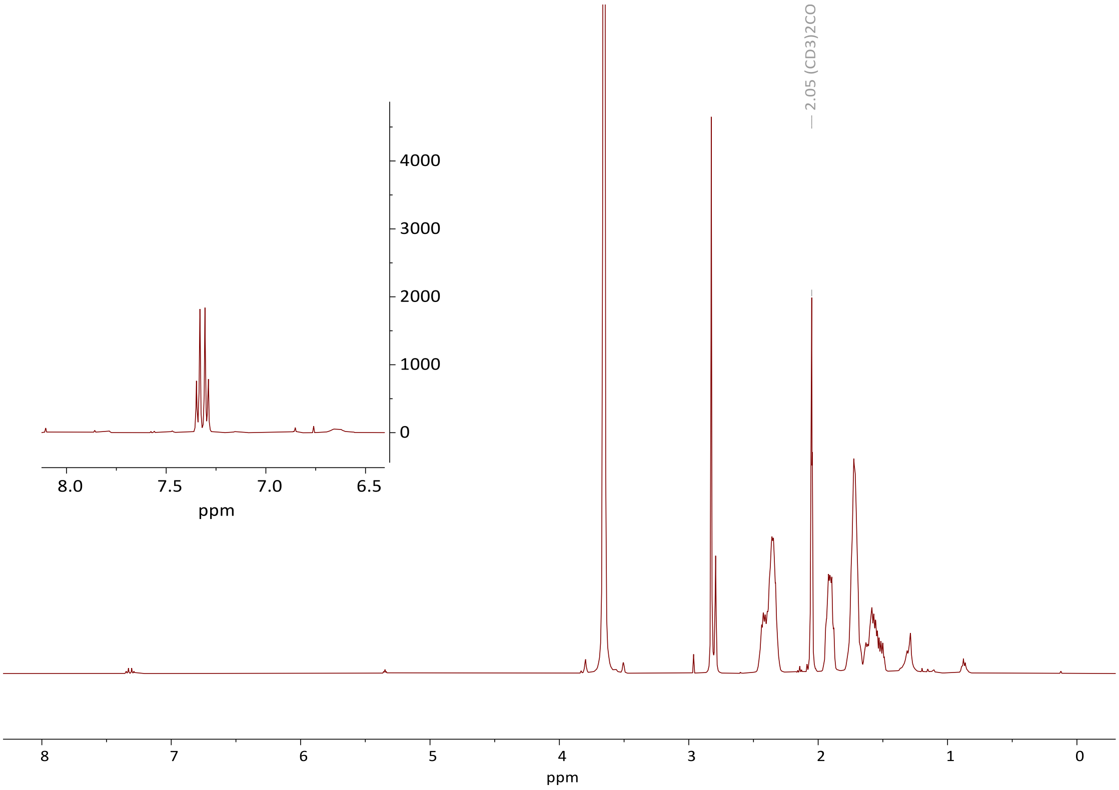

Spectrum S114. ^{1}H NMR (500 MHz, Acetone-d_6, 298 K) spectrum of post-sonication polymer **S20** before being washed with methanol.

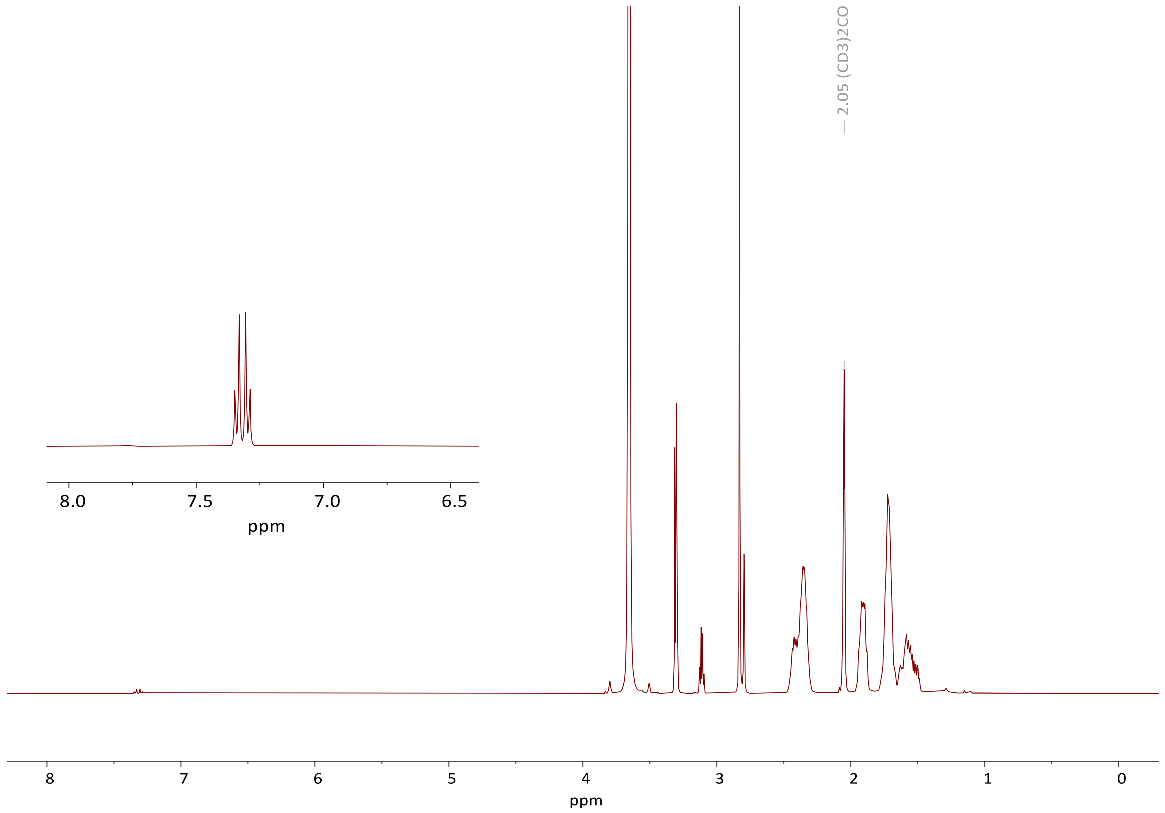

Spectrum S115. ^{1}H NMR (500 MHz, Acetone-d_6, 298 K) spectrum of post-sonication polymer **S20** after being washed with methanol.

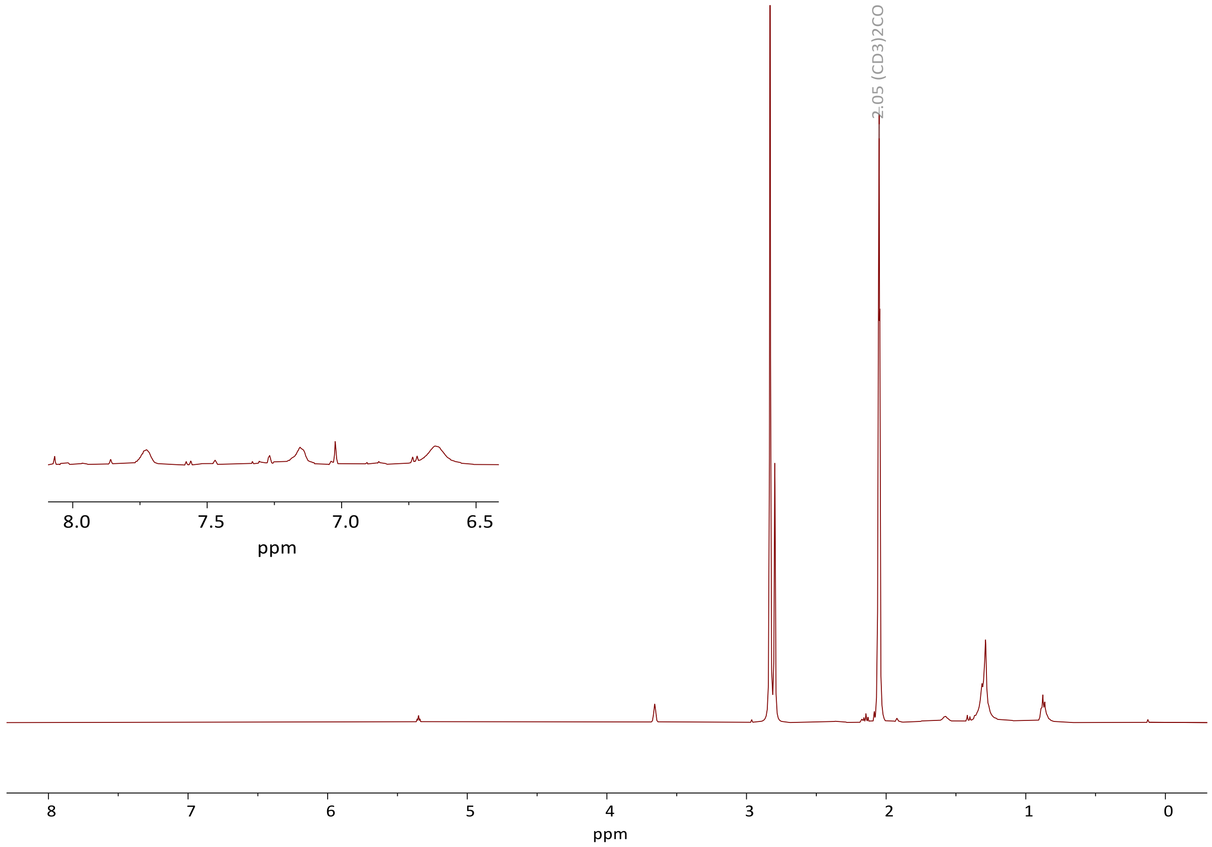

Spectrum S116. ^{1}H NMR (500 MHz, Acetone-d_6, 298 K) spectrum of the MeOH extract from post-sonication polymer **S20**.

8.3.27 Post-Sonication ^{1}H NMR Spectra of Polymer S21

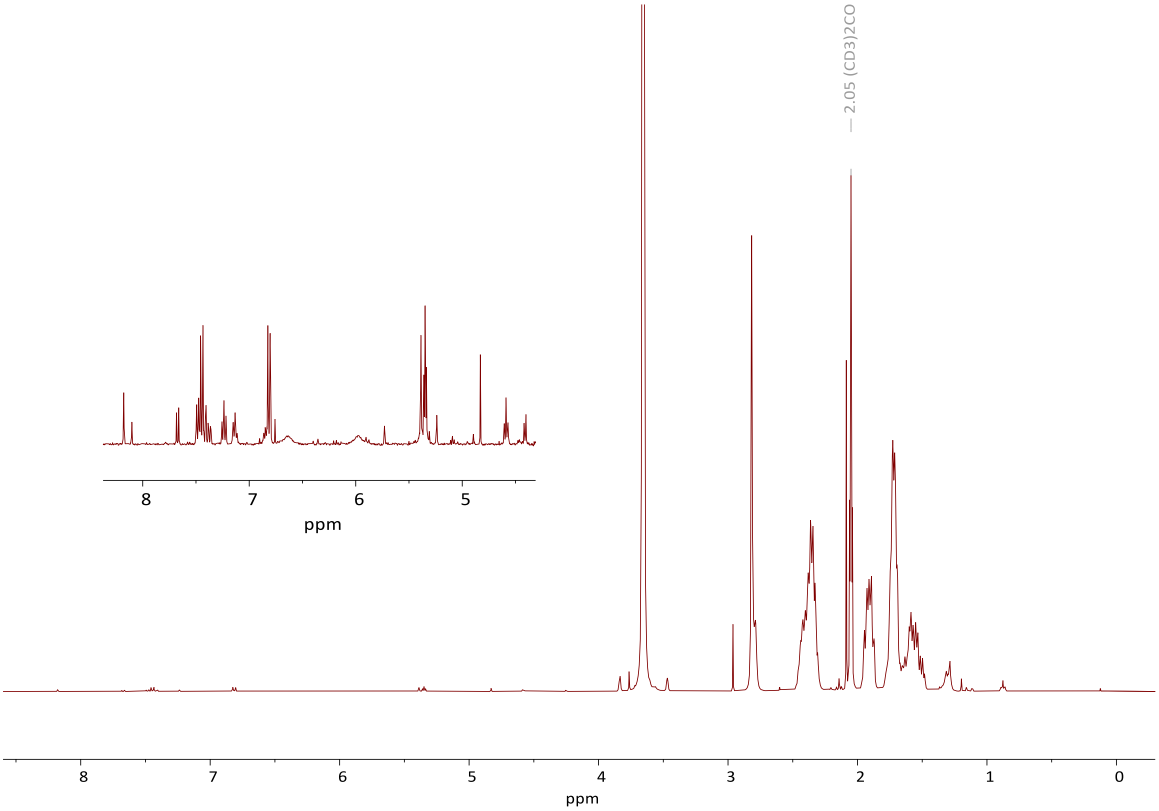

Spectrum S117. ^{1}H NMR (400 MHz, Acetone-d_6, 298 K) spectrum of post-sonication polymer **S21** before being washed with methanol.

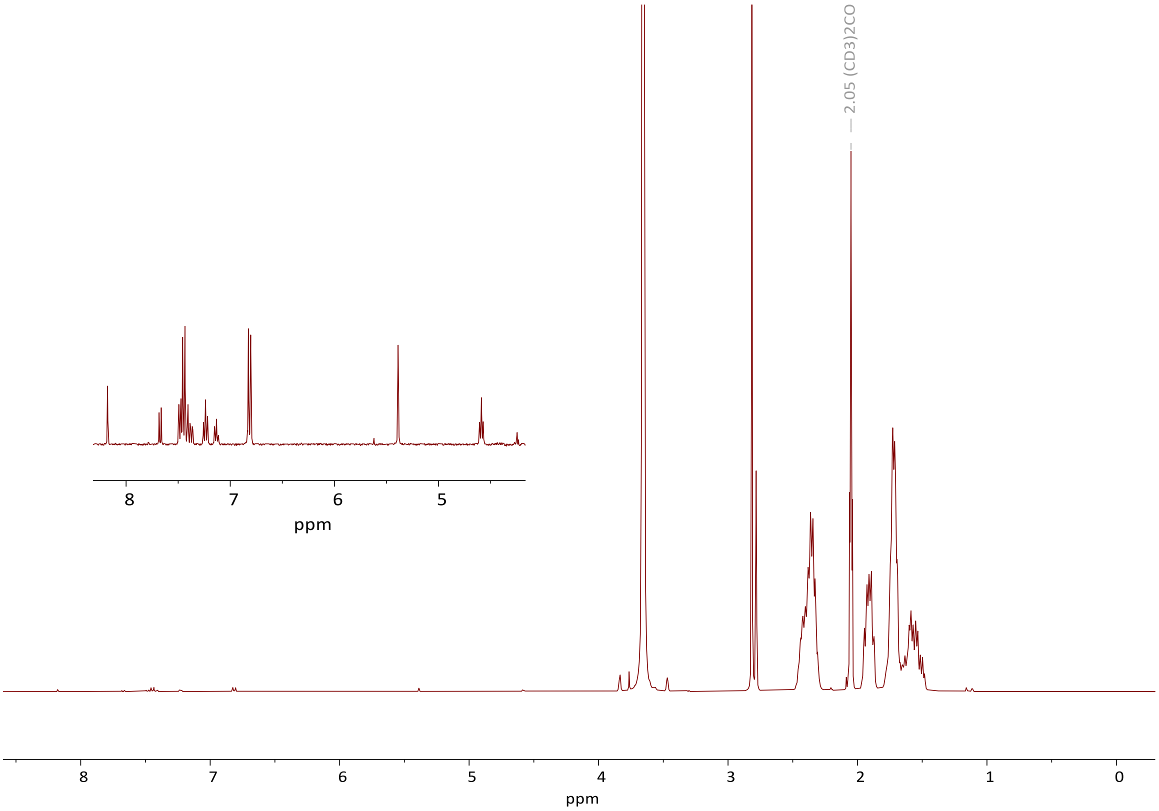

Spectrum S118. ^{1}H NMR (400 MHz, Acetone-d_6, 298 K) spectrum of post-sonication polymer **S21** after being washed with methanol.

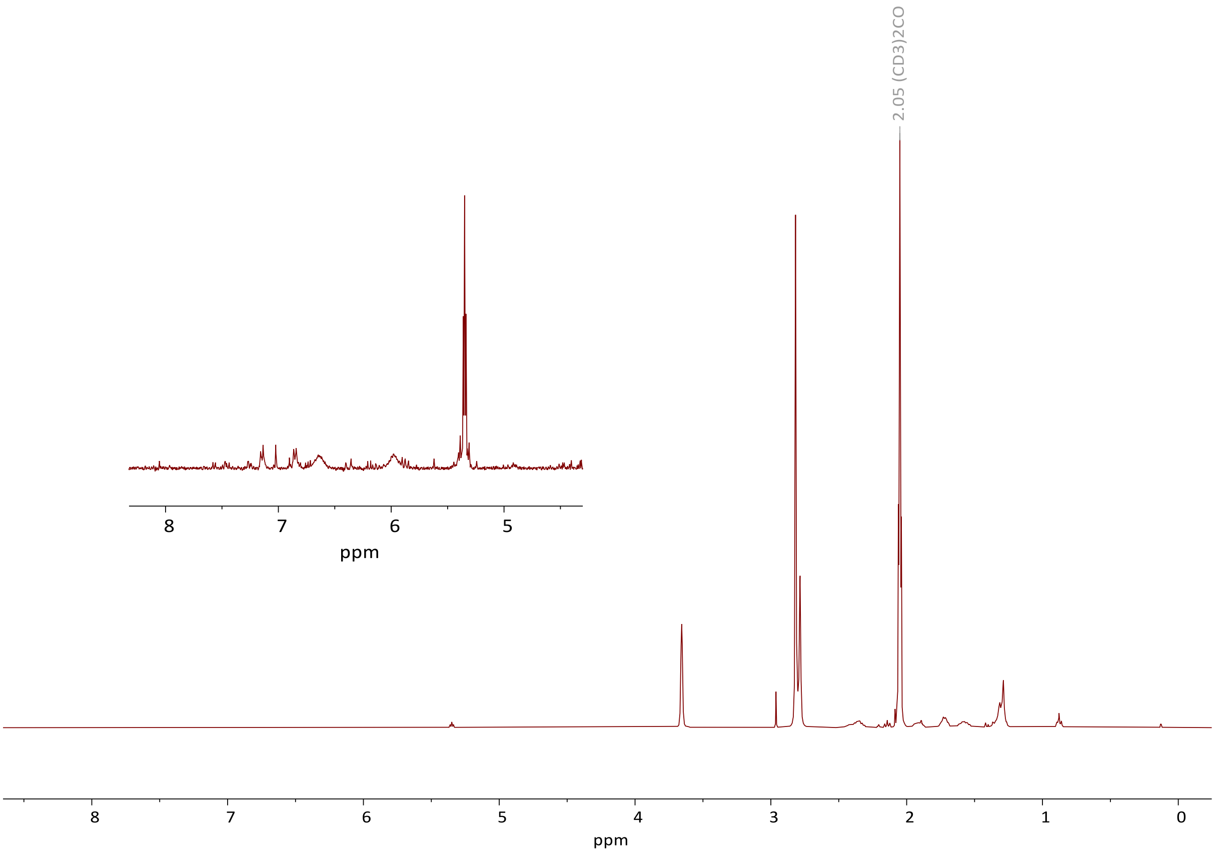

Spectrum S119. ^1H NMR (400 MHz, Acetone-d_6, 298 K) spectrum of the MeOH extract from post-sonication polymer **S21**.

9 Mass Spectrometry Isotopic Patterns

9.1 Isotopic distribution of S2

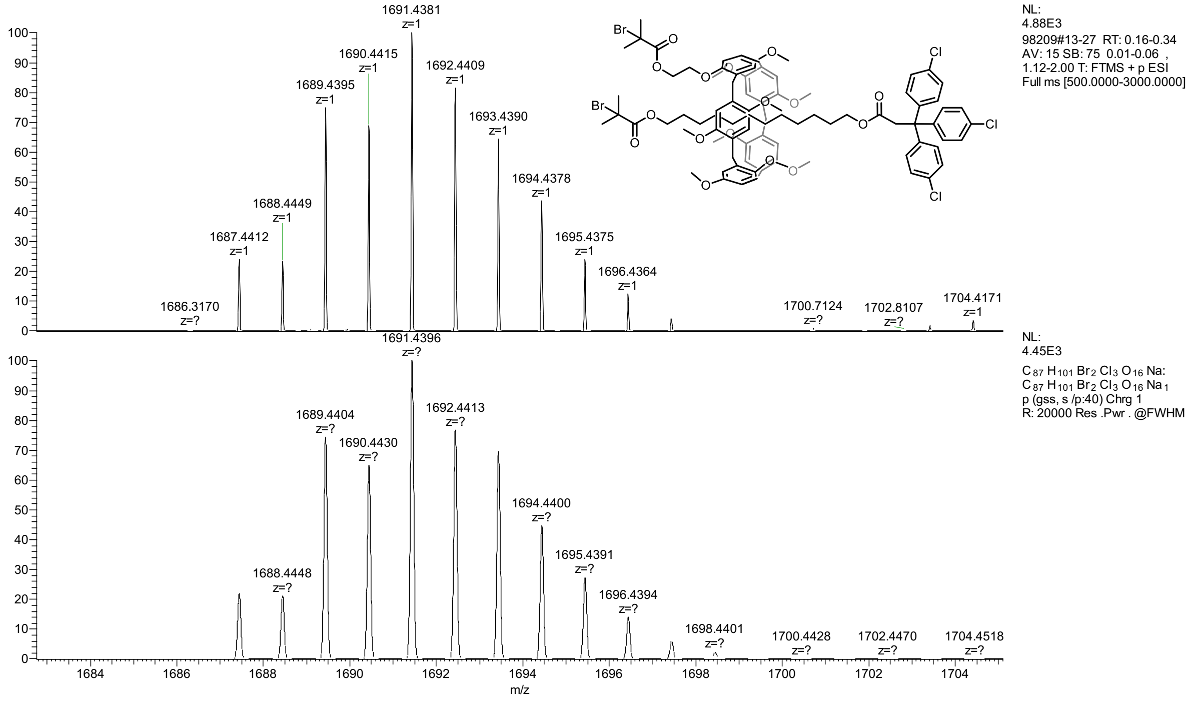

Spectrum S120. Isotopic distribution of **S2**. Top: Measured isotopic distribution for $C_{87}H_{101}Br_2Cl_3O_{16}Na$ ([M+Na]$^+$, +ESI). Bottom: Simulated isotopic distribution for $C_{87}H_{101}Br_2Cl_3O_{16}Na^+$.

9.2 Isotopic distribution of S4

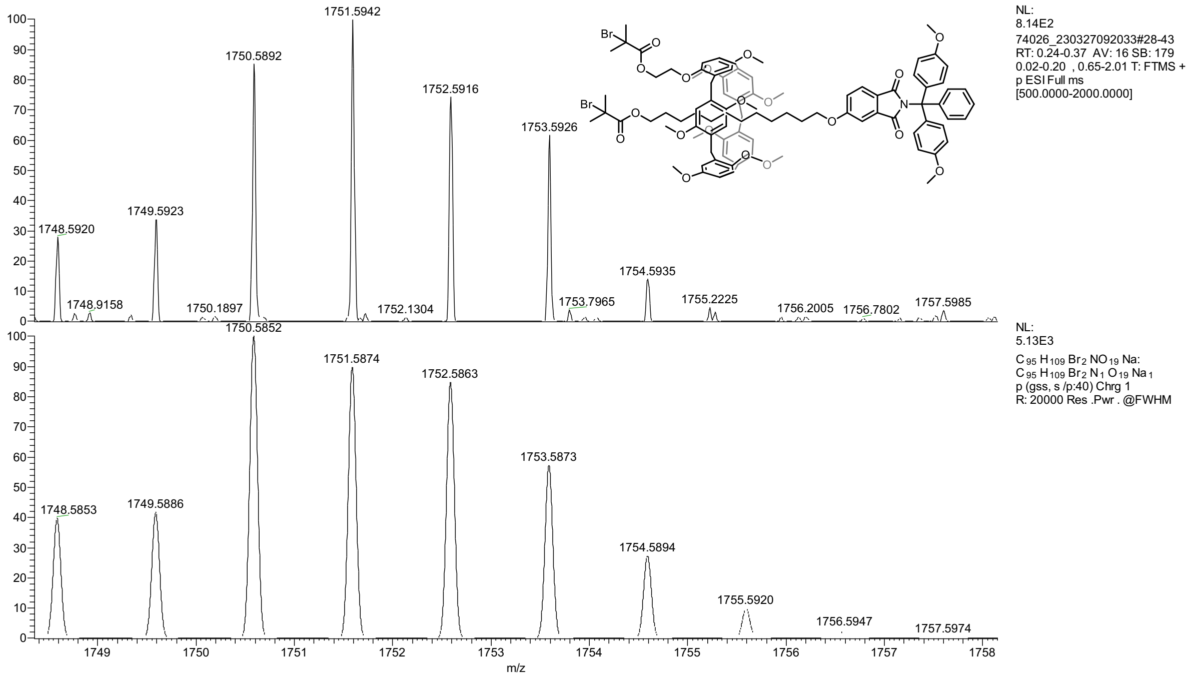

Spectrum S121. Isotopic distribution of **S4**. Top: Measured isotopic distribution for $C_{95}H_{109}Br_2NO_{19}Na$ ([M+Na]$^+$, +ESI). Bottom: Simulated isotopic distribution for $C_{95}H_{109}Br_2NO_{19}Na^+$.

9.3 Isotopic distribution of S6

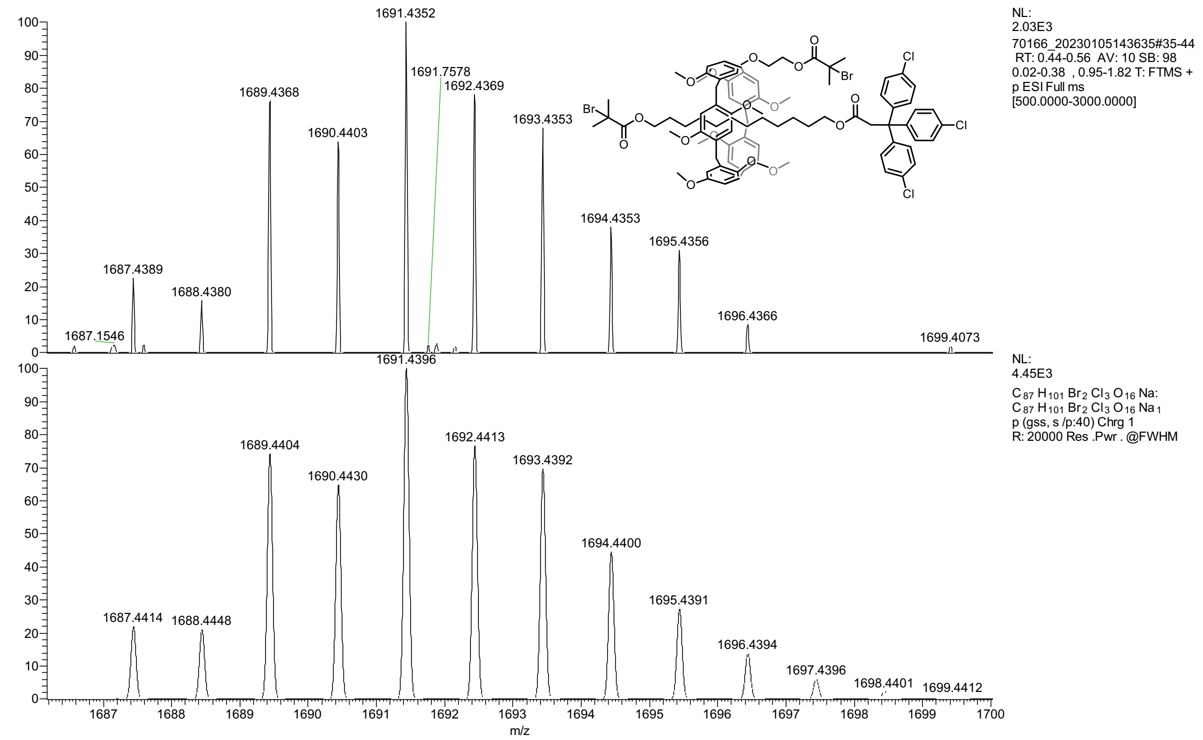

Spectrum S122. Isotopic distribution of **S6**. Top: Measured isotopic distribution for $C_{87}H_{101}Br_2Cl_3O_{16}Na$ ([M+Na]$^+$, +ESI). Bottom: Simulated isotopic distribution for $C_{87}H_{101}Br_2Cl_3O_{16}Na^+$.

9.4 Isotopic distribution of S16

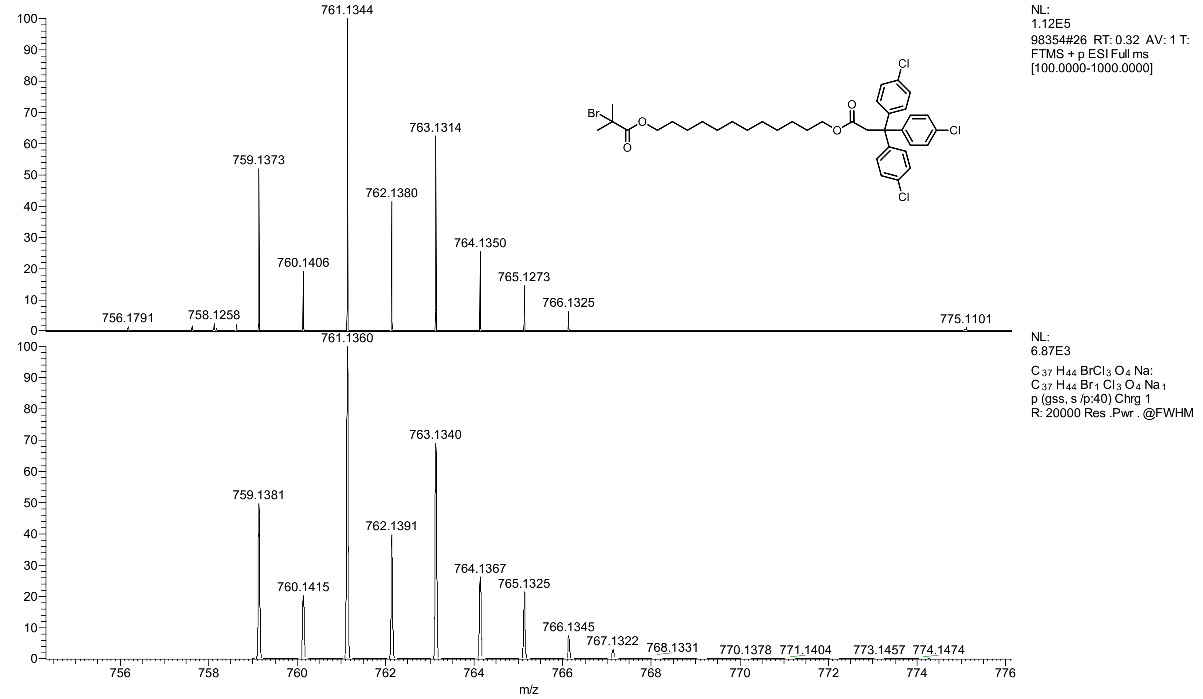

Spectrum S123. Isotopic distribution of **S16**. Top: Measured isotopic distribution for $C_{37}H_{44}BrCl_3O_4Na$ ([M+Na]$^+$, +ESI). Bottom: Simulated isotopic distribution for $C_{37}H_{44}BrCl_3O_4Na^+$.

9.5 Isotopic distribution of S9

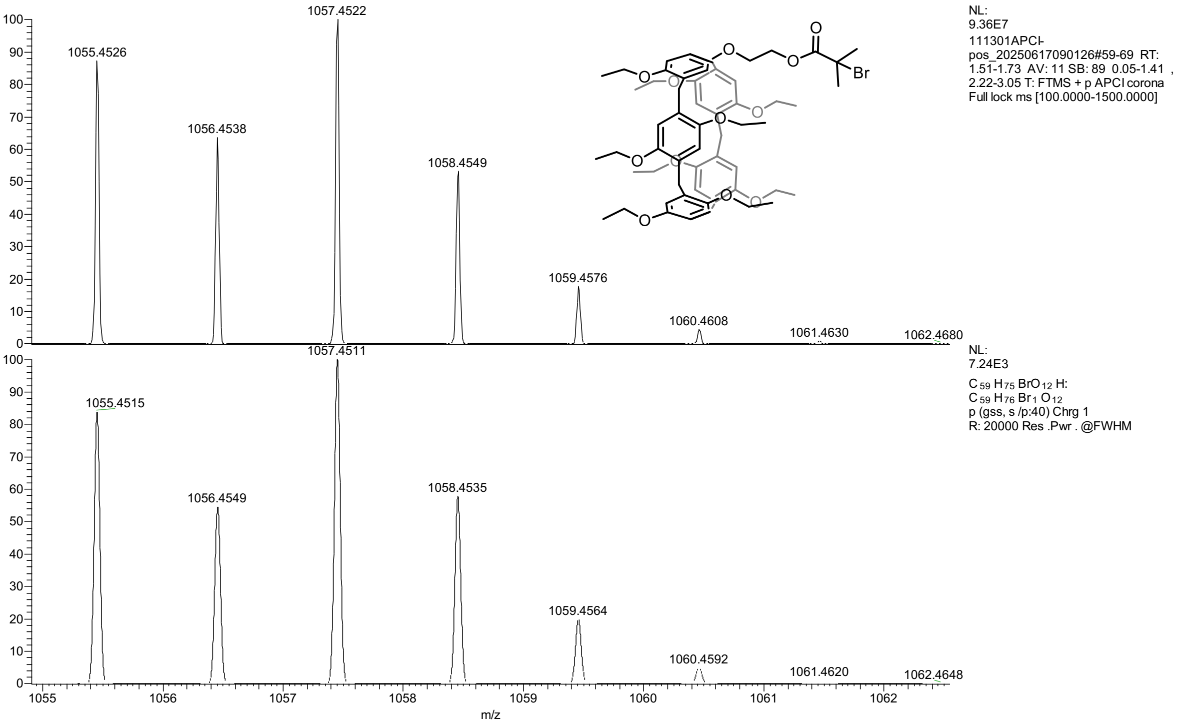

Spectrum S124. Isotopic distribution of **S9**. Top: Measured isotopic distribution for $C_{59}H_{75}BrO_{12}H$ ($[M+H]^+$, +APCI). Bottom: Simulated isotopic distribution for $C_{59}H_{75}BrO_{12}H^+$.

9.6 Isotopic distribution of S11

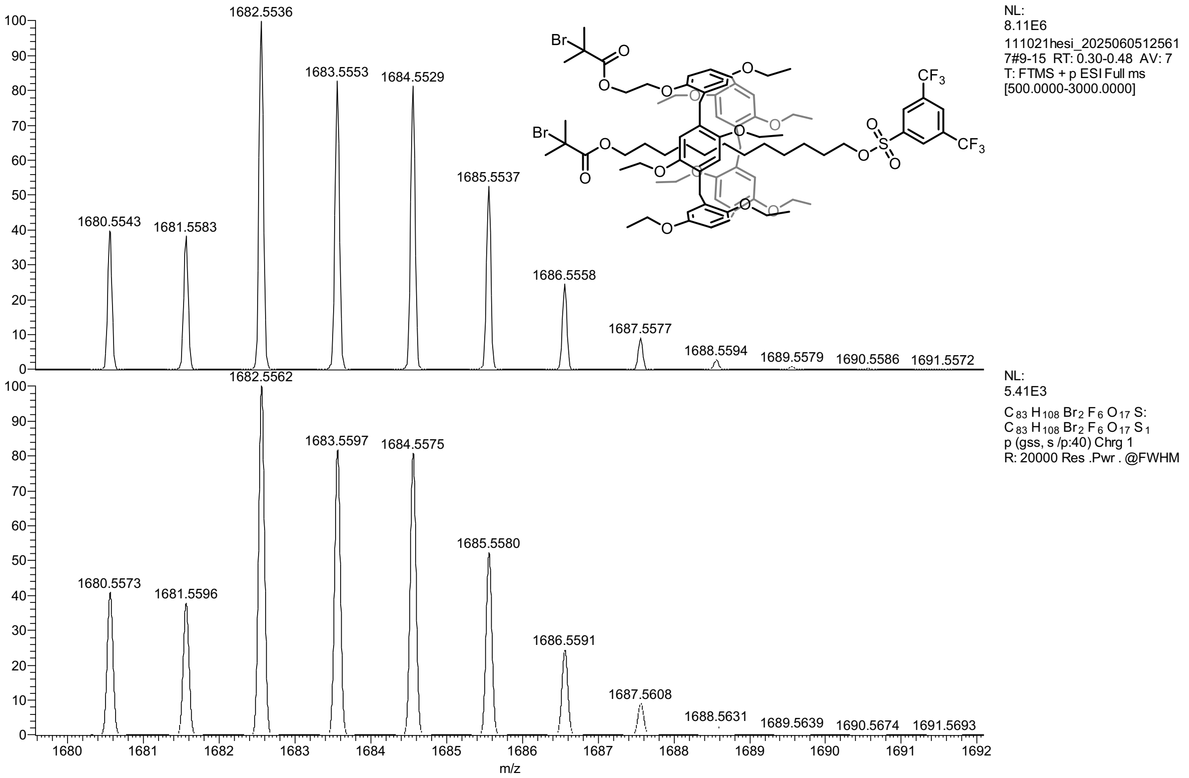

Spectrum S125. Isotopic distribution of **S11**. Top: Measured isotopic distribution for $C_{83}H_{108}Br_2F_6O_{17}S$ ([M-electron]$^+$, +ESI). Bottom: Simulated isotopic distribution for $C_{83}H_{108}Br_2F_6O_{17}S^+$.

9.7 Isotopic distribution of S12

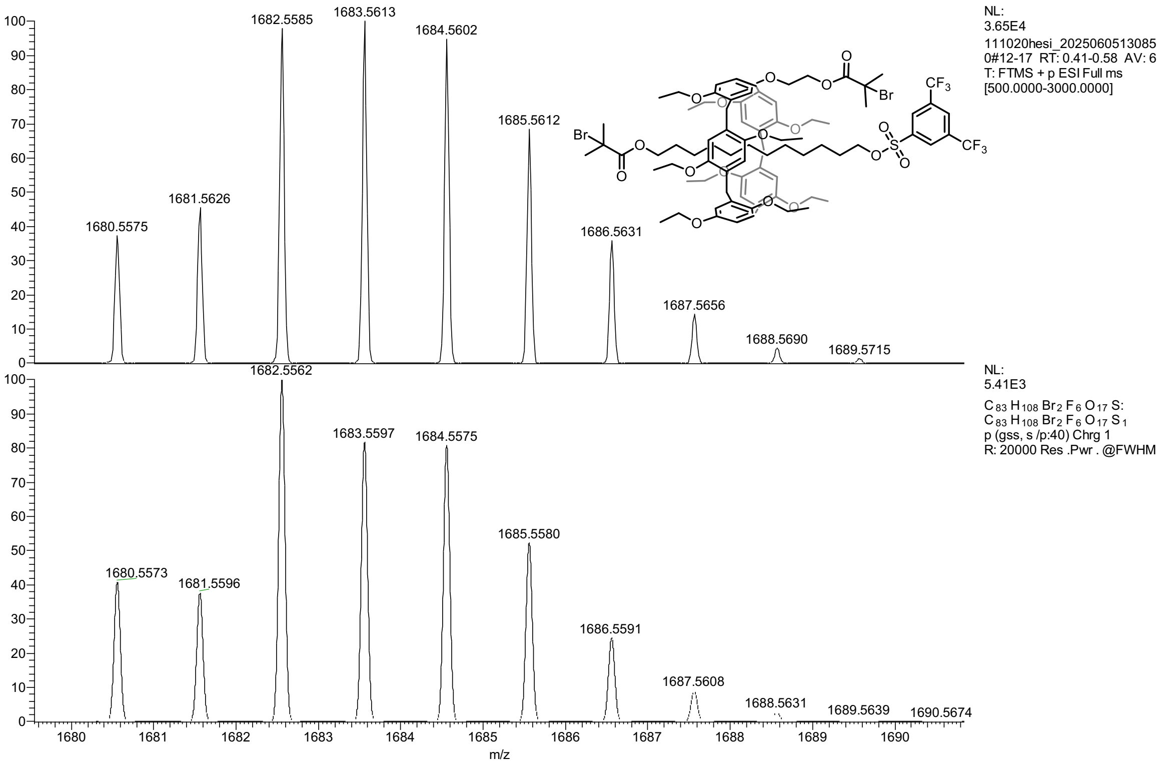

Spectrum S126. Isotopic distribution of **S12**. Top: Measured isotopic distribution for $C_{83}H_{108}Br_2F_6O_{17}S$ ([M-electron]$^+$, +ESI). Bottom: Simulated isotopic distribution for $C_{83}H_{108}Br_2F_6O_{17}S^+$.

9.8 Isotopic distribution of S13

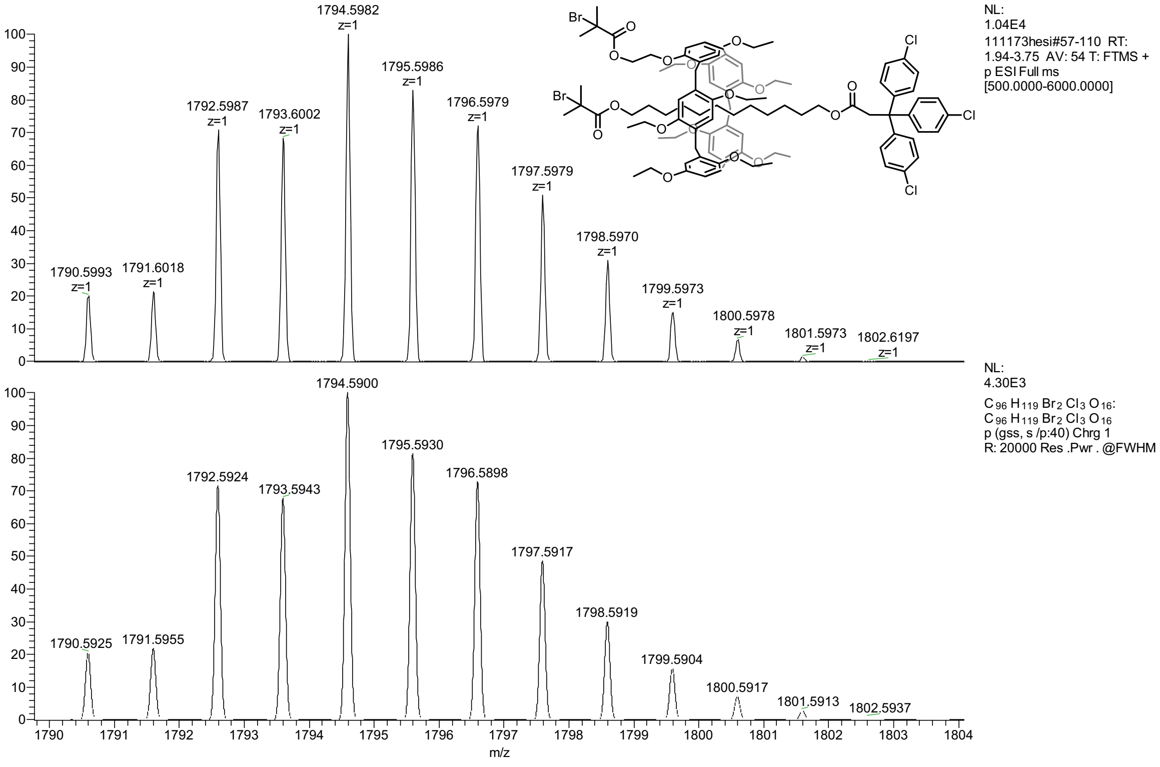

Spectrum S127. Isotopic distribution of **S13**. Top: Measured isotopic distribution for $C_{96}H_{119}Br_2Cl_3O_{16}$ ([M-electron]$^+$, +ESI). Bottom: Simulated isotopic distribution for $C_{96}H_{119}Br_2Cl_3O_{16}^+$.

9.9 Isotopic distribution of S14

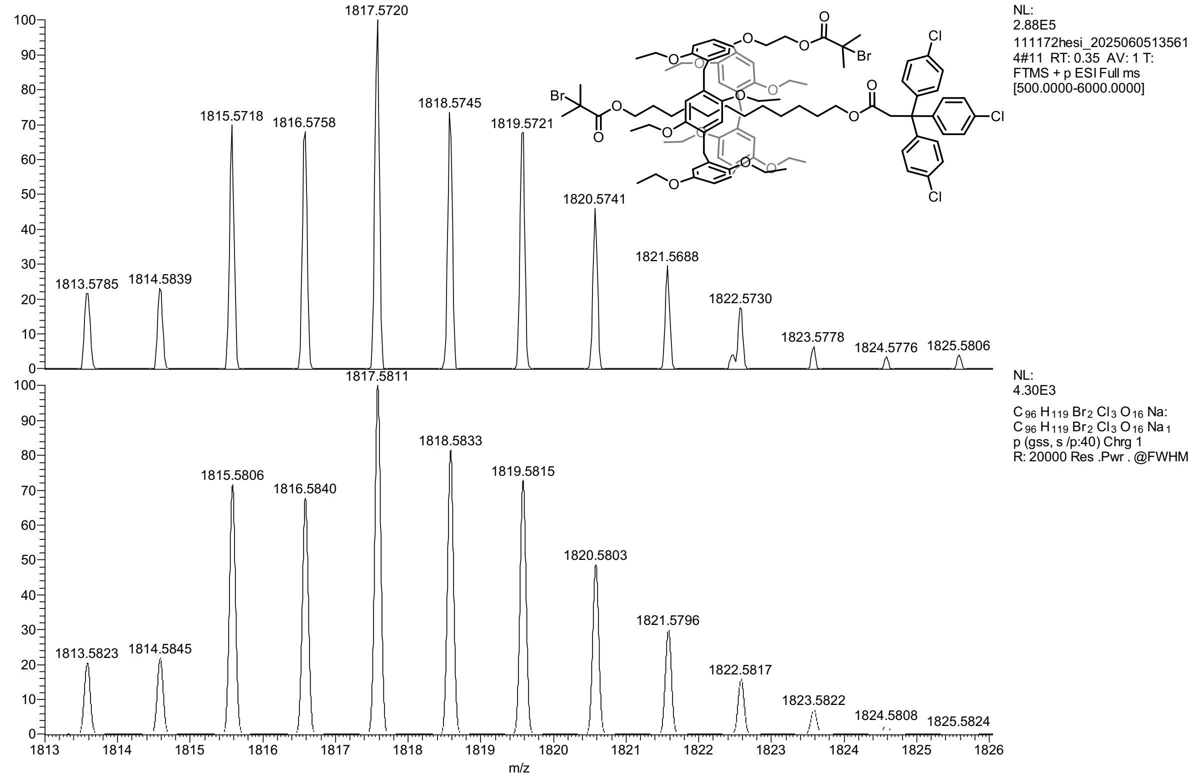

Spectrum S128. Isotopic distribution of **S14**. Top: Measured isotopic distribution for $C_{96}H_{119}Br_2Cl_3O_{16}Na$ ($[M+Na]^+$, +ESI). Bottom: Simulated isotopic distribution for $C_{96}H_{119}Br_2Cl_3O_{16}Na^+$.

10 References

(1) Chen, L.; Nixon, R.; De Bo, G. *Nature* **2024**, *628*, 320-325.

(2) Yao, Y.; Wei, X.; Cai, Y.; Kong, X.; Chen, J.; Wu, J.; Shi, Y. *J. Colloid Interface Sci.*, **2018**, *525*, 48-53.

(3) Hickenboth, C. R.; Moore, J. S.; White, S. R.; Sottos, N. R.; Baudry, J.; Wilson, S. R. *Nature* **2007**, *446*, 423-427.

(4) Zhang, C.; Huang, Z.; Lu, J.; Luo, N.; Wang, F. *J. Am. Chem. Soc.* **2018**, *140*, 2032-2035.

(5) Tian, Z.; Wang, A.; Hou, T. ; Huang, J.; Cao, S. ; Zhou, W.; Qian, J; Cui, J.; He, M. *Appl. Catal. A Gen.* **2024**, *677*, 119692.

(6) Sato, T.; Nalepa, D. E. *J. Appl. Polym. Sci.* **1978**, *22*, 865-867.

(7) Beyer, M. K. *J. Chem. Phys.* **2000**, *112*, 7307-7312.

(8) Bussi, G., Donadio, D., & Parrinello, M. *J. Chem. Phys.* **2007**, 126, 014101.

(9) Ribas‐Arino, J. ; Shiga, M. ; Marx, D. *Angew. Chem. Int. Ed.* **2009**, *48*, 4190–4193.
